# Deep RNA sequencing analysis of readthrough gene fusions in human prostate adenocarcinoma and reference samples

**DOI:** 10.1186/1755-8794-4-11

**Published:** 2011-01-24

**Authors:** Serban Nacu, Wenlin Yuan, Zhengyan Kan, Deepali Bhatt, Celina Sanchez Rivers, Jeremy Stinson, Brock A Peters, Zora Modrusan, Kenneth Jung, Somasekar Seshagiri, Thomas D Wu

**Affiliations:** 1 Departments of Bioinformatics and Molecular Biology, Genentech, Inc., South San Francisco, California 94080, USA

## Abstract

**Background:**

Readthrough fusions across adjacent genes in the genome, or transcription-induced chimeras (TICs), have been estimated using expressed sequence tag (EST) libraries to involve 4-6% of all genes. Deep transcriptional sequencing (RNA-Seq) now makes it possible to study the occurrence and expression levels of TICs in individual samples across the genome.

**Methods:**

We performed single-end RNA-Seq on three human prostate adenocarcinoma samples and their corresponding normal tissues, as well as brain and universal reference samples. We developed two bioinformatics methods to specifically identify TIC events: a targeted alignment method using artificial exon-exon junctions within 200,000 bp from adjacent genes, and genomic alignment allowing splicing within individual reads. We performed further experimental verification and characterization of selected TIC and fusion events using quantitative RT-PCR and comparative genomic hybridization microarrays.

**Results:**

Targeted alignment against artificial exon-exon junctions yielded 339 distinct TIC events, including 32 gene pairs with multiple isoforms. The false discovery rate was estimated to be 1.5%. Spliced alignment to the genome was less sensitive, finding only 18% of those found by targeted alignment in 33-nt reads and 59% of those in 50-nt reads. However, spliced alignment revealed 30 cases of TICs with intervening exons, in addition to distant inversions, scrambled genes, and translocations. Our findings increase the catalog of observed TIC gene pairs by 66%.

We verified 6 of 6 predicted TICs in all prostate samples, and 2 of 5 predicted novel distant gene fusions, both private events among 54 prostate tumor samples tested. Expression of TICs correlates with that of the upstream gene, which can explain the prostate-specific pattern of some TIC events and the restriction of the *SLC45A3-ELK4 *e4-e2 TIC to *ERG*-negative prostate samples, as confirmed in 20 matched prostate tumor and normal samples and 9 lung cancer cell lines.

**Conclusions:**

Deep transcriptional sequencing and analysis with targeted and spliced alignment methods can effectively identify TIC events across the genome in individual tissues. Prostate and reference samples exhibit a wide range of TIC events, involving more genes than estimated previously using ESTs. Tissue specificity of TIC events is correlated with expression patterns of the upstream gene. Some TIC events, such as *MSMB-NCOA4*, may play functional roles in cancer.

## Background

Readthrough gene fusions, or transcription-induced chimeras (TICs), occur when consecutive genes on a genome strand are spliced together. Their existence was first reported experimentally in isolated cases [1-4], and later surveyed computationally using analyses of expressed sequence tags (ESTs). Two different EST-based studies have been carried out to date. In one study [5], researchers clustered ESTs and then aligned these clusters to the genome, looking for alignments that crossed gene boundaries. The other study [6] involved identification of potential tandem gene pairs and sought ESTs that spanned both genes in a pair. These studies indicate that at least 4-6% of genes in the genome may be involved in TIC formation, although their prevalence was found to be generally low.

Nevertheless, in some cases, TICs appear to be expressed highly and generate functional protein products, with possible implications in cancer. For example, the *HHLA1-OC90 *TIC is expressed highly in teratocarcinoma cell lines [7], while the *CD205-DCL1 *TIC is expressed in Hodgkin lymphoma cell lines [8]. A TIC between the oncogene *RBM14 *and *RBM4 *generates a fusion protein called transcriptional coactivator *CoAZ *[9]. Another TIC between *RBM6 *and *RBM5 *is found in several cancer tissues and cell lines, but not in non-tumor tissues, and is associated with larger breast tumor sizes [10]. Likewise, a TIC between exon 4 of *SLC45A3 *and exon 2 of *ELK4 *found in prostate adenocarcinomas [11] has an erythroblast transformation-specific (ETS) oncogene family member as its downstream gene. In addition to the e4-e2 TIC isoform, which was observed specifically in *ERG*-negative prostate cancer samples, another isoform e1-e2 has been observed in both prostate cancer and benign prostate tissue, and found to be regulated by androgen levels [12].

Although EST-based studies have identified over 300 distinct TIC events so far, these events are spread over the multiple RNA libraries from which the ESTs were derived. Accordingly, an EST-based study cannot reveal the extent or diversity of TIC occurrences in an individual sample. The ability to study TICs in a single sample would facilitate the discovery of associations between TIC events and phenotypic traits, such as propensity for particular cancer types or other diseases, or sensitivity to specific treatments.

One clue to the occurrence of TIC events within samples comes from RACE (rapid amplification of cDNA ends) studies, in which specifically targeted transcript regions are extended upstream (5' RACE) or downstream (3' RACE) and then aligned to genomic tiling arrays to reveal their gene structure [13]. In one large-scale 5' RACE study covering 1% of the genome targeted by the Encyclopedia of DNA Elements (ENCODE) project across 12 human tissues and 3 cell lines, an upstream extension indicative of a TIC event was found in 136 of the 410 loci studied [14].

Because RACE can assay only specific transcripts selected in advance, it can miss TIC events that may predominate or have functional relevance in a particular sample. In contrast, a genome-wide study of transcriptional phenomena in individual tissues is now possible with the recent advent of deep, or next-generation, sequencing technology. Such technology provides a sampling of the entire range of transcriptional phenomena in single tissues, by generating large volumes of short reads of 30-100 nt [15]. However, analyzing such RNA-Seq data to study TICs poses its own set of unique challenges. Although many studies to date have analyzed RNA-Seq data for the tasks of expression, sequence polymorphisms, and even gene fusions in general, none so far have tried to specifically detect TIC events, and previous studies have reported relatively few such events. Four TICs were reported in targeted sequencing analysis of K562 [16]. Another study of the VCaP and K562 cell lines and the HBR and UHR samples using paired-end reads reported 76 fusion events [17], of which 23 appear to be TICs. A recent study of 25 prostate cancer samples [18] using an algorithm called FusionSeq [19] identified 11 readthrough fusion candidates and experimentally verified 9 of them.

In this study, we explore two different methods for detecting TICs in RNA-Seq data with high sensitivity. One method involves a targeted alignment approach where reads are aligned to a set of artificial exon-exon target sequences constructed in advance. Such a targeted alignment strategy has been highly effective in studying the extent of intragenic alternative splicing in the human genome, even in short reads of only 32 nt [20-23]. But for intergenic splicing events in general, targeted alignment is not applicable as a computational strategy, because it is infeasible to generate all possible exon-exon pairs over the human genome. In this paper, we demonstrate that a targeted alignment approach is nevertheless well suited for sensitive detection of TIC events across the universe of possible exon-exon pairs of this type.

Our other method is to align the reads to a reference genome using a program that can split an individual alignment to different locations in a genome. Various such alignment tools or pipelines have been developed for detecting spliced reads in short read data within local regions of a genome, including QPALMA [24] and TopHat [25]. Other recent programs, including SplitSeek [26] and our own program GSNAP [27], provide the additional capability of finding splicing events involving genes from distant or interchromosomal locations in a genome. The spliced alignment approach is more general, because it can identify novel or distant gene fusions not enumerable by a targeted alignment approach. However, it is less sensitive, especially when reads are very short, because it requires enough material on both sides of the exon-exon junction for accurate alignment. For example, GSNAP requires at least 14 nt on both sides of the exon-exon junction to to find a novel spliced alignment, without any further assistance, such as a user-provided database of known splice sites. In most cases, at least 20 nt are required to uniquely identify a genomic location in the unmasked part of the human genome [28], and even more if mismatches, SNPs, or indels are allowed. Therefore, spliced alignment is generally effective only for reads having exon-exon junctions in their middle regions, away from the 14-20 nt margins at their ends.

Another strategy that is applicable when paired-end reads are available is to align the two ends separately and look for cases where the two ends align to different gene transcripts. Studies suggest that a paired-end strategy has greater sensitivity for finding gene fusions [17], and the FusionSeq algorithm [19] is based on paired-end reads. However, the single-end data in our study precluded this approach.

In this paper, we show that both the targeted alignment and spliced alignment approaches can be used in complementary ways to study TICs and gene fusions in individual cancer and normal samples assayed by deep transcriptional sequencing. We applied both methods to sets of single-end reads that we obtained by sequencing the transcriptomes of three primary human prostate adenocarcinomas (denoted by T1, T2, and T3) and their matched normal samples (N1, N2, and N3), as well as the human brain reference (HBR) and universal human reference (UHR) samples used in the Microarray Quality Control (MAQC) project [29,30]. One of the adenocarcinomas, T1, is ETS-negative, and the remaining adenocarcinomas are ETS-positive, with the positive expression of an ETS family member conferred by a *TMPRSS2-ERG *gene fusion [31]. We also implemented filtering methods that are necessary to remove possible false positive alignments due to gene families or other homologous genes. After filtering, we were still left with sequence-based support for a large number of TIC events among our samples, which afforded us an opportunity to further characterize the phenomenon.

RNA-Seq data can provide not only splicing information but also information about expression levels, which can help us understand the expression patterns of TICs. Although experimental evidence indicates that some TICs are expressed ubiquitously over different tissues, while others are expressed specifically in particular tissues [6], the mechanism for these varying expression patterns has not been well studied.

Expression information can also provide clues about the mechanism of TIC formation. The prevailing hypothesis is that TIC events represent a type of transcriptional "leakage," in which termination of transcription fails for the upstream, or 5^' ^gene, resulting in the two adjacent 5^' ^and 3^' ^genes existing on a single transcript [6]. The splicing machinery then acts on this transcript to give rise to the TIC. However, in contrast to this *cis*-mechanism, some evidence has pointed to a *trans*-mechanism, where genes on two separate transcripts are spliced together. The *trans*-mechanism has been demonstrated both for genomically distant genes [32] and for adjacent genes involved in TICs [9]. We therefore seek evidence related to TIC formation by integrating expression and splicing analyses from our RNA-Seq data, and from supporting experiments.

## Results

### Targeted alignment method

Based on 27,157 well-annotated RefSeq transcript alignments to the human genome, we identified 2,470,383 exon-exon junctions between same-strand transcripts that spanned a potential intron of 200,000 bp or less. We also identified 1,856,519 possible intragenic exon-exon junctions by taking all pairs of exons within a given transcript, regardless of distance. Each of these junctions was extended by 80 nt on each side and used as targets for the alignment of reads. An alignment therefore supports a given exon-exon junction when it crosses the midpoint by a certain overhang. Longer overhang requirements provide greater specificity, while shorter ones are more sensitive.

Using an overhang of 11 nt, intragenic splicing was supported by 2-4% of 33-nt reads and by 6-11% of 50-nt reads (Table 1), showing that longer reads provided significantly more raw material for identifying splicing events. For TIC splicing, a threshold of 8 nt yielded a unique alignment to a TIC target in about 1 in 30,000 reads. At 11 nt, the frequency dropped to 1 per 40,000-120,000 reads. Overall, we found evidence for TIC splicing to be rare in RNA-Seq data.

**Table 1:** Results of alignment to intragenic and TIC targets

				Overhang 8			Overhang 11			
	Length	Total reads	Intragenic	Pct	TIC	Pct	Intragenic	Pct	TIC	Pct
T1	33	30,797,857	966,447	3.1	1329	.0043	655,926	2.1	338	.0011
T2	33	32,029,444	1,216,580	3.8	1104	.0034	826,518	2.7	272	.0008
T3	33	65,479,803	2,171,327	3.3	2499	.0038	1,476,928	2.3	860	.0013
N1	50,75	33,509,416	3,862,551	11.5	1229	.0037	3,391,849	10.1	925	.0027
N2	50	33,125,582	2,925,272	8.8	945	.0029	2,449,004	7.4	559	.0017
N3	33	30,987,067	1,190,231	3.8	1213	.0039	810,039	2.6	507	.0016
HBR	50	53,238,798	3,487,753	6.6	1704	.0032	2,930,589	5.5	960	.0018
UHR	50	59,561,348	5,301,691	8.9	2377	.0040	4,445,658	7.5	1335	.0022

To achieve sensitivity with such few reads, we started with the set of TIC alignments with an overhang of 8 nt, and used a clustering method to to obtain specificity. Clustering allowed a given TIC exon-exon junction to be supported by the entire collection of alignments in demonstrating a sufficient overhang. Before clustering, though, we filtered our set of TIC alignments to remove those that corresponded to intragenic alternate splicing events. These spurious TIC alignments arose because they spanned a particular splice combination across two RefSeq transcripts for the same gene that was not represented by any single RefSeq transcript. This filtering process eliminated 3702 (29%) out of 12,400 putative TIC alignments.

In the clustering process, we took the remaining 8698 TIC-aligning reads over all samples, grouped them according to their exon-exon junction, and created a multiple sequence alignment for the cluster, resulting in 608 clusters. To reduce the incidence of false positives due to poor alignments, we implemented a filtering method based on the consistency of matches and mismatches on both sides of the exon-exon junction, essentially requiring that at least one read have a match to the genome at all 11 bp on both sides of the junction. Our filtering criteria were designed to eliminate false alignments, but still accommodate sequencing errors, which can occur at a rate of 1% or more in short read data.

The filtering step eliminated 137 (23%) of the clusters to leave 471 TIC candidates supported by 3373 TIC alignments. However, the number of supporting reads was not evenly distributed over the different candidates. In particular, 2195 (65%) TIC alignments supported 12 candidates corresponding to various pairs of exons from the human leukocyte antigen *(HLA) *genes, namely, from *HLA-B *to *HLA-C *(7 intergenic splicing candidates), from *HLA-DRB1 *to *HLA-DRB3 *(1 candidate), and from *HLA-G *to *HLA-A *(4 candidates). Because these genes are highly polymorphic and share sequence similarity with one another (e.g., 92% sequence identity between *HLA-B *and *HLA-C)*, such TIC candidates most likely represent misalignment due to sequence errors or polymorphisms rather than true TIC events. Another example of likely false positives were 15 TIC candidates supported by 41 TIC alignments involving pairings of the metallothionein family members *MT1A, MT1B, MT1E, MT1F, MT1 H, MT1 M, MT1X, MT2A*, and *MT3*.

To eliminate such cases of false positives due to homologous genes, we implemented another filtering step based on sequence similarity among the TIC splice and its component 5' and 3' genes. Among the TIC candidates eliminated were 26 TIC candidates involving pairs of various zinc finger proteins; 7 TIC candidates involving pairs of keratins *KRT5, KRT6A, KRT8, KRT14, KRT17, KRT31, KRT32, KRT76, KRT77, KRT78, KRT81*, and *KRT83*; and 4 TIC candidates involving pairs of kallikreins *KLK2, KLK3, KLK9*, and *KLK11*. Overall, our homology filtering step eliminated 32% of the TIC candidates to leave a final set of 339 TIC events supported by 822 alignments (Additional files 1 and 2).

To assess the false discovery rate (FDR) of our analysis pipeline, we modified our set of artificial exon-exon junctions by removing 5 nt from both sides of the junction, and performed the same alignment and filtering steps against these modified junctions. This method was previously used in determining an FDR rate for alternative splicing predictions [20]. This control experiment gave a total of 5 putative TIC candidates, yielding an estimated FDR rate of 5/339 = 1.5%.

Of the 822 surviving TIC-aligning reads, 459 (56%) came from the MAQC samples sequenced at 50-nt read lengths, 229 (28%) from the N1 and N2 samples sequenced at 50- and 75-nt read lengths, and the remaining 134 (16%) from the prostate samples sequenced at 33-nt read lengths. This skewed distribution is likely to be due largely to the utility of longer read lengths in detecting TICs, rather than the underlying frequency of TIC events in the samples.

Among the 339 TIC events, two-thirds (212) were supported by a single alignment and one-third (127) were supported by multiple alignments. The TIC events with the largest numbers of supporting reads were *PMF1-BGLAP *e4/5-e2/4 (55 reads), *AZGP1-GJC3 *e2/4-e2/2 (41 reads), *BPTF*-*KPNA2 *e9/30-e2/11 (23 reads), *RBM14-RBM4 *e1/3*-*e2/4 (18 reads), and *C15orf38-AP3S2 *e5/6*-*e2/6 (16 reads). (In this notation, we indicate the number of exons in the gene, so "e4/5" denotes the fourth exon out of five in the gene.)

**Figure 1:**
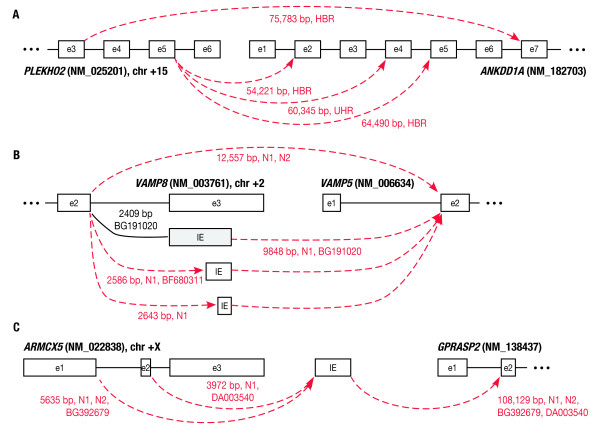
**Complex isoforms observed in transcription-induced chimeras**. TIC splicing events are shown by dashed arrows, labeled with splice distance and samples or ESTs with supporting alignments. Standard splicing is shown by solid lines. (A) Multiple isoforms observed for *PLEKHO2-ANKDD1A *TIC in the human brain reference (HBR) and universal human reference (UHR) samples. (B) Direct TIC splicing and TICs with multiple forms of intervening exons (labeled IE) for *VAMP8-VAMP5*, all observed in a single prostate sample N1. Shaded box represents an intervening exon found previously [5], but not in this study. (C) TIC with an intergenic exon between *ARMCX5 *and *GPRASP2*, all observed in N1.

The 339 TIC events showed several cases of multiple TIC isoforms across 302 distinct gene pairs. About 11%, or 32, of the gene pairs had multiple isoforms, with the pair *PLEKHO2-ANKDD1A *having 4 isoforms (Figure 1A), and three pairs, *KIAA1984-C9orf86, GCSH-C16orf46*, and *RBM14-RBM4*, each having three isoforms. In almost all cases of multiple isoforms, the isoforms had one splice site in common, suggesting the existence of a preference for that splice site. The finding of multiple TIC isoforms has been reported experimentally in isolated cases, but not in previous EST-based surveys, which were not designed to identify them.

### Comparison with existing databases

The AceView database [33] is intended to store all observed alternative splicing events in various genomes. Manual examination of our TIC events on the AceView Web site indicated that many are annotated as complex loci, those in which a protein product is generated from the fusion of the two genes, although the two genes may still have distinct expression. We performed a search of AceView for all of our TIC events and found that 88 (26%) were previously identified in that database. The 32 gene pairs with multiple isoforms were more often listed in AceView as complex loci, with 40% (13) having that annotation.

**Figure 2:**
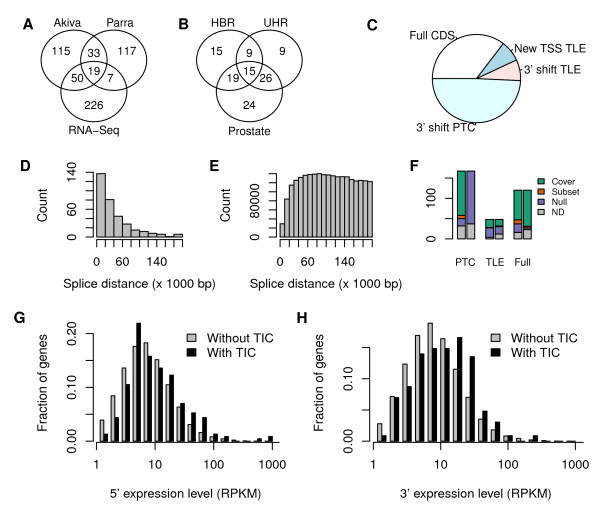
**Characteristics of TIC events**. (A) Comparison of TIC gene pairs found in previous EST-based surveys and those found by RNA-Seq in this study. (B) Distribution of TIC events across tissues. Only TIC events with multiple supporting reads are included. HBR = human brain reference, UHR = universal human reference. (C) Coding potential of TIC events. The label "Full CDS" indicates that the coding region (CDS) extends from the original transcription start site (TSS) of the 5' gene and to the original stop codon of the 3' gene; "3' shift" signifies a frameshift in the 3' gene; "New TSS" indicates that the TIC breakpoint occurs before the original TSS of the 5' gene and a new TSS is predicted from the longest open reading frame; "TLE" indicates that termination occurs in the last exon of the transcript; and "PTC" indicates premature termination codon, subjecting the transcript to nonsense-mediated decay. (D) Distribution of TIC splice distances. (E) Distribution of splice distances in the artificial exon-exon junctions. (F) Predicted effect on domains. Separate results are presented for TICs having a PTC, or having a TLE despite a new TSS or 3^' ^frameshift, or having a full CDS. Each pair of bars show the effect on the 5' (left) and 3' (right) domains. "ND" indicates that no domain was originally present in the 5^' ^or 3^' ^gene; "Null" indicates no intersection of the predicted TIC domains with the original domains; "Subset" indicates that at least one, but not all domains were preserved in the TIC; and "Cover" indicates all domains were preserved. (G) Distribution of expression levels in 5^' ^genes with observed TICs downstream compared to those without. (H) Distribution of expression levels in 3^' ^genes with observed TICs compared to those without. For panels F and G, distributions are taken over genes with at least one observed intragenic splice in a given sample and with a potential TIC exon within 200,000 bp in the downstream or upstream direction, respectively.

We compared our TIC events with the 212 EST-based events reported by Akiva and colleagues, and found that 37 fusion events (11%) were identified with the same splice sites, with another 39 gene pairs (12%) identified with a different pair of splice sites. In comparison with the 176 human TIC events reported by Parra and colleagues, these values were 14 (4%) and 16 (5%), respectively. A comparison among the EST-based surveys and our study at the gene pair level shows relatively little overlap among the three studies (Figure 2A). Therefore, our RNA-Seq study increases the catalog of gene pairs with observed TIC events by 66%.

We also scanned genomic alignments of all GenBank ESTs to find support for our TIC events, and found supporting ESTs for 100 events (29%), which covered 82 of those found in AceView plus an additional 18 events. In addition, 12 of our TIC events (4%) were also found by Maher and colleagues in their analysis of the HBR and UHR samples. A comparison of our findings with the FusionSeq-based prostate cancer study [18] showed that we had reads for 6 of their 11 candidates, including *VMAC-CAPS*, which was not otherwise supported by external evidence. However, one of their candidates, *ZNF649-ZNF577*, was removed by our conservative homology filtering step, because we found that the two genes share a region with 42 matches in a window of size 50. Altogether, 128 (38%) of our TIC events had support from another database or study. We noted a difference in support between our candidates that had a single read finding and those that had multiple reads, with external support for only 59 (28%) of the 212 single-read candidates, but for 69 (54%) of the 127 multiple-read candidates.

### Characteristics of TIC events

Among the 127 events supported by multiple alignments, 95 (74%) had reads from different samples. If we consider the prostate tumor and normal samples as a single tissue type, then 79 events (62%) had support from two or more different tissue sources in prostate, brain, and universal reference. The distribution of tissue sources among these multiply-supported TIC events supports a ubiquitous expression pattern for some events, with others that may potentially be brain- or prostate-specific (Figure 2B).

Distances of TIC splices showed an exponential distribution (Figure 2D), consistent with an earlier study [5]. Approximately one-fourth had distances of less than 12,000 bp, one-half less than 26,000 bp, and three-quarters less than 54,000 bp. These values appear larger than the median of 8500 bp reported in the previous study, but that study measured the distance between genes, rather than the distance between spliced exons. When we computed the intergenic distance for each TIC, we obtained a comparable median of 8866 bp. To rule out the possibility that the preference of TICs for shorter splice distances was due to our underlying set of artificial exon-exon junctions, we compiled a comparative distribution over the artificial splice distances (Figure 2E), which shows a much different distribution favoring longer distances uniformly above 40,000 bp.

TIC splices occurred predominantly between the last donor site of the 5' gene and the first acceptor site of the 3' gene. In our set of readthrough fusions, this splicing pattern between the *(n - *1) and +2 exon represented 54% of the cases, somewhat more than the 44% seen in a previous study [5]. Exons upstream of the (*n *- 1) exon spliced with the +2 exon 25% of the time, while the (*n *- 1) exon spliced with exons downstream of the +2 exon 10% of the time. Therefore, alternate choices were more likely to occur with the donor site than with the acceptor site. In 12% of the cases, a TIC event spliced both an upstream exon other than (*n *- 1) and a downstream exon other than +2.

To determine whether our TICs were likely to generate a functional protein, we computed a probable coding sequence (CDS) for each TIC (Figure 2C). For TIC breakpoints that occur after the transcription start site (TSS) of the 5' gene, we expect that the TSS should be preserved, serving as the start of the reading frame for the rest of the TIC transcript. Starting from the original TSS, the reading frame was preserved for the 3' gene in 120 cases (35%), ending at the original stop codon, and frameshifted in the other 193 cases (57%) with a post-TSS breakpoint. In the remaining 26 cases (8%) with a pre-TSS breakpoint, the TIC transcript must utilize a new TSS, in either the 5' or 3' gene, and we predicted the TSS in such cases based on the longest open reading frame. In 22 of the 26 cases, the new TSS site was in the 3^' ^gene and preserved its reading frame. Altogether, 155 TIC events (46%) preserved the frame of the 3' gene, which is somewhat higher than the chance expectation of 33% and the finding of 36% reported in a previous study [6].

In the 54% of cases where a frameshift occurred in the 3' gene, we determined whether the CDS ended within the last exon, because transcripts with a premature termination codon (PTC) should be degraded by the cellular nonsense-mediated decay (NMD) mechanism [34,35]. We found a PTC in 167 (91%) of the 184 TIC events with a frameshift in the 3' gene. Overall, our analysis indicates that half of TIC events should be degraded by NMD, and half should generate a protein product.

We extended our analysis to predict the domains encoded by the TIC protein products, and to compare them with the original domains of the 5' and 3' genes. We found that conservation of domains depended on whether the reading frame was preserved (Figure 2F). For TIC events with a full CDS from the original TSS of the 5' gene to the original stop codon of the 3' gene, domains should be lost only when the breakpoint occurs before a 5' domain or after a 3^' ^domain. Among the 5' genes that originally had an identifiable domain, all of the domains were preserved in 70% of TIC events and at least one was preserved in an additional 10%. For the 3' genes, these values were 92% and 4%, respectively, indicating that 3^' ^domains are highly likely to be preserved in these TICs. Among the 86 TIC events with identifiable domains in both the 5' and 3' genes, all domains of both genes were preserved by the TIC protein in two-thirds (58) of cases.

For TIC proteins that had a 3' frameshift or a new TSS, but still had their terminating codon in the last exon, domains were more likely to be lost. All 5' domains were preserved in only 45% of cases, and all 3' domains in only 44% of cases. TIC proteins with a premature termination codon (PTC) have a frameshift in the 3' gene, and therefore preserve no 3' domains. Theoretically, the predicted effect on 5' domains shows that they should largely stay intact, with 78% of these cases preserving all 5' domains. However, since proteins with a PTC are subject to degradation by NMD, they should not produce a protein product.

### Expression of TICs and their constituent genes

One advantage of RNA-Seq data is that it can provide expression levels in addition to splicing information. Although our targeted alignments are not useful for determining expression, alignment of reads to either known transcripts or the genome can provide expression levels of genes or even exons. Our spliced alignment method, described later, provided genomic alignments for comparing the expression levels of 5' and 3' genes. We used these alignments to compare the expression of genes involved in observed TIC events with those not involved. To remove possible confounding factors, we required that the genes have an exon with a potential TIC exon within 200,000 bp and that they be expressed in the given sample, as determined by having an observed intragenic splice in that sample. The results (Figure 2G and 2H) indicate that 5' and 3' genes with higher expression levels are more likely to give rise to observable TIC events.

**Figure 3:**
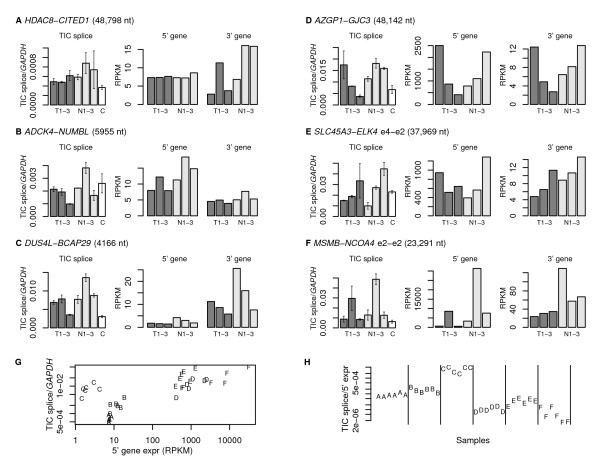
**Expression of TICs and their component genes**. (A-F) Each panel contains expression data for a TIC and its component genes, and is labeled with the splice distance. The leftmost plot in each panel shows the expression of the TIC splice using qRT-PCR measurements relative to *GAPDH *in prostate tumor samples T1-T3, matched normal prostate samples N1-N3, and a commercial sample of normal prostate (C). Error bars indicate the standard error over 2 replicate measurements. The rightmost plots in each panel show expression of the 5^' ^and 3^' ^genes for the T1-T3 and N1-N3 samples, as measured by RNA-Seq in reads per kilobase per million total reads (RPKM). TICs are presented from panel A to panel F in order of increasing TIC splice expression. For panels A-C, expression of the 5^' ^and 3^' ^genes are plotted on the same scale. For panels D-F, because expression of the 3^' ^gene is extremely low relative to that of the 5^' ^gene, expression of each 3^' ^gene is plotted on its own scale. Panel F for *MSMB*-*NCOA4 *has the greatest variance of expression values across samples and shows that TIC splice expression correlates with 5^' ^gene expression, but not 3^' ^gene expression. (G) Relationship between TIC and 5^' ^gene expression, shown as a scatterplot. (H) TIC splicing efficiency, computed as TIC splice expression divided by the 5' gene expression, for each sample. In panels G and H, plot symbols A-F correspond to the TICs labeled in panels A-F.

However, RNA-Seq is limited in its ability to measure expression levels of rare splicing events, such as TICs. To obtain expression levels of TIC splice events and to confirm our computational predictions, we performed experimental qRT-PCR assays of six TIC events from among the 37 that had multiple supporting reads including one from a prostate tumor sample. Regardless of which samples had an observed TIC splice, we evaluated these TICs across our set of six prostate tumor and normal samples, as well as in a commercial sample of pooled normal prostate RNA. In all cases, the TIC events were detected in all samples (Figure 3A-F, left sets of barplots), showing a ubiquitous expression pattern across prostate tissues. However, we did see some variability in expression across our samples, especially for *MSMB-NCOA4*, which was highest in the T2 and N2 samples; *SLC45A3-ELK4*, which was highest in T3 and N3; and *AZGP1-GJC3*, which were low in T2 and T3.

We also saw differences in the overall expression levels across different TIC events, and the TIC events in Figure 3A-F are arranged in increasing order of measured TIC splice expression. The two fusions with the highest overall expression levels relative to *GAPDH *were *MSMB-NCOA4 *and *SLC45A3-ELK4*, each showing expression levels of up to 0.05 times the level of *GAPDH*. The expression levels of the remaining fusions tested were much lower, with *ADCK4-NUMBL *having only 0.004, and *HDAC8-CITED1 *only 0.0009 the level of *GAPDH*.

We used our RNA-Seq data to determine the expression of the 5' and 3' genes in each sample (Figure 3A-F, right sets of barplots). In two of the TIC events (Figure 3A-B), the 5' and 3' gene expression levels are comparable, and in one TIC event, *DUS4L*-*BCAP29 *(Figure 3C), the 3' gene expression is higher than that of the 5' gene expression. However, in the remaining three TIC events (Figure 3D-F), expression of the 5' gene is several orders of magnitude higher than that of the 3' gene. When we compare the expression pattern of the TIC splice with that of its component genes, in most cases, we found a general pattern of correlation between expression of the TIC event and that of the 5' gene, although there was also some correlation with 3' gene expression. These results are generally consistent with other cases studied in the literature, which have also shown correlations between TIC expression and that of the 5' and 3' genes [10]. However, in our data, the TIC event *MSMB*-*NCOA4*, which had the greatest variance in expression across samples showed consistency of TIC expression with that of the 5' but not the 3' gene (Figure 3E).

We can compute the relationship between the TIC splice expression with that of the 5' gene, either as a scatterplot (Figure 3G) or as a ratio (Figure 3H). This relationship reflects the efficiency of TIC splicing relative to the amount of 5' transcript available. Since the data are plotted on a logarithmic scale, they suggest that TIC splicing efficiency varies among different events by several orders of magnitude, with the *DUS4L*-*BCAP29 *event having an efficiency 3000 times as high as that of *MSMB*-*NCOA4*. TIC splicing efficiency does not appear to be related to splicing distance, since we see much different levels for the two TIC events with the shortest splicing distances of 4166 and 5955 nt.

### Tissue specificity of TICs

**Figure 4:**
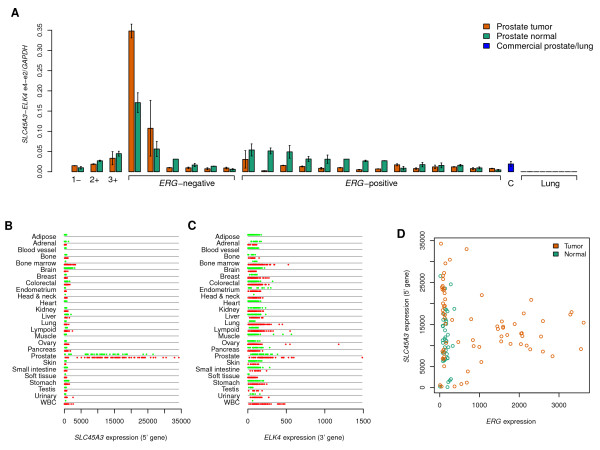
**Expression patterns of the SLC45A3-ELK4 e4-e2 TIC and related genes**. (A) qRT-PCR levels of *SLC45A3*-*ELK4 *e4-e2 TIC in the sequenced prostate tumor and normal sample pairs T1/N1, T2/N2, and T3/N3 pairs (labeled as 1-3, and marked with "-" for ERG-negative and "+" for *ERG*-positive status), plus panels of 6 *ERG*-negative and 14 *ERG*-positive prostate tumor and normal matched samples, a commercial sample of prostate normal RNA, and 9 lung cancer cell lines. (B) Microarray-based expression profile of *SLC45A3 *(Affymetrix probe 228696-at on GeneChip HG-U133B) across human tissues, showing prostate specificity. Samples are organized by tissue, with normal samples above (green) and cancer samples below (red). (C) Microarray-based expression profile of *ELK4 *(Affymetrix probe 206919-at on GeneChip HG-U133A). (D) Relationship of *SLC45A3 *and *ERG *(Affymetrix probe set 241926-s-at on GeneChip HG-U133B) expression levels in prostate tumor and normal samples, showing that highest expression of *SLC45A3 *is restricted to samples with low expression of *ERG*.

Previous reports of the *SLC45A3*-*ELK4 *e4-e2 TIC have found it to be specific to prostate cancer samples, particularly those that lack *ERG *expression [11]. We performed a detailed study of this TIC by qRT-PCR in an additional panel of 20 matched prostate tumor and normal samples (Figure 4A). Our assay detected at least some level of TIC expression in all prostate samples but none in any of 9 lung cancer cell lines, revealing that expression is indeed tissue-specific. We also saw variability of TIC expression with extremely high levels in 2 of 7 *ERG-*negative tumors, at 0.35 and 0.11 times the expression of *GAPDH*, and to a lesser degree, in their matched normal samples. In the remaining 5 *ERG-*negative tumors, and in all 16 *ERG-*positive tumors, expression of the TIC was detectable, but at lower levels, and with only three minor exceptions, expression of the TIC was lower in cancer than in the matched normal sample. Our data support earlier findings that low expression of *ERG *appears to be a prerequisite, but not a sufficient condition, for high cancer levels of the e4-e2 TIC event.

To explain the prostate-specific expression pattern of the *SLC45A3-ELK4 *TIC, we examined expression data from 2823 human normal and 1437 tumor samples measured on the Affymetrix HG-U133 GeneChip, taken from the GeneLogic (Gaithersburg, MD) database. We found that the 5' gene *SLC45A3 *is expressed specifically in prostate tumor and normal samples (Figure 4B), whereas the 3' gene *ELK4 *is expressed broadly across multiple tissues (Figure 4C). Therefore, the prostate-specific expression pattern of this TIC appears to be consistent with expression of the 5' gene.

We further compared the expression of *SLC45A3 *with that of *ERG *in prostate samples (Figure 4D). The highest levels of expression of the 5' gene are found in the *ERG*-negative prostate tumor samples, although not in all of them. Hence, we found further similarity of the expression pattern of the TIC event and that of the 5' gene relative to *ERG *expression, where low levels of *ERG *expression appear to be a prerequisite, but not a sufficient condition, for the highest levels of the 5' gene expression.

We also used the GeneLogic database to find other examples of TIC events with prostate-specific expression of the 5' gene. We found such patterns for *MSMB-NCOA4, AZGP1-GJC3, ENTPD5-FAM161B, TMC5-CP110, TPD52-MRPS28, IVD-BAHD1*, and *KLK11-KLK7*. Among these gene pairs, the read evidence was strongest for *MSMB-NCOA4 *(8 reads over two isoforms) and *AZGP1-GJC3 *(41 reads), with only 1 or reads for each of the other gene pairs.

**Figure 5:**
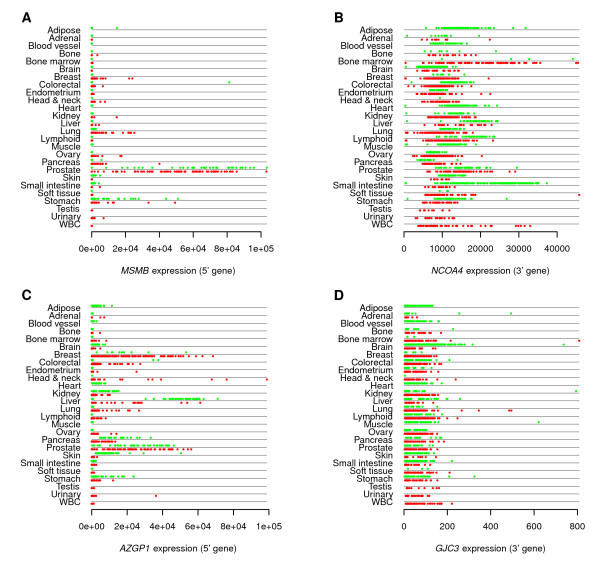
**Expression profiles for **5' **and **3' **genes in prostate-specific TICs**. Expression profiles for (A) *MSMB*, (B) *NCOA4*, (C) *AZGP1*, and (D) *GJC3*. Panels A and C represent the 5' genes of TIC events, while B and D represent 3' genes. Affymetrix probe sets used are 207430js-at, 210774-s-at, 209309-at, and 215060-at, respectively. Data are taken from the GeneLogic (Gaithersburg, MD) database. Expression levels are indicated by position along the *x *axis. Samples are grouped by tissue of origin. Samples in red represent cancer samples, and those in green represent normal samples.

*MSMB *(Figure 5A) shows high expression in normal stomach and lung cancer samples, in addition to prostate samples, and appears to show higher expression in prostate normal samples than in prostate cancer samples. The expression of *AZGP1 *(Figure 5C) is high in breast, head and neck, and liver samples in addition to prostate. For both of these 5' genes, their corresponding 3' genes (Figure 5B and 5D) do not show evidence of prostate specificity.

The prostate specificity of our supporting reads was consistent with the microarray-based prostate specificity of the 5' genes. For example, the *MSMB-NCOA4 *event was supported by 3 prostate tumor reads and 2 normal reads for the e3-e2 isoform, and by 1 tumor and 2 normal reads for the e2-e2 isoform, and was not observed in the HBR or UHR samples. Likewise, the *AZGP1-GJC3 *profile was supported by 7 reads across all three prostate tumor samples, and by 33 reads across all three normal prostate samples; it was also supported by one read from the HBR sample. The TIC events with prostate-specific 5' genes each had TIC support from one or two prostate samples, without any reads from HBR or UHR.

**Figure 6:**
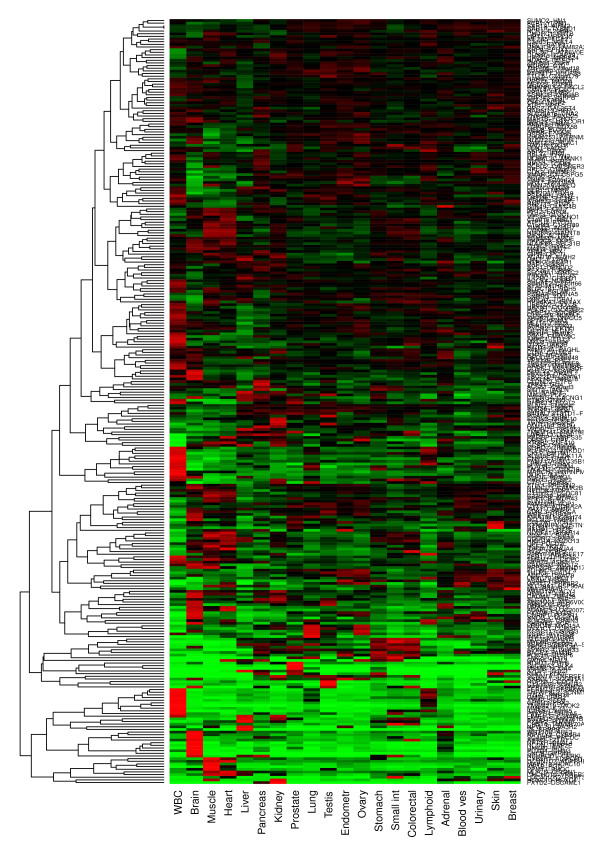
**Tissue specificity of **5' **genes in observed TICs**. Heatmap of gene expression across a panel of normal tissues for the 5' genes corresponding to all observed TICs. Data are taken from the GeneLogic (Gaithersburg, MD) database. Each bar in the heatmap represents the mean expression of the 5' gene in the given tissue. Expression is scaled within each gene to have uniform standard deviation over all genes, and then plotted using its logarithmic value, further transformed by the normal distribution function to achieve a bounded range of colors. Gene expression level is indicated by color, with red indicating increased expression, and green indicating decreased expression.

To survey the extent of tissue specificity in all of our observed TIC events, we constructed a heatmap to show the mean expression of the 5' gene across a panel of normal tissues (Figure 6). This heatmap shows that 5' genes have a varying degrees of tissue specificity. Although it is difficult to define tissue specificity precisely, approximately half of the genes, in the bottom part of the heatmap, are strongly specific to one organ, with many expressed specifically in either leukocytes or in the brain. An additional one-fourth show weaker levels of tissue specificity, and one-fourth have relatively uniform expression over all tissues. The implication of these findings for the tissue specificity of TIC expression depends on the dependence of TIC expression on that of the 5' gene.

### Spliced alignment method

We also explored an alternative, spliced alignment strategy for finding TICs by identifying splicing events within individual reads using our GSNAP program [27]. Our program provides options for finding splicing based on a probabilistic splice site model or a user-provided database of known splice sites. We used both sources of evidence in our alignments to the human genome, with the same set of splice sites based on RefSeq transcripts as we used for the targeted alignment method. Local splicing involving known splice sites within 200,000 bp should therefore yield a subset of the results found in the targeted alignment approach. This method should be less sensitive than targeted alignment, because the version of GSNAP we used requires 14 nt on each side of an exon-exon junction to identify a spliced alignment, rather than the 8 nt we used for targeted alignment. (More recent versions of GSNAP can detect short overhangs when a database of known splice sites is provided.)

For the resulting spliced alignments, we applied the same clustering and filtering steps as for the targeted alignment approach, and found over two-thirds (231) of the 339 TIC events that were found by targeted alignment. These events were based upon 427 TIC alignments, which is half of the number found with targeted alignment. However, the sensitivity rate depended heavily on read length. For samples sequenced at 33-nt, spliced alignment yielded only 24 TIC alignments, which is only 18% of those found with targeted alignment. For samples sequenced at 50-nt or more, spliced alignment yielded 59% of the TIC alignments found by targeted alignment.

### Transcription-induced chimeras with intervening exons

Although spliced alignment is less sensitive than targeted alignment, it does have the ability to find novel splicing at locations not included in a database of known splice sites. We observed many novel splice sites occurring within genes, corresponding to cryptic splice sites or novel exons. However, we were especially interested in novel exons in the context of TIC events. A previous study [5] found several cases where EST clusters revealed a novel exon between two genes in a TIC, accounting for 12% of the gene pairs in their study. Such transcription-induced chimeras with intervening exons, which we call TICIEs, are more challenging to find using RNA-Seq 33- to 50-nt short reads, because they are generally not long enough to span the two introns surrounding most exons. However, we can detect parts of a TICIE by finding novel splicing events on both ends of an apparent intervening exon.

To identify such events, we looked for spliced alignments of 200,000 bp or less involving one known site from the database and one novel site based on a probabilistic splice model as implemented in GSNAP. For each sample, we recorded pairs where the novel splice sites were within 300 bp of each other, which was our threshold for exon length. For the T1, T2, T3, and N3 samples with 33-nt reads, we found 22, 39, 70, and 40 novel exons, respectively. For the N1, N2, HBR, and UHR samples with longer reads, we found 1315, 753, 1427, and 1502 novel exons, respectively. Novel splicing is reported by GSNAP more frequently with longer read lengths, because the program uses a sliding scale of alignment length and probabilistic model score to help find true positive novel splicing events.

The vast majority of these novel exons represent intragenic cases where both known splice sites belonged to the same gene. To find TICIE events, we extracted novel exons where the known splice sites were on different genes and further applied our homology filtering step described previously to eliminate probable false positive events. We further considered only cases where the known genes were coding and well annotated, as indicated by a RefSeq accession prefix of "NM_".

We found 30 TIC events having an intervening exon, with 22 (73%) having their intervening exon occurring between the *(n - *1) exon and +2 exon (Additional file 3). Therefore, the *(n - *1) to +2 exon pattern was stronger in TICIE events than in TICs, where only 51% had this pattern. In half (14) of the cases, the intervening exon was located in the intergenic region, distinct from exons of the original genes; in 7 cases, it overlapped the last exon of the 5' gene; and in 8 cases, it overlapped the first exon of the 3' gene. In the remaining case, *EIF3K-ACTN4 *e4/8-e2/21, the intervening exon was a novel exon between e4 and e5 of the 5' gene.

These TICIE events involved 26 distinct gene pairs, because four gene pairs had a second isoform. Three of these isoforms involved an alternate splice site in the intervening exon, (e.g., Figure 1B), while the remaining isoform involved an alternate splice site in the upstream gene (Figure 1C). For 6 of the gene pairs, our previous targeted alignment analysis had also found a direct TIC event without an intervening exon between the 5' and 3' gene pairs. One example, *VAMP8-VAMP5 *(Figure 1B), shows that the direct TIC and multiple TICIE isoforms can be found in the same sample N1.

We measured the coding potential of the TICIE events both with and without the intervening exon, and compared each with the original genes. Without the intervening exon, 10 events would give a full CDS, 14 started from the original TSS but had a frameshift of the 3' gene, and 6 had a new TSS. With the intervening exon, these counts changed to 5, 19, and 6, respectively. Therefore, intervening exons generally had a detrimental effect on the coding potential, relative to the original frames. The lack of preference for coding potential was also supported by the fact that intervening exons had lengths that were a multiple of 3 in 12 cases, approximately the same as the 10 that would be expected by random chance. The 18 intervening exons with lengths that were not multiples of 3 caused the 3' gene to go out of frame in 4 cases; go into frame in 2 cases; change one premature stop codon to a different premature stop codon in 8 cases; and had no effect in 4 cases because the new TSS occurred after the intervening exon. The intervening exons caused loss of domains in 6 cases relative to the TIC if it had lacked the intervening exon, and a gain of domains in one case by restoring the frame of the 3' gene. We checked to see if any intervening exons introduced a new domain into the protein, but did not find any such cases.

We found that EST-genomic alignments supported three-fourths of our TICIE splicing events: 3 of the 30 events had EST support for their upstream-to-intervening splices only; 11 had EST support for their intervening-to-downstream splices only; and 9 had EST support for both splices. Among the 9 TICIE events with EST support for both splices, 4 had at least one EST that spanned both the upstream-to-intervening and intervening-to-downstream splices (including the two events in Figure 1C).

We compared our TICIE gene pairs with those found by Akiva and colleagues in their study, and found five in common, although in three cases, the previous study found TIC events for the gene pair rather than our TICIE events. In one case, *ZNF763-CHST7*, our intergenic exon was identical to that found previously (although the upstream splice site was different), and in the other case, *VAMP8-VAMP5*, we found two intergenic exons that were different from that found previously, with all three exons sharing the same 3' end and having different 5' ends (Figure 1B).

### Distant fusions

Another advantage of the spliced alignment approach is its ability to detect long-range intrachromosomal fusions and interchromosomal events. Although the identification of translocations in RNA-Seq data is not novel, our single-end short reads are not as ideal for this purpose as paired-end or longer reads of 100*-*200 nt [11]. Nevertheless, our results based on GSNAP on short reads are noteworthy for comparison with previous studies, especially those analyzing the same HBR and UHR samples [17]. In addition, our analysis of distant fusions provides a contrast with our analysis of TIC events, even though both are supported by the spliced alignment approach.

Our genomic alignments gave a total of 29 long-range intrachromosomal fusion candidates of greater than 200,000 bp; 36 scrambled candidates in which the acceptor splice site was upstream of the donor splice site; 59 inversion candidates with the splice sites on opposite strands of the same chromosome; and 223 interchromosomal candidates, or translocations (Additional files 4 and 5).

Almost all of these fusions were identified through the 50-nt reads from N1, N2, HBR, and UHR. Filtering steps are also necessary to eliminate false positives from among these candidates, due to various causes, including library artifacts. Since most of our candidates were supported by a single read, one simple filtering criterion was to consider only fusions supported by multiple reads, of which we found 19 (Table 2).

**Table 2:** Distant gene fusions with multiple read support

Samples	Donor Gene (RefSeq)	Exon	5' chr	3' chr	Acceptor Gene (RefSeq)	Exon	Distance	Notes
Long distance								
HBR(2)	IQCJ (NM_001042705)	4/5	+3	+3	SCHIP1 (NM_014575)	2/8	501759	5
N3(1), T2(5), T3(15)	TMPRSS2 (NM_005656)	1/14	-21	-21	ERG (NM_004449)	4/11	3062463	7
Scrambled exons								
UHR(2)	RPS6KB1 (NM_003161)	4/15	+17	+17	TMEM49 (NM_030938)	12/12	74936	3,6
UHR(2)	GCN1L1 (NM_006836)	2/58	-12	-12	MSI1 (NM_002442)	12/15	157217	
Inversions								
UHR(7)	GAS6 (NM_000820)	12/15	+13	-13	RASA3 (NM_007368)	23/24	185397	6
UHR(2)	TGOLN2 (NM_006464)	3/4	-2	+2	USP39 (NM_006590)	11/13	320033	
UHR(4)	ARFGEF2 (NM_006420)	1/39	+20	-20	SULF2 (NM_018837)	3/21	1172861	2,4,6
UHR(2)	LITAF (NM_001136472)	1/4	-16	+16	DECR2 (NM_020664)	2/9	11193286	
N1(2)	REV1 (NM_016316)	3/23	-2	+2	CPSF3 (NM_016207)	10/18	89944295	
Translocations								
UHR(4)	BCAS4 (NM_017843)	1/6	+20	+17	BCAS3 (NM_017679)	23/24		3,4,6
UHR(3)	BCR (NM_004327)	14/23	+22	+9	ABL1 (NM_005157)	2/11		1,6
N1(2)	CAMTA1 (NM_015215)	3/23	+1	-12	SPPL3 (NM_139015)	3/11		
UHR(2)	DYNC1H1 (NM_001376)	24/78	+14	+12	EIF4B (NM_001417)	8/15		
T3(2)	MBTPS1 (NM_003791)	22/23	-16	+15	SERF2 (NM_001018108)	3/3		
N2(2)	OGT (NM_181672)	6/22	+X	-5	RBM22 (NM_018047)	4/11		
N1(2)	ROR2 (NM_004560)	1/9	-9	-17	USP36 (NM_025090)	2/20		
T3(2)	SEC31A (NM_014933)	1/27	-4	-6	C6orf62 (NM_030939)	2/5		7
UHR(2)	TIMM9 (NM_012460)	3/6	-14	-8	PRKDC (NM_006904)	26/86		
N1(2)	ZDHHC8 (NM_013373)	4/11	+22	+19	UBL5 (NM_024292)	3/5		

Each sample shows the number of supporting reads for the given fusion in parentheses. Exon values refer to the exon number and total number of exons in the given RefSeq transcript. Notes: (1) Known fusion in chronic myelogenous leukemia. (2) Observed in [53]. (3) Observed in [54]. (4) Observed in [55]. (5) Observed in [38]. (6) Observed in [17]. (7) Confirmed by PCR in this study.

In contrast with TIC events, which often had read support from multiple samples, distant gene fusions were each found in only a single sample, with the exception of *TMPRSS2-ERG*, which was found in T2, T3, and N3 (although the N3 had only a single read which may be due to contamination with adjacent tumor tissue). Also, whereas we found many cases of multiple isoforms for TIC events, such isoforms were rare for distant fusions, with the exception of the *RPS6KB1-TMEM49 *scramble, where we found two isoforms, e2-e12 and e4-e12, in the UHR sample. We cannot determine from the transcriptional sequence data if these isoforms are present at the genomic DNA level, or are due to alternative splicing. However, numerous isoforms have been found for the *TMPRSS2-ERG *fusion junction, including multiple isoforms in the same sample, suggesting that they are due to alternative splicing occurring after a single genomic event [36].

We also found that spliced alignment using even 50-nt reads can be challenging, as demonstrated by our alignment of the *BCAS4-BCAS3 *fusion. We found this fusion only because GSNAP provides SNP-tolerant alignment, since the minor T allele rather than the reference G allele occurs at SNP rs2272962 in *BCAS4 *13 nt upstream of the exon-exon junction. Without our SNP-tolerance feature, this SNP would preclude a consecutive 14-nt stretch of upstream matches needed by GSNAP for genomic localization. Alternatively, we could also have avoided this difficulty with longer read lengths that would have given sufficient sequence specificity to tolerate a SNP near the fusion junction.

As shown in Table 2, many of our distant gene fusions have confirmatory evidence from the literature, especially in the MCF7 breast cancer cell line. The presence of cell line fusions in UHR can be explained by its derivation from 10 cancer cell lines, originating from breast adenocarcinoma, cervical adenocarcinoma, glioblastoma multiforme, melanoma, hepatoblastoma, embryonal carcinoma, liposarcoma, Hodgkin's lymphoma, plasmacytoma and T-cell lymphoblastic leukemia [37]. One long-distance fusion, *IQCJ-SCHIP1*, found in HBR and spanning 501,759 bp, has been found previously to be highly expressed in the brain [38]. There are no other RefSeq transcripts between these two genes and the fusion pattern fits the characteristic (*n *- 1) to +2 pattern, so this fusion may possibly represent an unusually long TIC. Our method identified five of the seven most prevalent fusions in UHR as reported by a method using paired-end reads [17]. In addition, we also observed the *NUP214-XKR3 *fusion reported in that paper, but had only a single read supporting that fusion.

In contrast, the only distant gene fusions with evidence from 33-nt reads were two long-range interchromosomal fusions *(C16orf58-NUPR1 *and *TMPRSS2-ERG *e1-e4), three apparent inversions, and 14 apparent translocations. Among these putative fusions, the only ones with multiple supporting reads were *TMPRSS2-ERG *(with 5 reads from T2, 15 from T3, and 1 from N3) and the translocations *MBPTS1-SERF2 *and *SEC31A-C6orf62 *(each with 2 reads from T3).

**Figure 7:**
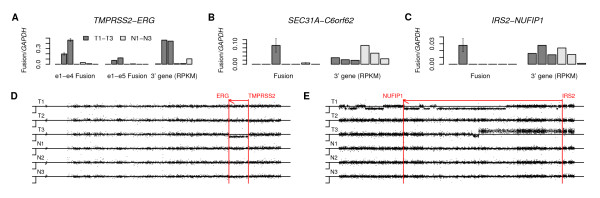
**Distant fusions**. (A) Expression level of *TMPRSS2-ERG *e1-e4 and e1-e5 fusion splices in prostate tumor and normal samples measured by qRT-PCR, compared with *ERG *expression as measured by RNA-Seq. qRT-PCR measurements are shown for prostate tumor samples T1-T3, matched normal prostate samples N1-N3, and a commercial sample of normal prostate. RNA-Seq measurements are shown fir T1-T3 and N1-N3. (B) Comparison of *SEC31A*-*C6orf62 *expression level with downstream *C6orf62 *expression. Fusion is observed only in the T3 sample. (C) Comparison of *IRS2*-*NUFIP1 *expression level with downstream *NUFIP1 *expression. Fusion is observed only in the T2 sample. (D) CGH microarray data for chromosome 21, containing the *TMPRSS2*-*ERG *fusion. A corresponding genomic deletion is observed in the T3 sample, but not in T2, indicating that the gene fusion in T2 is due to translocation. (E) CGH microarray data for chromosome 13, containing the *IRS2*-*NUFIP1 *fusion. No corresponding genomic deletions are observed.

The *TMPRSS2-ERG *fusion is known to be prevalent in prostate cancer, with the predominant isoform being e1-e4 [31,39], which we found in our reads. The second most common isoform is e1-e5 [36], which we did not find in our reads. However, we performed quantification of both isoforms in our samples by qRT-PCR, and found that both isoforms were present in T2 and T3 and at low levels in N2 and N3 (Figure 7A), with the e1-e5 isoform being present at one-tenth the level of the e1-e4 isoform. Since the *TMPRSS2-ERG *fusion is known have a genomic origin, due either to a translocation or to a 3-million-bp genomic deletion between the genes [40], the presence of both isoforms in the same sample can be explained either by tumor heterogeneity or by alternative splicing. We performed comparative genomic hybridization (CGH) microarray analyses of our samples, and found the deletion to be visibly evident in T3 but not in T2 (Figure 7D), indicating that T2 is of the translocation type.

We further tested for the presence of the other candidate long-range interchromosomal fusion *C16orf58-NUPR1 *in our prostate samples but failed to find it by qRT-PCR, suggesting that spliced alignment of 33-nt reads can give false positives in distant fusions. On the other hand, we also tested for the translocation *SEC31A-C6orf62 *e1-e2, and confirmed its presence in the T3 sample, but not in other samples (Figure 7B).

Although the version of GSNAP we used requires at least 14-nt on each side of the exon-exon junction to to report a candidate gene fusion, we relaxed this criterion to see if we could identify other distant fusion events, and obtained the candidate long-range intrachromosomal gene fusions *KRT24-NCOR1, LIN37-GPSN2*, and *IRS2-NUFIP1*. We tested each of these candidates by qRT-PCR, but found confirmation only for *IRS2-NUFIP1 *e1-e8 in T2 (Figure 7C). This fusion spans 64 million bp on chromosome 13. However, our CGH microarray data showed no evidence of a genomic deletion (Figure 7E), suggesting translocation as the probable mechanism.

To evaluate the generalizability of the two novel distant gene fusions that we verified experimentally, we tested for the presence of both *SEC31A-C6orf62 *and *IRS2-NUFIP1 *by qRT-PCR in an additional 51 primary prostate tumor samples, but were unable to detect these fusions in any of these other samples, indicating that they are private fusion events. In addition, when we examined the expression of the downstream genes using our RNA-Seq data, we found no evidence that these fusion events increase the expression of the 3' gene (Figure 7B and 7C). This is in contrast with *TMPRSS2-ERG*, where presence of the fusion greatly increases expression of the downstream gene (Figure 7A).

Functional analysis of *IRS2-NUFIP1 *shows that it has an in-frame coding region starting from the original TSS of *IRS2 *and maintaining the frame of *NUFIP1*. It retains the IRS and PH domains of *IRS2*, but because the breakpoint occurs after the NUFIP1 domain of *NUFIP1*, it loses that domain and presumably functionality of the 3^' ^gene. For the *SEC31A-C6orf62 *fusion, the breakpoint occurs before the TSS of *SEC31A*. The longest open reading frame of the gene fusion gives a peptide that consists of the 171 amino acids on the C-terminal of the original 229-aa protein for *C6orf62*. No domains in the Pfam database were found for either *SEC31A *or *C6orf62*.

## Discussion

Our ability to identify TIC events with high sensitivity using short reads highlights the utility of a targeted alignment approach in dealing with RNA-Seq data. Previous analyses of transcriptional data have reported relatively few TIC events, despite their use of longer or paired-end reads. In contrast, the 339 distinct TIC events found in our study greatly expands the known universe for this phenomenon. Combined with the EST-based surveys, these events contribute towards a total of at least 567 gene pairs with observed TIC events so far. These gene pairs comprise twice as many genes, already within the previous estimates that 4-6% of genes in the genome are involved in TIC formation [6]. Given the low degree of overlap among the two EST-based studies and our RNA-Seq study, we would expect that future RNA-Seq studies should contribute even more new TIC events, suggesting that 4-6% involvement is an underestimate. Rather, our study is in line with the RACE-based finding that perhaps one-third of genes have a TIC event in some tissue [14].

Our study has identified a wider range of distinct TIC events than have been found in other RNA-Seq studies to date, even those that have focused on finding gene fusion events. We believe that there are two major reasons for this disparity. First, previous RNA-Seq studies have looked at the more general problem of finding gene fusions, rather than having a specific procedure for finding TICs. The general approach to identifying gene fusions involves an initial step to identify candidate fusion gene pairs based on discordant mappings between the ends of paired-end reads, and then a second step to align reads to exon-exon junctions between the candidate gene pairs. However, the initial step may miss gene pair candidates since it requires sufficient evidence from paired-end reads for their consideration. In contrast, our TIC-specific analysis begins with the entire set of possible sufficiently close and adjacent gene pairs in the genome as candidates, which provides greater sensitivity for finding TICs. In addition, filtering procedures that are designed to limit false positives for the general class of gene fusions may be more stringent than those needed to identify TICs specifically.

A second reason for the disparity is that TIC events appear to occur at relatively low levels across the genome. Using our criterion of requiring an 11-nt overhang over the exon-exon junction, we found that TIC events represented only 1 per 40,000-120,000 reads, and these were spread over the hundreds of different gene pairs having a TIC event. Therefore, bioinformatics methods that use frequency as a criterion may miss such TIC events. For example, the FusionSeq algorithm [19] relies upon a metric called SPER that represents the number of supporting reads per million mapped reads. A frequency-based criterion such as this may miss phenomena such as TICs that occur at low levels, and will be biased toward TICs that have high levels of expression. Likewise, the analysis by Maher and colleagues [11] ranked their candidates by the total number of mate pairs or long reads that spanned the fusion junction per million mapped reads.

We believe that a probabilistic approach may ultimately be more useful than a simple frequency-based or counting approach. If a plausible biological mechanism exists for TIC formation, then TIC events should be given a higher prior probability, so that less read evidence is necessary for making an inference about the presence of a TIC event. In addition, in a probabilistic analysis, spliced alignments across TIC gene pairs should provide higher odds ratios than alignments across more distant gene fusions, because the short distance of TIC gene pairs in the genome limits the universe of possible alternative hypotheses. These probabilistic ideas are embodied in our own analysis where, for example, we accepted TIC candidates based on a single read, but required multiple reads before considering a gene fusion candidate. It is possible, though, that our 212 single-read candidates are less likely to be true positives, since only 28% of them had external supporting evidence, whereas 54% of our 127 multiple-read candidates did so. Alternatively, the single-read candidates may represent TICs that are expressed at lower levels and therefore less likely to have been found by EST-based approaches.

Nevertheless, our computational estimate of the false discovery rate for our pipeline is 1.5%, suggesting that 98% of our TIC events represent true biological events. Our experimental validation rate of 6 out of 6 is consistent with a recent study of gene fusions in prostate cancer [18], in which 9 of the 11 predicted readthrough fusions were experimentally validated. In addition, the predominance of short splicing distances and the (*n *- 1) to +2 pattern in our data would not occur if our fusion events were due to library artifact or spurious alignments. It is also unlikely that sequencing errors, which typically give mismatches or indels, could artefactually generate nucleotide sequences from disparate exons.

However, our study also highlights some of the limitations of a targeted alignment strategy, especially its inability to be used generally to find gene fusion events. In our process of constructing exon-exon targets, we found approximately 180,000 unique donor sites and 180,000 unique acceptor sites in the human genome. To represent all exon-exon pairs would require 32 billion exon-exon targets, and for targets each of length 150 nt, we would have a need to index 5 trillion nucleotides, a value that exceeds the limits of GSNAP, for example, by a factor of 1000. What makes targeted alignment feasible for the universe of TICs is the constraint that the splice sites lie within 200,000 bp, which limits the number of exon-exon junctions to 2.5 million, a small fraction of the total set of possibilities. Nevertheless, targeted alignment can still be used as a sensitive method for finding preselected gene fusion events. In fact, an analogous experimental strategy using microarrays has been developed where all combinations of exon-exon junctions between selected pairs of fusion genes are represented by oligonucleotide probes [41]. However, our study also shows that as read lengths become longer, a spliced alignment approach is also effective in identifying most of the TIC events found by the targeted alignment approach.

Another limitation of targeted alignment in this study is that we restricted our analysis to readthrough fusions across adjacent genes on the same genomic strand. Our analysis therefore excludes gene pairs in the same genomic region but on opposite strands, which have been called converging or diverging fusions [11] or *cis*-type fusions [18,19]. Our analysis also excludes fusions between nearby genes that skip across an intervening gene. Nevertheless, a targeted alignment strategy could be readily extended to handle these types of events.

In contrast with these other local fusion types and more distant gene fusions, which generally have a DNA-based mechanism, readthrough fusions have a plausible RNA-based mechanism. In particular, the biological mechanism supported by our study and by previous studies is that TICs are largely a "leakage" or *cis*-based event. Although TICs could potentially be caused by a *trans*-splicing mechanism between two different transcripts, the proximity of the gene pairs makes a *cis *mechanism involving a single transcript more plausible. A *cis*-based mechanism is also supported by our identification of 30 TICIE events, since intergenic exons are unlikely to be incorporated by a *trans-*splicing mechanism between two separate gene transcripts.

Under a leakage mechanism, the first step to generating a TIC involves a failure to terminate transcription of the 5' gene. Such a mechanism is suggested by previous findings [11] that TICs have a broad pattern of expression across multiple samples, in contrast with other types of gene fusions that tend to have a restricted pattern of expression in particular samples. Further support is provided by this study which indicates that TICs are be found more readily when the upstream gene is expressed at higher levels, and that expression levels of TICs correlate with those of the 5' gene, although we often observe some association with 3' gene expression as well.

A tissue-specific pattern of the 5' gene can therefore give rise to tissue-specific expression of TICs. For example, for the TICs *MSMB-NCOA4 *and *AZGP1-GJC3*, our read evidence is restricted to prostate samples, which corresponds to the prostate-specific expression pattern of the 5' gene and not the 3' gene. Furthermore, we can explain the prostate-specific and *ERG*-negative expression pattern of the *SLC45A3-ELK4 *fusion based on expression of the 5' gene *SLC45A3*. Similarly, previous researchers observed the *HHLA1-OC1 *fusion in those teratocarcinoma cell lines where *HHLA1 *was expressed highly [7].

However, we cannot rule out the possibility that other factors may increase or decrease the expression of particular TIC events. In our study, we found widely differing levels of efficiency of TIC expression relative to expression of the 5' gene. Evidence from other researchers suggests that expression of TICs may be increased by cellular stimuli, as has been shown for the *SLC45A3-ELK4 *e1-e2 fusion [12] and for trans-splicing products [32]. It is also possible that genomic deletions or other alterations in intergenic regions may remove or alter signals for transcriptional termination, making TIC events more likely to occur in some samples.

The second step in a leakage mechanism should involve splicing between the two genes on the same transcript. As with typical splicing, such splicing would be opportunistic, tending to occur between the closest pair of splice sites at the shortest possible distance, which we also observe. However, alternate splicing could still occur between genes, and such alternate choices presumably explain our finding of several cases of gene pairs having multiple TIC isoforms. The fact that TICs conserve the +2 splice site more strongly than the *(n - *1) splice site could be explained by the more discriminating signals that surround acceptor sites, including the polypyrimidine tract and the lariat signal.

In contrast with the large number of TICs found in this study, we found relatively few distant gene fusions, and experimentally verified only two novel ones in prostate adenocarcinoma: *IRS2-NUFIP1 *and *SEC31A-C6orf62*. One reason for this is that our data consisted of short 33-bp single-end reads, whereas paired-end data are more sensitive for finding distant gene fusions [17]. Another reason may simply be the relative infrequency of gene fusion events in prostate cancer; a recent RNA-Seq study [18] of 25 prostate cancers (7 ETS-positive and 18 ETS-negative) using paired-end 50-mers yielded only 7 verifiable gene fusions. Interestingly, in that study, the 5 gene fusions that did not involve an ETS-family gene were all found in ETS-positive samples. Likewise, the two gene fusions found in our study were also found in ETS-positive samples, consistent with the hypothesis that *TMPRSS2-ERG *rearrangements may correlate with other rearrangements, possibly induced by a common predisposing mechanism such as binding by androgen receptor to the genome [42].

The functional role, if any, of TICs remains an open question. Our study suggests that half of TICs terminate in their last exon, therefore avoiding degradation by nonsense-mediated decay, and among these proteins, domains should largely stay intact. Our computational analysis is subject to some caveats, however. Alternate polyadenylation sites can result in a last exon different from the one we have predicted, and alternate transcription start sites can mean that the actual coding region is different from the one predicted. Experimental evidence to determine the precise peptides encoded by TICs may require high-throughput proteomic analyses [43]. In addition, we should note that the loss of a domain in a TIC does not necessarily imply decreased gene function, since a loss of negative regulatory region can result in increased function of a gene.

Accordingly, it appears that transcription-induced chimeras, like gene fusions and other splicing anomalies [44], may play a role in cancer. Examination of the TIC events in our study reveals several that involve cancer-associated genes, including the oncogenes *E2F1, MAFG, MRAS, NTRK1*, and *RHOC*. One TIC discovered in our study with particular relevance to prostate cancer is *MSMB-NCOA4*, for which we found two isoforms, e3-e2 and e2-e2, with the latter isoform supported by three ESTs. Both of these isoforms fit the (*n *- 1) to +2 pattern, since the *MSMB *gene has alternate forms with two or three exons. Analysis of the e3-e2 fusion suggests that it may be subject to NMD, but the e2-e2 fusion should maintain the frame of the 3' gene and preserve the 5' and 3' domains. The upstream partner, microseminoprotein beta, codes for a constituent of semen and has been shown in two genome-wide association studies [45,46] to be linked to prostate cancer risk. The downstream partner, also known as *ARA70 *(androgen receptor associated protein 70), is also potentially relevant to prostate cancer, since it is known to enhance the transcriptional activity of androgen receptor in prostate cancer cells [47]. Taken together, the high expression of the upstream gene in prostate tissue, combined with the physiological relevance of the downstream gene, suggests a possible role for this TIC in prostate cancer biology.

However, we should note that many TICs, and indeed many gene fusions, may not have a functional role at all. They may be private or passenger events, much like those found in surveys of point mutations in cancer [48]. The novel distant gene fusions found in this study, *IRS2-NUFIP1 *and *SEC31A-C6orf62*, appear to fall into this category. We found these fusions to present in tumor samples and not in their matched normal samples, confirming that they were somatic in origin, but each fusion occurred only once among 54 prostate tumor samples tested. In addition, among the 7 prostate cancer gene fusions found in the FusionSeq-based study [18], only two had a duplicate occurrence among the 200 additional prostate tumor samples they tested. Nevertheless, even driver gene fusions may be rare, as in the case of *R3HDM2-NFE2*, which was found in only 2 of 76 lung adenocarcinoma samples [49]. Therefore, additional work may be necessary to further characterize the function of candidate fusion events or the TIC events found in our study. Nevertheless, deep transcriptional sequencing remains an important approach for identifying novel phenomena that can serve as candidates for further investigation.

## Conclusions

A targeted alignment approach provides a sensitive method for identifying TICs in short read data, while spliced alignment reveals numerous cases of intervening exons between adjacent genes as well as gene fusions. Both methods applied to deep transcriptional sequencing data demonstrate a large number and diverse range of TIC events within individual tissues. The low degree of overlap among the two EST-based studies and our RNA-Seq study suggest that TIC events are widespread and that previous estimates of 4*-*6% genes involved in TICs are an underestimate. Combined evidence from RNA-Seq-based expression and qRT-PCR measurements support a *cis*-splicing mechanism, in which the tissue and cancer specificity of TIC events are controlled by expression patterns of the upstream gene.

## Methods

### Samples

Samples used in this study are listed in Additional file 6. All of the prostate samples in our study were reviewed by Board-certified pathologists at our institution. Three human primary prostate tumors (T1, T2, and T3) and adjacent matched normal tissue samples (N1, N2, and N3) were obtained from commercial sources with appropriate consent and institutional approval. Pathology review showed that the tumor samples had a tumor content of at least 70%.

The human brain reference (HBR) RNA sample was obtained from Ambion catalog number 6050. The universal human reference (UHR) samples was obtained from Stratagene catalog number 740000.

Normal human prostate control (C) total RNA was obtained from Clontech (Mountain View, CA), catalog number 636550, and used in TaqMan verification.

The *SLC45A3-ELK4 *fusion was evaluated in an additional panel of 20 prostate tumors and their matched normals, as well as 9 lung cancer cell lines: DMS79, H23, H522, H1703, H520, H1838, H1563, H1688, and H1734. The *IRS2-NUFIP1 *and *SEC31A-C6orf62 *fusions were tested in an additional 51 prostate tumors, as shown in Additional file 6.

### Transcriptional sequencing

RNA and DNA were extracted using the Qiagen AllPrep RNA/DNA kit. Libraries used in sequencing were constructed by random priming with polyA. Messenger RNA was isolated from total RNA by poly-dT capture and enrichment. Adapters for sequencing were ligated to the cDNA per manufacturer's instructions (Illumina, Hayward, CA). The processed libraries were sequenced by Illumina on a Genome Analyzer using their single-end protocol.

Read lengths were 33 nt for samples N3, T1, T2, and T3; 50 nt for N2, HBR, and UHR; and 50 and 75 nt for sample N1. Samples T3 and N1 were each sequenced over two different Illumina flow cells. We obtained 226 million single-end reads from 8 sequencing runs for tumors T1, T2, and T3, and matched normals N1, N2, and N3. We obtained 53 million reads for HBR and 60 million reads for UHR.

### Targeted alignment

We aligned transcripts from RefSeq release 31 to the human genome build 36.1 using the best alignment from GMAP to obtain 46,546 alignments. We restricted our analysis to those well-annotated RefSeq transcripts starting with "NM_", or 27,157 alignments. These alignments contained 179,909 unique donor sites and 180,874 unique acceptor sites. Sites were often found in more than one transcript, due to alternative transcripts in RefSeq. We therefore labeled each donor and acceptor site with the set of exons from all alternative transcripts containing that site.

We found that 115,240 donor sites had a potential TIC acceptor site within 200,000 nt on the same genomic strand, where that site had no associated transcripts in common with the donor site. Likewise, we found that 108,298 acceptor sites had a potential TIC donor site. A computer script generated all potential TIC exon-exon junctions by taking 80 nt upstream and 80 nt downstream of all possible TIC donor-acceptor pairs. Pairings of these sites yielded 2,470,383 possible TIC exon-exon junctions, each of length 160 nt. To reduce the occurrence of false positive alignments, we also generated all possible intragenic exon-exon junctions from all pairs of exons within the same transcript, resulting in 1,856,519 potential intragenic exon-exon junctions.

We constructed index files for both the TIC and intragenic exon-exon junctions using the GMAP_SETUP program. We mapped short reads to the artificial exon-exon junctions using GSNAP (version 2010-07-27), allowing up to a score of 5 ("-m 5" flag), where mismatches count as 1 point and indel gap openings count as 1 ("-i 1"). We considered a read to be evidence for an exon-exon junction if it had a unique alignment to a target with zero or one mismatch and no indels and if the alignment extended past the midpoint of the target by a certain overhang amount.

After clustering the alignments by exon-exon junction, we implemented a filtering process to reduce the incidence of false positives due to poor alignments. We accepted only clusters that had 11 or more consistent match positions on both sides of the exon-exon junction and no consistent patterns of mismatches on either side. A consistent match position was one that matched in the genome in at least one of the reads of the multiple sequence alignment.

We also implemented a filtering process to remove false positives due to homologous genes. We aligned a given candidate TIC exon-exon junction against each RefSeq transcript containing one of the exons using GMAP [28]. We constructed the nucleotide fragment corresponding to the longest overhang observed in each side of the exon-exon junction. We rejected the TIC candidate if 90% or more of the splice junction aligned to any component gene transcript. We also aligned the upstream RefSeq genes against the downstream genes for possible evidence of similarity, again using GMAP. If we found any local alignment having 40 matches within a window of 50 nt, we also rejected the TIC candidate.

To assess the false discovery rate (FDR) of our analysis pipeline, we applied a previously developed technique for estimating the FDR for alternative splicing predictions [20]. For each of our artificial exon-exon junctions, we removed 5 bp from both the 5^' ^and 3^' ^exons, at the -10 to -6 and the +6 to +10 positions relative to the junction, and applied the same alignment and filtering steps against these junctions.

All clustering and filtering steps were performed using computer scripts written in Perl.

### Analysis of coding regions and domains

Coding regions and protein sequences for the 5' and 3' genes were obtained from the CDS field in the RefSeq entry for the gene. Chimeras were constructed by concatenating component exons from the 5^' ^and 3^' ^genes. If the original transcriptional start site (TSS) of the 5^' ^gene was included in the chimera, the coding region and protein was extended from that TSS. If the chimeric breakpoint occurred before the TSS, then the coding region and protein were predicted computationally from the longest open reading frame.

Domains were predicted from protein sequences using hmmscan version 3.0b3 in the HMMER package http://hmmer.janelia.org, and the Pfam database of protein domains version 24.0 [50]. A domain was considered present in the original genes if it had an E-value of 1e-6 or lower in any single position in the protein sequence. It was considered present in the chimeric protein if it appeared in the list above the default inclusion threshold, which is based on a per-sequence E-value of 0.01. The set of chimeric domains was then compared with each of the domain sets for the original 5^' ^and 3^' ^proteins. If the original 5^' ^or 3^' ^protein had no domains, that relationship was considered "ND" (no domain). Otherwise, the chimeric set of domains was characterized as being either null (no intersection with the original domains); a proper, non-empty subset; or covering all of the original domains.

### Spliced alignment

We aligned the reads using GSNAP [27] to human genome build 36.1, allowing for three mismatches ("-m 3" flag) and an indel penalty of 1 ("-i 1"), with the SNP-tolerance feature ("-V") enabled for dbSNP version 129 and both novel ("-N 1") and known splice detection ("-s") enabled for known splice sites from RefSeq release 31. Subsequent collection and filtering steps were performed using computer scripts written in Perl.

These alignments were also used for measuring gene expression levels. Expression was measured in reads per kilobase per million total reads (RPKM) by counting the number of reads aligning to exons in a given gene, and then normalizing by the total length of the exons and the total number of reads.

### Validation of TIC and fusion events

TIC and fusion events were confirmed using TaqMan assays. For each event, a probe and two sets of forward and reverse primers were designed and synthesized (IDT, Coralville, IA), and tested in a pilot study using the original prostate sample that generated the short read providing evidence for the TIC or fusion event. Primers that gave good experimental results in the pilot study were selected for further use in subsequent assays and measurements. Additional file 7 lists the primers and probes used in the study, with both sets of primers listed for events where the pilot study failed to confirm the original event.

The *GAPDH *control primer and probe set was obtained from Applied Biosystems. Probes were designed based on the unique exon junctions formed by fusion events. Assays were performed using 25 ng RNA per reaction using the Qiagen QuantiTect Probe RT-PCR kit. The one step qRT-PCR reaction was performed at 50 deg for 30 min. and 95 deg for 15 min., followed by 40 cycles at 94 deg for 15 sec. and 60 deg for 1 min. Data was collected with the ABI PRISM 7000 Sequence Detection System. Target amount relative to *GAPDH *was computed by the comparative CT method [51]. Each qRT-PCR reaction was performed twice to obtain a mean value and standard error.

### CGH microarray protocols

Genomic DNA was labeled and hybridized to Agilent CGH 244K microarrays (Santa Clara, CA) using the manufacturer's recommended protocols. Human male genomic DNA (Promega P/N G1471) was used as reference. Individual log2 ratios of background-subtracted signal intensities were obtained from the Agilent Feature Extraction software version 9.5.

### Data availability

Sequencing and microarray data used in this study have been deposited with the NCBI Gene Expression Omnibus database [52] and are accessible through GEO Series accession number GSE24284, with the CGH microarray data accessible through SubSeries accession number GSE24282 and the sequencing data accessible through SubSeries accession number GSE24283.

## Competing interests

The authors declare that they have no competing interests.

## Authors' contributions

BAP identified prostate samples for study and performed laboratory validation with DB and JS. SN, ZK, KJ, and TDW performed data analyses, with analysis design supervised by TDW. WY performed experimental qRT-PCR validation, and CSR and ZM performed microarray hybridations. SS and TDW designed the study and provided scientific oversight. SN and TDW prepared the manuscript, which was read and approved by all authors.

## Pre-publication history

The pre-publication history for this paper can be accessed here:

http://www.biomedcentral.com/1755-8794/4/11/prepub

## Supplementary Material

Additional file 1 **TIC events found by targeted detection approach**. Click here for file

Additional file 2 **Supporting reads for TIC events**. Click here for file

Additional file 3 **TICs with intervening exons**. Click here for file

Additional file 4 **Distant fusions found by spliced alignment approach**. Click here for file

Additional file 5 **Supporting reads for distant fusions**. Click here for file

Additional file 6 **Sample information and pathology evaluation**. Click here for file

Additional file 7 **Primers and probes used for qRT-PCR measurements**. Click here for file
