# Supplementary material for: Deep RNA sequencing analysis of readthrough gene fusions in human prostate adenocarcinoma and reference samples
Source: BMC Med Genomics. 2011 Jan 24;4:11. doi: 10.1186/1755-8794-4-11 (PMC3041646; doi:10.1186/1755-8794-4-11)
Supplement: Additional file 1 — TIC events found by targeted detection approach. [file 1755-8794-4-11-S1.PDF]

Additional file 1 — TIC events found by targeted detection approach.

Reads									Donor		Acceptor								
T1	T2	T3	N1	N2	N3	H	U	Chr	Gene	Exon	Gene	Exon	Distance	CDS	Ace	ESTs	Akiva	Parra	Maher
0	0	1	1	0	0	0	0	−19	DUS3L (NM_020175)	12/13	PRR22 (NM_001134316)	2/3	943	l	Y	1			
0	0	0	1	0	0	1	0	+6	STK19 (NM_004197)	7/8	C4A, C4B (NM_007293)	2/41	1503	F		0			
1	0	0	1	0	0	2	2	−22	TYMP (NM_001953)	9/10	SCO2 (NM_005138)	2/2	1576	l	Y	2			
0	0	0	0	0	0	0	2	−3	COL7A1 (NM_000094)	117/118	UCN2 (NM_033199)	2/2	1646	l	Y	1	Y		
0	0	0	0	0	0	0	1	+6	CSNK2B (NM_001320)	6/7	LY6G5B (NM_021221)	2/3	1647	p	Y	2			
0	0	0	0	0	0	1	0	+3	IL17RC (NM_153460)	17/19	CRELD1 (NM_001031717)	2/12	1716	p		0			
0	0	0	0	1	0	0	0	−14	TM9SF1 (NM_006405)	5/6	IPO4 (NM_024658)	2/30	1741	p		0			
1	0	0	0	3	0	0	2	−22	TYMP (NM_001953)	8/10	SCO2 (NM_005138)	2/2	1821	l	Y	4			
0	0	0	0	0	0	0	2	+20	LIME1 (NM_017806)	5/6	SLC2A4RG (NM_020062)	2/8	1877	F	Y	3	A	A	
0	0	0	0	0	0	0	1	−16	SLC12A4 (NM_005072)	22/24	LCAT (NM_000229)	2/6	2156	p		0			
0	0	0	0	0	0	2	0	+12	BLOC1S1 (NM_001487)	3/4	RDH5 (NM_002905)	3/5	2465	p	Y	14	Y	Y	
0	0	0	0	0	0	0	1	−14	NEDD8 (NM_006156)	3/4	MDP1 (NM_138476)	3/6	2470	F	Y	1	A		
0	0	1	0	0	0	0	0	+5	HARS2 (NM_012208)	12/13	ZMAT2 (NM_144723)	2/6	2740	F		0	A	A	
0	0	0	0	0	0	1	0	−6	MRPL2 (NM_015950)	6/7	CUL7 (NM_014780)	2/26	2748	p		0			
0	0	0	0	0	0	1	0	+22	AIFM3 (NM_001018060)	19/20	LZTR1 (NM_006767)	2/21	2902	F	Y	1			
0	0	0	0	0	0	1	0	+19	MIA (NM_006533)	3/4	RAB4B (NM_016154)	2/8	2939	p	Y	1	Y	Y	
0	0	0	0	0	0	1	0	−12	CHD4 (NM_001273)	39/40	NOP2 (NM_006170)	2/16	2946	p		0			
0	0	0	1	0	0	0	0	−19	GMIP (NM_016573)	20/21	LPAR2 (NM_004720)	2/3	3186	p		0			
0	0	0	0	0	0	0	1	−6	GPSM3 (NM_022107)	7/8	PBX2 (NM_002586)	3/9	3204	p		0			
0	0	0	1	0	0	0	1	+17	STARD3 (NM_006804)	14/15	TCAP (NM_003673)	2/2	3371	l		0			
0	0	0	0	0	0	0	1	−7	TSC22D4 (NM_030935)	4/5	C7orf61 (NM_001004323)	2/3	3865	F		0			
0	0	0	0	0	0	0	1	−X	IL2RG (NM_000206)	7/8	CXorf65 (NM_001025265)	5/6	3872	p		0			
0	0	0	0	0	0	0	1	−7	GPC2 (NM_152742)	9/10	GAL3ST4 (NM_024637)	2/4	3977	p		1			
0	0	0	0	0	0	0	1	+6	PPT2 (NM_005155)	8/9	EGFL8 (NM_030652)	4/9	4078	p		0			
0	0	1	0	0	1	1	0	+7	DUS4L (NM_181581)	7/8	BCAP29 (NM_001008405)	2/9	4166	F		0			
0	0	0	0	0	0	4	0	−22	MAPK12 (NM_002969)	11/12	HDAC10 (NM_032019)	2/20	4291	p		0			B
0	0	0	0	1	0	1	0	−5	MXD3 (NM_031300)	5/6	RAB24 (NM_130781)	2/9	4460	p	Y	3	Y		
0	0	0	0	0	0	0	3	−20	E2F1 (NM_005225)	6/7	NECAB3 (NM_031231)	2/13	4682	p		0			
0	1	0	0	0	0	0	0	+16	HSF4 (NM_001538)	13/15	NOL3 (NM_003946)	2/4	5060	l		0	A		
0	0	0	0	0	0	0	1	+16	FAM173A (NM_023933)	3/5	HAGHL (NM_207112)	2/7	5470	p		0			
0	0	0	0	0	0	0	3	−16	VKORC1 (NM_024006)	2/3	PRSS53 (NM_001039503)	2/11	5485	F	Y	2			
0	0	0	0	0	0	0	1	+X	MED12 (NM_005120)	44/45	NLGN3 (NM_018977)	2/7	5585	p		0			
1	0	0	1	3	0	2	2	−18	RPL17 (NM_000985)	6/7	C18orf32 (NM_001035005)	2/3	5587	p	Y	10	Y		
0	0	0	1	0	0	0	0	+19	PSENEN (NM_172341)	3/4	LIN37 (NM_019104)	2/9	5652	p	Y	0		Y	
0	0	0	0	1	0	0	1	+9	GALT (NM_000155)	10/11	IL11RA (NM_004512)	2/13	5653	F		0	A	A	
0	0	1	0	0	0	1	0	+3	ABHD14A (NM_015407)	3/5	ACY1 (NM_000666)	2/15	5672	p	Y	3	Y	Y	B
0	0	0	1	0	1	0	0	+17	MLLT6 (NM_005937)	19/20	CISD3 (NM_001136498)	2/4	5783	F		0			
0	0	0	0	0	0	1	0	−3	TUSC2 (NM_007275)	2/3	HYAL2 (NM_003773)	2/4	5821	p	Y	1			
0	0	1	0	0	2	1	0	−19	ADCK4 (NM_024876)	14/15	NUMBL (NM_004756)	2/10	5955	F		0			U
1	0	5	9	7	1	18	14	+1	PMF1 (NM_007221)	4/5	BGLAP (NM_199173)	2/4	6069	p	Y	19		Y	B,U
0	0	1	1	0	0	0	0	+16	TMEM219 (NM_194280)	5/6	TAOK2 (NM_004783)	2/19	6159	p		0			
0	0	0	1	0	0	0	0	+16	FBXL19 (NM_001099784)	10/11	ORAI3 (NM_152288)	2/2	6236	l		0			
0	0	0	0	0	0	1	0	−10	CAMK2G (NM_172169)	20/21	NDST2 (NM_003635)	3/15	6273	p		0			
0	0	0	0	0	0	1	0	−19	COPE (NM_007263)	9/10	LASS1, GDF1 (NM_001492)	2/8	6297	F		0			
0	0	0	1	1	0	0	8	+3	ARPC4 (NM_005718)	5/6	TTLL3 (NM_001025930)	2/13	6607	p	Y	17		A	

Reads									Donor		Acceptor								
T1	T2	T3	N1	N2	N3	H	U	Chr	Gene	Exon	Gene	Exon	Distance	CDS	Ace	ESTs	Akiva	Parra	Maher
0	0	0	0	0	0	1	0	−1	PPM1J (NM_005167)	9/10	RHOC (NM_001042678)	2/5	6653	F	Y	1	Y		
0	0	0	1	0	0	3	0	+9	TMEM141 (NM_032928)	4/5	KIAA1984 (NM_001039374)	2/14	6743	F	Y	1	Y	Y	
0	0	1	0	0	0	0	0	−19	MED26 (NM_004831)	2/3	SLC35E1 (NM_024881)	2/6	6752	p		0			
0	0	0	1	0	0	0	0	+5	UQCRQ (NM_014402)	2/3	LEAP2 (NM_052971)	2/3	6914	p		0	A		
0	0	1	0	1	0	0	3	−1	SLC39A1 (NM_014437)	4/5	CRTC2 (NM_181715)	2/14	7053	F		0	A		
0	0	0	4	0	0	3	4	+11	SIDT2 (NM_001040455)	25/26	TAGLN (NM_003186)	2/5	7086	F		1	Y	Y	
0	0	0	3	0	0	0	0	+16	MMP25 (NM_022468)	9/10	IL32 (NM_001012633)	2/8	7114	p		0			
0	1	0	2	3	0	0	2	+7	DTX2 (NM_020892)	11/12	UPK3B (NM_030570)	2/4	7211	p		1		Y	
0	0	0	0	0	0	0	1	−11	UBXN1 (NM_015853)	7/8	C11orf48 (NM_024099)	3/7	7714	F		0			
0	0	0	0	0	1	0	0	+X	MAGIX (NM_024859)	5/6	PLP2 (NM_002668)	2/5	7776	p		0			
1	0	4	0	0	0	0	1	−1	VPS72 (NM_005997)	4/5	TMOD4 (NM_013353)	10/10	7897	F	Y	1	Y	Y	
0	0	0	0	0	0	0	1	+19	GPI (NM_000175)	14/18	PDCD2L (NM_032346)	2/7	7991	F	Y	1	Y	Y	
0	1	0	1	0	0	0	1	−11	HNRNPUL2 (NM_001079559)	13/14	BSCL2 (NM_001130702)	2/12	8175	p	Y	1			
0	0	0	0	0	0	2	0	−11	TRIM3 (NM_006458)	12/13	HPX (NM_000613)	2/10	8575	p		0			
0	0	0	0	1	0	1	0	+3	ABHD14A (NM_015407)	1/5	ACY1 (NM_000666)	2/15	8842	F	Y	2	A	A	B
0	0	0	0	0	0	1	0	+11	VPS11 (NM_021729)	13/15	HMBS (NM_000190)	2/14	8954	p		0			
0	0	2	0	0	0	0	1	−17	FAM117A (NM_030802)	7/8	SLC35B1 (NM_005827)	2/9	9096	F		0			
0	0	0	0	0	0	0	1	+1	GUK1 (NM_000858)	8/9	GJC2 (NM_020435)	2/2	9283	l		0			
0	0	0	2	1	0	2	1	+19	VMAC (NM_001017921)	1/2	CAPS (NM_004058)	2/5	9303	F		0			
0	0	0	0	0	0	0	1	−2	OBSL1 (NM_015311)	20/21	CHPF (NM_024536)	2/4	9339	p		1			
0	0	1	0	0	0	0	1	−10	NDUFB8 (NM_005004)	4/5	SEC31B (NM_015490)	2/26	9401	p	Y	2			B,U
0	0	0	0	0	0	0	1	+19	PGLS (NM_012088)	4/5	FAM129C (NM_173544)	2/16	9418	F		0			
0	0	0	0	0	0	2	0	−9	MED22 (NM_133640)	4/5	SURF6 (NM_006753)	2/5	9542	p	Y	1	Y		
2	1	0	0	0	1	0	0	−4	FAM175A (NM_139076)	8/9	HELQ (NM_133636)	2/18	9548	p		0			
0	0	0	0	1	0	0	0	−17	MAFG (NM_002359)	1/3	SIRT7 (NM_016538)	2/10	9559	t		0			
0	0	0	0	0	1	0	0	+14	PTGR2 (NM_152444)	9/10	ZNF410 (NM_021188)	2/12	9588	p		0	A	A	
0	0	0	0	1	0	0	0	−17	SCRN2 (NM_138355)	7/8	MRPL10 (NM_145255)	2/5	9599	p		0			
0	0	1	3	0	0	1	0	+1	TNFAIP8L2 (NM_024575)	1/2	SCNM1 (NM_024041)	2/7	9748	t	Y	1	Y		
0	0	0	0	0	0	0	1	−16	ZDHHC1 (NM_013304)	4/11	TPPP3 (NM_015964)	2/4	9839	p		0			
0	0	1	3	0	0	3	1	+12	FAM119B (NM_015433)	2/3	TSFM (NM_005726)	2/6	9981	p	Y	1	Y		
0	0	0	0	0	0	1	0	+6	RIPPLY2 (NM_001009994)	3/4	CYB5R4 (NM_016230)	2/16	10013	p	Y	1			
0	0	0	0	0	0	3	0	+10	SNCG (NM_003087)	3/5	C10orf116 (NM_006829)	2/3	10071	l		0			
0	0	0	0	0	0	0	1	−7	PMS2 (NM_000535)	14/15	RSPH10B (NM_173565)	2/21	10150	p		0			
0	0	0	0	0	0	1	0	−7	TSC22D4 (NM_030935)	4/5	C7orf61 (NM_001004323)	3/3	10566	F		0			
0	0	1	0	0	1	0	0	−19	CADM4 (NM_145296)	7/9	ZNF428 (NM_182498)	2/3	10643	p		0			
0	0	0	0	1	0	0	0	+16	FBXL19 (NM_001099784)	9/11	ORAI3 (NM_152288)	2/2	10648	l		0			
0	0	0	0	0	0	0	1	+22	SLC2A11 (NM_030807)	11/13	MIF (NM_002415)	2/3	10747	p		0		A	
0	0	0	0	0	0	1	0	−9	ZER1 (NM_006336)	15/16	ZDHHC12 (NM_032799)	2/5	10851	p		0			
0	0	0	0	0	0	1	0	−2	ANKRD39 (NM_016466)	3/4	ANKRD23 (NM_144994)	2/9	10889	F	Y	1	A		B,U
0	0	0	1	0	0	0	0	−1	C1orf43 (NM_001098616)	6/7	C1orf189 (NM_001010979)	3/4	11731	F		0			
0	0	0	0	0	0	1	1	−17	MAFG (NM_002359)	1/3	SIRT7 (NM_016538)	4/10	11845	t		0			
0	0	0	0	0	0	1	0	−1	SLC35E2 (NM_182838)	5/6	CDK11A (NM_024011)	2/20	11858	p	Y	2			
0	0	0	0	0	0	1	0	−17	FLCN (NM_144997)	13/14	PLD6 (NM_178836)	2/2	11886	l		0			
0	0	0	0	0	0	0	1	−1	CYB5R1 (NM_016243)	8/9	ADIPOR1 (NM_015999)	2/8	11901	p		0			
0	0	0	0	0	0	0	3	+22	SLC2A11 (NM_030807)	9/13	MIF (NM_002415)	2/3	11902	F		0		A	
0	0	0	0	1	0	1	0	−17	DHRS13 (NM_144683)	4/5	FLOT2 (NM_004475)	2/11	11963	F		0			
0	1	0	0	0	0	0	0	+12	PRR13 (NM_018457)	2/4	PCBP2 (NM_005016)	2/15	11992	p		0	A	A	
0	0	0	0	0	0	1	1	−16	CORO7 (NM_024535)	27/28	TIMM16 (NM_016069)	2/5	11994	F	Y	1	A		

Reads									Donor		Acceptor								
T1	T2	T3	N1	N2	N3	H	U	Chr	Gene	Exon	Gene	Exon	Distance	CDS	Ace	ESTs	Akiva	Parra	Maher
0	0	0	0	0	0	1	1	−7	URGCP (NM_001077663)	5/6	MRPS24 (NM_032014)	2/4	12282	p	Y	1	Y		
0	0	0	1	1	0	0	0	+2	VAMP8 (NM_003761)	2/3	VAMP5 (NM_006634)	2/3	12557	F		0	A	A	
0	0	0	1	1	0	0	0	+5	LRRC70 (NM_181506)	1/2	IPO11 (NM_016338)	28/30	12640	t	Y	12			
0	0	0	0	0	0	2	3	−22	CHCHD10 (NM_213720)	3/4	VPREB3 (NM_013378)	2/2	12929	F		0			
0	0	0	1	0	0	0	0	−1	PBXIP1 (NM_020524)	10/11	PMVK (NM_006556)	2/5	13156	F		0			
0	0	0	0	3	0	0	0	+17	RPL27 (NM_000988)	2/5	IFI35 (NM_005533)	2/7	13347	F		0			
0	0	0	0	0	0	0	1	+11	COX8A (NM_004074)	1/2	OTUB1 (NM_017670)	2/7	13542	p		0			
0	0	0	0	1	0	2	1	−19	CLDND2 (NM_152353)	2/4	ETFB (NM_001985)	2/6	13577	p	Y	2	Y		
0	0	0	0	0	0	0	1	−19	WIZ (NM_021241)	7/8	AKAP8L (NM_014371)	2/14	13588	p		0			
0	0	0	0	0	0	0	1	+19	TOMM40 (NM_006114)	6/10	APOE (NM_000041)	3/4	13693	F		0			
3	0	1	2	2	0	0	1	+19	SPINT2 (NM_021102)	6/7	C19orf33 (NM_033520)	2/4	13799	F	Y	1			
0	0	0	0	0	0	0	2	+14	BCL2L2 (NM_004050)	3/4	PABPN1 (NM_004643)	2/7	13981	F	Y	1	Y	Y	
0	0	0	0	1	0	0	0	−19	ZNF606 (NM_025027)	6/7	C19orf18 (NM_152474)	2/6	14003	F		0			
0	0	0	0	0	0	3	0	−1	CCDC19 (NM_012337)	11/12	VSIG8 (NM_001013661)	2/7	14031	p		1			
0	0	0	0	0	0	0	1	−11	LGR4 (NM_018490)	17/18	CCDC34 (NM_030771)	2/6	14073	p		0			
2	3	1	2	0	2	4	2	−15	C15orf38 (NM_182616)	5/6	AP3S2 (NM_005829)	2/6	14075	F	Y	2	Y		B
0	0	0	0	0	0	3	0	−22	CHCHD10 (NM_213720)	2/4	VPREB3 (NM_013378)	2/2	14175	l		0			
0	0	0	0	0	0	0	1	+8	GATA4 (NM_002052)	6/7	NEIL2 (NM_145043)	2/5	14362	p		0			
0	0	0	1	1	0	3	1	−22	KLHL22 (NM_032775)	6/7	SCARF2 (NM_153334)	2/11	14454	p		0	A		
0	0	0	0	0	0	0	1	−12	SCNN1A (NM_001038)	12/13	TNFRSF1A (NM_001065)	2/10	14482	F		1			
0	0	0	0	0	0	1	0	−1	CCDC19 (NM_012337)	11/12	VSIG8 (NM_001013661)	3/7	14652	p		0			
0	0	0	0	0	0	2	0	−13	DCUN1D2 (NM_001014283)	6/7	ADPRHL1 (NM_138430)	2/7	14722	F		0			
0	0	0	0	0	0	0	1	−19	ERF (NM_006494)	1/4	GSK3A (NM_019884)	2/11	14835	F	Y	1	Y		
0	0	0	0	0	0	0	1	+12	CDK2 (NM_001798)	6/7	RAB5B (NM_002868)	2/6	15621	p		0			
0	0	0	0	0	0	1	0	−12	ATF7 (NM_006856)	11/12	NPFF (NM_003717)	2/3	16133	l		0			
0	0	0	0	0	0	0	1	+6	SLC29A1 (NM_001078175)	12/14	HSP90AB1 (NM_007355)	2/12	16201	F		0			
0	1	0	0	0	0	0	0	−10	PPP3CB (NM_001142354)	12/13	ZMYND17 (NM_001024593)	2/7	16423	F		0			
0	0	0	0	0	0	2	0	+9	KIAA1984 (NM_001039374)	13/14	C9orf86 (NM_024718)	2/15	16458	F		0			
0	0	0	0	0	0	1	0	+7	SUN1 (NM_001130965)	18/20	GET4 (NM_015949)	2/9	16539	p		0			
0	0	0	0	0	0	0	1	+22	SHANK3 (NM_001080420)	22/23	ACR (NM_001097)	2/5	16833	F		0			
0	0	6	0	0	1	0	0	+12	HOXC6, HOXC4 (NM_153693)	1/3	HOXC5 (NM_018953)	2/2	16863	tl	Y	2	A		
0	0	0	1	1	0	1	0	−10	SLC25A16 (NM_152707)	8/9	DNA2 (NM_001080449)	2/20	16980	F		0			
0	0	0	0	0	0	1	0	−19	S1PR5 (NM_030760)	1/2	KEAP1 (NM_012289)	2/6	17317	t		0			
0	0	0	3	0	0	0	1	−5	PRKAA1 (NM_206907)	9/10	TTC33 (NM_012382)	2/5	17494	p		0			
0	0	0	0	0	0	0	1	−5	TRIM52 (NM_032765)	1/2	GNB2L1 (NM_006098)	2/8	17656	p		0			
0	0	0	0	0	0	1	0	+17	PPP1R1B (NM_032192)	6/7	STARD3 (NM_006804)	2/15	17754	p		0			
0	0	1	0	0	0	0	0	+8	ZFP41 (NM_173832)	2/3	GLI4 (NM_138465)	2/4	18019	p		0		A	U
0	0	0	0	0	0	1	0	+5	AHRR (NM_020731)	9/12	EXOC3 (NM_007277)	2/13	18143	p		0			
0	0	0	0	1	1	0	2	−15	ST20 (NM_001100879)	2/3	MTHFS (NM_006441)	2/3	18274	F	Y	2	A		
0	0	0	1	0	0	0	1	+17	RPL38 (NM_000999)	3/5	TTYH2 (NM_032646)	2/14	18294	p		0			
0	0	0	0	0	0	1	0	+16	CKLF (NM_181641)	2/3	CMTM1 (NM_181269)	2/3	18755	F		0		A	
0	0	0	0	0	0	1	0	+9	KIAA1984 (NM_001039374)	7/14	C9orf86 (NM_024718)	2/15	18897	p		0			
0	0	0	0	0	0	1	1	−1	MED8 (NM_052877)	7/8	ELOVL1 (NM_022821)	2/8	19422	p	Y	1	Y		
0	0	0	2	0	0	0	0	−16	NUDT21 (NM_007006)	6/7	AMFR (NM_001144)	2/14	19986	p		0			
0	0	0	0	1	0	0	0	−3	PFKFB4 (NM_004567)	13/14	SHISA5 (NM_016479)	2/6	20692	p		0			
0	0	0	0	0	0	1	0	+9	KIAA1984 (NM_001039374)	6/14	C9orf86 (NM_024718)	2/15	20738	p	Y	1			
1	0	1	0	3	0	2	1	−21	C21orf59 (NM_021254)	5/7	TCP10L (NM_144659)	3/5	20745	F	Y	1			
0	0	0	0	0	0	2	0	−5	SNCB (NM_001001502)	6/7	GPRIN1 (NM_052899)	2/2	21336	l		0			

Reads									Donor		Acceptor								
T1	T2	T3	N1	N2	N3	H	U	Chr	Gene	Exon	Gene	Exon	Distance	CDS	Ace	ESTs	Akiva	Parra	Maher
0	0	0	0	0	0	0	1	−11	ASAM (NM_024769)	5/7	HSPA8 (NM_006597)	2/9	21755	F		0			
0	0	0	1	0	0	0	0	−16	GFOD2 (NM_030819)	3/4	C16orf48 (NM_032140)	6/7	21900	p		0	A		
0	0	0	0	0	1	1	0	−2	CLEC4F (NM_173535)	6/7	FIGLA (NM_001004311)	2/5	21937	p		0			
0	0	0	0	0	0	0	2	+16	TBC1D24 (NM_020705)	2/7	ATP6V0C (NM_001694)	2/3	22104	p	Y	0			
1	0	2	2	0	0	0	0	+10	MSMB (NM_002443)	3/4	NCOA4 (NM_005437)	2/10	22271	p		0			
0	1	0	0	0	0	0	0	−6	HBS1L (NM_006620)	17/18	ALDH8A1 (NM_022568)	2/7	22362	F	Y	1			
0	0	2	0	0	0	0	0	+2	VAX2 (NM_012476)	2/3	ATP6V1B1 (NM_001692)	2/14	22372	p		0			
0	0	0	0	0	0	0	1	−1	MOBKL2C (NM_201403)	1/4	MKNK1 (NM_003684)	2/14	22430	t		1			
0	0	0	0	1	0	0	0	−14	RDH11 (NM_016026)	6/7	VTI1B (NM_006370)	2/6	22479	p		0	A		
0	0	0	7	1	0	3	7	+11	RBM14 (NM_006328)	1/3	RBM4 (NM_002896)	2/4	22642	p	Y	65	A		
0	0	0	0	1	0	0	2	−5	LMAN2 (NM_006816)	7/8	MXD3 (NM_031300)	2/6	22799	F	Y	1	Y		
0	1	0	1	0	1	0	0	+10	MSMB (NM_138634)	2/3	NCOA4 (NM_005437)	2/10	23291	F		3			
0	1	0	1	0	1	1	2	+8	ZFP41 (NM_173832)	2/3	GLI4 (NM_138465)	3/4	23363	p	Y	0		Y	U
0	0	0	1	1	0	0	0	−14	ENTPD5 (NM_001249)	15/16	FAM161B (NM_152445)	2/9	23404	F		0			
0	0	0	0	0	0	1	0	+6	CNPY3 (NM_006586)	5/6	GNMT (NM_018960)	2/6	24004	p		0	A	A	
0	0	0	0	0	0	1	0	+X	NDUFA1 (NM_004541)	1/3	AKAP14 (NM_178813)	2/7	24141	p		1			
0	0	0	0	0	0	1	0	+1	KIF1B (NM_015074)	46/47	PGD (NM_002631)	2/13	24254	F		0	A	A	
0	0	2	0	0	0	0	0	−21	C21orf59 (NM_021254)	5/7	TCP10L (NM_144659)	4/5	24329	F		0			
0	0	0	0	0	0	1	0	+1	NCDN (NM_001014839)	7/8	TFAP2E (NM_178548)	4/7	24420	F		0			
0	0	0	0	0	0	0	1	−19	PLAUR (NM_002659)	6/7	CADM4 (NM_145296)	2/9	24434	F		0			
0	0	0	0	0	0	2	1	+12	PXMP2 (NM_018663)	2/5	PGAM5 (NM_138575)	2/6	24481	F		0			
0	0	0	0	1	0	0	0	+7	INMT (NM_006774)	2/3	FAM188B (NM_032222)	2/18	24493	p	Y	1			
0	0	0	0	0	0	1	1	+19	MARCH2 (NM_016496)	5/6	HNRNPM (NM_031203)	2/17	24537	p		0			
0	0	0	0	0	0	0	1	+11	POLA2 (NM_002689)	17/18	CDC42EP2 (NM_006779)	2/2	24553	l		0			U
0	0	0	0	0	1	0	0	−7	ATP5J2 (NM_001003714)	2/3	PTCD1 (NM_015545)	2/8	24817	F		1			
0	0	0	0	1	0	0	1	−20	ZNF343 (NM_024325)	5/6	SNRPB (NM_003091)	2/7	24940	p	Y	1	Y		
0	0	0	2	0	0	0	0	−17	SUMO2 (NM_006937)	3/4	HN1 (NM_016185)	2/5	26080	p		0	A		
0	0	0	0	0	0	1	0	+12	ACAD10 (NM_001136538)	21/22	ALDH2 (NM_000690)	2/13	26172	F		1	Y	Y	
0	0	0	0	0	0	0	1	−3	ROPN1 (NM_017578)	5/7	CCDC14 (NM_022757)	5/12	26246	p		0			
0	0	0	0	0	0	1	1	+11	RBM14 (NM_006328)	1/3	RBM4 (NM_002896)	3/4	26392	F	Y	1	A		
0	0	0	0	0	0	1	0	−3	HIGD1A (NM_014056)	3/4	CCDC13 (NM_144719)	2/16	27676	p	Y	2			
0	0	0	0	1	0	3	5	−7	UPK3BL, POLR2J2 (NM_032959)	8/9	RASA4 (NM_006989)	2/21	27744	F		0			
0	0	0	1	0	0	0	1	−8	ZHX1 (NM_007222)	2/4	C8orf76 (NM_032847)	2/6	28128	t	Y	3			
0	0	0	0	0	0	0	1	−11	C11orf59 (NM_017907)	4/5	NUMA1 (NM_006185)	2/27	28377	p		0			
0	0	0	0	0	0	1	0	+3	TF (NM_001063)	16/17	SRPRB (NM_021203)	2/8	28437	p		0			
0	0	0	0	0	0	1	0	+2	KIAA1841 (NM_032506)	21/22	C2orf74 (NM_001143959)	2/4	28565	p	Y	0			
0	0	0	0	0	0	0	1	+20	TGIF2 (NM_021809)	2/3	C20orf24 (NM_018840)	2/4	28748	F	Y	2	Y		
0	0	0	1	3	0	0	1	+11	RBM14 (NM_006328)	1/3	RBM4 (NM_002896)	4/4	28969	l	Y	4	Y		
0	0	0	0	0	0	1	0	+17	CACNG4 (NM_014405)	3/4	CACNG1 (NM_000727)	2/4	28995	F		0			
0	0	0	0	0	0	1	0	+22	TCN2 (NM_000355)	7/9	SLC35E4 (NM_001001479)	2/2	29102	l		0			
0	0	0	0	0	0	1	0	−3	PIGZ (NM_025163)	1/3	NCBP2 (NM_007362)	2/4	29254	t		0			
0	0	0	0	0	0	1	0	+19	MAP1S (NM_018174)	6/7	FCHO1 (NM_015122)	5/29	29333	F		0			
0	0	1	1	0	0	0	0	+2	LIPT1 (NM_015929)	2/3	MRPL30 (NM_145212)	2/6	29750	t	Y	3			
0	0	0	0	0	0	0	1	−15	CLN6 (NM_017882)	1/7	CALML4 (NM_033429)	3/5	29820	p		0	A		
0	0	0	0	2	0	2	6	−7	UPK3BL, POLR2J2 (NM_032959)	7/9	RASA4 (NM_006989)	3/21	29841	p		0			
0	0	0	0	0	0	1	0	−16	CHST6 (NM_021615)	2/3	TMEM170A (NM_145254)	2/3	29977	t		0			
0	0	1	0	0	0	2	0	−2	ADCY3 (NM_004036)	20/21	C2orf79 (NM_001013663)	2/2	30142	F		0			
0	0	0	0	0	0	0	1	−12	WNT10B (NM_003394)	1/5	ARF3 (NM_001659)	2/5	30266	t		0			

Reads									Donor		Acceptor								
T1	T2	T3	N1	N2	N3	H	U	Chr	Gene	Exon	Gene	Exon	Distance	CDS	Ace	ESTs	Akiva	Parra	Maher
0	0	0	0	0	1	0	0	−10	SAR1A (NM_020150)	1/7	TYSND1 (NM_001040273)	2/2	30271	tl		0			
0	0	0	0	0	0	1	0	+11	ACCS (NM_032592)	7/15	EXT2 (NM_207122)	2/14	30306	F		0			
0	0	0	0	0	0	1	0	+3	GXYLT2 (NM_001080393)	6/7	PPP4R2 (NM_174907)	2/9	30357	p	Y	1			
0	0	0	0	0	0	1	0	−16	GCSH (NM_004483)	4/5	C16orf46 (NM_001100873)	4/4	30368	l		0			
0	0	0	0	0	0	1	0	+15	SCAMP5 (NM_138967)	3/7	PPCDC (NM_021823)	3/6	30635	p		0			
1	0	0	0	0	0	0	0	+16	TMC5 (NM_001105248)	21/22	CP110 (NM_014711)	3/15	30703	p		0			
0	0	1	0	0	0	0	0	−1	TAGLN2 (NM_003564)	1/5	CCDC19 (NM_012337)	2/12	32144	t	Y	4	Y		
0	0	0	0	0	0	0	1	−18	MYO5B (NM_001080467)	39/40	ACAA2 (NM_006111)	2/10	32490	p		0	A		
0	0	0	0	0	0	1	0	+11	ASRGL1 (NM_025080)	5/7	SCGB1A1 (NM_003357)	2/3	32969	F		0			
0	0	0	0	0	0	1	0	+11	ACCS (NM_032592)	5/15	EXT2 (NM_207122)	2/14	33001	F		0			
0	0	0	0	0	0	1	0	−1	PYCR2 (NM_013328)	5/6	LEFTY1 (NM_020997)	2/4	33175	p		0			
0	0	0	1	0	0	0	0	−16	GCSH (NM_004483)	1/5	C16orf46 (NM_001100873)	3/4	33992	l		0			
0	0	0	1	0	0	0	0	+15	PSMA4 (NM_002789)	8/9	CHRNA5 (NM_000745)	2/6	34112	F		0			
0	0	0	0	0	0	1	0	−19	KLF16 (NM_031918)	1/2	REXO1 (NM_020695)	2/16	34409	F		0			
0	0	0	0	0	0	0	1	−1	HSPB11 (NM_016126)	5/6	YIPF1 (NM_018982)	2/11	34440	p		0			
0	0	1	0	0	0	0	0	+1	SLC35A3 (NM_012243)	6/8	HIAT1 (NM_033055)	2/12	34488	F		0			
0	0	0	1	0	0	0	0	+16	TMC5 (NM_001105248)	21/22	CP110 (NM_014711)	4/15	35252	p		0			
0	0	0	0	0	0	1	0	+1	GPX7 (NM_015696)	2/3	FAM159A (NM_001042693)	2/3	35917	p		0			
0	0	0	0	0	0	1	0	−22	C22orf39 (NM_173793)	2/3	HIRA (NM_003325)	2/25	36599	p	Y	7	Y		
0	1	0	0	0	0	1	0	+5	SERF1B, SERF1A (NM_021967)	2/3	SMN2 (NM_017411)	2/9	36899	p		0	A		
0	0	0	0	0	0	1	0	−3	SMARCC1 (NM_003074)	26/28	CSPG5 (NM_006574)	3/5	37191	p		0			
0	1	0	0	1	0	1	0	−1	TSTD1 (NM_001113205)	1/3	F11R (NM_016946)	2/10	37526	F	Y	8			
0	0	2	0	0	0	0	0	−1	SLC45A3 (NM_033102)	4/5	ELK4 (NM_001973)	2/5	37969	F		0			
0	0	0	1	1	0	0	0	+7	FAM188B (NM_032222)	17/18	AQP1 (NM_198098)	2/4	39072	p		0			
0	0	0	0	0	0	0	1	+20	TPD52L2 (NM_199360)	8/9	DNAJC5 (NM_025219)	2/5	39096	p		0			
0	0	0	0	0	0	1	0	−X	PDZD4 (NM_032512)	1/8	IDH3G (NM_004135)	2/13	39387	F		0	A		
0	0	0	0	1	0	0	0	−8	TPD52 (NM_005079)	5/6	MRPS28 (NM_014018)	2/3	39439	F		0			
0	0	0	0	0	1	0	0	+2	LIPT1 (NM_145199)	1/2	MRPL30 (NM_145212)	4/6	39699	tl		0			
0	0	0	0	0	0	0	1	−11	FXYD2 (NM_001680)	5/6	DSCAML1 (NM_020693)	2/33	39855	p		0			
0	0	0	0	0	0	1	0	+1	VPS45 (NM_007259)	14/15	PLEKHO1 (NM_016274)	2/6	40359	p		0			
0	0	0	0	0	0	0	1	−8	LYPLA1 (NM_006330)	8/9	TCEA1 (NM_006756)	2/10	40519	F		0			
0	0	0	0	1	0	0	0	−X	RPS6KA3 (NM_004586)	11/22	EIF1AX (NM_001412)	3/7	41154	F		0			
0	0	0	0	0	0	0	1	+22	PEX26 (NM_017929)	3/6	TUBA8 (NM_018943)	2/5	41465	p	Y	2	Y		
0	1	0	0	0	0	1	1	−16	GCSH (NM_004483)	1/5	C16orf46 (NM_001100873)	4/4	42036	l		0			
0	0	0	0	0	0	0	1	−22	NPTXR (NM_014293)	4/5	DNAL4 (NM_005740)	3/4	42073	F		0			
0	0	0	1	0	0	0	0	+15	IVD (NM_002225)	11/12	BAHD1 (NM_014952)	2/7	42088	F		0			
1	1	0	0	0	0	0	1	−19	FBXO46 (NM_001080469)	1/2	SNRPD2 (NM_004597)	2/3	42282	t		0			
0	0	0	1	1	0	7	1	+20	SYS1 (NM_033542)	3/4	DBNDD2 (NM_001048221)	2/4	42773	p	Y	7	Y		
0	0	0	0	1	0	0	0	−15	DYX1C1 (NM_130810)	9/10	CCPG1 (NM_004748)	2/8	43063	p	Y	0			
0	0	0	0	0	0	0	1	−5	VDAC1 (NM_003374)	1/9	C5orf15 (NM_020199)	2/3	44626	t		0			
0	0	0	0	1	0	0	1	+11	FEN1 (NM_004111)	1/2	FADS2 (NM_004265)	2/12	44749	t		0			
0	0	0	1	1	0	0	0	+16	MLYCD (NM_012213)	4/5	OSGIN1 (NM_182980)	2/6	45272	p		0	A		
0	0	0	0	0	0	1	0	+4	FAM47E (NM_001136570)	3/8	STBD1 (NM_003943)	2/2	45300	l	Y	2			
0	0	0	0	1	0	0	0	+1	SRP9 (NM_003133)	2/3	EPHX1 (NM_000120)	2/9	45355	p	Y	2			
0	0	1	0	0	0	0	0	−19	KLK11 (NM_006853)	5/6	KLK7 (NM_005046)	6/6	45400	F		0			
0	0	0	0	1	0	0	0	+20	TPD52L2 (NM_003288)	5/7	DNAJC5 (NM_025219)	2/5	45514	p	Y	1			
0	0	0	0	0	0	0	1	−10	SAR1A (NM_020150)	1/7	AIFM2 (NM_032797)	2/9	46315	t	Y	2			
0	0	0	0	0	0	0	1	−13	C13orf38 (NM_001144981)	7/8	SOHLH2 (NM_017826)	2/11	46512	p	Y	11			

Reads									Donor		Acceptor								
T1	T2	T3	N1	N2	N3	H	U	Chr	Gene	Exon	Gene	Exon	Distance	CDS	Ace	ESTs	Akiva	Parra	Maher
0	0	0	0	0	0	2	1	+11	SDHAF2 (NM_017841)	3/4	C11orf66 (NM_145017)	5/14	46574	p	Y	2	A	A	
0	0	0	1	0	0	0	0	−6	SFRS18 (NM_015491)	1/11	COQ3 (NM_017421)	4/7	47729	t		0			
0	1	0	0	0	0	0	0	−11	DPAGT1 (NM_001382)	8/9	HYOU1 (NM_006389)	17/26	47936	F		0			
2	3	2	15	15	3	1	0	−7	AZGP1 (NM_001185)	2/4	GJC3 (NM_181538)	2/2	48142	F	Y	6			
0	0	1	0	0	0	0	0	+22	BIK (NM_001197)	1/5	TSPO (NM_000714)	2/4	48406	t		0			
0	0	0	0	0	0	0	1	+11	C11orf73 (NM_016401)	3/5	CCDC81 (NM_021827)	2/14	48520	p		0			
0	0	1	0	0	0	0	0	−7	STAG3L2 (NM_001025202)	6/8	GTF2IRD2 (NM_173537)	2/16	48744	p		0			
0	0	2	1	0	0	0	1	−X	HDAC8 (NM_018486)	10/11	CITED1 (NM_004143)	2/3	48798	F		0			
0	0	0	1	0	0	1	0	−9	TOPORS (NM_005802)	2/3	DDX58 (NM_014314)	2/18	49834	p	Y	12	Y		
0	0	0	1	0	0	1	0	−20	FKBP1A (NM_000801)	4/5	SDCBP2 (NM_080489)	2/9	51640	p		1	A		
0	0	0	0	0	0	0	1	−17	KIAA0753 (NM_014804)	18/19	PITPNM3 (NM_031220)	2/20	51696	p		0			
0	0	1	1	0	0	0	0	−5	PPP2CA (NM_002715)	1/7	SKP1 (NM_170679)	2/6	51737	F	Y	1			
0	0	0	0	0	0	1	0	+3	NCK1 (NM_006153)	2/4	IL20RB (NM_144717)	2/7	52238	F		0	A		
0	0	0	0	0	0	1	0	+2	ADAM23 (NM_003812)	24/26	LOC200726 (NM_001102659)	3/3	52498	F		0			
0	0	0	0	0	0	1	0	+1	RPL11 (NM_000975)	5/6	TCEB3 (NM_003198)	2/11	53112	F	Y	2			
0	0	0	0	0	0	1	0	+15	MAP2K5 (NM_145160)	21/22	LBXCOR1 (NM_001031807)	2/10	53183	p		0			
0	0	0	0	0	0	2	0	+15	PLEKHO2 (NM_025201)	5/6	ANKDD1A (NM_182703)	2/15	54221	p		0			
0	0	0	0	0	0	0	1	+1	RPL11 (NM_000975)	4/6	TCEB3 (NM_003198)	2/11	54229	F	Y	2			
0	1	0	1	0	0	0	1	+11	SDHAF2 (NM_017841)	1/4	C11orf66 (NM_145017)	5/14	54505	F		0	A	A	
0	0	0	1	0	0	0	1	−7	POLR2J2 (NM_032959)	4/9	RASA4 (NM_006989)	2/21	55107	p		2			
0	0	1	0	2	0	4	2	+9	C9orf30 (NM_080655)	2/3	TMEFF1 (NM_003692)	2/10	56408	F	Y	5	A	Y	
0	0	0	0	1	0	0	0	−9	BICD2 (NM_015250)	7/8	IPPK (NM_022755)	2/13	56571	F		0			
0	0	0	0	0	0	1	0	−7	POLR2J2 (NM_032959)	4/9	RASA4 (NM_006989)	3/21	56795	p		0			
0	0	0	1	0	0	0	1	+5	UBE2D2 (NM_181838)	7/8	CXXC5 (NM_016463)	2/3	56902	p		0			
0	0	0	2	0	0	1	1	+1	C1orf151 (NM_001032363)	1/4	NBL1 (NM_005380)	2/4	57901	p		2			
0	0	0	1	0	0	0	0	+5	PCBD2 (NM_032151)	2/4	CATSPER3 (NM_178019)	2/8	59472	p		0			
0	0	0	0	0	0	0	2	+15	PLEKHO2 (NM_025201)	5/6	ANKDD1A (NM_182703)	4/15	60345	F		1			
0	0	0	1	0	0	0	0	−1	CTBS (NM_004388)	6/7	GNG5 (NM_005274)	3/4	61286	F	Y	15	Y		
0	0	0	0	1	0	0	0	+3	LRRC33 (NM_198565)	2/3	PIGX (NM_017861)	2/6	62210	p		1	Y		
0	0	0	1	0	0	0	0	+18	ELAC1 (NM_018696)	3/4	SMAD4 (NM_005359)	2/12	62356	p	Y	1	A		
0	0	0	0	0	0	0	1	+1	NTRK1 (NM_001007792)	2/17	PEAR1 (NM_001080471)	3/23	62554	F		0			
0	0	0	0	0	0	0	1	−2	SNRPG (NM_003096)	1/4	TIA1 (NM_022173)	3/13	62763	p		0			
0	0	0	0	0	0	0	1	+22	CYTSA (NM_015330)	14/17	ADORA2A (NM_000675)	2/3	63810	p		0			
0	0	0	0	0	0	1	0	−20	ACSS1 (NM_032501)	2/14	C20orf3 (NM_020531)	2/9	64065	F		0			
0	0	0	0	0	0	1	0	+15	PLEKHO2 (NM_025201)	5/6	ANKDD1A (NM_182703)	5/15	64490	F		0			
0	0	1	0	0	0	0	0	+1	APITD1 (NM_198544)	1/5	PEX14 (NM_004565)	2/9	64705	F		0			
0	0	0	0	0	0	0	1	+1	SFT2D2 (NM_199344)	1/8	TBX19 (NM_005149)	2/8	65017	p	Y	1	Y		
0	0	0	0	0	0	0	1	+12	HOXC10 (NM_017409)	1/2	HOXC4 (NM_014620)	2/4	67149	p		0	A		
0	0	0	0	0	0	1	0	+20	SFRS6 (NM_006275)	5/6	L3MBTL (NM_015478)	5/19	68058	p		0			
0	0	0	0	0	0	2	0	+7	RHBDD2 (NM_001040457)	4/5	POR (NM_000941)	2/16	70140	F		0	A		
0	0	0	0	0	0	0	1	+21	WRB (NM_004627)	3/5	SH3BGR (NM_007341)	2/7	70538	F	Y	2	Y		
0	0	0	0	0	0	1	0	+22	CYTSA (NM_015330)	14/17	ADORA2A (NM_000675)	3/3	71262	l		0			
0	0	0	1	0	0	0	0	+18	ELAC1 (NM_018696)	2/4	SMAD4 (NM_005359)	2/12	72358	p	Y	10	Y		
0	0	2	0	1	0	4	5	−20	FKBP1A (NM_054014)	2/4	SDCBP2 (NM_080489)	2/9	72398	p	Y	14	Y		
0	0	0	0	0	1	2	1	−3	HACL1 (NM_012260)	16/17	COLQ (NM_005677)	2/17	73720	p		0			
0	0	0	0	0	0	0	1	+19	EIF3K (NM_013234)	4/8	ACTN4 (NM_004924)	2/21	74497	l		0			
0	0	0	0	0	1	0	0	−17	RAD51L3 (NM_002878)	9/10	RFFL (NM_057178)	2/7	74639	p		1	Y		
0	0	0	0	0	0	0	1	+12	NDUFA9 (NM_005002)	10/11	GALNT8 (NM_017417)	7/11	75632	F		0			

Reads									Donor		Acceptor								
T1	T2	T3	N1	N2	N3	H	U	Chr	Gene	Exon	Gene	Exon	Distance	CDS	Ace	ESTs	Akiva	Parra	Maher
0	0	0	0	0	0	1	0	+15	PLEKHO2 (NM_025201)	3/6	ANKDD1A (NM_182703)	7/15	75783	F		0			
0	0	0	0	0	0	1	0	−2	NEUROD1 (NM_002500)	1/2	CERKL (NM_001030311)	2/14	76350	t	Y	0			
0	0	0	0	1	0	8	4	−6	RPS10 (NM_001014)	5/6	NUDT3 (NM_006703)	2/5	76396	F	Y	3			
0	0	0	0	0	0	1	0	−15	SGK269 (NM_024776)	5/6	TSPAN3 (NM_005724)	2/7	76749	F		0			
0	0	0	2	0	0	0	1	+14	CHURC1 (NM_145165)	3/4	FNTB (NM_002028)	2/12	78168	F	Y	6			
0	0	0	0	0	0	1	0	+3	MRAS (NM_012219)	2/6	ESYT3 (NM_031913)	2/23	78971	p		0			
0	0	0	0	3	0	0	0	+14	CHURC1 (NM_145165)	2/4	FNTB (NM_002028)	2/12	80122	p	Y	2			
0	0	0	1	0	0	0	1	−6	RNASET2 (NM_003730)	6/9	RPS6KA2 (NM_001006932)	2/22	80635	p		0			
0	0	0	0	0	0	0	1	+21	BACE2 (NM_012105)	8/9	FAM3B (NM_206964)	2/7	81051	F		0			
0	0	0	0	0	0	2	0	+11	P2RY6 (NM_176797)	1/3	ARHGEF17 (NM_014786)	2/21	82233	t		0			
0	0	0	0	0	0	0	1	−1	NSL1 (NM_015471)	4/6	BATF3 (NM_018664)	2/3	85306	p		0			
0	0	0	1	0	0	0	0	+5	PCBD2 (NM_032151)	2/4	CATSPER3 (NM_178019)	3/8	85806	F		0			
0	0	0	0	1	0	0	1	+14	TTLL5 (NM_015072)	31/32	C14orf179 (NM_001102564)	2/9	86660	p		1			
0	0	0	0	0	0	1	0	+3	HDAC11 (NM_024827)	3/10	FBLN2 (NM_001004019)	2/18	86750	p		0			
0	0	0	0	1	0	0	0	−2	WDR92 (NM_138458)	7/8	C1D (NM_006333)	2/5	87382	p		0			
0	0	0	0	0	0	1	0	+3	HDAC11 (NM_024827)	2/10	FBLN2 (NM_001004019)	2/18	88920	F		0			
0	0	0	0	0	0	1	0	−17	RAD51L3 (NM_133629)	2/7	RFFL (NM_057178)	2/7	92549	p		0	A		
0	0	1	0	0	0	4	0	−2	GPR75 (NM_006794)	1/2	ASB3 (NM_016115)	2/10	94242	t	Y	8	A		
0	0	0	0	0	0	1	0	−5	CDKL3 (NM_001113575)	12/13	PPP2CA (NM_002715)	2/7	96458	F		0			
0	0	0	0	0	0	0	1	−7	FBXL18 (NM_024963)	4/5	TNRC18 (NM_001080495)	3/30	96635	p		0			
0	0	0	1	0	0	9	0	−1	CTNNBIP1 (NM_020248)	5/6	CLSTN1 (NM_001009566)	2/19	97792	F		0			
0	0	0	0	0	0	3	0	−2	CNRIP1 (NM_015463)	2/3	PPP3R1 (NM_000945)	2/6	100025	F		0			
0	0	0	0	0	0	1	0	+3	VWA5B2 (NM_138345)	4/19	EIF4G1 (NM_004953)	26/26	100993	F		0			
0	0	1	0	0	0	0	0	+3	RPSA (NM_002295)	5/7	MOBP (NM_182935)	4/4	101605	l		0			
0	1	0	0	0	0	0	0	−7	FBXL18 (NM_024963)	4/5	TNRC18 (NM_001080495)	5/30	101894	p		0			
0	0	0	0	0	0	1	0	−3	C3orf31 (NM_138807)	6/7	VGLL4 (NM_014667)	2/6	106469	p		0			
0	0	0	0	0	0	0	1	−17	FAM18B2 (NM_145301)	5/6	CDRT4 (NM_173622)	4/4	107581	l		0			
0	0	0	0	0	0	1	0	−7	NUDCD3 (NM_015332)	5/6	CAMK2B (NM_001220)	2/24	108071	p		0			
0	0	0	0	0	0	1	0	+14	FCF1 (NM_015962)	4/8	YLPM1 (NM_019589)	19/21	113113	F		0			
0	0	0	0	0	0	1	0	+2	RAMP1 (NM_005855)	1/3	UBE2F (NM_080678)	2/10	113363	p		0			
0	0	0	0	0	0	0	1	−12	POC1B (NM_172240)	9/12	DUSP6 (NM_001946)	2/3	115744	p		0			
0	0	0	6	1	0	5	0	−3	TMEM111 (NM_018447)	1/8	CIDEC (NM_022094)	5/6	116437	p		0			
0	0	0	0	1	0	0	0	−15	C15orf40 (NM_144597)	1/3	HOMER2 (NM_004839)	2/9	117395	p		0			
0	0	0	0	0	0	1	0	+12	PPFIBP1 (NM_177444)	4/5	MRPS35 (NM_021821)	8/8	120065	F		0			
0	0	0	0	0	0	0	2	−3	RAD18 (NM_020165)	12/13	OXTR (NM_000916)	2/4	121795	p		0			
0	0	0	0	0	0	1	0	−10	TIMM23 (NM_006327)	6/7	AGAP7 (NM_001077685)	2/7	123745	F		0			
0	0	1	0	0	0	0	0	+15	IDH3A (NM_005530)	1/11	DNAJA4 (NM_001130182)	6/7	130614	p		0			
0	0	0	0	0	0	1	0	+1	RNF115 (NM_014455)	1/9	PDZK1 (NM_002614)	3/10	135700	p		0			
0	0	1	0	0	0	0	0	+16	ITGAL (NM_002209)	29/31	PRR14 (NM_024031)	9/12	136631	p		0			
0	0	0	0	0	0	0	1	−3	RAD18 (NM_020165)	12/13	OXTR (NM_000916)	4/4	137162	l		0			
0	0	0	1	0	0	0	0	−1	TAF13 (NM_005645)	2/4	CLCC1 (NM_015127)	10/12	137568	p		0			
0	0	0	0	0	0	1	0	−7	PRKAG2 (NM_016203)	5/16	RHEB (NM_005614)	2/8	141054	F		0			
1	0	2	4	5	0	4	7	+17	BPTF (NM_004459)	9/30	KPNA2 (NM_002266)	2/11	142944	p		0			B
0	0	0	0	0	1	0	0	+12	TULP3 (NM_003324)	1/12	TEAD4 (NM_201443)	9/11	146979	p		0			
0	0	0	0	1	0	0	0	+11	FERMT3 (NM_031471)	7/15	RPS6KA4 (NM_003942)	10/17	148773	F		0			
0	0	0	0	0	0	0	1	−11	CTSC (NM_001814)	5/7	RAB38 (NM_022337)	2/3	150574	F		0			
0	0	0	0	0	0	1	0	+11	PGAP2 (NM_014489)	1/7	STIM1 (NM_003156)	2/12	158900	t		0			
0	0	0	0	0	0	0	1	−X	FAM122B (NM_145284)	8/9	PLAC1 (NM_021796)	2/3	179698	p		0			

Reads									Donor		Acceptor								
T1	T2	T3	N1	N2	N3	H	U	Chr	Gene	Exon	Gene	Exon	Distance	CDS	Ace	ESTs	Akiva	Parra	Maher
0	0	0	0	0	0	2	0	+9	NOXA1 (NM_006647)	9/14	ARRDC1 (NM_152285)	4/8	180540	F		0			
0	0	0	0	0	0	0	1	+1	DEGS1 (NM_003676)	1/3	CNIH4 (NM_014184)	3/5	182460	p		0			
0	0	1	0	2	0	0	0	−19	ELL (NM_006532)	7/12	KIAA1683 (NM_025249)	3/4	184025	p		0			
0	0	0	0	1	0	0	0	+2	PPP1CB (NM_206876)	2/9	WDR43 (NM_015131)	15/18	189284	p		0	A		
0	0	0	0	1	0	0	0	+6	C6orf153 (NM_033112)	2/7	CUL9 (NM_015089)	36/41	197183	p		0			
1	0	2	0	0	1	1	0	+8	ZNF596 (NM_173539)	1/6	FBXO25 (NM_183421)	3/11	198925	t		0			

[0]H: Reads observed in HBR sample. U: Reads observedin UHR sample. CDS: F = Full CDS, t = new TSS preserving $3'$ stop codon, l = $3'$ frameshift extending to last exon, p = $3'$ frameshift with premature termination codon (PTC), tl = new TSS and frameshift extending to last exon; tp = new TSS and frameshift with PTC. Ace: Y = Fusion splice listed in AceView. Akiva: Y = Observed directly, A = Observed an alternate fusion between these genes. Parra: Y = Observed. Maher: B = Observed in human brain reference, U = Observed universal human reference.
