# Supplementary material for: Deep RNA sequencing analysis of readthrough gene fusions in human prostate adenocarcinoma and reference samples
Source: BMC Med Genomics. 2011 Jan 24;4:11. doi: 10.1186/1755-8794-4-11 (PMC3041646; doi:10.1186/1755-8794-4-11)
Supplement: Additional file 2 — Supporting reads for TIC events. [file 1755-8794-4-11-S2.PDF]

Supplemental Table S2. Supporting reads for TIC events

- DUS3L–PRR22, -19:5736394..5735450 (distance: 943 nt)
  Donor exon (score:0.976824):NM_020175.exon12/13
  Acceptor exon (score:0.996911): NM_001134316.exon2/3
  Translation: tle
  5′ gene domains: Dus 2.8e-58
  3′ gene domains:
  TIC domains: Dus 3.1e-58
  AceView: DUS3L.bApr07
  Supporting ESTs: BP215972 (cerebrum normal)

```
 T3  CAGCCGACTGGATCCGCATCAG  GCTCCTTGAAC
 N1      CGACTGGATCCGCATCAG  GCTCCTTGAACCTGTATCATCCCCCAGACCCAGAGAAAGAGGTGTTTCCAGCCCCAC
```

- STK19–C4A—C4B, +6:32056557..32058061 (distance: 1503 nt)
  Donor exon (score:0.999088):NM_032454.exon7/8, NM_004197.exon7/8
  Acceptor exon (score:0.999457): NM_007293.exon2/41, NM_001002029.exon2/41
  Translation: full
  5′ gene domains: Stk19 8.4e-40
  3′ gene domains: A2M_comp 2.5e-74, A2M_recep 4.8e-29, A2M 6.4e-28, A2M_N_2 2.9e-18, NTR 1.1e-18, A2M_N 5.5e-18, Thiol-ester_cl 5e-14, ANATO 5e-11
  TIC domains: Stk19 1.5e-33, A2M_comp 3.3e-74, A2M_recep 5.9e-29, A2M 7.8e-28, A2M_N_2 3.6e-18, NTR 1.4e-18, A2M_N 6.8e-18, Thiol-ester_cl 6.1e-14, ANATO 6e-11

```
 N1  GCGGCTTGGCCTCACCTAgCATGTGCACGACCTCATTGGGGCCCAGCTAGTGGACTG  GTTGCTCTTGTTCTCTCC
HBR                               ACCTCATTGGGGCCCAGCTAGTGGACTG  GgTGCTCTTGTTCTCTCCTTCT
```

- TYMP–SCO2, -22:49311296..49309719 (distance: 1576 nt)
  Donor exon (score:0.990073):NM_001113756.exon8/9, NM_001113755.exon9/10, NM_001953.exon9/10
  Acceptor exon (score:0.998065): NM_005138.exon2/2
  Translation: tle
  5′ gene domains: Glycos_transf_3 7e-58, Glycos_trans_3N 5.3e-14, PYNP_C 2.5e-10
  3′ gene domains: SCO1-SenC 1.5e-45
  TIC domains: Glycos_transf_3 5.6e-58, Glycos_trans_3N 4.6e-14, PYNP_C 2e-06
  AceView: ECGF1andSCO2.iApr07
  Supporting ESTs: CT004063 (uncharacterized tissue normal), BG392981 (uncharacterized tissue neoplasia)

```
 T1                                          GCGCCGTG  GAGCATCAGATCCATGCTGCTGCTG
 N1  GGGTGGGCGCAGAGCTGCTGGTCGACGTGGGTCAGAGGCTGCGCCGTG  GAGCATCAGATCCATGCTGCTGCTGAC
HBR          GCAGAGCTGCTGGTCGACGTGGGaCAGAGGCTGCGCCGTG  GAGCATCAGA
HBR                           CGTGGGaCAGAGGCTGCGCCGTG  GAGCATCAGATCCATGCTGCTGCTGAC
UHR                   CTGGTCGACGTGGGaCAGAGGCTGCGCCGTG  GAGCATCAGATCCATGCTG
UHR                                    GAGGCTGCGCCGTG  GAGCATCAGATCCATGCTGCTGCTGACTCGGAGCCC
```

- COL7A1–UCN2, -3:48577220..48575573 (distance: 1646 nt)
  Donor exon (score:0.999014):NM_000094.exon117/118
  Acceptor exon (score:0.998618): NM_033199.exon2/2
  Translation: tle
  5′ gene domains: Collagen 1.5e-09, fn3 2.8e-16, VWA 5.2e-42, Kunitz_BPTI 7.5e-18
  3′ gene domains: UCN2 7.6e-23
  TIC domains: Collagen 1.5e-09, fn3 2.8e-16, VWA 5.2e-42, Kunitz_BPTI 7.5e-18
  AceView: COL7A1andUCN2andPFKFB4.tApr07
  Supporting ESTs: BE272482 (kidney neoplasia)

```
UHR  GGGTGGTCCAGAGCCAGGGGACAG  CCTGACCTCACGATGACCAGGTGTGC
UHR         CCAGAGCCAGGGGACAG  CCTGACCTCACGATGACCAGGTGTGCTCTGCTG
```

- CSNK2B–LY6G5B, +6:31745264..31746912 (distance: 1647 nt)
  Donor exon (score:0.998583):NM_001320.exon6/7
  Acceptor exon (score:0.998987): NM_021221.exon2/3
  Translation: ptc
  5′ gene domains: CK_II_beta 5.2e-85
  3′ gene domains:
  TIC domains: CK_II_beta 7.9e-82
  AceView: CSNK2BandLY6G5B.aApr07
  Supporting ESTs: BX363221 (cervix neoplasia), BX363222 (cervix neoplasia)

```
UHR  CTGCCAACCAGTTTGTGCCCAG  TTCCTGTTCCCGACATCCGGACGTGCCA
```

- IL17RC–CRELD1, +3:9949387..9951104 (distance: 1716 nt)
  Donor exon (score:0.998710):NM_032732.exon16/18, NM_153461.exon17/19, NM_153460.exon17/19
  Acceptor exon (score:0.999744): NM_001031717.exon2/12, NM_001077415.exon2/11
  Translation: ptc
  5′ gene domains: SEFIR 1e-38
  3′ gene domains: DUF3456 7.5e-09, EGF_CA 1.8e-08
  TIC domains:

```
HBR  CCTTCTCAAAAAGGATCACGCGAAAG  GTCCCCAGCCTGGGTAAAGATGGC
```

- TM9SF1–IPO4, -14:23729426..23727684 (distance: 1741 nt)
  Donor exon (score:0.995853):NM_006405.exon5/6
  Acceptor exon (score:0.979529): NM_024658.exon2/30
  Translation: ptc
  5′ gene domains: EMP70 4.1e-185
  3′ gene domains: IBN_N 7.1e-07
  TIC domains: EMP70 9.7e-139

```
 N2  ACATGACTGTTGGAGGCTTCCTGCCTTTCAG  GCCACGGAACAGCTCCAGA
```

- TYMP–SCO2, -22:49311541..49309719 (distance: 1821 nt)
  Donor exon (score:0.996775):NM_001113756.exon7/9, NM_001113755.exon8/10, NM_001953.exon8/10
  Acceptor exon (score:0.998065): NM_005138.exon2/2
  Translation: tle
  5′ gene domains: Glycos_transf_3 7e-58, Glycos_trans_3N 5.3e-14, PYNP_C 2.5e-10
  3′ gene domains: SCO1-SenC 1.5e-45
  TIC domains: Glycos_transf_3 4.1e-58, Glycos_trans_3N 3.9e-14
  AceView: ECGF1andSCO2.nApr07
  Supporting ESTs: BF036691 (ovary neoplasia), BM787227 (uncharacterized tissue neoplasia), BG337936 (uterus neoplasia), BM787182 (uncharacterized tissue neoplasia)

```
 T1                GAGCTGCTGGCGCCCGCAGATG  GAGCATCAGAT
 N2  CCGGGAGCAGGAGGAGCTGCTGGCGCCCGCAGATG  GAGCATCAGATCCAT
 N2    GGGAGCAGGAGGAGCTGCTGGCGCCCGCAGATG  GAGCATCAGATCCATGC
 N2                            CGCAGATG  GAGCATCAGATCCATGCTGCTGCTGACTCGGAGCCCCACAGC
UHR                   GCTGGCGCCCGCAGATG  GAGCATCAGATCCATGCTGCTGCTGACTCGGAG
UHR                         GCCCGCAGATG  GAGCATCAGATCCATGCTGCTGCTGACTCGGAGCCCCAC
```

- LIME1–SLC2A4RG, +20:61840296..61842174 (distance: 1877 nt)
  Donor exon (score:0.999320):NM_017806.exon5/6
  Acceptor exon (score:0.999693): NM_020062.exon2/8
  Translation: full
  5′ gene domains:
  3′ gene domains:
  TIC domains:
  AceView: LIME1andSLC2A4RG.aApr07
  Supporting ESTs: DB187986 (liver neoplasia), AI671731 (kidney normal), DA642562 (liver normal)

```
UHR  GCAGGGGAAGACTGAGGTGACCCCGGCCtCTCAG  GGCTCTTCCGTGGGCG
UHR            GACTGAGGTGACCCCGGCCGCTCAG  GGCTCTTCCGTGGGCGGCGGCcTCG
```

- SLC12A4–LCAT, -16:66536773..66534616 (distance: 2156 nt)
  Donor exon (score:0.997355):NM_005072.exon22/24
  Acceptor exon (score:0.865231): NM_000229.exon2/6
  Translation: ptc
  5′ gene domains: AA_permease 4.8e-39, KCl_Cotrans_1 1.4e-15
  3′ gene domains: LACT 1.7e-118
  TIC domains: AA_permease 4.6e-39, KCl_Cotrans_1 1.4e-15

```
UHR  CCCTGACAATTTCCGGGAGCTGGTGCACATTAAGCC  TGCCCGGCTGCCTG
```

- BLOC1S1–RDH5, +12:54399274..54401740 (distance: 2465 nt)
  Donor exon (score:0.995905):NM_001487.exon3/4
  Acceptor exon (score:0.999879): NM_002905.exon3/5
  Translation: ptc
  5′ gene domains: GCN5L1 7.8e-52
  3′ gene domains: adh_short 1.7e-22
  TIC domains: GCN5L1 1.8e-33
  AceView: bloflabu.aApr07

Supporting ESTs: AW404869 (lymph node normal), DA498402 (brain normal), N45654 (brain uncharacterized histology), DA739721 (testis neoplasia), DA782141 (brain normal), DA786255 (brain normal), DA864590 (placenta normal), DA123793 (cerebellum normal), DA317028 (cerebrum normal), BG722558 (testis normal), DA811047 (brain normal), DB472774 (cerebrum uncharacterized histology), DA243440 (brain normal), DA506510 (brain normal)

```
HBR  AGTGGATCGGAATGGTGGAGAACTTCAACCAGGCACTCAAG  GGCTTTTTG
HBR                 GAGAACTTCAACCAGGCACTCAAG  GGCTTTTTGGTCTGGTGAATAATGCT
```

- NEDD8–MDP1, -14:23757179..23754708 (distance: 2470 nt)
  Donor exon (score:0.995433):NM_006156.exon3/4
  Acceptor exon (score:0.995389): NM_138476.exon3/6
  Translation: full
  5′ gene domains: ubiquitin 2.5e-27, Rad60-SLD 6.8e-18
  3′ gene domains:
  TIC domains: ubiquitin 6.4e-17, Rad60-SLD 1.8e-11
  AceView: NEDD8andMDP-1andCHMP4A.fApr07
  Supporting ESTs: DN993851 (brain normal)

```
UHR  CAGTGGCAAGCAGAT  TGATGGAACTGTACGAGATAGGCGGGGCCAAGACG
```

- HARS2–ZMAT2, +5:140057847..140060588 (distance: 2740 nt)
  Donor exon (score:0.999046):NM_012208.exon12/13
  Acceptor exon (score:0.999758): NM_144723.exon2/6
  Translation: full
  5′ gene domains: tRNA-synt_2b 1.9e-18, HGTP_anticodon 2.9e-17
  3′ gene domains:
  TIC domains: tRNA-synt_2b 3.3e-18, HGTP_anticodon 6.9e-14

```
 T3  AGCAGAGAGGAG  ACAAAAAACTTGGACTTTCGC
```

- MRPL2–CUL7, -6:43131261..43128512 (distance: 2748 nt)
  Donor exon (score:0.992201):NM_015950.exon6/7
  Acceptor exon (score:0.999575): NM_014780.exon2/26
  Translation: ptc
  5′ gene domains: Ribosomal_L2_C 7.5e-33, Ribosomal_L2 6.9e-28
  3′ gene domains: Cullin 6.1e-85, Cul7 2.3e-33, APC10 6.5e-09
  TIC domains: Ribosomal_L2 5.1e-28, Ribosomal_L2_C 5.3e-15

```
HBR  AGCTGCCCTCTAAGAGGCAGATGCAG  GTGCCAGGATGGTGGGAGAACTCC
```

- AIFM3–LZTR1, +22:19664413..19667316 (distance: 2902 nt)
  Donor exon (score:0.985155):NM_144704.exon19/21, NM_001018060.exon19/20
  Acceptor exon (score:0.998465): NM_006767.exon2/21
  Translation: full
  5′ gene domains: Pyr_redox_2 1e-43, Pyr_redox 5.4e-17, Rieske 1.3e-15
  3′ gene domains: Kelch_1 4.6e-08, BTB 1.2e-20, Kelch_2 1.5e-07
  TIC domains: Pyr_redox_2 5.2e-43, Pyr_redox 1.6e-16, Rieske 4.1e-15, Kelch_1 8.1e-08, BTB 2.3e-20, Kelch_2 2.6e-07
  AceView: AIFM3andLZTR1.bApr07
  Supporting ESTs: DA241116 (brain normal)

```
HBR  CAGGgCGTGCCATCCGGAAGCGGGAGGTGGA  GCGCAGCAAGCACACAGTG
```

- MIA–RAB4B, +19:45974824..45977764 (distance: 2939 nt)
  Donor exon (score:0.998321):NM_006533.exon3/4
  Acceptor exon (score:0.935274): NM_016154.exon2/8
  Translation: ptc
  5′ gene domains: SH3_2 3e-10
  3′ gene domains: Ras 5.7e-58, Miro 2.6e-21, Arf 1.2e-13
  TIC domains: SH3_2 3e-10
  AceView: MIA_.lApr07
  Supporting ESTs: AA461365 (embryonic tissue normal)

```
HBR  GCAAAGTCGATGTGAAGACAGAC  ACTTCCTCTTCAAATTCCTGGTGATTG
```

- CHD4–NOP2, -12:6550296..6547349 (distance: 2946 nt)
  Donor exon (score:0.998868):NM_001273.exon39/40
  Acceptor exon (score:0.986007): NM_001033714.exon2/16, NM_006170.exon2/16
  Translation: ptc
  5′ gene domains: CHDCT2 2.6e-94, DUF1086 6e-79, SNF2_N 3.9e-78, CHDNT 2.6e-30, DUF1087 1.8e-26, PHD 2.4e-11, Chromo 9.3e-13, Helicase_C 1.1e-17, HDA2-3 1.6e-09

3′ gene domains: Nol1_Nop2_Fmu 1.3e-109, P120R 1.2e-07
TIC domains: CHDCT2 2.7e-94, DUF1086 6.1e-79, SNF2_N 4e-78, CHDNT 2.6e-30, DUF1087 1.8e-26, PHD 2.5e-11, Chromo 9.5e-13, Helicase_C 1.1e-17, HDA2-3 1.7e-09

```
HBR  CGCCTGGCAAACCGGGCACCCGAACCTACCCCACAGCAG  TACCATGGGGC
```

- GMIP–LPAR2, -19:19602280..19599093 (distance: 3186 nt)
  Donor exon (score:0.999297):NM_016573.exon20/21
  Acceptor exon (score:0.999764): NM_004720.exon2/3
  Translation: ptc
  5′ gene domains: RhoGAP 2.8e-49
  3′ gene domains: 7tm_1 2.5e-35, 7TM_GPCR_Srsx 7.8e-08
  TIC domains: RhoGAP 2.3e-49

```
 N1  AAGACACCAAAGATGGGGGAGGGGAAG  ATGGTCATCATGGGCCAGTGCTACTACAACGAGACCATCGGCTTCTTC
```

- GPSM3–PBX2, -6:32267464..32264259 (distance: 3204 nt)
  Donor exon (score:0.999426):NM_022107.exon7/8
  Acceptor exon (score:0.987278): NM_002586.exon3/9
  Translation: ptc
  5′ gene domains: GoLoco 1.7e-08
  3′ gene domains: PBC 3.1e-101, Homeobox 1.6e-19
  TIC domains: GoLoco 7.4e-08

```
UHR  TCCTCAGTCACCAG  GCCTCAGCATTCGGAGCTCCCAGGAGGAGGAGCCGG
```

- STARD3–TCAP, +17:35072123..35075495 (distance: 3371 nt)
  Donor exon (score:0.983186):NM_006804.exon14/15
  Acceptor exon (score:0.999471): NM_003673.exon2/2
  Translation: tle
  5′ gene domains: MENTAL 4e-73, START 5.3e-47
  3′ gene domains: Telethonin 1.7e-116
  TIC domains: MENTAL 3.4e-73, START 6.8e-39

```
 N1  TGCACCTTTGTCTGGATTCTTAATACAGATCTCAAG  CTGCTCCCTGCATGAGGAGGACACCCAGAGACATGAGAC
UHR                     CTTAATACAGATCTCAAG  CTGCTCCCTGCATGAGGAGGACACCCAGAGAC
```

- TSC22D4–C7orf61, -7:99903111..99899245 (distance: 3865 nt)
  Donor exon (score:0.998599):NM_030935.exon4/5
  Acceptor exon (score:0.999405): NM_001004323.exon2/3
  Translation: full
  5′ gene domains: TSC22 2.6e-29
  3′ gene domains:
  TIC domains:

```
UHR  AAAATCGAGCAAGCCATG  GTTTTCTTCTGGAGGCAAAAAATTAAACCAAC
```

- IL2RG–CXorf65, -X:70244852..70240979 (distance: 3872 nt)
  Donor exon (score:0.997259):NM_000206.exon7/8
  Acceptor exon (score:0.997573): NM_001025265.exon5/6
  Translation: ptc
  5′ gene domains: IL6Ra-bind 3.9e-12
  3′ gene domains:
  TIC domains: IL6Ra-bind 3.2e-12

```
UHR  CTGAATACCACGGGAACTTTTCG  TTGCACTGCGCATACAATGTGATGCCC
```

- GPC2–GAL3ST4, -7:99606820..99602842 (distance: 3977 nt)
  Donor exon (score:0.994955):NM_152742.exon9/10
  Acceptor exon (score:0.999482): NM_024637.exon2/4
  Translation: ptc
  5′ gene domains: Glypican 1.3e-180
  3′ gene domains: Gal-3-0_sulfotr 9.7e-56
  TIC domains: Glypican 1.3e-174
  Supporting ESTs: DC406323 (thymus uncharacterized histology)

```
UHR  ACACGACCTGGgCGGGCAGGACGCGG  ACGCTGACAGAGGCAAAAAATCTGC
```

- PPT2–EGFL8, +6:32238377..32242456 (distance: 4078 nt)
  Donor exon (score:0.996240):NM_138717.exon8/9, NM_005155.exon8/9
  Acceptor exon (score:0.984695): NM_030652.exon4/9
  Translation: ptc
  5′ gene domains: Palm_thioest 5.5e-17
  3′ gene domains: EMI 2.1e-13, EGF_CA 3.4e-07
  TIC domains: Palm_thioest 1e-13

```
UHR  GACCGTCCTGGAGATGGAGGAGCAACTG  GACCATGTACCGCGTTATGTGG
```

- DUS4L–BCAP29, +7:107004273..107008440 (distance: 4166 nt)
  Donor exon (score:0.998599):NM_181581.exon7/8
  Acceptor exon (score:0.998543): NM_001008405.exon2/9, NM_018844.exon2/8
  Translation: full,ptc
  5′ gene domains: Dus 1.8e-73
  3′ gene domains: Bap31 1.2e-61
  TIC domains: Dus 3e-63, Bap31 9.5e-61

```
 T3           CTGGGACAGATG  GTGTGAAGAAAAAAAATGACAC
 N3  GCGGATTACTGGGACAGATG  GTGTGAAGAAAAA
HBR            TGGGACAGATG  GTGTGAAGAAAAAAAATGACACTCCAATGGGCTGCAGTGG
```

- MAPK12–HDAC10, -22:49035753..49031461 (distance: 4291 nt)
  Donor exon (score:0.996614):NM_002969.exon11/12
  Acceptor exon (score:0.928336): NM_032019.exon2/20
  Translation: ptc
  5′ gene domains: Pkinase 1.3e-70, Pkinase_Tyr 1e-30
  3′ gene domains: Hist_deacetyl 1.9e-85
  TIC domains: Pkinase 1.2e-70, Pkinase_Tyr 9.3e-31

```
HBR  CTTTGACGACGTTGACCGCACACTGGATGAATGGAAGC  CCCCGAGTGCGA
HBR  CTTTGACGACGTTGACCGCACACTGGATGAATGGAAGC  CCCCGAGTGCGA
HBR          ACGTTGACCGCACACTGGATGAATGGAAGC  CCCCGAGTGCGAGATCGAGC
HBR          tCGTTGACCGCACACTGGATGAATGGAAGC  CCCCGAGTGCGAGATCGAGC
```

- MXD3–RAB24, -5:176667388..176662927 (distance: 4460 nt)
  Donor exon (score:0.999297):NM_031300.exon5/6, NM_001142935.exon5/6
  Acceptor exon (score:0.998062): NM_130781.exon2/9
  Translation: ptc
  5′ gene domains: HLH 2.7e-10
  3′ gene domains: Ras 2.4e-45, Miro 1.4e-18, Arf 6.1e-13
  TIC domains: HLH 2.2e-10
  AceView: LMAN2andMXD3andRAB24.vbApr07
  Supporting ESTs: BM922588 (uncharacterized tissue normal), BP330389 (gastrointestinal tract uncharacterized histology), BX451531 (brain normal)

```
 N2            CTCAGACTCAGACCAAG  GACCCTGACGTGGCTGAAGCGGCCCCGGGAGCA
HBR  CCTCTGAGCGCTCAGACTCAGACCAAG  GACCCTGACGTGGCTGAAGCGGC
```

- E2F1–NECAB3, -20:31728572..31723889 (distance: 4682 nt)
  Donor exon (score:0.996680):NM_005225.exon6/7
  Acceptor exon (score:0.999798): NM_031231.exon2/13, NM_031232.exon2/12
  Translation: ptc
  5′ gene domains: E2F_TDP 2.2e-21
  3′ gene domains: ABM 5.2e-10
  TIC domains: E2F_TDP 1.6e-21

```
UHR  TCACCACAGATCCCAGCCAGTCTCTACTCAGCCTGGAGCAAG  GTTTTCCG
UHR      CACAGATCCCAGCCAGTCTCTACTCAGCCTGGAGCAAG  GTTTTCCGCAGA
UHR            TCCCAGCCAGTCTCTACTCAGCCTGGAGCAAG  GTTTTCCGCAGAGCAGAC
```

- HSF4–NOL3, +16:65760505..65765566 (distance: 5060 nt)
  Donor exon (score:0.997831):NM_001040667.exon13/15, NM_001538.exon13/15
  Acceptor exon (score:0.999643): NM_003946.exon2/4
  Translation: tle
  5′ gene domains: HSF_DNA-bind 2.3e-33
  3′ gene domains: CARD 2.2e-18
  TIC domains: HSF_DNA-bind 3.7e-33

```
 T2  GAGCTGTCCTTG  CCCCGACAATGGGCAACGCGC
```

- FAM173A–HAGHL, +16:711942..717413 (distance: 5470 nt)
  Donor exon (score:0.998348):NM_023933.exon3/5
  Acceptor exon (score:0.856396): NM_207112.exon2/7
  Translation: ptc
  5′ gene domains:
  3′ gene domains: Lactamase_B 6.8e-27
  TIC domains:

```
UHR  TcTCGCCGCAAGGATCTCTGGAAG  CCCTTCCTAGGGTGTGGAGAGCGGGC
```

- VKORC1–PRSS53, -16:31012134..31006648 (distance: 5485 nt)
  Donor exon (score:0.997808):NM_024006.exon2/3
  Acceptor exon (score:0.999897): NM_001039503.exon2/11
  Translation: full
  5′ gene domains: VKOR 1.8e-37
  3′ gene domains: Trypsin 6.9e-43
  TIC domains: VKOR 7.9e-21, Trypsin 9.1e-43
  AceView: VKORC1andPOL3S.iApr07
  Supporting ESTs: DA711817 (uncharacterized tissue neoplasia), DB164391 (thymus uncharacterized histology)

```
UHR  GTTGCATCTTCTACACACTACAGCTATTGTTAG  GTCTTCAAGCCGCTCAG
UHR    TGCATCTTCTACACACTACAGCTATTGTTAG  GTCTTCAAGCCGCTCAGCG
UHR      CATCTTCTACACACTACAGCTATTGTTAG  GTCTTCAAGCCGCTCAGCGTG
```

- MED12–NLGN3, +X:70278539..70284125 (distance: 5585 nt)
  Donor exon (score:0.973441):NM_005120.exon44/45
  Acceptor exon (score:0.999782): NM_018977.exon2/7
  Translation: ptc
  5′ gene domains: Med12-LCEWAV 3.4e-213, Med12-PQL 1.8e-91, Med12 2.1e-15
  3′ gene domains: COesterase 2.4e-193
  TIC domains: Med12-LCEWAV 3.4e-213, Med12-PQL 1.8e-91, Med12 2.1e-15

```
UHR  GGTCCGGCAACTTCAACAACAGCTCTCTA  GCCTGTCTGGCCCTtAGGGAG
```

- RPL17–C18orf32, -18:45269727..45264139 (distance: 5587 nt)
  Donor exon (score:0.997888):NM_000985.exon6/7, NM_001035006.exon6/7
  Acceptor exon (score:0.990119): NM_001035005.exon2/3
  Translation: ptc
  5′ gene domains: Ribosomal_L22 4.9e-38
  3′ gene domains:
  TIC domains: Ribosomal_L22 9.5e-38
  AceView: RPL17andSNORD58AandU58.bApr07
  Supporting ESTs: BG197808 (uncharacterized tissue neoplasia), BX393839 (uncharacterized tissue neoplasia), AV716493 (uncharacterized tissue normal), BX393840 (uncharacterized tissue neoplasia), BP309158 (brain uncharacterized histology), BI461805 (testis normal), BI601271 (brain normal), CB216016 (uterus normal), BG106268 (lymphoreticular neoplasia), BG617881 (liver normal)

```
 T1                                  GAGGAGGTTGCCCAGAAGAAAAAG  TTGAGGAGC
 N1  CGGAAAAGGAACAGATTGTTCCTAAACCAGAAGAGGAGGTTGCCCAGAAGAAAAAG  TTGAGGAGCTCAAGCTTGG
 N2                   GTTCCTAAACCAGAAGAGGAGGTTGCCCAGAAGAAAAAG  TTGAGGAGCTC
 N2                           ACCAGAAGAGGAGGTTGCCCAGAAGAAAAAG  TTGAGGAGCTCAAGCTTGG
 N2                                     GAGGTTGCCCAGAAGAAAAAG  TTGAGGAGCTCAAGCTTGGGAAAATGGTG
HBR                       CTAAACCAGAAGAGGAGGTTGCCCAGAAGAAAAAG  TTGAGGAGCTCAAGC
HBR                                                 AAGAAAAAG  TTGAGGAGCTCAAGCTTGGGAAAATGGTGTGCATTCCTTGc
UHR                          AACCAGAAGAGGAGGTTGCCCAGAAGAAAAAG  TTGAGGAGCTCAAGCTTG
UHR                                        GTTGCCCAGAAGAAAAAG  TTGAGGAGCTCAAGCTTGGGAAAATGGTGTGC
```

- PSENEN–LIN37, +19:40929264..40934917 (distance: 5652 nt)
  Donor exon (score:0.994069):NM_172341.exon3/4
  Acceptor exon (score:0.997787): NM_019104.exon2/9
  Translation: ptc
  5′ gene domains: PEN-2 2.2e-43
  3′ gene domains:
  TIC domains: PEN-2 2.8e-22
  AceView: PSENENandF25965.cApr07

```
 N1  TACACAGAACAGAGCCAAATCAAAGGCT  AGCTGGAGATGGcCAAAGCCCG
```

- GALT–IL11RA, +9:34639561..34645215 (distance: 5653 nt)
  Donor exon (score:0.993243):NM_000155.exon10/11
  Acceptor exon (score:0.999746): NM_147162.exon2/11, NM_001142784.exon2/13, NM_004512.exon2/13
  Translation: full
  5′ gene domains: GalP_UDP_transf 2.3e-66, GalP_UDP_tr_C 3.1e-66
  3′ gene domains:
  TIC domains: GalP_UDP_transf 9.7e-66, GalP_UDP_tr_C 1.2e-60

```
 N2         CCTGAGCAG  ATGAGCAGCAGCTGCTCAGtGCTGAGCAGGGTCCTGGTGGC
UHR  CTCACCCCTGAGCAG  ATGAGCAGCAGCTGCTCAGGGCTGAGCAGGGTCCT
```

- ABHD14A–ACY1, +3:51987430..51993103 (distance: 5672 nt)
  Donor exon (score:0.998715):NM_015407.exon3/5
  Acceptor exon (score:0.995360): NM_000666.exon2/15
  Translation: ptc
  5′ gene domains: Hydrolase_4 7.6e-07
  3′ gene domains: Peptidase_M20 1.1e-34, M20_dimer 1.4e-13
  TIC domains: Hydrolase_4 0.008
  AceView: ABHD14AandACY1.qApr07
  Supporting ESTs: DA590982 (lung normal), DA965938 (stomach normal), BM545401 (cerebrum normal)

```
 T3  GCCGTGGCCCTTGACCTTCCAG  CTCACCACGCG
HBR            CTTGACCTTCCAG  CTCACCACGCGCAGCGCCATGACCAGCAAGGGTCCCG
```

- MLLT6–CISD3, +17:34134755..34140539 (distance: 5783 nt)
  Donor exon (score:0.995926):NM_005937.exon19/20
  Acceptor exon (score:0.998984): NM_001136498.exon2/4
  Translation: full
  5′ gene domains:
  3′ gene domains: zf-CDGSH 1.1e-14
  TIC domains: zf-CDGSH 2.4e-13

```
 N1  GACCAACCCCTTCCTCAGCCTGTCGGGAGCAGAGGGCAGTGGCGGTGGCCCCAAAGGAGGG  GACCTGAACCCGCG
 N3                                               GGTGGCCCCAAAGGAGGG  GACCTGAACCCGCGG
```

- TUSC2–HYAL2, -3:50338792..50332970 (distance: 5821 nt)
  Donor exon (score:0.999069):NM_007275.exon2/3
  Acceptor exon (score:0.996349): NM_033158.exon2/4, NM_003773.exon2/4
  Translation: ptc
  5′ gene domains:
  3′ gene domains: Glyco_hydro_56 1e-144
  TIC domains:
  AceView: TUSC2andHYAL2.iApr07
  Supporting ESTs: DA988477 (synovium uncharacterized histology)

```
HBR  CGAGTGCATAAGAATCTGATTCCTCAG  TTCCTGAGCTGGTGCCAGGCAGG
```

- ADCK4–NUMBL, -19:45890696..45884740 (distance: 5955 nt)
  Donor exon (score:0.983186):NM_024876.exon14/15, NM_001142555.exon13/14
  Acceptor exon (score:0.988904): NM_004756.exon2/10
  Translation: full
  5′ gene domains: ABC1 3.1e-32
  3′ gene domains: NumbF 2.1e-40, PID 6.9e-32, PTB 8.9e-07
  TIC domains: ABC1 8.8e-32, NumbF 4.2e-40, PID 1.5e-31, PTB 1.8e-06

```
 T3      CAGGCTTTGAAACCAAG  GGCGGACCCCGGAGGC
 N3  CTCACAGGCTTTGAAACCAAG  GGCGGACCCCGG
 N3  CTCACAGGCTTTGAAACCAAG  GGCGGACCCCGG
HBR         GCTTTGAAACCAAG  GGCGGACCCCGGAGGCCTGAGCGGCACCTGCCCCCc
```

- PMF1–BGLAP, +1:154472898..154478968 (distance: 6069 nt)
  Donor exon (score:0.997970):NM_007221.exon4/5
  Acceptor exon (score:0.999659): NM_199173.exon2/4
  Translation: ptc
  5′ gene domains: Nnf1 2.7e-27
  3′ gene domains: Gla 3.5e-17
  TIC domains: Nnf1 2.9e-27
  AceView: PMF1andBGLAP.bApr07

Supporting ESTs: CD388852 (embryonic tissue normal), AI131030 (heart normal), BG575884 (mammary gland neoplasia), BI828617 (brain normal), BG752226 (eye normal), BQ774541 (bone neoplasia), CD049192 (embryonic tissue normal), BG752923 (eye normal), BG401176 (kidney normal), CA503394 (lung normal), BF309795 (muscle neoplasia), BF968865 (adrenal cortex neoplasia), BF525767 (brain neoplasia), BM971540 (lung normal), AA464650 (ovary neoplasia), BG536872 (lung normal), BI917653 (brain normal), BM738565 (stomach neoplasia), BF792670 (adrenal cortex neoplasia)

```
 T1                            GGCCCAGCAGCAGGCCTGGCAG  GTGCGgAGCCC
 T3                           AGGCCCAGCAGCAGGCCTGGCAG  GTGCGAAGCC
 T3                            GGCCCAGCAGCAGGCCTGGCAG  GTGCGAAGCCC
 T3                              CCCAGCAGCAGGCCTGGCAG  GTGCGAAGCCCAG
 T3                                   CAGCAGGCCTGGCAG  GTGCGAAGCCCAGCGGTG
 T3                                        GGCCTGGCAG  GTGCGAAGCCCAGCGGTGCAGAG
 N1  GGTGGAGGAGCTGCAGCTACAGGTCCAGGCCCAGCAGCAGGCCTGGCAG  GTGCGAAGCCCAGCGGTGCAGAGTCC
 N1     TGGAGGAGCTGCAGCTACAGGTCCAGGCCCAGCAGCAGGCCTGGCAG  GTGCGAAGCCCAGCGGTGCAGAGTCCAG
 N1         GAGCTGCAGCTACAGGTCCAGGCCCAGCAGCAGGCCTGGCAG  GTGCGAAGCCCAGCGGTGCAGAGTCCAGCAAAG
 N1             TGCAGCTACAGGTCCAGGCCCAGCAGCAGGCCTGGCAG  GTGCGAAGCCCAGCGGTGCAGAGTCCAGCcAAGGTGC
 N1              GCAGCTACAGGgCCAGGCCCAGCAGCAGGCCTGGCAG  GTGCGAAGCCCAGCGGTGCAGAGTCCAGCAAAGGTGCA
 N1                          CAGGCCCAGCAGCAGGCCTGGCAG  GTGCaAAGCCCAGCGGTGCAGAGTCC
 N1                            GGCCCAGCAGCAGGCCTGGCAG  GTGCGAAGCCCAGCGGTGCAGAGTCCAG
 N1                                 AGCAGCAGGCCTGGCAG  GTGCGAAGCCCAGCGGTGCAGAGTCCAGCAAAG
 N1                                 AGCAGCAGGCCTGGCAG  GTGCGAAGCCCAGCGGTGCAGAGTCCAGCAAAG
 N2                        TCCAGGCCCAGCAGCAGGCCTGGCAG  GTGCGAAGCCCAGCGGTGCAGAGT
 N2                            GGCCCAGCAGCAGGCCTGGCAG  GTGCGAAGCCCAGCGGTGCAGAGTCCAG
 N2                                CAGCAGCAGGCCTGGCAG  GTGCGAAGCCCAGCGGTGCAGAGTCCAGCAAA
 N2                                CAGCAGCAGGCCTGGCAG  GTGCGAAGCCCAGCGGTGCAGAGTCCAGCAAA
 N2                                   CAGCAGGCCTGGCAG  GTGCGAAGCCCAGCGGTGCAGAGTCCAGCAAAGGT
 N2                                   CAGCAGGCCTGGCAG  GTGCGAAGCCCAGCGGTGCAGAGTCCAGCcAAGGT
 N2                                      CAGGCCTGGCAG  GTGCGAAGCCCAGCGGTGCAGAGTCCAGCAAAGGTGCA
 N3                              CCCAGCAGCAGGCCTGGCAG  GTGCGAAGCCCAG
HBR         GAGCTGCAGCTACAGGTCCAGGCCCAGCAGCAGGCCTGGCAG  GTGCGAAG
HBR          AGCTGCAGCTACAGGTCCAGGCCCAGCAGCAGGCCTGGCAG  GTGCGAAGC
HBR           GCTGCAGCTACAGGTCCAGGCCCAGCAGCAGGCCTGGCAG  GTGCGAAGCC
HBR            CTGCAGCTACAGGTCCAGGCCCAGCAGCAGGCCTGGCAG  GTGCGAAGCCC
HBR               CAGCTACAGGTCCAGGCCCAGCAGCAGGCCTGGCAG  GTGCGAAGCCCAGC
HBR               CAGCTACAGGTCCAGGCCCAGCAGCAGGCCTGGCAG  GTGCGAAGCCCAGC
HBR                   TACAGGTCCAGGCCCAGCAGCAGGCCTGGCAG  GTGCGAAGCCCAGCGGTG
HBR                    ACAGGTCCAGGCCCAGCAGCAGGCCTGGCAG  GTGCGAAGCCCAGCGGTGC
HBR                       GGTCCAGGCCCAGCAGCAGGCCTGGCAG  GTGCGAAGCCCAGCGGTGCAGA
HBR                         TCCAGGCCCAGCAGCAGGCCTGGCAG  GTGCGAAGCCCAGCGGTGCAGAGT
HBR                           CAGGCCCAGCAGCAGGCCTGGCAG  GTGCGAAGCCCAGCGGTGCAGAGTCC
HBR                             GGCCCAGCAGCAGGCCTGGCAG  GTGCGAAGCCCAGCGGTGCAGAGTCCAG
HBR                             GGCCCAGCAGCAGGCCTGGCAG  GTGCGAAGCCCAGCGGTGCAGAGTCCAG
HBR                              GCCCAGCAGCAGGCCTGGCAG  GTGCGAAGCCCAGCGGTGCAGAGTCCAGC
HBR                              GCCCAGCAGCAGGCCTGGCAG  GTGCGAAGCCCAGCtGTGCAGAGTCCAGC
HBR                                   CAGCAGGCCTGGCAG  GTGCGAAGCCCAGCGGTGCAGAGTCCAGCAAAGGT
HBR                                      CAGGCCTGGCAG  GTGCGAAGCCCAGCGGTGCAGAGTaCAGCAAAGGTGCA
HBR                                       AGGCCTGGCAG  GTGCGAAGCCCAGCGGTGCAGAGTCCAGCAAAGGTGCAG
UHR             TGCAGCTACAGGTCCAGGCCCAGCAGCAGGCCTGGCAG  GTGCGAAGCCCc
UHR             TGCAGCTACAGGTCCAGGCCCAGCAGCAGGCCTGGCAG  GTGCGAAGCCCA
UHR              GCAGCTACAGGTCCAGGCCCAGCAGCAGGCCTGGCAG  GTGCGAAGCCCAG
UHR                    ACAGGTCCAGGCCCAGCAGCAGGCCTGGCAG  GTGCGAAGCCCAGCGGTGC
UHR                    gCAGGTCCAGGCCCAGCAGCAGGCCTGGCAG  GTGCGAAGCCCAGCGGTGC
UHR                              GCCCAGCAGCAGGCCTGGCAG  GTGCGAAGCCCAGCGGTGCAGAGTCCAGC
UHR                              GCCCAGCAGCAGGCCTGGCAG  GTGCGAAGCCCAGCGGTGCAGAGTCCAGC
UHR                              GCCCAGCgGCAGGCCTGGCAG  GTGCGAAGCCCAGCGGTGCAGAGTCCAGC
UHR                                CAGCAGCAGGCCTGGCAG  GTGCGAAGCCCAGCGGTGCAGAGTCCAGCAAA
UHR                                 AGCAGCAGGCCTGGCAG  GTGCGAAGCCCAGCGGTGCAGAGTCCAGCAAAG
UHR                                 AGCAGCAGGCCTGGCAG  GTGCGAAGCCCAGCGGTGCAGAGTCCAGCAAAG
UHR                                 AGCAGCAGGCCTGGCAG  GTGCGAAGCCCAGCGGTGCAGAGTCCAGCAAAG
UHR                                     GCAGGCCTGGCAG  GTGCGAAGCCCAGCGGTGCAGAGTCCAGCAAAGGTGC
UHR                                      CAGGCCTGGCAG  GTGCGAAGCCCAGCGGTGCAGAGTCCAGCAAAGGTGCA
```

- TMEM219–TAOK2, +16:29890400..29896560 (distance: 6159 nt)
  Donor exon (score:0.999069):NM_001083613.exon5/6, NM_194280.exon5/6
  Acceptor exon (score:0.997140): NM_016151.exon2/16, NM_004783.exon2/19

Translation: ptc
5′ gene domains:
3′ gene domains: Pkinase 8.2e-62, Pkinase_Tyr 6.9e-44
TIC domains:

```
 T3                         CCGACTTGTTTCTCAG  GCCAGGCCCCACTCTCA
 N1  CTGAGGGCACTGGCCTAGTTCCCGACTTGTTTCTCAG  GCCAGGCCCCACT
```

- FBXL19–ORAI3, +16:30865770..30872007 (distance: 6236 nt)
  Donor exon (score:0.997591):NM_001099784.exon10/11
  Acceptor exon (score:0.999921): NM_152288.exon2/2
  Translation: tle
  5′ gene domains: zf-CXXC 7.1e-14, F-box 6.1e-09
  3′ gene domains: Orai-1 6.2e-113
  TIC domains: zf-CXXC 6.5e-14, F-box 5.6e-09

```
 N1  GTGCACCTCAATCTTGCTG  GTGGCCATGGTGGAGGTGCAGCTGGAGAGTGACCACGAGTACCCACCAGGCCTGCT
```

- CAMK2G–NDST2, -10:75244767..75238493 (distance: 6273 nt)
  Donor exon (score:0.951403):NM_172173.exon19/20, NM_001222.exon18/19, NM_172170.exon19/20, NM_172171.exon20/21, NM_172169.exon20/21
  Acceptor exon (score:0.999820): NM_003635.exon3/15
  Translation: ptc
  5′ gene domains: Pkinase 3.5e-74, CaMKII_AD 1.8e-60, Pkinase_Tyr 7.7e-35
  3′ gene domains: HSNSD 2.2e-249, Sulfotransfer_1 5e-45
  TIC domains: Pkinase 3.5e-74, CaMKII_AD 1.8e-60, Pkinase_Tyr 7.7e-35

```
HBR  CTGCCGCACCGCTGCAGTGAGCTCAGCCACAG  GTAGAGGGGAAGAGATTG
```

- COPE–LASS1—GDF1, -19:18871682..18865384 (distance: 6297 nt)
  Donor exon (score:0.999297):NM_199444.exon8/9, NM_007263.exon9/10, NM_199442.exon8/9
  Acceptor exon (score:0.999261): NM_021267.exon2/8, NM_198207.exon2/6, NM_001492.exon2/8
  Translation: full,ptc
  5′ gene domains: Coatomer_E 4.8e-76
  3′ gene domains: TRAM_LAG1_CLN8 2e-34, TGF_beta 1.2e-36, TGFb_propeptide 1.9e-11
  TIC domains: Coatomer_E 2.8e-69, TRAM_LAG1_CLN8 5.6e-34

```
HBR  AGGTCCCATCCCTTCATCAAGGAGTACCAGGCCAAG  CCCCTGGCGAAGCG
```

- ARPC4–TTLL3, +3:9820697..9827305 (distance: 6607 nt)
  Donor exon (score:0.995526):NM_005718.exon5/6, NM_001024959.exon5/6, NM_001024960.exon5/6
  Acceptor exon (score:0.998873): NM_001025930.exon2/13
  Translation: ptc
  5′ gene domains: ARPC4 4.3e-37
  3′ gene domains: TTL 4.5e-84
  TIC domains: ARPC4 1.3e-36
  AceView: ARPC4andTTLL3.vtApr07
  Supporting ESTs: DB154746 (thymus uncharacterized histology), AL601968 (uncharacterized tissue uncharacterized histology), DB161115 (thymus uncharacterized histology), DB148847 (thymus uncharacterized histology), DB040549 (testis normal), DA863779 (placenta normal), DA435984 (uncharacterized tissue neoplasia), DB090955 (testis uncharacterized histology), DB114925 (thymus uncharacterized histology), DB162954 (thymus uncharacterized histology), DB235378 (uncharacterized tissue uncharacterized histology), BM549815 (uncharacterized tissue normal), DA861814 (placenta normal), DA339988 (cerebrum normal), DB097496 (testis uncharacterized histology), DA675719 (uncharacterized tissue normal), DB228540 (uncharacterized tissue uncharacterized histology)

```
 N1       CGTGCCCGCATTGTGGCTGAAGAGTTCCTTAAGAAT  GTTTCCCGGTCCTCTGGCGAGGATCCTCCtAGGCGTCTC
 N2                      GCTGAAGAGTTCCTTAAGAAT  GgTTCCCGGTCCTCTGGCGAGGATCCTCC
UHR  ATGCCCGTGCCCGCATTGTGGCTGAAGAGTTCCTTAAGAAT  GTTTCCCGG
UHR  ATGCCCGTGCCCGCATTGTGGCTGAAGAGTTCCTTAAGAAT  GTTTCCCGG
UHR        GTGCCCGCATTGTGGCTGAAGAGTTCCTTAAGAAT  GTTTCCCGGTCCTCT
UHR                      GCTGAAGAGTTCCTTAAGAAT  GTTTCCCGGTCCTCTGGCGAGGATCCTCC
UHR                         GAAGAGTTCCTTAAGAAT  GTTTCCCGGTCCTCTGGCGAGGATCCTCCAAG
UHR                         GAAGAGTTCCTTAAGAAT  GTTTCCCGGTCCTCTGGCGAGGATCCTCCAAG
UHR                         GAAGAGTTCCTTAAGAAT  GTTTCCCGGTCCTCTGGCGAGGATCCTCCcAG
UHR                                  CTTAAGAAT  GTTTCCCGGTCCTCTGGCGAGGATCCTCCAAGGCGTCTCAC
```

- PPM1J–RHOC, -1:113054605..113047951 (distance: 6653 nt)
  Donor exon (score:0.998583):NM_005167.exon9/10
  Acceptor exon (score:0.994276): NM_175744.exon3/6, NM_001042679.exon3/6, NM_001042678.exon2/5

Translation: full
5′ gene domains: PP2C 9.6e-13
3′ gene domains: Ras 6.9e-58, Miro 1.5e-16, Arf 8e-10
TIC domains: PP2C 1.4e-12, Ras 1.6e-56, Miro 1.8e-15, Arf 1.5e-08
AceView: PPM1JandRHOC.oApr07
Supporting ESTs: BI459837 (testis normal)

```
HBR  TGACCACAGCAG  CCCCACCATGGCTGCAATCCGAAAGAAGCTGGTGATCG
```

- TMEM141–KIAA1984, +9:138806631..138813375 (distance: 6743 nt)
  Donor exon (score:0.998325):NM_032928.exon4/5
  Acceptor exon (score:0.999463): NM_001039374.exon2/14
  Translation: full
  5′ gene domains:
  3′ gene domains:
  TIC domains:
  AceView: TMEM141andKIAA1984andC9orf86.mApr07
  Supporting ESTs: BG333348 (lung neoplasia)

```
 N1  GAGAAATGCAACAACCTCTGGCTCTTCCTGGAGACCGGGCAGCTCCCCAAAGACAGGAGCACAG  AGCAGTGTCGG
HBR                                     CGGGCAGCTCCCCAAAGACAGGAGCACAG  AGCAGTGTCGGGCACTCCAGA
HBR                                         CAGCTCCCCAAAGACAGGAGCACAG  AGCAGTGTCGGGCACTCCAGATCCA
HBR                                                         GGAGCACAG  AGCAGTGTCGGGCACTCCAGATCCAAGGGGaGAAAGAGAAT
```

- MED26–SLC35E1, -19:16550146..16543393 (distance: 6752 nt)
  Donor exon (score:0.999046):NM_004831.exon2/3
  Acceptor exon (score:0.999112): NM_024881.exon2/6
  Translation: ptc
  5′ gene domains: Med26 1.4e-13
  3′ gene domains: TPT 1e-35
  TIC domains:

```
 T3  CAAAGAGGCACTTGAG  TCAAGGCCACCATGCCC
```

- UQCRQ–LEAP2, +5:132230626..132237541 (distance: 6914 nt)
  Donor exon (score:0.997047):NM_014402.exon2/3
  Acceptor exon (score:0.998744): NM_052971.exon2/3
  Translation: ptc
  5′ gene domains: UcrQ 9.1e-35
  3′ gene domains: LEAP-2 4.4e-51
  TIC domains: UcrQ 3.8e-19

```
 N1  CCGCATTCGGGAGTCTTTCTTTCGCGTGGTGCCGC  ATAGATGGCTCCCCA
```

- SLC39A1–CRTC2, -1:152201320..152194266 (distance: 7053 nt)
  Donor exon (score:0.998558):NM_014437.exon4/5
  Acceptor exon (score:0.985007): NM_181715.exon2/14
  Translation: full
  5′ gene domains: Zip 7.1e-60
  3′ gene domains:
  TIC domains: Zip 4e-12

```
 T3                 TGGCAGCCTTGCACGTGACG  TTACAGGCCCAAA
 N2  CCATAGATGAGGCCCTGGCAGCCTTGCACGTGACG  TTACAGGCCCAAAAA
UHR     TAGATGAGGCCCTGGCAGCCTTGCACGTGACG  TTACAGGCCCAAAAACTG
UHR         TGAGGCCCTGGCAGCCTTGCACGTGACG  TTACAGGCCCAAAAACTGCGAC
UHR                 TGGCAGCCTTGCACGTGACG  TTACAGGCCCAcAAACTGCGACTGGCATAC
```

- SIDT2–TAGLN, +11:116571841..116578928 (distance: 7086 nt)
  Donor exon (score:0.997591):NM_001040455.exon25/26
  Acceptor exon (score:0.976550): NM_001001522.exon2/5, NM_003186.exon2/5
  Translation: full
  5′ gene domains:
  3′ gene domains: CH 5.1e-12, Calponin 5e-12
  TIC domains: Calponin 3.7e-11, CH 1.2e-10
  Supporting ESTs: T05025 (brain normal)

```
 N1     CACTTCCTCTCCTCCATCGCCATGTTCGGGTCCTTCCTG  CTTTCCCCAGACATGGCCAACAAGGGTCCTTCCTAT
 N1           CTCTCCTCCATCGCCATGTTCGGGTCCTTCCTG  CTTTCCCCAGACATGGC
```

```
 N1                   TCCATCGCCATGTTCGGGTCCTTCCTG  CTTTCCCCAGACATGGCCAACAAGGGTCCTTCCTATGGCATGAGCCGC
 N1                                      CCTTCCTG  CTTTCCCCAGACATGGCCAACAAGGGTCCTTCCTATGGCATGAGCCGCGAAGTGCAGTCCAAAATCG
HBR           CTCTCCTCCATCGCCATGTTCGGGTCCTTCCTG  CTTTCCCCAGACATGGC
HBR                            GTTCGGGTCCTTCCTG  CTTTCCCCAGACATGGCCAACAAGGGgCCTTCCT
HBR                               CGGGTCCTTCCTG  CTTTCCCCAGACATGGCCAACAAGGGTCCTTCCTATG
UHR  TGGCACTTCCTCTCCTCCATCGCCATGTTCGGGTCCTTCCTG  CTTTCCCC
UHR     CACTTCCTCTCCTCCATCGCCATGTTCGGGTCCTTCCTG  CTTTCCCCAGA
UHR           CTCTCCTCCATCGCCATGTTCGGGTCCTTCCTG  CTTTCCCCAGACATGGC
UHR           CTCTCCcCCATCGCCATGTTCGGGTCCTTCCTG  CTTTCCCCAGACATGGC
```

- MMP25–IL32, +16:3048671..3055786 (distance: 7114 nt)
  Donor exon (score:0.991783):NM_022468.exon9/10
  Acceptor exon (score:0.990585): NM_001012633.exon2/8, NM_001012634.exon2/6, NM_001012635.exon2/6, NM_004221.exon2/7, NM_001012632.exon2/7, NM_001012631.exon3/8
  Translation: ptc
  5′ gene domains: Peptidase_M10 6.3e-47, Hemopexin 4.7e-15, PG_binding_1 2.5e-10
  3′ gene domains:
  TIC domains: Peptidase_M10 4.8e-47, Hemopexin 4e-15, PG_binding_1 2.1e-10

```
 N1  CGCGACCTGAGCCTCTGGGAAGGCGCGCCCCCCTCCCCTGACGATGTCACCGTCAGCAACGCAG  GCCTTGGCTCC
 N1                                 CCTGACGATGTCACCGTCAGCAACGCAG  GCCTTGGCTCCTTGAACTTTTG
 N1                                                   AGCAACGCAG  GCCTTGGCTCCTTGAACTTTTGGCCGCCATGTGCTTCCCG
```

- DTX2–UPK3B, +7:75971698..75978910 (distance: 7211 nt)
  Donor exon (score:0.999297):NM_020892.exon11/12, NM_001102595.exon9/10, NM_001102594.exon10/11, NM_001102596.exon7/8
  Acceptor exon (score:0.999647): NM_030570.exon2/4, NM_182684.exon3/6
  Translation: ptc
  5′ gene domains: WWE 3.4e-15
  3′ gene domains:
  TIC domains: WWE 2.9e-15
  Supporting ESTs: AA161025 (pancreas neoplasia)

```
 T2                            GGGCCGCAAG  CCTCCAGGGGCTTaCAGAACCCG
 N1           TcCCTTCCAGACAACGCCCAGGGCCGCAAG  CCTCCAGGGGCTTCCAGAACCCGGAGACACTGGCTGACATTCCGG
 N1            ACCTcCCAGACAACGCCCAGGGCCGCAAG  CCTCCAGGGGCTTCCAGAACCCGGAGACACTGGCTGACATTCCGGC
 N2  CCGCCAGTGCTACCTTCCAGACAACGCCCAGGGCCGCAAG  CCTCCAGGGG
 N2                TTCCAGACAACGCCCAGGGCCGCAAG  CCTCCAGGGGCTTCCAGAACCCGG
 N2                           CAGGGCCGCAAG  CCTCCAGGGGCTTCCAGAACCCGGAGACACTGGCTGAC
UHR                TTCCAGACAACGCCCAGGGCCGCAAG  CCTCCAGGGGCTTCCAGAACCCGG
UHR                          CCCAGGGCCGCAAG  CCTCCAGGGGCTTCCAGAACCCGGAGACACTGGCTG
```

- UBXN1–C11orf48, -11:62201566..62193851 (distance: 7714 nt)
  Donor exon (score:0.998107):NM_015853.exon7/8
  Acceptor exon (score:0.999731): NM_024099.exon3/7
  Translation: full
  5′ gene domains: UBX 2.6e-19, UBA 1.3e-08
  3′ gene domains:
  TIC domains: UBA 2e-08

```
UHR  CCAAGCGGGAGTATGACCAGTGTCGCATACAG  GATTTCTCTCAAAAGTCC
```

- MAGIX–PLP2, +X:48908643..48916420 (distance: 7776 nt)
  Donor exon (score:0.951736):NM_001099681.exon4/5, NM_001099680.exon3/4, NM_001099682.exon4/5, NM_024859.exon5/6
  Acceptor exon (score:0.998993): NM_002668.exon2/5
  Translation: ptc
  5′ gene domains: PDZ 2.5e-13
  3′ gene domains: MARVEL 1.6e-12
  TIC domains: PDZ 1.5e-13

```
 N3  CCGAAAAGGAGTTG  ATATTATGCCTGGTGATCC
```

- VPS72–TMOD4, -1:149417101..149409203 (distance: 7897 nt)
  Donor exon (score:0.998363):NM_005997.exon4/5
  Acceptor exon (score:0.999601): NM_013353.exon10/10
  Translation: full
  5′ gene domains: YL1 4.3e-55, YL1_C 3.3e-12
  3′ gene domains: Tropomodulin 2.3e-67
  TIC domains: YL1 2e-44
  AceView: VPS72andTMOD4.jApr07
  Supporting ESTs: AI763406 (uncharacterized tissue neoplasia)

```
 T1                              CGTTGACATAGAAGG  GTCGCCAGCAAAAGAAGA
 T3                           AaAACGTTGACATAGAAGG  GTCGCCAGCAAAAG
 T3                              CGTTGACATAGAAGG  GTCGCCAGCAAAAGAAGA
 T3                              CGTTGACATAGAAGG  GTCGCCAGCAAAAGAAGA
 T3                               GTTGACATAGAAGG  GTCGCCAGCAAAAGAAGAG
UHR  TGGGGAGCCAGGCCCCAAGGAAGAGAACGTTGACATAGAAGG  GTCGCCAG
```

- GPI–PDCD2L, +19:39579402..39587394 (distance: 7991 nt)
  Donor exon (score:0.999341):NM_000175.exon14/18
  Acceptor exon (score:0.999731): NM_032346.exon2/7
  Translation: full
  5′ gene domains: PGI 6.9e-245
  3′ gene domains: PDCD2_C 4.4e-51
  TIC domains: PGI 6.3e-187, PDCD2_C 2e-50
  AceView: GPIandPDCD2L.gApr07
  Supporting ESTs: BF983334 (gastrointestinal tract neoplasia)

```
UHR  CCCAGCACCCCATACGGAAGGGTCTGCATCACAAG  GATGCTCTGCCCACC
```

- HNRNPUL2–BSCL2, -11:62239548..62231372 (distance: 8175 nt)
  Donor exon (score:0.995433):NM_001079559.exon13/14
  Acceptor exon (score:0.998524): NM_001130702.exon2/12
  Translation: ptc
  5′ gene domains: SPRY 1.7e-21, SAP 3.2e-08
  3′ gene domains: Seipin 1.2e-64
  TIC domains: SPRY 1.6e-21, SAP 3.2e-08
  AceView: HNRPUL2andBSCL2.bApr07
  Supporting ESTs: CN346952 (embryonic tissue normal)

```
 T2                  ACAATCGGGAT  GGACCCTCCATTTTCCATATCC
 N1                  ACAATCGGGAT  GGACCCTCCATTTTCCATATnCAGGAAAATGTGATGCGC
UHR  GACTATTACAGACAATACAATCGGGAT  GGACCCTCCATTTTCCATATCCA
```

- TRIM3–HPX, -11:6427112..6418536 (distance: 8575 nt)
  Donor exon (score:0.999388):NM_006458.exon12/13, NM_033278.exon11/12
  Acceptor exon (score:0.999721): NM_000613.exon2/10
  Translation: ptc
  5′ gene domains: NHL 6.4e-10, Filamin 7.1e-20, zf-B_box 1.6e-10
  3′ gene domains: Hemopexin 2.4e-12
  TIC domains: NHL 2.5e-08, Filamin 6.5e-20, zf-B_box 1.5e-10

```
HBR  GCAACAGCCGCATCCAG  GACTAGTGCCCATGGGAATGTTGCTGAAGGCGA
HBR   CAACAGCCGCATCCAG  GACTAGTGCCCATGGGAATGTTGCTGAAGGCGAG
```

- ABHD14A–ACY1, +3:51984260..51993103 (distance: 8842 nt)
  Donor exon (score:0.999476):NM_015407.exon1/5
  Acceptor exon (score:0.995360): NM_000666.exon2/15
  Translation: full
  5′ gene domains: Hydrolase_4 7.6e-07
  3′ gene domains: Peptidase_M20 1.1e-34, M20_dimer 1.4e-13
  TIC domains: Peptidase_M20 1.4e-34, M20_dimer 1.6e-13
  AceView: ABHD14AandACY1.vcApr07
  Supporting ESTs: CF552723 (muscle normal), DA123957 (cerebellum normal)

```
 N2  CGCCTGGGCGGGGCCCGCCCGCTCATCCCGTTGGGCCCG  gTCACCACGCG
HBR          CGGGGCCCGCCCGCTCATCCCGTTGGGCCCG  CTCACCACGCGCAGCGCCA
```

- VPS11–HMBS, +11:118455220..118464175 (distance: 8954 nt)
  Donor exon (score:0.997485):NM_021729.exon13/15
  Acceptor exon (score:0.995749): NM_000190.exon2/14, NM_001024382.exon2/14
  Translation: ptc
  5′ gene domains: VPS11_C 3.2e-19, Clathrin 5.1e-13
  3′ gene domains: Porphobil_deam 5.3e-90, Porphobil_deamC 4.2e-19
  TIC domains: Clathrin 4e-13

```
HBR  ATCCGCCAGGAGATCCAAGAGCTCAAGGCCAG  GAAGAAAACAGCCCAAAG
```

- FAM117A–SLC35B1, -17:45148526..45139429 (distance: 9096 nt)
  Donor exon (score:0.998413):NM_030802.exon7/8

Acceptor exon (score:0.997093): NM_005827.exon2/9
Translation: full
5′ gene domains:
3′ gene domains: UAA 3.9e-86, EamA 8e-10, TPT 3.6e-12
TIC domains: UAA 6.3e-78, EamA 2.8e-09, TPT 2.1e-11

```
 T3  GgGCGTGTGTTTGAAGAAGCCAC  AACAAGAGGA
 T3  GTGCGTGTGTTTGAAaAAGCCAC  AACAAGAGGA
UHR      GTGTGTTTGAAGAAGCCAC  AACAAGAGGAAAGTATGGGGAAGGAGCCAAG
```

- GUK1–GJC2, +1:226402780..226412064 (distance: 9283 nt)
  Donor exon (score:0.987623):NM_000858.exon8/9
  Acceptor exon (score:0.998925): NM_020435.exon2/2
  Translation: tle
  5′ gene domains: Guanylate_kin 1.8e-71
  3′ gene domains: Connexin 5.9e-50, Connexin_CCC 4.7e-32
  TIC domains: Guanylate_kin 7e-71

```
UHR  GGCCTACGCAGAGCTGAAGGAGGCGCTCTCTGAG  GACCCGCCCGCCCGCC
```

- VMAC–CAPS, +19:5856092..5865396 (distance: 9303 nt)
  Donor exon (score:0.998471):NM_001017921.exon1/2
  Acceptor exon (score:0.999745): NM_004058.exon2/5, NM_080590.exon2/5
  Translation: full
  5′ gene domains:
  3′ gene domains:
  TIC domains:

```
 N1         GCCGCCCTAGACGAACTGGGTCGCGCCAAGGACCG  CTGCCCAGAGCCCAGACCAAGCATGGACGCCGTGGATGCC
 N1          CCGCCCTAGACGAACTGGGTCGCGCCAAGGACCG  CTGCCCAGAGCCCAGACCAAGCATGGACGCCGTGGATGCCA
 N2          CCGCCCTAGACGAACTGGGTCGCGCCAAGGACCG  CTGCCCAGAGCCCAGA
HBR  GCTGCGCGCCGCCCTAGACGAACTGGGTCGCGCCAAGGACCG  CTGCCCAG
HBR                         TGGGTCGCGCCAAGGACCG  CTGCCCAGAGCCCAGACCAAGCATGGACGCC
UHR           CGCCCTAGACGAACTGGGTCGCGCCAAGGACCG  CTGCCCAGAGCCCAGAC
```

- OBSL1–CHPF, -2:220124495..220115155 (distance: 9339 nt)
  Donor exon (score:0.998363):NM_015311.exon20/21
  Acceptor exon (score:0.999673): NM_024536.exon2/4
  Translation: ptc
  5′ gene domains: I-set 2e-17, ig 1e-06
  3′ gene domains: CHGN 1.6e-168
  TIC domains: I-set 2e-17, ig 1.1e-06
  Supporting ESTs: DC374009 (brain normal)

```
UHR  CACGGCTGCTGGTAGAGG  GACCCGCTACATCAtCACGGAGCTGGGCATCA
```

- NDUFB8–SEC31B, -10:102276146..102266744 (distance: 9401 nt)
  Donor exon (score:0.997977):NM_005004.exon4/5
  Acceptor exon (score:0.996365): NM_015490.exon2/26
  Translation: ptc
  5′ gene domains: NDUF_B8 2e-90
  3′ gene domains:
  TIC domains: NDUF_B8 4.8e-75
  AceView: NDUFB8andSEC31B.iApr07
  Supporting ESTs: BP320034 (placenta uncharacterized histology), DR000705 (brain normal)

```
 T3             CTACCAGCCTGTG  TGGGCTGAGGGCCATGTCAG
UHR  GTGTcCCCTGTCTACCAGCCTGTG  TGGGCTGAGGGCCATGTCAGTTTTCT
```

- PGLS–FAM129C, +19:17489659..17499078 (distance: 9418 nt)
  Donor exon (score:0.997943):NM_012088.exon4/5
  Acceptor exon (score:0.998012): NM_001098524.exon2/16, NM_173544.exon2/16
  Translation: full
  5′ gene domains:
  3′ gene domains:
  TIC domains:

```
UHR  GGCAACTGGAGAAGGCAAGGCAGCTGTTCTGAAG  GTGCCCCTGAGCCGAG
```

- MED22–SURF6, -9:135200801..135191258 (distance: 9542 nt)
  Donor exon (score:0.994343):NM_133640.exon4/5
  Acceptor exon (score:0.998513): NM_006753.exon2/5
  Translation: ptc
  5′ gene domains: Med22 1e-34
  3′ gene domains: SURF6 2e-51
  TIC domains: Med22 6.2e-35
  AceView: SURF5andSURF6.gApr07
  Supporting ESTs: BG480521 (placenta neoplasia)

```
HBR  TTGACCTCTACGAGCTGGAGGAGGAGTATTACTCGTCCAG  CTGGCAAAAC
HBR                        GAGTATTACTCGTCCAG  CTGGCAAAACTCAAGGCTCAGAAACTGCAGGGC
```

- FAM175A–HELQ, -4:84603671..84594122 (distance: 9548 nt)
  Donor exon (score:0.999075):NM_139076.exon8/9
  Acceptor exon (score:0.999449): NM_133636.exon2/18
  Translation: ptc
  5′ gene domains:
  3′ gene domains: DEAD 4.5e-14, Helicase_C 4.7e-08
  TIC domains:

```
 T1  CACAGATTCAGGCAGCAA  CCTAATGACAGTGAA
 T1   ACAGATTCAGGCAGCAA  CCTAATGACAGTGAAG
 T2  CACAGATTCAGGCAGCAA  CCTAATGACAGTGAA
 N3           CAGGCAGCAA  CCTAATGACAGTGgAGTGGACAT
```

- MAFG–SIRT7, -17:77478695..77469135 (distance: 9559 nt)
  Donor exon (score:0.997977):NM_002359.exon1/3
  Acceptor exon (score:0.999799): NM_016538.exon2/10
  Translation: tss
  5′ gene domains: bZIP_Maf 2.2e-42
  3′ gene domains: SIR2 5.1e-21
  TIC domains: SIR2 2e-18

```
 N2  CCGGCCTCGGCCCGCCGGGCCCGCAGTGTG  GTGTCGCGCATCCTGAGGAA
```

- PTGR2–ZNF410, +14:73418894..73428483 (distance: 9588 nt)
  Donor exon (score:0.999067):NM_152444.exon9/10
  Acceptor exon (score:0.999423): NM_021188.exon2/12
  Translation: ptc
  5′ gene domains: ADH_zinc_N 3.3e-15
  3′ gene domains:
  TIC domains: ADH_zinc_N 3.3e-15

```
 N3  GGAAAACATGGGAG  GTTACATTGATTACCCACC
```

- SCRN2–MRPL10, -17:43270635..43261035 (distance: 9599 nt)
  Donor exon (score:0.995853):NM_138355.exon7/8
  Acceptor exon (score:0.983544): NM_145255.exon2/5
  Translation: ptc
  5′ gene domains: Peptidase_C69 7.6e-09
  3′ gene domains: Ribosomal_L10 2.7e-07
  TIC domains: Peptidase_C69 6.3e-09

```
 N2  GGGGCTnATGGAGAGAGATCAG  GCCGGCTGCCTACCCTCCAGACTGTCCG
```

- TNFAIP8L2–SCNM1, +1:149395822..149405571 (distance: 9748 nt)
  Donor exon (score:0.999069):NM_024575.exon1/2
  Acceptor exon (score:0.999187): NM_024041.exon2/7
  Translation: tss
  5′ gene domains: DUF758 3.8e-87
  3′ gene domains:
  TIC domains:
  AceView: TNFAIP8L2andSCNM1.fApr07
  Supporting ESTs: BG531092 (testis neoplasia)

```
 T3                     CTGGCACCCAGCACAGCAG  AAAAGAAGAGTCGG
 N1  GGAACATCCAAGGCAAGACTGGCACCCAGCACAGCAG  AAAAGAAGAGTCG
 N1  GGAACATCCAAGGCAAGACTGGCACCCAGCACAGCAG  AAAAGAAGAGTCGGGGACCTCCTcGCCAGTTACATTCC
 N1                           CCCAGCACAGCAG  AAAAGAAGAGTCGGGGACCTCCTAGCCAGTTACATTC
HBR              GCAAGACTGGCACCCAGCACAGCAG  AAAAGAAGAGTCGGGGACCTCCTAG
```

- ZDHHC1–TPPP3, -16:65992361..65982521 (distance: 9839 nt)
  Donor exon (score:0.982837):NM_013304.exon4/11
  Acceptor exon (score:0.937064): NM_016140.exon3/5, NM_015964.exon2/4
  Translation: ptc
  5′ gene domains: zf-DHHC 1.4e-19
  3′ gene domains: p25-alpha 3.1e-58
  TIC domains:

```
UHR  ACTTGTGCAACGTGGATGT  GGTGGCATGGCAGCGAGCACAGACATGGCTG
```

- FAM119B–TSFM, +12:56453178..56463160 (distance: 9981 nt)
  Donor exon (score:0.993340):NM_206914.exon2/4, NM_015433.exon2/3
  Acceptor exon (score:0.998690): NM_005726.exon2/6
  Translation: ptc
  5′ gene domains: Methyltransf_16 9.7e-38
  3′ gene domains: EF_TS 1.6e-31
  TIC domains: Methyltransf_16 1.9e-13
  AceView: FAM119BandTSFM.mApr07
  Supporting ESTs: BF311717 (brain neoplasia)

```
 T3                        GGCAGCGCTGCAGG  GCTGGGTCTCTTCTGCGTC
 N1  CAGGCATCGTGGGGATCTTGGCAGCGCTGCAGG  GCTGGGTCTCTTCTGCGTCAGTCGCCCCcGCCAAGGCACACA
 N1                        GGCAGCGCTGCAGG  GCTGGGTCTCTTCTGCGTCAGTCGCCCCAGCCAAGG
 N1                         GCAGCGCTGCAGG  GCTGGGTCTCTTCTGCGTCAGTCGCCCCAGCCcAGGC
HBR      CATCGTGGGGATCTTGGCAGCGCTGCAGG  GCTGGGTCTCTTCTGCGTCAG
HBR                       TGGCAGCGCTGCAGG  GCTGGGTCTCTTCTGCGTCAGTCGCCCCAGCCAAG
HBR                         GCAGCGCTGCAGG  GCTGGGTCTCTTCTGCGTCAGTCGCCCCAGCCAAGGC
UHR                            GCGCTGCAGG  GCTGGGTCTCTTCTGCGTCAGTCGCCCCAGCCcAGGCACA
```

- RIPPLY2–CYB5R4, +6:84620599..84630613 (distance: 10013 nt)
  Donor exon (score:0.999388):NM_001009994.exon3/4
  Acceptor exon (score:0.999921): NM_016230.exon2/16
  Translation: ptc
  5′ gene domains:
  3′ gene domains: Cyt-b5 5.1e-24, FAD_binding_6 3.3e-20, NAD_binding_1 7.7e-16, CS 4.4e-11
  TIC domains:
  AceView: C6orf159andCYB5R4.gApr07
  Supporting ESTs: DA307205 (cerebrum normal)

```
HBR  GCTTTACCAATTCAGGCACCCAGTCAG  GTACCTTTAAAACAGGGCAGAAG
```

- SNCG–C10orf116, +10:88709865..88719937 (distance: 10071 nt)
  Donor exon (score:0.998710):NM_003087.exon3/5
  Acceptor exon (score:0.998139): NM_006829.exon2/3
  Translation: tle
  5′ gene domains: Synuclein 1.1e-59
  3′ gene domains:
  TIC domains: Synuclein 1.9e-56

```
HBR  GGCGGAGAACATCGCGGTCACCTCCGGGGTGGTGCGCAAG  TGTCAGCGGC
HBR   GCGGAGAACATCGCGGTCACCTCCGGGGTGGTGCGCAAG  TGTCAGCGGCC
HBR   GCGGAaAACATCGCGGTCACCTCCGGGGTGGTGCGCAAG  TGTCAGCGGCC
```

- PMS2–RSPH10B, -7:5983745..5973594 (distance: 10150 nt)
  Donor exon (score:0.999715):NM_000535.exon14/15
  Acceptor exon (score:0.995321): NM_173565.exon2/21
  Translation: ptc
  5′ gene domains: MutL_C 2.1e-25, DNA_mis_repair 1.8e-22, HATPase_c 1.4e-10
  3′ gene domains: MORN 5e-07
  TIC domains: MutL_C 2.6e-23, DNA_mis_repair 1.7e-22, HATPase_c 1.3e-10

```
UHR  AGATGTTTGCCTCCAGAGCCTGCCGGAAGTCG  AGAGGTGGCATCTCACcA
```

- TSC22D4–C7orf61, -7:99903111..99892544 (distance: 10566 nt)
  Donor exon (score:0.998599):NM_030935.exon4/5
  Acceptor exon (score:0.999683): NM_001004323.exon3/3
  Translation: full
  5′ gene domains: TSC22 2.6e-29
  3′ gene domains:
  TIC domains:

`HBR  CAACAAAATCGAGCAAGCCATG  TCATCAGTGAGTTCgGCCTCAGTCAGCT`

- CADM4–ZNF428, -19:48821070..48810426 (distance: 10643 nt)
  Donor exon (score:0.997965):NM_145296.exon7/9
  Acceptor exon (score:0.998388): NM_182498.exon2/3
  Translation: ptc
  5′ gene domains: I-set 3.1e-12, V-set 7.4e-07, C2-set_2 1.9e-11
  3′ gene domains:
  TIC domains: I-set 2.6e-12, V-set 6.2e-07, C2-set_2 1.6e-11

```
T3        GTGGTCTACG  CATCCCTCTCTACCTGCCAACAT
N3  TACTTGTGGTCTACG  CATCCCTCTCTACCTGCC
```

- FBXL19–ORAI3, +16:30861358..30872007 (distance: 10648 nt)
  Donor exon (score:0.876514):NM_001099784.exon9/11
  Acceptor exon (score:0.999921): NM_152288.exon2/2
  Translation: tle
  5′ gene domains: zf-CXXC 7.1e-14, F-box 6.1e-09
  3′ gene domains: Orai-1 6.2e-113
  TIC domains: zf-CXXC 5.6e-14, F-box 4.8e-09

`N2  GTTGCTGCTGCCTCCACCAGACACCAAACCAG  GTGGCCATGGTGGAGGTG`

- SLC2A11–MIF, +22:22556211..22566959 (distance: 10747 nt)
  Donor exon (score:0.999088):NM_001024939.exon10/12, NM_001024938.exon10/12, NM_030807.exon11/13
  Acceptor exon (score:0.998653): NM_002415.exon2/3
  Translation: ptc
  5′ gene domains: Sugar_tr 2.8e-90, MFS_1 4.4e-13
  3′ gene domains: MIF 8.2e-52
  TIC domains: Sugar_tr 5e-68, MFS_1 3.3e-15

`UHR  CcTCCTCAGCTTTGGCATTGGCCCTG  TACATCGCGGTGCACGTGGTCCCG`

- ZER1–ZDHHC12, -9:130535526..130524674 (distance: 10851 nt)
  Donor exon (score:0.999088):NM_006336.exon15/16
  Acceptor exon (score:0.993994): NM_032799.exon2/5
  Translation: ptc
  5′ gene domains:
  3′ gene domains: zf-DHHC 1e-22
  TIC domains:

`HBR  GACCAAGGAAATGGCCCG  AGCTGCGGCAATGGGAGGAGCAGGGGGAGCTG`

- ANKRD39–ANKRD23, -2:96882865..96871975 (distance: 10889 nt)
  Donor exon (score:0.995905):NM_016466.exon3/4
  Acceptor exon (score:0.999368): NM_144994.exon2/9
  Translation: full
  5′ gene domains: Ank 1.3e-09
  3′ gene domains: Ank 1.2e-09
  TIC domains: Ank 1.9e-09, Ank 1.9e-09
  AceView: ANKRD39andANKRD23.eApr07
  Supporting ESTs: CV806720 (embryonic tissue normal)

`HBR  CATGACCAGTCTGCATAAG  GTAAGTGGAGAAAGAGTTGAAGGGAAAGTGT`

- C1orf43–C1orf189, -1:152451420..152439688 (distance: 11731 nt)
  Donor exon (score:0.994703):NM_001098616.exon6/7, NM_138740.exon4/5, NM_015449.exon5/6
  Acceptor exon (score:0.897743): NM_001010979.exon3/4
  Translation: full
  5′ gene domains: NICE-3 3.4e-50
  3′ gene domains:
  TIC domains: NICE-3 4e-50

`N1  CCCTGAGTGAGCTGGCCACTGC  GTGGCGAAATTCACATACTCACTGTCTG`

- MAFG–SIRT7, -17:77478695..77466849 (distance: 11845 nt)
  Donor exon (score:0.997977):NM_002359.exon1/3
  Acceptor exon (score:0.994994): NM_016538.exon4/10
  Translation: tss
  5′ gene domains: bZIP_Maf 2.2e-42
  3′ gene domains: SIR2 5.1e-21
  TIC domains: SIR2 2e-18

```
HBR  GGCCCGCCGGGCCCGCAGTGTG  GCAGCGTCTATCCCAGACTACCGGGGCC
UHR                GCAGTGTG  GCAGCGTCTATCCCAGACTACCGGGGCCCTAATGGAGTGTGG
```

- SLC35E2–CDK11A, -1:1655989..1644130 (distance: 11858 nt)
  Donor exon (score:0.998715):NM_182838.exon5/6
  Acceptor exon (score:0.997735): NM_024011.exon2/20, NM_033529.exon2/20
  Translation: ptc
  5′ gene domains:
  3′ gene domains: Pkinase 5.9e-68, Pkinase_Tyr 2.8e-31
  TIC domains:
  AceView: SLC35E2andCDC2L1andCDC2L2.lApr07
  Supporting ESTs: AI814674 (pancreas neoplasia), DB316137 (brain normal)

```
HBR  AAAATCTCCCCGGTGACTTTCAG  TGTTTTAACTCAAATGGGTGATGAAAA
```

- FLCN–PLD6, -17:17059024..17047137 (distance: 11886 nt)
  Donor exon (score:0.991976):NM_144997.exon13/14
  Acceptor exon (score:0.999426): NM_178836.exon2/2
  Translation: tle
  5′ gene domains: Folliculin 2.3e-55
  3′ gene domains:
  TIC domains: Folliculin 2.3e-55

```
HBR  TCTGCCTCAAGGAGGAGTGGATGAA  GGATCCAGGTCCGGCACGATCAAaA
```

- CYB5R1–ADIPOR1, -1:201198817..201186915 (distance: 11901 nt)
  Donor exon (score:0.996904):NM_016243.exon8/9
  Acceptor exon (score:0.999220): NM_015999.exon2/8, NM_001127687.exon3/9
  Translation: ptc
  5′ gene domains: NAD_binding_1 5.1e-32, FAD_binding_6 8.4e-32
  3′ gene domains: HlyIII 1.5e-68
  TIC domains: FAD_binding_6 8.9e-32, NAD_binding_1 1.2e-17

```
UHR  TAAGCTCTGGTTCgCTCTGGATCATCCCCCAAAAG  TGTCTACCATCAGAG
```

- SLC2A11–MIF, +22:22555056..22566959 (distance: 11902 nt)
  Donor exon (score:0.992015):NM_001024939.exon8/12, NM_001024938.exon8/12, NM_030807.exon9/13
  Acceptor exon (score:0.998653): NM_002415.exon2/3
  Translation: full
  5′ gene domains: Sugar_tr 2.8e-90, MFS_1 4.4e-13
  3′ gene domains: MIF 8.2e-52
  TIC domains: Sugar_tr 6.7e-58, MFS_1 5e-13, MIF 1.7e-33

```
UHR  CGGGACTGGGAGCTGCGAGCTGCTCACGGCGGTTGTTAGT  TACATCGCGG
UHR             CTGCGAGCTGCTCACGGCGGTTGTTAGT  TACATCGCGGTGCACGTGGTCC
UHR                 GAGCTGCTCACGGCGGTTGTTAGT  TACATCGCGGTGCACGTGGTCCCGcA
```

- DHRS13–FLOT2, -17:24252134..24240170 (distance: 11963 nt)
  Donor exon (score:0.999069):NM_144683.exon4/5
  Acceptor exon (score:0.998250): NM_004475.exon2/11
  Translation: full
  5′ gene domains: adh_short 6.8e-21, KR 6.9e-09, Epimerase 3.6e-08
  3′ gene domains: Band_7 1.5e-21
  TIC domains: adh_short 1.9e-20, KR 1.7e-08, Epimerase 2e-07, Band_7 4e-19

```
 N2  CTTGAGGCCACTGGCGTCACCTGCTATGCAGCCCACCCAG  GGGGCTGTTG
HBR                GTCACCTGCTATGCAGCCCACCCAG  GGGGCTGTTGTcGTTCCGACTATAA
```

- PRR13–PCBP2, +12:52122784..52134777 (distance: 11992 nt)
  Donor exon (score:0.998516):NM_001005354.exon2/4, NM_018457.exon2/4
  Acceptor exon (score:0.995134): NM_001128914.exon2/13, NM_031989.exon2/15, NM_001098620.exon2/14, NM_001128912.exon2/15 NM_005016.exon2/15, NM_001128911.exon2/15, NM_001128913.exon2/14
  Translation: ptc
  5′ gene domains:
  3′ gene domains: KH_1 4.3e-16
  TIC domains:

```
 T2  GTGGAATCCCAATGCCG  TTTTTGGCTTTCACCC
```

- CORO7–TIMM16, -16:4345288..4333293 (distance: 11994 nt)
Donor exon (score:0.997652):NM_024535.exon27/28
Acceptor exon (score:0.999193): NM_016069.exon2/5
Translation: full
5′ gene domains: DUF1900 6.7e-50, WD40 4.9e-09
3′ gene domains: Pam16 2.4e-62
TIC domains: DUF1900 8e-50, WD40 5.7e-09, Pam16 1.3e-59
AceView: CORO7andMagmas.fApr07
Supporting ESTs: DB357305 (embryonic tissue normal)

```
HBR               ACGAGGACGAGTGG  GCCAAGTACCTGGCCCAGATCATTGTGATGGGCGTG
UHR  TTGAAGGCGTGGACGAGGACGAGTGG  GCCAAGTACCTGGCCCAGATCATT
```

- URGCP–MRPS24, -7:43887783..43875500 (distance: 12282 nt)
Donor exon (score:0.998942):NM_017920.exon4/5, NM_001077663.exon5/6, NM_001077664.exon5/6
Acceptor exon (score:0.999922): NM_032014.exon2/4
Translation: ptc
5′ gene domains:
3′ gene domains:
TIC domains:
AceView: URG4andMRPS24.pApr07
Supporting ESTs: AI027170 (parathyroid neoplasia)

```
HBR                   GGACAATGATTTTCCAgCAG  GTGCTGTCCTGGAGCCGAGAGCTGCCTTGC
UHR  GTACAAATGAGGCTCAGGACAATGATTTTCCAACAG  GgGCTGTCCTGGAG
```

- VAMP8–VAMP5, +2:85659801..85672359 (distance: 12557 nt)
Donor exon (score:0.915999):NM_003761.exon2/3
Acceptor exon (score:0.976946): NM_006634.exon2/3
Translation: full
5′ gene domains: Synaptobrevin 4.8e-33
3′ gene domains: Synaptobrevin 1.5e-26
TIC domains: Synaptobrevin 4.4e-26, Synaptobrevin 4.4e-26

```
N1  ATCCTGGCCCGGGGGGAAAACTTGGAACATCTCCGCAACAAGACAGAGGATCTGGAAGCCACA  GCAcGAATAGAG
N2                                 CCGCAACAAGACAGAGGATCTGGAAGCCACA  GCAGGAATAGAGTTGGAnC
```

- LRRC70–IPO11, +5:61910519..61923160 (distance: 12640 nt)
Donor exon (score:0.997831):NM_181506.exon1/2
Acceptor exon (score:0.998198): NM_001134779.exon28/30, NM_016338.exon28/30
Translation: tss
5′ gene domains:
3′ gene domains: IBN_N 1e-18
TIC domains:
AceView: KIF2AandIPO11.pApr07
Supporting ESTs: DA297393 (cerebrum normal), AA471187 (bone marrow neoplasia), BF214634 (bone marrow neoplasia), BF243591 (bone marrow neoplasia), BM992147 (bone normal), DA621973 (kidney normal), DA938960 (spleen normal), R56970 (heart normal), DA886353 (vascular normal), BF241594 (bone marrow neoplasia), BF243224 (bone marrow neoplasia), BM918621 (pooled tissue normal)

```
N1                      CGAATTTCCTTTTCTTG  TGTTATCCAAGATAAATTCTGTGGGATTATAAA
N2  CAGCTGAGTGTTAATAATACGAATTTCCTTTTCTTG  TGTTATCCAAGATA
```

- CHCHD10–VPREB3, -22:22438315..22425385 (distance: 12929 nt)
Donor exon (score:0.999106):NM_213720.exon3/4
Acceptor exon (score:0.990585): NM_013378.exon2/2
Translation: full
5′ gene domains: CHCH 4e-07
3′ gene domains: V-set 6.4e-14
TIC domains: CHCH 9.4e-07, V-set 3.3e-13

```
HBR  AGGGCTTCAGCGAGGCCCTGAAGCAGTGCAAGTACTACCATG  TTTCCCAG
HBR                CCCTGAAGCgGTGCAAGTACTACCATG  TTTCCCAGACAGTCCTGGCCCAG
UHR          GCGAGGCCCTGAAGCAGTGCAAGTACTACCATG  TTTCCCAGACAGTCCTG
UHR          GCGAGGCCCTGAAGCAGTGCAAGTACTACCATG  TTTCCCAGACAGTCCTG
UHR          GCGAGGCCCTGAAGCAGTGCAAGTACTACCATG  TTTCCCAGACAGTCCTG
```

- PBXIP1–PMVK, -1:153184672..153171515 (distance: 13156 nt)
  Donor exon (score:0.987623):NM_020524.exon10/11
  Acceptor exon (score:0.999073): NM_006556.exon2/5
  Translation: full
  5′ gene domains:
  3′ gene domains: P-mevalo_kinase 1.9e-51
  TIC domains: P-mevalo_kinase 3.6e-40

```
 N1  ATGACTTTGAnGACTTCATCTTCAGCCACTTCTTTGGAGACAAAGCACTGAAGAAGAG  ACTTGGAGCTGATGTCT
```

- RPL27–IFI35, +17:38404374..38417722 (distance: 13347 nt)
  Donor exon (score:0.993342):NM_000988.exon2/5
  Acceptor exon (score:0.999237): NM_005533.exon2/7
  Translation: full
  5′ gene domains: Ribosomal_L27e 1.4e-38
  3′ gene domains: NID 2e-43, IFP_35_N 3.9e-15
  TIC domains: NID 2.3e-43, IFP_35_N 4.3e-15

```
 N2  CGCTACTCCGGACGCAAAGCTGTCATCGTGAAG  GCCCTCCACGCCCTTCA
 N2            GACGCAAAGCTGTCATCGTGAAG  GCCCTCCACGCCCTTCAGGAGGAGCAG
 N2            GACnCAAAGCTGTCATCGTGAAG  GCCCTCCACGCCCTTCAGGAGGAGCAG
```

- COX8A–OTUB1, +11:63498842..63512385 (distance: 13542 nt)
  Donor exon (score:0.994922):NM_004074.exon1/2
  Acceptor exon (score:0.999299): NM_017670.exon2/7
  Translation: ptc
  5′ gene domains: COX8 6.6e-25
  3′ gene domains: Peptidase_C65 6.2e-87
  TIC domains:

```
UHR  CATTCGTTGCCGCCGGAGGGGAAGCTTGGGATCATG  GTGTTAACTGTCTG
```

- CLDND2–ETFB, -19:56562952..56549374 (distance: 13577 nt)
  Donor exon (score:0.994096):NM_152353.exon2/4
  Acceptor exon (score:0.998034): NM_001985.exon2/6
  Translation: ptc
  5′ gene domains: PMP22_Claudin 9.7e-14
  3′ gene domains: ETF 3.6e-42
  TIC domains: PMP22_Claudin 1.1e-07
  AceView: CLDND2andETFB.jApr07
  Supporting ESTs: N78358 (brain uncharacterized histology), DA070646 (cerebellum normal)

```
 N2              GCGCCTTCCTCTTCCTCGGCG  ATCCGAGTGAAGCCTGACAGGACCGGTGT
HBR  CCAGACCACGAGCGCCTTCCTCTTCCTCGGCG  ATCCGAGTGAAGCCTGAC
HBR    AGACCACGAGCGCCTTCCTCTTCCTCGGCG  ATCCGAGTGAAGCCTGACAG
UHR  CCAGACCACGAGCGCCTTCCTCTTCCTCGGCG  ATCCGAGTGAAGCCTGAC
```

- WIZ–AKAP8L, -19:15395994..15382405 (distance: 13588 nt)
  Donor exon (score:0.998939):NM_021241.exon7/8
  Acceptor exon (score:0.998186): NM_014371.exon2/14
  Translation: ptc
  5′ gene domains:
  3′ gene domains: AKAP95 1.7e-73
  TIC domains:

```
UHR  GCAACATCTACACCCTCAAATGCAG  GCTTTGTCCAGGGATCTGAAACCAC
```

- TOMM40–APOE, +19:50089163..50102857 (distance: 13693 nt)
  Donor exon (score:0.999426):NM_006114.exon6/10, NM_001128916.exon6/10, NM_001128917.exon5/9
  Acceptor exon (score:0.981551): NM_000041.exon3/4
  Translation: full
  5′ gene domains: Porin_3 6.4e-81
  3′ gene domains: Apolipoprotein 6.3e-60
  TIC domains: Porin_3 8.9e-33, Apolipoprotein 2.3e-59

```
UHR  GGAACCCAGACGTCCTCGTGGGTTCAG  GATGCCAGGCCAAGGTGGAGCAA
```

- SPINT2–C19orf33, +19:43473055..43486855 (distance: 13799 nt)
  Donor exon (score:0.999286):NM_021102.exon6/7
  Acceptor exon (score:0.998976): NM_033520.exon2/4

Translation: full
5′ gene domains: Kunitz_BPTI 2.3e-19
3′ gene domains:
TIC domains: Kunitz_BPTI 3e-19
AceView: SPINT2andC19orf33.cApr07
Supporting ESTs: AJ712734 (bone marrow uncharacterized histology)

```
 T1                        CTGCCCCTTGGCTCAAAGG  CCCTGGAGCCCACC
 T1                          GCCCCTTGGCTCAAAGG  CCCTGGAGCCCACCTC
 T1                                TGGCTCAAAGG  CCCTGGAGCCCACCTCCCAGAA
 T3                     CCCCTGCCCCTTGGCTCAAAGG  CCCTGGAGCCC
 N1             AGAATCCTCCCCTGCCCCTTGGCTCAAAGG  CCCTGGAGCCCACCTCCCAGAAGCCCGGTGTGGGGGCGGGCCACG
 N1                     CCCCTGCCCCTTGGCTCAAAGG  CCCTGGAGCCCACCTCCCAGAAGCCCGG
 N2              GAATCCTCCCCTGCCCCTTGGCTCAAAGG  CCCTGGAGCCCACCTCCCAGA
 N2                             CTTGGCTCAAAGG  CCCTGGAGCCCACCTCCCAGAAGCCCGGTGTGGGGGC
UHR  cGCCAGCAGGAGAATCCTCCCCTGCCCCTTGGCTCAAAGG  CCCTGGAGCC
```

- BCL2L2–PABPN1, +14:22847248..22861230 (distance: 13981 nt)
  Donor exon (score:0.997918):NM_004050.exon3/4
  Acceptor exon (score:0.987926): NM_004643.exon2/7
  Translation: full
  5′ gene domains: Bcl-2 1.2e-28, BH4 2.2e-12
  3′ gene domains: RRM_1 1.1e-15
  TIC domains: Bcl-2 3.9e-28, BH4 4.6e-12, RRM_1 1.3e-15
  AceView: BCL2L2andPABPN1.bApr07
  Supporting ESTs: BI910270 (uncharacterized tissue normal)

```
UHR  CGCgGCTGGCTGACTGGATCCACAGCAGTGGGGGCTGG  GAGCTGGAAGCT
UHR               CTGGATCCACAGCAGTGGGGGCTGG  GAGCTGGAAGCTATCAAAGCTCGAG
```

- ZNF606–C19orf18, -19:63191387..63177383 (distance: 14003 nt)
  Donor exon (score:0.999069):NM_025027.exon6/7
  Acceptor exon (score:0.972118): NM_152474.exon2/6
  Translation: full
  5′ gene domains: KRAB 6.4e-24
  3′ gene domains:
  TIC domains: KRAB 1.9e-24

```
 N2  CGCACTTGTCCAG  GCAGTAAACGGTnACAACCACCCAGAAACATCACCAA
```

- CCDC19–VSIG8, -1:158109358..158095326 (distance: 14031 nt)
  Donor exon (score:0.994600):NM_012337.exon11/12
  Acceptor exon (score:0.998850): NM_001013661.exon2/7
  Translation: ptc
  5′ gene domains:
  3′ gene domains: V-set 8.5e-11
  TIC domains:
  Supporting ESTs: DA055201 (cerebellum normal)

```
HBR  CATCGATGAGATCAAGAGGAAAAAGCTTGAAGAGCTGAG  CACTGCTGTCT
HBR                  AGGAAAAAGCTTGAAGAGCTGAG  CACTGCTGTCTGCTGTGCGGATCAACG
HBR                      AAAAGCTTGAAGAGCTGAG  CACTGCTGTCTGCTGTGCGGATCAACGGGGA
```

- LGR4–CCDC34, -11:27349738..27335664 (distance: 14073 nt)
  Donor exon (score:0.998490):NM_018490.exon17/18
  Acceptor exon (score:0.950091): NM_080654.exon2/3, NM_030771.exon2/6
  Translation: ptc
  5′ gene domains: 7tm_1 3.5e-15, LRRNT 6.7e-07
  3′ gene domains:
  TIC domains: LRRNT 3.5e-07

```
UHR  CCATTGgACACCTTCAACAG  CACTCAGGTTGAATCAGAAAATAACCAAGA
```

- C15orf38–AP3S2, -15:88247452..88233376 (distance: 14075 nt)
  Donor exon (score:0.991450):NM_182616.exon5/6
  Acceptor exon (score:0.997549): NM_005829.exon2/6
  Translation: full
  5′ gene domains: UPF0552 4.8e-109
  3′ gene domains: Clat_adaptor_s 1.7e-52

TIC domains: UPF0552 3e-108, Clat_adaptor_s 5.2e-41
AceView: C15orf38andAP3S2.gApr07
Supporting ESTs: BM741813 (stomach neoplasia), BM772450 (stomach neoplasia)

```
 T1                         CAGAGGACGAGGAGTGG  CCAGAAGAAATTCAAC
 T1                         CAGAGGACGAGGAGTGG  CCAGAAGAAATTCAAC
 T2                         CAGAGGACGAGGAGTGG  CCAGAAGAAATTCAAC
 T2                                CGAGGAGTGG  CCAGAAGAAATTCAACAGCAGAT
 T2                                CGAGGAGTGG  CCAGAAGAAATTCAACAGCAGAT
 T3                                CGAGGAGTGG  CCAGAAGAAATTCAACAGCcGAT
 N1              GGGGnATGGGGCAGAGGACGAGGAGTGG  CCAGAAGAAATTCAACAGCAGATTGTTCGAGAGACTTTCCATCTAGT
 N1                      GGCAGAGGACGAGGAGTGG  CCAGAAGAAATTCAACAGCAGATTGTTCGAG
 N3                     GGGCAGAGGACGAGGAGTGG  CCAGAAGAAATTC
 N3                     GGGCAGAGGACGAGGAGTGG  CCtGAAGAAATTC
HBR  ATCCGAGAGCAGGGGGATGGGGCAGAGGACGAGGAGTGG  CCAGAAGAAAT
HBR       AGAGtAGGGGGATGGGGCAGAGGACGAGGAGTGG  CCAGAAGAAATTCAAC
HBR           CAGGGGGATGGGGCAGAGGACGAGGAGTGG  CCAGAAGAAATTCAACAGCA
HBR                    GGGGCAGAGGACGAGGAGTGG  CCAGAAGAAATTCAACAGCAGATTGTTCG
UHR    CCGAGAGCAGGGGGATGGGGCAGAGGACGAGGAGTGG  CCAGAAGAAATTt
UHR                   TGGGGCAGAGGACGAGGAGTGG  CCAGAAGAAATTtAACAGCAGATTGTTC
```

- CHCHD10–VPREB3, -22:22439561..22425385 (distance: 14175 nt)
  Donor exon (score:0.998715):NM_213720.exon2/4
  Acceptor exon (score:0.990585): NM_013378.exon2/2
  Translation: tle
  5′ gene domains: CHCH 4e-07
  3′ gene domains: V-set 6.4e-14
  TIC domains:

```
HBR  AGCTCGGAGCCCTCCCAGCCTGCTGTCCAGCAG  TTTCCCAGACAGTCCTG
HBR     TCGGAGCCCTCCCAGCCTGCTGTCCAGCAG  TTTCCCAGACAGTCCTGGCC
HBR      CGGAGCCCTCCCAGCCTGCTGTCCAGCAG  TTTCCCAGACAGTCCTGGCCC
```

- GATA4–NEIL2, +8:11652001..11666364 (distance: 14362 nt)
  Donor exon (score:0.999100):NM_002052.exon6/7
  Acceptor exon (score:0.999314): NM_001135748.exon3/5, NM_001135746.exon2/5, NM_145043.exon2/5
  Translation: ptc
  5′ gene domains: GATA-N 1.2e-49, GATA 2.6e-16
  3′ gene domains: H2TH 1.6e-13, Fapy_DNA_glyco 2.1e-07
  TIC domains: GATA-N 8.7e-50, GATA 2.2e-16

```
UHR  GTCATCTCACTACGGGCACAGCAGCTCCGTGTCCCAG  GGATGCCAGAAGG
```

- KLHL22–SCARF2, -22:19130730..19116275 (distance: 14454 nt)
  Donor exon (score:0.999388):NM_032775.exon6/7
  Acceptor exon (score:0.999708): NM_153334.exon2/11, NM_182895.exon2/11
  Translation: ptc
  5′ gene domains: Kelch_1 6.1e-12, BTB 5.5e-27, Kelch_2 4.2e-07, BACK 6e-16
  3′ gene domains:
  TIC domains: Kelch_1 5.5e-12, BTB 4.8e-27, BACK 5.2e-16, Kelch_2 0.0033

```
 N1           CGGATACAGGAGGGACGTGCACCAG  CTCCCAGGTGCCCACGTGCTGCGCT
 N2              ATACAGGAGGGACGTGCACCAG  CTCCCAGGTGCCCACGTGCTGCGCTGGC
HBR         GCCGGATACAGGAGGGACGTGCACCAG  CTCCCAGGTGCCCACGTGCTGCG
HBR                    GAGGGACGTGCACCAG  CTCCCAGGTGCCgACGTGCTGCGCTGGCTGGAGG
HBR                      GGGACGTGCACCAG  CTCCCAGGTGCCCACGTGCTGCGCaGGCTGGAGGCA
UHR  CAACGATGCCGGATACAGGAGGGACGTGCACCAG  CTCCCAGGTGCCCACG
```

- SCNN1A–TNFRSF1A, -12:6328154..6313671 (distance: 14482 nt)
  Donor exon (score:0.999123):NM_001038.exon12/13
  Acceptor exon (score:0.999511): NM_001065.exon2/10
  Translation: full
  5′ gene domains: ASC 1.7e-109
  3′ gene domains: TNFR_c6 1.4e-11, Death 6.9e-18
  TIC domains: ASC 2.4e-96, TNFR_c6 3.6e-11, Death 2.1e-17
  Supporting ESTs: DA950392 (spleen normal)

```
UHR  CAAAACCAATTCTGAGTCTCCCTCTGTCACG  GTGCTCCTGGAGCTGTTGG
```

- CCDC19–VSIG8, -1:158109358..158094705 (distance: 14652 nt)
  Donor exon (score:0.994600):NM_012337.exon11/12
  Acceptor exon (score:0.999610): NM_001013661.exon3/7
  Translation: ptc
  5′ gene domains:
  3′ gene domains: V-set 8.5e-11
  TIC domains:

  `HBR  AAAAGCTTGAAGAGCTGAG  TTCCTTAGTTACCAGGACAAGAGGATCAACC`

- DCUN1D2–ADPRHL1, -13:113161628..113146905 (distance: 14722 nt)
  Donor exon (score:0.998778):NM_001014283.exon6/7
  Acceptor exon (score:0.999877): NM_138430.exon2/7, NM_199162.exon2/7
  Translation: full
  5′ gene domains: DUF298 7.6e-40
  3′ gene domains: ADP_ribosyl_GH 2.9e-30
  TIC domains: DUF298 1.2e-29, ADP_ribosyl_GH 7.6e-31

  `HBR  GCGGATGATATGTCTAACTACGATGAAGAAG  ACTACTGGTGCCTGGATGA`
  `HBR   CGGATGATATGTCTAACTACGATGAAGAAG  ACTACTGGTGCCTGGATGAT`

- ERF–GSK3A, -19:47450970..47436134 (distance: 14835 nt)
  Donor exon (score:0.991783):NM_006494.exon1/4
  Acceptor exon (score:0.999249): NM_019884.exon2/11
  Translation: full
  5′ gene domains: Ets 5.9e-36
  3′ gene domains: Pkinase 1.2e-64, Pkinase_Tyr 3.3e-26
  TIC domains: Pkinase 6.4e-65, Pkinase_Tyr 2e-26
  AceView: ERFandGSK3A.hApr07
  Supporting ESTs: BG718674 (testis normal)

  `UHR  CCCCGGGCCCCCCAGCATGAAGACCCCGGCGGACACAG  GTGACAGCGGGA`

- CDK2–RAB5B, +12:54651298..54666920 (distance: 15621 nt)
  Donor exon (score:0.997259):NM_001798.exon6/7, NM_052827.exon5/6
  Acceptor exon (score:0.999685): NM_002868.exon2/6
  Translation: ptc
  5′ gene domains: Pkinase 5.2e-47, Pkinase_Tyr 2e-28
  3′ gene domains: Ras 1.4e-61, Miro 4.1e-20, Arf 3.5e-12
  TIC domains: Pkinase 3.5e-47, Pkinase_Tyr 1.4e-28

  `UHR  ACGGAGCTTGTTATCG  GAGTGTTGAAGCCTGGAAATCCCCTCCCCTTCCC`

- ATF7–NPFF, -12:52203327..52187193 (distance: 16133 nt)
  Donor exon (score:0.997808):NM_001130060.exon11/12, NM_006856.exon11/12, NM_001130059.exon10/11
  Acceptor exon (score:0.957019): NM_003717.exon2/3
  Translation: tle
  5′ gene domains: bZIP_1 3.3e-12, bZIP_2 1.4e-09
  3′ gene domains:
  TIC domains: bZIP_1 3.1e-12, bZIP_2 1.3e-09

  `HBR  AGGCTATTTAG  GAGGAAGACAGCGAACCCCTCCCACCACAGGATGCCCAG`

- SLC29A1–HSP90AB1, +6:44308143..44324345 (distance: 16201 nt)
  Donor exon (score:0.999173):NM_001078175.exon12/14, NM_001078174.exon11/13, NM_001078176.exon11/13, NM_001078177.exon1[illegible]
  NM_004955.exon11/13
  Acceptor exon (score:0.970537): NM_007355.exon2/12
  Translation: full
  5′ gene domains: Nucleoside_tran 3.1e-129
  3′ gene domains: HSP90 6.6e-269, HATPase_c 3.7e-11
  TIC domains: Nucleoside_tran 1.2e-75, HSP90 2e-268, HATPase_c 6.9e-11

  `UHR  CGGAGCCTCACAGCTGTATTCATGTGG  ATGCCTGAGGAAGTGCACCATGG`

- PPP3CB–ZMYND17, -10:74874489..74858065 (distance: 16423 nt)
  Donor exon (score:0.956665):NM_001142353.exon12/14, NM_021132.exon12/14, NM_001142354.exon12/13
  Acceptor exon (score:0.989864): NM_001024593.exon2/7
  Translation: full
  5′ gene domains: Metallophos 2.3e-42
  3′ gene domains: zf-MYND 3.7e-11
  TIC domains: Metallophos 7.1e-42, zf-MYND 8.8e-11

```
 T2  GACGGCAGACCCTGCAAAGTG  GTATCACTGGAC
```

- KIAA1984–C9orf86, +9:138821339..138837798 (distance: 16458 nt)
  Donor exon (score:0.928349):NM_001039374.exon13/14
  Acceptor exon (score:0.999488): NM_024718.exon2/15
  Translation: full
  5′ gene domains:
  3′ gene domains: Miro 1.1e-07
  TIC domains: Miro 2.2e-07

```
HBR  CCAGGATCAGCTTTGAGAACCGGGAGGAGGATATGATCG  TGAAGATAGTG
HBR                            GGAGGATATGATCG  TGAAGATAGTGATCCGGGGAGACAGGAACACGGGCA
```

- SUN1–GET4, +7:875679..892219 (distance: 16539 nt)
  Donor exon (score:0.979221):NM_025154.exon16/18, NM_001130965.exon18/20
  Acceptor exon (score:0.998904): NM_015949.exon2/9
  Translation: ptc
  5′ gene domains: MRP 2.6e-66, Sad1_UNC 2.6e-47
  3′ gene domains: DUF410 2.1e-103
  TIC domains: MRP 2.7e-66, Sad1_UNC 6.3e-30

```
HBR  CTTCGCCGTCTAT  GTACATGTCCCAGAGCAAGCACACGGAGGCCCGGGAG
```

- SHANK3–ACR, +22:49507731..49524565 (distance: 16833 nt)
  Donor exon (score:0.998185):NM_001080420.exon22/23
  Acceptor exon (score:0.998864): NM_001097.exon2/5
  Translation: full
  5′ gene domains: SAM_1 7e-20, SAM_2 1e-12, SH3_2 3.4e-12
  3′ gene domains: Trypsin 2.4e-69
  TIC domains: SH3_2 3.8e-12, Trypsin 4.4e-68

```
UHR  CGCAGCAGCTCG  TGGCCCCTGTGGGTTACGGTTCAGGCAAAACCCACAGG
```

- HOXC6—HOXC4–HOXC5, +12:52697465..52714329 (distance: 16863 nt)
  Donor exon (score:0.999715):NM_153693.exon1/3, NM_014620.exon1/4
  Acceptor exon (score:0.974754): NM_018953.exon2/2
  Translation: tss-tle
  5′ gene domains: Homeobox 2e-22
  3′ gene domains: Homeobox 9.1e-22
  TIC domains:
  AceView: HOXC_.mApr07
  Supporting ESTs: BE396026 (uterus neoplasia), BE394964 (uterus neoplasia)

```
 T3  GGGAGAGTGACAGCAGCGCTCG  AGACGGACGGC
 T3     AGAGTGACAGCAGCGCTCG  AGACGGACGGCAAG
 T3     AGAGTGACAGCAGCGCTCG  AGACGGACGGCAAG
 T3          GACAGCAGCGCTCG  AGACGGACGGCAAGCGGTC
 T3             AGCAGCGCTCG  AGACGGACGGCAAGCGGTCCCG
 T3               CAGCGCTCG  AGACGGACGGCAAGCGGTCCCGAA
 N3  GGGAGAGTGACAGCAGCGCTCG  AGACGGACGGg
```

- SLC25A16–DNA2, -10:69916907..69899926 (distance: 16980 nt)
  Donor exon (score:0.994599):NM_152707.exon8/9
  Acceptor exon (score:0.998337): NM_001080449.exon2/20
  Translation: full
  5′ gene domains: Mito_carr 3.3e-26
  3′ gene domains: Dna2 1.3e-73
  TIC domains: Mito_carr 2.6e-25, Dna2 1.6e-73

```
 N1  GGCGAATGCAATTAGGAACTGTTCTGCCGGAATTTGAAAAGTGCCT  ATTTCAGAAGAAAGTGGTAGCTTCCTTTC
 N2                             CGGAATTTGAAAAGTGCCT  ATTTCAGAAGAAAGTGGTAGCTTCCTTTCCc
HBR                             CGGAATTTGAAAAGTGCCT  ATTTCAGAAGAAAGTGGTAGCTTCCTTTCCA
```

- S1PR5–KEAP1, -19:10489074..10471756 (distance: 17317 nt)
  Donor exon (score:0.993942):NM_030760.exon1/2
  Acceptor exon (score:0.999938): NM_203500.exon2/6, NM_012289.exon2/6
  Translation: tss
  5′ gene domains: 7tm_1 1.4e-36
  3′ gene domains: Kelch_1 9e-17, BACK 1e-32, BTB 3.2e-31, Kelch_2 2.5e-07
  TIC domains: Kelch_1 9e-17, BACK 1e-32, BTB 3.2e-31, Kelch_2 2.5e-07

```
HBR  CGCACGACCAGGGCGCAGA  GTCCCTGAGTGCCAGAGGTGGTGGTGTTGCT
```

- PRKAA1–TTC33, -5:40800373..40782878 (distance: 17494 nt)
  Donor exon (score:0.996946):NM_006251.exon8/9, NM_206907.exon9/10
  Acceptor exon (score:0.997037): NM_012382.exon2/5
  Translation: ptc
  5′ gene domains: Pkinase 2.5e-71, Pkinase_Tyr 5.8e-42
  3′ gene domains:
  TIC domains: Pkinase 2e-71, Pkinase_Tyr 4.7e-42

```
 N1  GTGGATAGTAGAACTTATCTACTGGATTTCCGTAGTATTGATG  AATGGCTTCCTTTGGGTGGAAGAGGAAAATTG
 N1                 TATCTACTGGATTTCCGTAGTATTGATG  AATGGCTTCCTTTGGGTGGAAG
 N1                                 GTAGTATTGATG  AATGGCTTCCTTTGGGTGGAAGAGGAAAATTGGTGAGA
UHR            GAACTTATCTACTGGATTTCCGTAGTATTGATG  AATGGCTTCCTTTGGGT
```

- TRIM52–GNB2L1, -5:180619608..180601951 (distance: 17656 nt)
  Donor exon (score:0.998325):NM_032765.exon1/2
  Acceptor exon (score:0.997495): NM_006098.exon2/8
  Translation: ptc
  5′ gene domains: zf-B_box 1.8e-09
  3′ gene domains: WD40 5.8e-12
  TIC domains: zf-B_box 1.6e-09

```
UHR  GGAGGTGGTGCAGGAGTACCAG  ATAAGACCATCATCATGTGGAAACTGAC
```

- PPP1R1B–STARD3, +17:35045505..35063260 (distance: 17754 nt)
  Donor exon (score:0.996977):NM_032192.exon6/7, NM_181505.exon6/7
  Acceptor exon (score:0.996681): NM_006804.exon2/15
  Translation: ptc
  5′ gene domains: DARPP-32 3.8e-42
  3′ gene domains: MENTAL 4e-73, START 5.3e-47
  TIC domains: DARPP-32 8e-42

```
HBR  AGGCTCTGAGGACCAAGTGGAAGACCCAGCACTAAGTG  CCCTGCTGCTGA
```

- ZFP41–GLI4, +8:144404885..144422905 (distance: 18019 nt)
  Donor exon (score:0.996170):NM_173832.exon2/3
  Acceptor exon (score:0.999292): NM_138465.exon2/4
  Translation: ptc
  5′ gene domains:
  3′ gene domains: zf-C2H2 5.1e-07
  TIC domains: zf-C2H2 2.6e-05

```
 T3  CCAGCATTCAG  GTCCCAGGTGTGACACCTTCAG
```

- AHRR–EXOC3, +5:481121..499265 (distance: 18143 nt)
  Donor exon (score:0.997047):NM_020731.exon9/12
  Acceptor exon (score:0.970183): NM_007277.exon2/13
  Translation: ptc
  5′ gene domains: PAS 6e-10
  3′ gene domains: Sec6 5e-160
  TIC domains: PAS 1.9e-10

```
HBR  CGCAGCCACCGCGGATGCAAA  TGCACAGAGAACACCCCTAGCATGAACAG
```

- ST20–MTHFS, -15:77987026..77968751 (distance: 18274 nt)
  Donor exon (score:0.999388):NM_001100879.exon2/3, NM_001100880.exon2/3
  Acceptor exon (score:0.998599): NM_006441.exon2/3
  Translation: full
  5′ gene domains:
  3′ gene domains: 5-FTHF_cyc-lig 8.7e-59
  TIC domains: 5-FTHF_cyc-lig 1.3e-49
  AceView: MTHFS.dApr07
  Supporting ESTs: BF793969 (adrenal cortex neoplasia), CK002935 (cerebellum normal)

```
 N2  GCGCGATCTCGGCTCACTGCAACCTCTGTCTCCCAGGTTCAG  GTGATTGC
 N3                     CAACCTCTGTCTCCCAGGTTCAG  GTGATTGCCC
UHR     CGATCTCGGCTCACTGCAACCTCTGTCTCCCAGGTTCAG  GTGATTGCCCA
UHR            GGCTCACTGCAACCTCTGTCTCCCAGGTTCAG  GTGATTGCCCACAGTGAG
```

- RPL38–TTYH2, +17:69711924..69730219 (distance: 18294 nt)
Donor exon (score:0.997470):NM_001035258.exon3/5, NM_000999.exon3/5
Acceptor exon (score:0.996037): NM_032646.exon2/14
Translation: ptc
5′ gene domains: Ribosomal_L38e 4.3e-34
3′ gene domains: Tweety 2.7e-178
TIC domains: Ribosomal_L38e 1.7e-05

```
 N1  GGAAATCAAGGACTTCCTGnTCACAGCCCGACGAAAGGATGCCAAAT  TCGCTGCTGTTCCTGGGGCTGGTGGCCG
UHR                                 CGAAAGGATGCCAAAT  TCGCTGCTGTTCCTGGGGCTGGTGGgCGCCGTCT
```

- CKLF–CMTM1, +16:65149752..65168508 (distance: 18755 nt)
Donor exon (score:0.996117):NM_016951.exon2/4, NM_181641.exon2/3, NM_001040138.exon2/4
Acceptor exon (score:0.998692): NM_181268.exon3/4, NM_181270.exon2/3, NM_181269.exon2/3, NM_181271.exon2/3, NM_052999.exon3/4
Translation: full
5′ gene domains:
3′ gene domains:
TIC domains:

```
HBR  ATTAATGAAGTGGTTATTTTGGCCTTTGCTT  GATCTTACCAACAGTATCA
```

- KIAA1984–C9orf86, +9:138818900..138837798 (distance: 18897 nt)
Donor exon (score:0.998236):NM_001039374.exon7/14
Acceptor exon (score:0.999488): NM_024718.exon2/15
Translation: ptc
5′ gene domains:
3′ gene domains: Miro 1.1e-07
TIC domains:

```
HBR  GCGAGAAGTACCGCCGG  TGAAGATAGTGATCCGGGGAGACAGGAACACGG
```

- MED8–ELOVL1, -1:43623304..43603881 (distance: 19422 nt)
Donor exon (score:0.999307):NM_052877.exon7/8
Acceptor exon (score:0.998182): NM_022821.exon2/8
Translation: ptc
5′ gene domains: Med8 1.2e-83
3′ gene domains: ELO 6.6e-63
TIC domains: Med8 1.5e-83
AceView: MED8andELOVL1.dApr07
Supporting ESTs: BI822262 (pooled tissue normal)

```
HBR           TCAAGTCGGCTTCCATGCATCCCTACCAGCG  AGTCCTTAGCCAGGATGGA
UHR  AACCAACATCAAGTCGGCTTCCATGCATCCCTACCAGCG  AGTCCTTAGCC
```

- NUDT21–AMFR, -16:55025744..55005757 (distance: 19986 nt)
Donor exon (score:0.997570):NM_007006.exon6/7
Acceptor exon (score:0.999434): NM_001144.exon2/14
Translation: ptc
5′ gene domains:
3′ gene domains: CUE 9.2e-10, zf-C3HC4 1.2e-07
TIC domains:

```
 N1  ATGACAATGCACCAGGATATGGACCCATCATTTCTAGTCTCCCTCAGCTGTTGAGCAG  GTTCTAGTAAATACCGC
 N1                              CATCATTTCTAGTCTCCCTCAGCTGTTGAGCAG  GTTCTAGTAAATACCGC
```

- PFKFB4–SHISA5, -3:48534423..48513730 (distance: 20692 nt)
Donor exon (score:0.988088):NM_004567.exon13/14
Acceptor exon (score:0.998308): NM_016479.exon2/6
Translation: ptc
5′ gene domains: 6PF2K 1.5e-102, PGAM 5.2e-37
3′ gene domains:
TIC domains: 6PF2K 1.6e-102, PGAM 5.6e-37

```
 N2  CGCACCGGGACAGGCCTCAG  CACGTGGTGAGGTGTGTATGGCTTCCCGTG
```

- KIAA1984–C9orf86, +9:138817059..138837798 (distance: 20738 nt)
Donor exon (score:0.998710):NM_001039374.exon6/14
Acceptor exon (score:0.999488): NM_024718.exon2/15
Translation: ptc

5′ gene domains:
3′ gene domains: Miro 1.1e-07
TIC domains:
AceView: TMEM141andKIAA1984andC9orf86.rApr07
Supporting ESTs: DB080725 (testis uncharacterized histology)

```
HBR  TCACGGATGAGGTCAAG  TGAAGATAGTGATCCGGGGAGACAGGAACACGG
```

- C21orf59–TCP10L, -21:32897342..32876596 (distance: 20745 nt)
  Donor exon (score:0.999075):NM_021254.exon5/7
  Acceptor exon (score:0.991567): NM_144659.exon3/5
  Translation: full
  5′ gene domains: DUF2870 2.7e-44
  3′ gene domains:
  TIC domains: DUF2870 1.4e-14
  AceView: C21orf59andTCP10LandC21orf77.qApr07
  Supporting ESTs: CN311301 (embryonic tissue normal)

```
 T1                 TATCGCCAAGATTCAGCAA  CCATTACAGCAGCA
 T3                 TATCGCaAAGATTCAGCAA  CCATTACAGCAGCA
 N2  GAAAAAAACCAAAATTATCGCCAAGATTCAGCAA  CCATTACAGCAGCAGAT
 N2  GAAAAAAACCAAAATTATCGCCAAGATTCAGCAA  CCATTACAGCAGCAGAT
 N2             AATTATCGCCAAGATTCAGCAA  CCATTACAGCAGCAGATCATCAGACTCC
HBR       AACaAAAATTATCGCCAAGATTCAGCAA  CCATTACAGCAGCAGATCATCA
HBR                 TATCGCCAAGATTCAGCAA  CCATTACAGCAGCAGATCATCAGACTCCACg
UHR                     GCCAAGATTCAGCAA  CCATTACAGCAGCAGATCATCAGACTCCACCAAGA
```

- SNCB–GPRIN1, -5:175980821..175959484 (distance: 21336 nt)
  Donor exon (score:0.999069):NM_003085.exon5/6, NM_001001502.exon6/7
  Acceptor exon (score:0.999313): NM_052899.exon2/2
  Translation: tle
  5′ gene domains: Synuclein 5.5e-56
  3′ gene domains:
  TIC domains: Synuclein 8.4e-56

```
HBR  GGGAGAGTTATGAGGACCCACCCCAG  AACCAGACAGCAGCCTTGGGTGCA
HBR      GAGTTATGAGGACCCACCCCAG  AACCAGACAGCAGCCTTGGGTGCAGGCG
```

- ASAM–HSPA8, -11:122459003..122437247 (distance: 21755 nt)
  Donor exon (score:0.975614):NM_024769.exon5/7
  Acceptor exon (score:0.994946): NM_006597.exon2/9, NM_153201.exon2/7
  Translation: full
  5′ gene domains: V-set 8.6e-16, I-set 5e-07
  3′ gene domains: HSP70 2.9e-231, MreB_Mbl 1.4e-17
  TIC domains: V-set 2.6e-15, I-set 1.5e-06, HSP70 1e-230, MreB_Mbl 3.3e-17

```
UHR  GAAAGCTGTGTGGTGCGAGTAACTGTACAGT  CAACCATGTCCAAGGGACC
```

- GFOD2–C16orf48, -16:66276861..66254960 (distance: 21900 nt)
  Donor exon (score:0.995646):NM_030819.exon3/4
  Acceptor exon (score:0.995455): NM_032140.exon6/7
  Translation: ptc
  5′ gene domains: GFO_IDH_MocA 2.2e-12
  3′ gene domains:
  TIC domains: GFO_IDH_MocA 2.2e-12

```
 N1  CATCCCCCCTCCACTCACCCGGCAGATATCCGTGAAGGCTCTAG  CTTGTTGGAGCGCAGGGACCTGTGGCGGCGG
```

- CLEC4F–FIGLA, -2:70890379..70868441 (distance: 21937 nt)
  Donor exon (score:0.996614):NM_173535.exon6/7
  Acceptor exon (score:0.999690): NM_001004311.exon2/5
  Translation: ptc
  5′ gene domains: Lectin_C 2.3e-18
  3′ gene domains: HLH 7.8e-16
  TIC domains: Lectin_C 8.9e-18

```
 N3  TTCAACGCCGCCCAGAACAAAGC  ATAAAAAATC
HBR           GCCCAGAACAAAGC  ATAAAAAATCTCAACCGTGGTTTTGCCAGATTGAAG
```

- TBC1D24–ATP6V0C, +16:2487115..2509220 (distance: 22104 nt)
Donor exon (score:0.998758):NM_020705.exon2/7
Acceptor exon (score:0.999748): NM_001694.exon2/3
Translation: ptc
5′ gene domains: TLD 2e-15, TBC 1.1e-23
3′ gene domains: ATP-synt_C 7.1e-19
TIC domains: TBC 4.7e-24
AceView: TBC1D24andATP6V0C.cApr07

```
UHR  CATCACCGTGAAGCAGAAGAG  CCCTGGGCGCTGCCTATGGCACAGCCAAG
UHR            AGCAGAAGAG  CCCTGGGCGCTGCCTATGGCACAGCCAAGAGCGGTACCGG
```

- MSMB–NCOA4, +10:51226862..51249134 (distance: 22271 nt)
Donor exon (score:0.994599):NM_002443.exon3/4
Acceptor exon (score:0.998152): NM_005437.exon2/10
Translation: ptc
5′ gene domains: PSP94 1.3e-58
3′ gene domains: ARA70 9.4e-43
TIC domains: PSP94 1.1e-35

```
T1                 AAATTTCATGTTGCACCCT  GAGCAGTGAGGAGA
T3             ACAGAAATTTCATGTTGCACCCT  GAGCAGTGAG
T3                            TGCACCCT  GAGCAGTGAGGAGAATGAATACCTT
N1  CTTGCTACGAAACAGAAATTTCATGTTGCACCCT  GAGCAGTGAGGAGAAT
N1         CGAAACAGAAATTTCATGTTGCACCCT  GAGCAGTGAGGAGAATGAATACC
```

- HBS1L–ALDH8A1, -6:135329160..135306797 (distance: 22362 nt)
Donor exon (score:0.994977):NM_006620.exon17/18
Acceptor exon (score:0.982936): NM_170771.exon2/6, NM_022568.exon2/7
Translation: full
5′ gene domains: DUF1916 2.8e-60, GTP_EFTU 1e-48, GTP_EFTU_D3 1.3e-20, GTP_EFTU_D2 2e-08
3′ gene domains: Aldedh 2.6e-169
TIC domains: DUF1916 5.9e-60, GTP_EFTU 2.4e-48, GTP_EFTU_D3 2.5e-20, GTP_EFTU_D2 3.7e-08, Aldedh 8.9e-162
AceView: HBS1LandALDH8A1.hApr07
Supporting ESTs: DW010012 (pooled tissue normal)

```
T2  GTTGTCACTGAG  ATCGAAGCCGCGGgCAAGGCC
```

- VAX2–ATP6V1B1, +2:71001923..71024296 (distance: 22372 nt)
Donor exon (score:0.999426):NM_012476.exon2/3
Acceptor exon (score:0.992581): NM_001692.exon2/14
Translation: ptc
5′ gene domains: Homeobox 3.3e-22
3′ gene domains: ATP-synt_ab 8.4e-60, ATP-synt_ab_C 1.7e-16, ATP-synt_ab_N 3.6e-10
TIC domains: Homeobox 1.1e-11

```
T3  cCTCCGAGACCCAG  CCTACAGGACTGTGTGCAG
T3   CTCCGAGACCCAG  CCTACAGGACTGTGTGCAGC
```

- MOBKL2C–MKNK1, -1:46854970..46832539 (distance: 22430 nt)
Donor exon (score:0.997057):NM_201403.exon1/4
Acceptor exon (score:0.997465): NM_198973.exon2/12, NM_003684.exon2/14, NM_001135553.exon2/13
Translation: tss
5′ gene domains: Mob1_phocein 1.1e-74
3′ gene domains: Pkinase 2.2e-66, Pkinase_Tyr 2.9e-30, Kdo 2.9e-07
TIC domains: Pkinase 2.2e-66, Pkinase_Tyr 2.9e-30, Kdo 2.9e-07
Supporting ESTs: DC415283 (uncharacterized tissue uncharacterized histology)

```
UHR  CAGAAGAGGACCC  GTTTCCATGTCAGAGGCCgATGGAGAACTGAAGATTG
```

- RDH11–VTI1B, -14:67221485..67199005 (distance: 22479 nt)
Donor exon (score:0.998581):NM_016026.exon6/7
Acceptor exon (score:0.995600): NM_006370.exon2/6
Translation: ptc
5′ gene domains: adh_short 2.6e-23, KR 1e-09
3′ gene domains: V-SNARE_C 7.7e-21, V-SNARE 1.1e-18
TIC domains: adh_short 2.2e-23, KR 8.4e-10

```
N2   TGAGATTCTAAGTGGGAATCATTTCAG  AAGAAAAGAAGAAATTGATCAGG
```

- RBM14–RBM4, +11:66141104..66163747 (distance: 22642 nt)
  Donor exon (score:0.999147):NM_006328.exon1/3
  Acceptor exon (score:0.999284): NM_002896.exon2/4
  Translation: ptc
  5′ gene domains: RRM_1 4.4e-16
  3′ gene domains: RRM_1 1.1e-16, zf-CCHC 6.1e-08
  TIC domains: RRM_1 1.8e-11, RRM_1 1.8e-11
  AceView: RBM14andTMEM137andRBM4.vbApr07
  Supporting ESTs: BQ918250 (lung neoplasia), BE262367 (brain neoplasia), BG328282 (colon neoplasia), CX871662 (embryonic tissue normal), DB494053 (brain uncharacterized histology), DC405878 (thymus uncharacterized histology), DC427705 (uncharacterized tissue normal), BP277131 (kidney uncharacterized histology), BX462562 (uncharacterized tissue neoplasia), CR996080 (uncharacterized tissue normal), DC409090 (thymus uncharacterized histology), BP326740 (prostate uncharacterized histology), CN419244 (embryonic tissue normal), DC307602 (cerebellum normal), DN993205 (brain normal), BP236465 (vascular uncharacterized histology), DC295484 (synovium uncharacterized histology), DC309044 (cerebellum normal), DC419714 (uterus uncharacterized histology), DC328123 (cerebrum normal), DC379633 (heart normal), CX785543 (embryonic tissue normal), DC355216 (vascular normal), DB497504 (brain uncharacterized histology), DC296259 (synovium uncharacterized histology), BI520153 (brain normal), DC302867 (cerebellum normal), DC308419 (cerebellum normal), DC317059 (cerebrum normal), DC366499 (uncharacterized tissue neoplasia), DC406849 (thymus uncharacterized histology), DC419899 (uterus uncharacterized histology), DC390921 (spleen normal), DA034366 (brain normal), DC422628 (uterus uncharacterized histology), DC422778 (uterus uncharacterized histology), BI602972 (brain normal), BQ438232 (uterus neoplasia), DA694494 (uncharacterized tissue neoplasia), DC310706 (cerebellum normal), DC424980 (cartilage normal), BI818673 (pooled tissue normal), BP279968 (cerebrum neoplasia), AU130147 (testis neoplasia), DC354532 (kidney normal), CN419230 (embryonic tissue normal), DB278934 (uterus uncharacterized histology), DB497283 (brain uncharacterized histology), DC331315 (cerebrum normal), DC413725 (ovary neoplasia), BQ897968 (brain neoplasia), DC323462 (brain normal), BI767632 (pooled tissue normal), BM544205 (ovary normal), BP198753 (endocrine normal), BX463075 (liver normal), BG114219 (bone neoplasia), BP351034 (esophagus neoplasia), DC401880 (testis uncharacterized histology), DC410340 (thymus uncharacterized histology), DC418021 (uncharacterized tissue uncharacterized histology), DC418681 (uncharacterized tissue uncharacterized histology), BQ940143 (peripheral nervous system normal), DC392644 (stomach normal), DC415444 (uncharacterized tissue uncharacterized histology)

```
N1   GCAGCCTCTTCGAGCGCCGCGGACGCGTCATCGAGTGTGACGTGGTGAAAG  GCTCTTGTCAGGATGGTGAAGCTG
N1      GCCTCTTCGAGCGCCGCGGACGCGTCATCGAGTGTGACGTGGTGAAAG  GCTCTTGTCAGGATGGTGAAGCTGTTC
N1                   CCGCGGACGCGTCATCGAGTGTGACGTGGTGAAAG  GCTCTTGTCAGGATGGTGAAGCTGTTCATCGGAAACCTGC
N1                    CGCGGACGCGTCATCGAGTGTGACGTGGTGAAAG  GCTCTTGTCAGGATGGTGAAGCTGTTCATCGGAAACCTGCC
N1                          CGCGTCATCGAGTGTGACGTGGTGAAAG  GCTCTTGTCAGGATGGTGAAGCTGTTCATCGGAAACCTGCCCCGGGA
N1                           GCGTCATCGAGTGTGACGTGGTGAAAG  GCTCTTGTCAGGATGGTGAAGCTGTTCATCGGAAACCTGCCCCGGGAG
N1                           GCGTCATCGAGTGTGACGTGGTGAAAG  GCTCTTGTCAGGATGGTGAAGCTGTTCATCGGAAACCTGCCCCGGGAG
N2                    CGCGGACGCGTCATCGAGTGTGACGTGGTGAAAG  GCTCTTGTCAGGATGG
HBR                      GGACGCGTCATCGAGTGTGACGTGGTGAAAG  GCTCTTGTCgGGATGGTGA
HBR                                     TGTGACGTGGTGAAAG  GCTCTTGTCAGGATGGTGAgGCTGTTCATCGGAA
HBR                                          CGTGGTGAAAG  GCTCTTGTCAGGATGGTGAAGCTGTTCATCGGAAACCTG
UHR                   CGCGGACGCGTCATCGAGTGTGACGTGGTGAAAG  GCTCTTGTCAGGATGG
UHR                       GACGCGTCATCGAGTGTGACGTGGTGAAAG  GCTCTTGTCAGGATGGTGAA
UHR                          GCGTCATCGAGTGTGACGTGGTGAAAG  GCTCTTGTCAGGATGGTGAAGCT
UHR                                TCGAGTGTGACGTGGTGAAAG  GCTCTTGTCAGGATGGTGAAGCTGTTCAT
UHR                                  GAGTGTGACGTGGTGAAAG  GCTCTTGTCAGGATGGTGAAGCTGTTCATCG
UHR                                          CGTGGTGAAAG  GCTCTTGTCAGGATGGTGAAGCTGTTCATCGGAAACCTG
UHR                                          CGgGGTGAAAG  GCTCTTGTCAGGATGGTGAAGCTGTTCATCGGAAACCTG
```

- LMAN2–MXD3, -5:176693891..176671091 (distance: 22799 nt)
  Donor exon (score:0.998607):NM_006816.exon7/8
  Acceptor exon (score:0.967284): NM_031300.exon2/6, NM_001142935.exon2/6
  Translation: full
  5′ gene domains: Lectin_leg-like 1.5e-100
  3′ gene domains: HLH 2.7e-10
  TIC domains: Lectin_leg-like 3.1e-100, HLH 1.1e-09
  AceView: LMAN2andMXD3andRAB24.aApr07
  Supporting ESTs: BX375057 (uncharacterized tissue neoplasia)

```
N2                            CCTCAAGTCGCCCAAAG  AGGCCGAGCATGGTTATGCGTCCCTGTGCCCGC
UHR  CCAAGATCGAGCCCAGCGTCAACTTCCTCAAGTCGCCCAAAG  AGGCCGAG
UHR                            CTCAAGTCGCCCAAAG  AGGCCGAGCATGGTTATGCGTCCCTGTGCCCGCA
```

- MSMB–NCOA4, +10:51225842..51249134 (distance: 23291 nt)
  Donor exon (score:0.997373):NM_138634.exon2/3, NM_002443.exon2/4

Acceptor exon (score:0.998152): NM_005437.exon2/10
Translation: full
5′ gene domains: PSP94 1.3e-58
3′ gene domains: ARA70 9.4e-43
TIC domains: PSP94 2.5e-10, ARA70 1e-42
Supporting ESTs: DB215804 (uncharacterized tissue uncharacterized histology), DB234208 (uncharacterized tissue uncharacterized histology), DB240089 (uncharacterized tissue uncharacterized histology)

```
 T2           GATTCAACCAGGA  GAGCAGTGAGGAGAATGAAT
 N1      TCCAGGAGATTCAACCAGGA  GAGCAGTGAGGAGAATGAATACCTTCCAAG
 N3  AGTTCCAGGAGATTCAACCAGGA  GAGCAGTGAG
```

- ZFP41–GLI4, +8:144404885..144428249 (distance: 23363 nt)
  Donor exon (score:0.996170):NM_173832.exon2/3
  Acceptor exon (score:0.990056): NM_138465.exon3/4
  Translation: ptc
  5′ gene domains:
  3′ gene domains: zf-C2H2 5.1e-07
  TIC domains: zf-C2H2 2.6e-05
  AceView: ZFP41andGLI4.nApr07

```
 T2                        GCCCAGCATTCAG  GCTCCCCTGGCTCCAGCCCT
 N1   GGCCCCAGCCCCACAGACCAGCCCAGCATTCAG  GCTCCCCTGGCTCCAGCCCTAAGGTGCTCTCCCAGCCGTCCG
 N3                           CAGCATTCAG  GCTCCCCTGGCTCCAGCCCTAAG
HBR                ACAGACCAGCCCAGCATTCAG  GCTCCCCTGGCTCCAGCCCTAAGGTGCTC
UHR  GGGCCCCAGCCCCACAGACCAGCCCAGCATTCAG  GCTCCCCTGGCTCCAG
UHR                ACAGACCAGCCCAGCATTCAG  GCTCCCCTGGCTCCAGCCCTAAGGTGCTC
```

- ENTPD5–FAM161B, -14:73506466..73483061 (distance: 23404 nt)
  Donor exon (score:0.999426):NM_001249.exon15/16
  Acceptor exon (score:0.999440): NM_152445.exon2/9
  Translation: full
  5′ gene domains: GDA1_CD39 5.2e-76
  3′ gene domains: UPF0564 6.7e-84
  TIC domains: GDA1_CD39 7.2e-70, UPF0564 2e-83

```
 N1                      nCAGTCTTACAG  ATATTTCCCCCCGAGTCCTTCGCAGACACAGAGGCAGG
 N2  CTTTGGCTTTGCAGACAGCACAGTCTTACAG  ATATTTCCCCCCGAGTCCT
```

- CNPY3–GNMT, +6:43013923..43037928 (distance: 24004 nt)
  Donor exon (score:0.996796):NM_006586.exon5/6
  Acceptor exon (score:0.985408): NM_018960.exon2/6
  Translation: ptc
  5′ gene domains: DUF3456 2e-44
  3′ gene domains: Methyltransf_12 1.5e-09, Methyltransf_11 1e-08
  TIC domains: DUF3456 9.6e-45

```
HBR  CAACCACGTGCTGAAGGGAAAAGACACCA  GGTGGACTCCATTATGCTGGT
```

- NDUFA1–AKAP14, +X:118890004..118914146 (distance: 24141 nt)
  Donor exon (score:0.999161):NM_004541.exon1/3
  Acceptor exon (score:0.990097): NM_001008534.exon2/6, NM_178813.exon2/7, NM_001008535.exon2/4
  Translation: ptc
  5′ gene domains:
  3′ gene domains:
  TIC domains:
  Supporting ESTs: DT932708 (pooled tissue normal)

```
HBR  GTTCACTAACGGGGGCAAG  CACTTGCAAACATTCCATCTGGCAATCTGAG
```

- KIF1B–PGD, +1:10358018..10382273 (distance: 24254 nt)
  Donor exon (score:0.999379):NM_015074.exon46/47
  Acceptor exon (score:0.997958): NM_002631.exon2/13
  Translation: full
  5′ gene domains: Kinesin 4.5e-119, DUF3694 4.3e-45, PH 1e-12, KIF1B 1.9e-09, FHA 1.3e-07
  3′ gene domains: 6PGD 2.4e-138, NAD_binding_2 2.7e-50
  TIC domains: Kinesin 6.5e-119, DUF3694 5.7e-45, PH 1.4e-12, KIF1B 2.5e-09, FHA 1.7e-07, 6PGD 4.8e-137, NAD_binding_2 3.6e-49

```
HBR  CTTCAACCCACTTCTAGCTGGCACAATACG  AGCTGACATCGCGCTGATCG
```

- C21orf59–TCP10L, -21:32897342..32873012 (distance: 24329 nt)
  Donor exon (score:0.999075):NM_021254.exon5/7
  Acceptor exon (score:0.999503): NM_144659.exon4/5
  Translation: full
  5′ gene domains: DUF2870 2.7e-44
  3′ gene domains:
  TIC domains: DUF2870 3.7e-15

```
 T3  AAATTATCGCCAAGATTCAGCAA  GCATTGGAAC
 T3       ATCGCCAAGATTCAGCAA  GCATTGGAACCTGCa
```

- NCDN–TFAP2E, +1:35802097..35826518 (distance: 24420 nt)
  Donor exon (score:0.999426):NM_001014839.exon7/8, NM_001014841.exon6/7, NM_014284.exon6/7
  Acceptor exon (score:0.999272): NM_178548.exon4/7
  Translation: full
  5′ gene domains: Neurochondrin 6e-205
  3′ gene domains: TF_AP-2 3e-93
  TIC domains: Neurochondrin 8.8e-197, TF_AP-2 1.1e-92

```
HBR  TGGGGCTCCTCATGGCCCGGCTCCTTAGCACCTCTCCAG  TGCCCATCCCC
```

- PLAUR–CADM4, -19:48848217..48823782 (distance: 24434 nt)
  Donor exon (score:0.998297):NM_001005376.exon6/7, NM_002659.exon6/7, NM_001005377.exon5/6
  Acceptor exon (score:0.991342): NM_145296.exon2/9
  Translation: full
  5′ gene domains: UPAR_LY6 2e-11
  3′ gene domains: I-set 3.1e-12, V-set 7.4e-07, C2-set_2 1.9e-11
  TIC domains: UPAR_LY6 1.1e-10, I-set 5.7e-12, V-set 1.3e-06, C2-set_2 3.5e-11

```
UHR  CTGGTAGCCACCGGCACTCACG  GGGCAGGACAGGAAGTACAGACAGAGAA
```

- PXMP2–PGAM5, +12:131777035..131801517 (distance: 24481 nt)
  Donor exon (score:0.998948):NM_018663.exon2/5
  Acceptor exon (score:0.957328): NM_138575.exon2/6
  Translation: full
  5′ gene domains: Mpv17_PMP22 2.7e-21
  3′ gene domains: PGAM 5.5e-15
  TIC domains: PGAM 6.4e-15

```
HBR  CTCTGAGATATGCCGTTTACGG  GCGAGAACCACTGTCTCTGATCAACGTG
HBR    CTGAGATATGCCGTTTACGG  GCGAGAACCACTGTCTCTGATCAACGTGCG
UHR    CTGAGATATGCCGTTTACGG  GCGAGAACCACTGTCTCTGATCAACGTGCG
```

- INMT–FAM188B, +7:30760079..30784573 (distance: 24493 nt)
  Donor exon (score:0.998583):NM_006774.exon2/3
  Acceptor exon (score:0.997543): NM_032222.exon2/18
  Translation: ptc
  5′ gene domains: NNMT_PNMT_TEMT 1.5e-102
  3′ gene domains:
  TIC domains: NNMT_PNMT_TEMT 2.4e-51
  AceView: INMT.lApr07
  Supporting ESTs: BQ722411 (peripheral nervous system normal)

```
 N2  GGTGAAATTCGCCTGTGAGCTGGAAGGAAACAG  GGCTTAAAGAAGACATG
```

- MARCH2–HNRNPM, +19:8401751..8426289 (distance: 24537 nt)
  Donor exon (score:0.999088):NM_001005415.exon4/5, NM_016496.exon5/6
  Acceptor exon (score:0.995618): NM_031203.exon2/17, NM_005968.exon2/16
  Translation: ptc
  5′ gene domains: zf-C3HC4 1.8e-10
  3′ gene domains: RRM_1 2.8e-19, HnRNP_M 6.5e-16
  TIC domains: zf-C3HC4 1.2e-10

```
HBR     CTGGACGCTG  TGAAGGAGAACGACCTGCTCAGAATGAGAAGAGGAAGGAG
UHR  CCTCTGGACGCTG  TGAAGGAGAACGACCTGCTCAGAATGAGAAGAGGAAG
```

- POLA2–CDC42EP2, +11:64820037..64844591 (distance: 24553 nt)
  Donor exon (score:0.999199):NM_002689.exon17/18
  Acceptor exon (score:0.998507): NM_006779.exon2/2
  Translation: tle
  5′ gene domains: Pol_alpha_B_N 1.4e-51, DNA_pol_E_B 2.8e-43
  3′ gene domains: PBD 6e-09
  TIC domains: Pol_alpha_B_N 1.2e-51, DNA_pol_E_B 2.5e-43

```
UHR  TCAGAGCTGAGGTACTTCGTGAAG  CTCCTCAGCCCTGGACCGGGGACAAG
```

- ATP5J2–PTCD1, -7:98895645..98870827 (distance: 24817 nt)
  Donor exon (score:0.974262):NM_001039178.exon2/3, NM_001003713.exon2/4, NM_004889.exon2/4, NM_001003714.exon2/3
  Acceptor exon (score:0.998030): NM_015545.exon2/8
  Translation: full
  5′ gene domains: WRW 9e-13
  3′ gene domains:
  TIC domains: WRW 3.6e-09
  Supporting ESTs: DC336558 (brain normal)

```
 N3  GTTTCAAAGAG  GCCCTGGAGGGACAGGAAAGCC
```

- ZNF343–SNRPB, -20:2421345..2396404 (distance: 24940 nt)
  Donor exon (score:0.998158):NM_024325.exon5/6
  Acceptor exon (score:0.999707): NM_198216.exon2/7, NM_003091.exon2/7
  Translation: ptc
  5′ gene domains: zf-C2H2 6.2e-07, KRAB 4.2e-21
  3′ gene domains: LSM 1.4e-18
  TIC domains: KRAB 2.1e-22
  AceView: ZNF343andSNRPB.tApr07
  Supporting ESTs: BG763634 (skin neoplasia)

```
 N2   AGGAATCTTCTCTCATTGG  ACGGTGGGCAAGAGCAGCAAGATGCTGCAGC
UHR  CAGGAATCTTCTCTCATgGG  ACGGTGGGCAAGAGCAGCAAGATGCTGCAG
```

- SUMO2–HN1, -17:70682442..70656361 (distance: 26080 nt)
  Donor exon (score:0.997420):NM_006937.exon3/4
  Acceptor exon (score:0.996447): NM_001002033.exon3/6, NM_016185.exon2/5, NM_001002032.exon2/4
  Translation: ptc
  5′ gene domains: Rad60-SLD 2.1e-26, ubiquitin 2.8e-12
  3′ gene domains:
  TIC domains: Rad60-SLD 4.9e-19, ubiquitin 6.6e-09

```
 N1  TGACGGGCAACCAATCAATGAAACAGACACACCTGCACAG  AGTTTTGCGG
 N1  TGACGGGCAACCAATCAATGAAACAcACACACCTGCACAG  AGTTTTGCGGCCTCCAGGTGGTGGATCCAATTTTT
```

- ACAD10–ALDH2, +12:110677932..110704105 (distance: 26172 nt)
  Donor exon (score:0.998715):NM_001136538.exon21/22, NM_025247.exon20/21
  Acceptor exon (score:0.999595): NM_000690.exon2/13
  Translation: full
  5′ gene domains: APH 3.1e-48, Acyl-CoA_dh_1 2e-34, Acyl-CoA_dh_2 5.3e-14, Acyl-CoA_dh_N 1.5e-11, Acyl-CoA_dh_M 1.8e-11, Hydrolase 1.6e-08
  3′ gene domains: Aldedh 8.4e-188
  TIC domains: APH 5.3e-48, Acyl-CoA_dh_1 1.6e-21, Acyl-CoA_dh_N 2.3e-11, Acyl-CoA_dh_M 2.7e-11, Hydrolase 2.6e-08, Acyl-CoA_dh_2 1.6e-08, Aldedh 1.4e-186
  Supporting ESTs: BG744459 (uncharacterized tissue normal)

```
HBR  CCATGGCCTCCCGAGTGATTGATCGTGCGATTCAG  ATTTTCATAAACAAT
```

- ROPN1–CCDC14, -3:125176916..125150669 (distance: 26246 nt)
  Donor exon (score:0.996827):NM_017578.exon5/7
  Acceptor exon (score:0.988014): NM_022757.exon5/12
  Translation: ptc
  5′ gene domains:
  3′ gene domains:
  TIC domains:

```
UHR  GTTTTTAGCCCTTGCTTGCAGCGCTCTGGGAGTT  CATCTTCAGATAATAA
```

- RBM14–RBM4, +11:66141104..66167497 (distance: 26392 nt)
Donor exon (score:0.999147):NM_006328.exon1/3
Acceptor exon (score:0.993211): NM_002896.exon3/4
Translation: full
5′ gene domains: RRM_1 4.4e-16
3′ gene domains: RRM_1 1.1e-16, zf-CCHC 6.1e-08
TIC domains: RRM_1 8.7e-11, RRM_1 8.7e-11, zf-CCHC 5.6e-08
AceView: RBM14andTMEM137andRBM4.kApr07
Supporting ESTs: BI821195 (pooled tissue normal)

```
HBR  GACGCGTCATCGAGTGTGACGTGGTGAAAG  GCAAACGAATGCACGTGCAG
UHR                TGTGACGTGGTGAAAG  GCAAACGAATGCACGTGCAGTTGTCCACCAGCCG
```

- HIGD1A–CCDC13, -3:42802524..42774847 (distance: 27676 nt)
Donor exon (score:0.992406):NM_001099668.exon3/4, NM_014056.exon3/4, NM_001099669.exon3/4
Acceptor exon (score:0.969554): NM_144719.exon2/16
Translation: ptc
5′ gene domains: HIG_1_N 7.1e-23
3′ gene domains:
TIC domains: HIG_1_N 5.1e-23
AceView: HIGD1AandCCDC13andHHATL.fApr07
Supporting ESTs: CU450571 (skin normal), DA307567 (cerebrum normal)

```
HBR  GCTTTGTTGTAGGAGCAATGACTGTTG  GGCAGGATGGCAGCAGATGAAAG
```

- UPK3BL—POLR2J2–RASA4, -7:102066397..102038652 (distance: 27744 nt)
Donor exon (score:0.999088):NM_001114403.exon5/6, NM_032959.exon8/9
Acceptor exon (score:0.999467): NM_001079877.exon2/20, NM_006989.exon2/21
Translation: full,ptc
5′ gene domains: RNA_pol_L 1.7e-08
3′ gene domains: RasGAP 6.4e-46, C2 6e-22, BTK 5.4e-13
TIC domains: RNA_pol_L 1.7e-08, RasGAP 9.3e-46, C2 8e-22, BTK 7e-13

```
 N2                      TGTGCTCATATACACCTG  CACTGGCAGCAGCGACCCCTACTGCATCGTGA
HBR  CTCCTCACGGTCCTCCTGGCTGTGCTCATATACACCTG  CACTGGCAGCAG
HBR      TCACGGTCCTCCTGGCTGTGCTCATATACACCTG  CACTGGCAGCAGCGAC
HBR                 CTGGCTGTGCTCATATACACCTG  CACTGGCAGCAGCGACtCCTACTGCAT
UHR      TCACGGTCCTCCTGGCTGTGCTCATATACACCTG  CACTGGCAGCAGCGAC
UHR      TCACGGTCCTCCTGGCTGTGCTCATATACACCTG  CACTGGCAGCAGCGAC
UHR         CGGTCCTCCTGGCTGTGCTCATATACACCTG  CACTGGCAGCAGCGACCCC
UHR                        CTCATATACACCTG  CACTGGCAGCAGCGACCCCTACTGCATCGTGAAGGT
UHR                          CATATACACCTG  CACTGGCAGCAGCGACCCCTACTGCATCGTGAAGGTGG
```

- ZHX1–C8orf76, -8:124348694..124320565 (distance: 28128 nt)
Donor exon (score:0.994286):NM_007222.exon2/4, NM_001017926.exon1/3
Acceptor exon (score:0.776020): NM_032847.exon2/6
Translation: tss
5′ gene domains: Homeobox 1.6e-07, Homez 5.3e-22
3′ gene domains:
TIC domains:
AceView: ZHX1andC8orf76.fApr07
Supporting ESTs: BF035882 (ovary neoplasia), BP340117 (spleen normal), DA893729 (muscle normal)

```
 N1  GGAAGCTGTGGCAA  TGGTTTTATGAAGAAACAGAAAGCAGTGATGATGTT
UHR   GAAGCTGTGGCAA  TGGTTTTATGAAGAAACAGAAAGCAGTGATGATGTTG
```

- C11orf59–NUMA1, -11:71486983..71458605 (distance: 28377 nt)
Donor exon (score:0.998325):NM_017907.exon4/5
Acceptor exon (score:0.999549): NM_006185.exon2/27
Translation: ptc
5′ gene domains:
3′ gene domains:
TIC domains:

```
UHR  ATTTGCAGCAG  GTGGAAACTAATTCTAAGCCAGACTGCTGGAGATCACCC
```

- TF–SRPRB, +3:134978772..135007210 (distance: 28437 nt)
Donor exon (score:0.995612):NM_001063.exon16/17
Acceptor exon (score:0.998844): NM_021203.exon2/8

Translation: ptc
5′ gene domains: Transferrin 9e-166
3′ gene domains: SRPRB 4.6e-74, Arf 2e-14
TIC domains: Transferrin 8.7e-166

```
HBR  GCTGTTGGTAACCTGAGAAAATGCTCCACCTCAT  GAGGACGGAGTCCCAG
```

- KIAA1841–C2orf74, +2:61214915..61243481 (distance: 28565 nt)
  Donor exon (score:0.999281):NM_032506.exon21/22
  Acceptor exon (score:0.998813): NM_001143959.exon2/4, NM_001143960.exon2/4
  Translation: ptc
  5′ gene domains: DUF3342 1e-106
  3′ gene domains:
  TIC domains: DUF3342 1e-106
  AceView: KIAA1841.fApr07

```
HBR  GAATACTGCTTGACATATC  TTTCCAAGGCAGGAAAGGTAAAGAGACAAAG
```

- TGIF2–C20orf24, +20:34640783..34669532 (distance: 28748 nt)
  Donor exon (score:0.995088):NM_021809.exon2/3
  Acceptor exon (score:0.999749): NM_018840.exon2/4, NM_199483.exon2/3
  Translation: full
  5′ gene domains: Coprinus_mating 8.7e-07
  3′ gene domains: Rab5ip 8.4e-26
  TIC domains: Rab5ip 1.4e-25
  AceView: TGIF2andC20orf24.eApr07
  Supporting ESTs: BX377269 (placenta normal), BX377268 (placenta normal)

```
UHR  GGACAGACCAACCTGTCAGTGCTGCAA  GATGAATTTTTAGATGTGATCTA
```

- RBM14–RBM4, +11:66141104..66170074 (distance: 28969 nt)
  Donor exon (score:0.999147):NM_006328.exon1/3
  Acceptor exon (score:0.999834): NM_002896.exon4/4
  Translation: tle
  5′ gene domains: RRM_1 4.4e-16
  3′ gene domains: RRM_1 1.1e-16, zf-CCHC 6.1e-08
  TIC domains: RRM_1 1e-11, RRM_1 1e-11
  AceView: RBM14andTMEM137andRBM4.veApr07
  Supporting ESTs: AA384695 (thyroid normal), AA410815 (ovary neoplasia), BE885635 (uterus neoplasia), AW958943 (uncharacterized tissue uncharacterized histology)

```
 N1   CGCGGACGCGTCATCGAGTGTGACGTGGTGAAAG  GTGGGATGTGTGTGGnCTGAAATTCCGAGCTGCGGTTGTGC
 N2  CCGCGGACGCGTCATCGAGgGTGACGTGGTGAAAG  GTGGGATGTGTGTGG
 N2   CGCGGACGCGTCATCGAGTGTGACGTGGTGAAAG  GTGGGATGTGTGTGGG
 N2          GCGTCATCGAGTGTGACGTGGTGAAAG  GTGGGATGTGTGTGGGCTGAAAT
UHR             TCATCGAGTGTGACGTGGTGAAAG  GTGGGATGTGTGTGGGgTGAAATTCC
```

- CACNG4–CACNG1, +17:62451578..62480574 (distance: 28995 nt)
  Donor exon (score:0.998715):NM_014405.exon3/4
  Acceptor exon (score:0.999378): NM_000727.exon2/4
  Translation: full
  5′ gene domains: PMP22_Claudin 3.3e-40
  3′ gene domains: PMP22_Claudin 1.5e-11
  TIC domains: PMP22_Claudin 1.5e-21, PMP22_Claudin 1.5e-21

```
HBR  GGCATCCTCTTCGTGGCTGCAG  AGAAGAACTGTTCCTACTTCAGGCATTT
```

- TCN2–SLC35E4, +22:29343482..29372585 (distance: 29102 nt)
  Donor exon (score:0.973998):NM_000355.exon7/9
  Acceptor exon (score:0.998526): NM_001001479.exon2/2
  Translation: tle
  5′ gene domains: Cobalamin_bind 2e-131
  3′ gene domains: TPT 2.7e-19, EamA 2.1e-08, UAA 7.6e-10
  TIC domains: Cobalamin_bind 2.1e-131

```
HBR  GTTAGGAGGATTCAC  GTGCCCTGCTGCAGGAGGAGAGGCTGGACGCGGcG
```

- PIGZ–NCBP2, -3:198179955..198150700 (distance: 29254 nt)
  Donor exon (score:0.998948):NM_025163.exon1/3
  Acceptor exon (score:0.989840): NM_001042540.exon2/4, NM_007362.exon2/4

Translation: tss,tss-tle
5′ gene domains: Glyco_transf_22 7.7e-59
3′ gene domains: RRM_1 1.7e-17
TIC domains: RRM_1 1.5e-05

```
HBR  CCGCCAGCGCTGAAGGCGCAGCCCGGAGGCCGCGCGG  GGTGACAATGAAG
```

- MAP1S–FCHO1, +19:17705237..17734571 (distance: 29333 nt)
  Donor exon (score:0.998827):NM_018174.exon6/7
  Acceptor exon (score:0.999097): NM_015122.exon5/29
  Translation: full
  5′ gene domains:
  3′ gene domains: SAFF 2.3e-20, FCH 2.5e-16
  TIC domains: SAFF 5.8e-20, FCH 7e-14

```
HBR  GCAAGCAGCATTGGGACCGTGACCTGCAG  GGCGAGAAAAATCATGGCTTT
```

- LIPT1–MRPL30, +2:99139321..99169072 (distance: 29750 nt)
  Donor exon (score:0.997346):NM_145197.exon2/4, NM_015929.exon2/3, NM_145198.exon2/4
  Acceptor exon (score:0.996491): NM_145212.exon2/6
  Translation: tss
  5′ gene domains: BPL_LplA_LipB 9.9e-22
  3′ gene domains: Ribosomal_L30 6.7e-10
  TIC domains: Ribosomal_L30 6.7e-10
  AceView: C2orf15andLIPT1andMRPL30.oApr07
  Supporting ESTs: DA451563 (uncharacterized tissue neoplasia), DB492713 (brain uncharacterized histology), AA772463 (parathyroid neoplasia)

```
 T3       cGCCACCACGCCCAG  CCTCTAAAGAGAGCAATC
 N1  GCGCCcGCCACCACGCCCAG  CCTCTAAAGAGAGCAATCACTACACTTATG
```

- CLN6–CALML4, -15:66308894..66279073 (distance: 29820 nt)
  Donor exon (score:0.996283):NM_017882.exon1/7
  Acceptor exon (score:0.990848): NM_033429.exon3/5
  Translation: ptc
  5′ gene domains:
  3′ gene domains:
  TIC domains:

```
UHR  CAGCTGGGCGCCTCCTTCCTGCAGGCCAG  AGTACAAGGAATGCTTCTCCC
```

- UPK3BL—POLR2J2–RASA4, -7:102066806..102036964 (distance: 29841 nt)
  Donor exon (score:0.997691):NM_001114403.exon4/6, NM_032959.exon7/9
  Acceptor exon (score:0.999087): NM_001079877.exon3/20, NM_006989.exon3/21
  Translation: ptc
  5′ gene domains: RNA_pol_L 1.7e-08
  3′ gene domains: RasGAP 6.4e-46, C2 6e-22, BTK 5.4e-13
  TIC domains: RNA_pol_L 1.7e-08

```
 N2            GCaACACTCGCCTGCAGCAAG  GACAGCCACAGTGTGGAAGACCCTGTGCC
 N2             CaACACTCGCCTGCAGCAAG  GACAGCCACAGTGTGGAAGACCCTGTGCCC
HBR       GTCCAGCaACACTCGCCTGCAGCAAG  GACAGCCACAGTGTGGAAGACCCT
HBR                  CTCGCCTGCAGCAAG  GACAGCCACAGTGTGGAAGACCCTGTGCCCCTTCT
UHR  AAGTGGTCCAGCaACACTCGCCTGCAGCAAG  GACAGCCACAGTGTGGAAG
UHR      GGTCCAGCaACACTCGCCTGCAGCAAG  GACAGCCACAGTGTGGAAGACCC
UHR            GCaACACTCGCCTGCAGCAAG  GACAGCCACAGTGTGGAAGACCCTGTGCC
UHR                  CTCGCCTGCAGCAAG  GACAGCCACAGTGTGGAAGACCCTGTGCCCCTTCT
UHR                  CTCGCCTGCAGCAAG  GACAGCCACAGTGTGGAAGACCCTGTGCCCCcTCT
UHR                     GCCTGCAGCAAG  GACAGCCACAGTGTGGAAGACCCTGTGCCCCTTCTGGG
```

- CHST6–TMEM170A, -16:74073216..74043238 (distance: 29977 nt)
  Donor exon (score:0.997977):NM_021615.exon2/3
  Acceptor exon (score:0.999033): NM_145254.exon2/3
  Translation: tss
  5′ gene domains: Sulfotransfer_1 2.5e-21
  3′ gene domains: Tmemb_170 1.9e-48
  TIC domains: Tmemb_170 5.1e-46

```
HBR  TGCAGCTCTCCCAGCAGCCCTGGACAGTG  AGATGTGGTATGGTGTATTCC
```

- ADCY3–C2orf79, -2:24897097..24866954 (distance: 30142 nt)
  Donor exon (score:0.998881):NM_004036.exon20/21
  Acceptor exon (score:0.999801): NM_001013663.exon2/2
  Translation: full
  5′ gene domains: Guanylate_cyc 3e-67
  3′ gene domains: PTH2 1.2e-09
  TIC domains: Guanylate_cyc 3.6e-61

```
 T3  CACGGGGGTCATGGGCAACATTCAG  GCCCCAGA
HBR     GGGGGTCATGGGCAACATTCAG  GCCCCAGATGAGACCACCCTcAAGGAGC
HBR                    ACATTCAG  GCCCCAGATGAcACCACCCTAAAGGAGCTGGCCGAGACCCTG
```

- WNT10B–ARF3, -12:47651505..47621238 (distance: 30266 nt)
  Donor exon (score:0.997880):NM_003394.exon1/5
  Acceptor exon (score:0.998779): NM_001659.exon2/5
  Translation: tss
  5′ gene domains: wnt 3.3e-116
  3′ gene domains: Arf 5.1e-79, SRPRB 5.2e-15, Ras 2.4e-13, Gtr1_RagA 1.5e-11, G-alpha 1.2e-08, Miro 3e-09
  TIC domains: Arf 5.1e-79, SRPRB 5.2e-15, Ras 2.4e-13, Gtr1_RagA 1.5e-11, G-alpha 1.2e-08, Miro 3e-09

```
UHR  AAACCACTGGAG  GATCAAGTCTTCCATACCAGGGACCAGGCCAAAACCAG
```

- SAR1A–TYSND1, -10:71600175..71569903 (distance: 30271 nt)
  Donor exon (score:0.997355):NM_001142648.exon1/8, NM_020150.exon1/7
  Acceptor exon (score:0.993018): NM_001040273.exon2/2, NM_173555.exon4/4
  Translation: tss-tle
  5′ gene domains: Arf 1.3e-63, SRPRB 1.1e-10, Ras 8.6e-08, Miro 5.4e-07
  3′ gene domains: Trypsin 4.3e-08
  TIC domains:

```
 N3  AGGAGCCCCTCAGGCC  GCATAATCACCAGCAAC
```

- ACCS–EXT2, +11:44055502..44085809 (distance: 30306 nt)
  Donor exon (score:0.999067):NM_001127219.exon7/15, NM_032592.exon7/15
  Acceptor exon (score:0.984107): NM_000401.exon4/16, NM_207122.exon2/14
  Translation: full,ptc
  5′ gene domains: Aminotran_1_2 2.1e-57
  3′ gene domains: Glyco_transf_64 3.7e-95, Exostosin 1.7e-49
  TIC domains: Aminotran_1_2 8e-12, Glyco_transf_64 6.2e-95, Exostosin 2.9e-49

```
HBR  GTCTACCTGGACAGTGAG  GAGTGTGAGGAAGAGGCTGTCTGTGTCATTAT
```

- GXYLT2–PPP4R2, +3:73099560..73129918 (distance: 30357 nt)
  Donor exon (score:0.998199):NM_001080393.exon6/7
  Acceptor exon (score:0.999138): NM_174907.exon2/9
  Translation: ptc
  5′ gene domains: Glyco_transf_8 8.8e-16
  3′ gene domains: PPP4R2 2.3e-87
  TIC domains: Glyco_transf_8 6.8e-16
  AceView: PPP4R2.fApr07
  Supporting ESTs: BE086819 (mammary gland neoplasia)

```
HBR  GAGCACTCTATGAAGCAATACGGGAT  ATTTTGAGAAGAGGGGGAAAAAGG
```

- GCSH–C16orf46, -16:79675569..79645200 (distance: 30368 nt)
  Donor exon (score:0.998367):NM_004483.exon4/5
  Acceptor exon (score:0.996115): NM_001100873.exon4/4
  Translation: tle
  5′ gene domains: GCV_H 4.9e-50
  3′ gene domains:
  TIC domains: GCV_H 9.9e-38

```
HBR  AACAAATCTTGTTATGAAGATG  CACAGGAAAAAGAAGATAAAGTAGGAGA
```

- SCAMP5–PPCDC, +15:73092199..73122835 (distance: 30635 nt)
  Donor exon (score:0.982837):NM_138967.exon3/7
  Acceptor exon (score:0.995459): NM_021823.exon3/6
  Translation: ptc
  5′ gene domains: SCAMP 2.1e-73
  3′ gene domains: Flavoprotein 9.8e-37
  TIC domains: SCAMP 7.4e-15

HBR ACCTCTGGATGT CTGGAAGTAGCAGTGGTCACAACTGAGAGAGCCAAACA

- TMC5–CP110, +16:19415986..19446690 (distance: 30703 nt)
  Donor exon (score:0.998387):NM_001105248.exon21/22, NM_024780.exon17/18, NM_001105249.exon20/21
  Acceptor exon (score:0.998850): NM_014711.exon3/15
  Translation: ptc
  5′ gene domains: TMC 1.2e-40
  3′ gene domains:
  TIC domains: TMC 1.1e-40

T1 GAACATGATGGCAGTCTTG TGTGACTGTGGGAA

- TAGLN2–CCDC19, -1:158161864..158129719 (distance: 32144 nt)
  Donor exon (score:0.998558):NM_003564.exon1/5
  Acceptor exon (score:0.999711): NM_012337.exon2/12
  Translation: tss
  5′ gene domains: CH 1e-16, Calponin 2.4e-13
  3′ gene domains:
  TIC domains:
  AceView: takerbo.aApr07
  Supporting ESTs: DA837969 (placenta normal), BX378374 (placenta normal), DA700400 (uncharacterized tissue neoplasia), DB038756 (testis normal)

T3 TCCAGCCCGCTTGAACG CCACTAAGCACAGCTG

- MYO5B–ACAA2, -18:45615712..45583221 (distance: 32490 nt)
  Donor exon (score:0.999388):NM_001080467.exon39/40
  Acceptor exon (score:0.998101): NM_006111.exon2/10
  Translation: ptc
  5′ gene domains: Myosin_head 5.9e-256, DIL 7.4e-38
  3′ gene domains: Thiolase_N 3.1e-100, Thiolase_C 4.4e-51
  TIC domains: Myosin_head 5.6e-256, DIL 7.2e-38

UHR GAACAATCCAG GTGTGTTTGTAGTTGCTGCTAAGCGAACGCCCTTTGGAG

- ASRGL1–SCGB1A1, +11:61913299..61946269 (distance: 32969 nt)
  Donor exon (score:0.992243):NM_001083926.exon5/7, NM_025080.exon5/7
  Acceptor exon (score:0.999154): NM_003357.exon2/3
  Translation: full
  5′ gene domains: Asparaginase_2 4.8e-73
  3′ gene domains: Uteroglobin 6.5e-32
  TIC domains: Asparaginase_2 1.4e-56, Uteroglobin 1.3e-19

HBR GCCGCGTTGGGGACTCACCGTGTCTAG CTTCTGCAGAGATCTGCCCGAGC

- ACCS–EXT2, +11:44052807..44085809 (distance: 33001 nt)
  Donor exon (score:0.995526):NM_001127219.exon5/15, NM_032592.exon5/15
  Acceptor exon (score:0.984107): NM_000401.exon4/16, NM_207122.exon2/14
  Translation: full,ptc
  5′ gene domains: Aminotran_1_2 2.1e-57
  3′ gene domains: Glyco_transf_64 3.7e-95, Exostosin 1.7e-49
  TIC domains: Glyco_transf_64 5.6e-95, Exostosin 2.6e-49

HBR GCAAGAGCCCAGTACCCCTCAGACCAGAGAAT GAGTGTGAGGAAGAGGCT

- PYCR2–LEFTY1, -1:224175531..224142355 (distance: 33175 nt)
  Donor exon (score:0.999643):NM_013328.exon5/6
  Acceptor exon (score:0.998943): NM_020997.exon2/4
  Translation: ptc
  5′ gene domains: F420_oxidored 1.7e-19
  3′ gene domains: TGFb_propeptide 7e-29, TGF_beta 8.3e-07
  TIC domains: F420_oxidored 1.2e-19

HBR GGCCTCCTGTATCCGAACACG AGGTGGCCGGCAGGTTCCTGGCGTTGGAG

- GCSH–C16orf46, -16:79687237..79653244 (distance: 33992 nt)
  Donor exon (score:0.998236):NM_004483.exon1/5
  Acceptor exon (score:0.998920): NM_001100873.exon3/4, NM_152337.exon4/4
  Translation: tle,ptc
  5′ gene domains: GCV_H 4.9e-50
  3′ gene domains:
  TIC domains:

N1 GCGCACTGGACCCGCTCTGCTCTCGG GTCCAAGGGTGGGGAAGGACTTCT

- PSMA4–CHRNA5, +15:76626095..76660208 (distance: 34112 nt)
  Donor exon (score:0.997806):NM_002789.exon8/9, NM_001102668.exon7/8, NM_001102667.exon8/9
  Acceptor exon (score:0.990406): NM_000745.exon2/6
  Translation: full
  5′ gene domains: Proteasome 3.9e-56, Proteasome_A_N 1.5e-14
  3′ gene domains: Neur_chan_LBD 3.2e-69, Neur_chan_memb 9.9e-43
  TIC domains: Proteasome 5.6e-53, Proteasome_A_N 4.4e-14, Neur_chan_LBD 6.3e-69, Neur_chan_memb 1.7e-42

  N1 GTTAGTAAACTCTCTGCTGAAAAAG GATTATCTGAACCTTCTTCTATTGCAAAACATGAAGATAGTTTGCTTAAG

- KLF16–REXO1, -19:1814040..1779630 (distance: 34409 nt)
  Donor exon (score:0.999388):NM_031918.exon1/2
  Acceptor exon (score:0.999622): NM_020695.exon2/16
  Translation: full
  5′ gene domains:
  3′ gene domains: Exonuc_X-T 1.2e-20
  TIC domains: Exonuc_X-T 1.4e-20

  HBR CACCTGCGGACGCACACAG GGCTGGGTTACGACCCCTACAACCCTGAGCT

- HSPB11–YIPF1, -1:54162165..54127724 (distance: 34440 nt)
  Donor exon (score:0.999237):NM_016126.exon5/6
  Acceptor exon (score:0.994718): NM_018982.exon2/11
  Translation: ptc
  5′ gene domains: F5_F8_type_C 8.4e-07
  3′ gene domains: Yip1 3e-21
  TIC domains: F5_F8_type_C 6.3e-06

  UHR AGCTTCAAAATGAAGAAATTGTG ATGAGGAAACTGAGGCCGCGAGGAGCa

- SLC35A3–HIAT1, +1:100253564..100288053 (distance: 34488 nt)
  Donor exon (score:0.998761):NM_012243.exon6/8
  Acceptor exon (score:0.988796): NM_033055.exon2/12
  Translation: full
  5′ gene domains: Nuc_sug_transp 3.7e-102, UAA 3e-08
  3′ gene domains: MFS_1 5.5e-33
  TIC domains: Nuc_sug_transp 3.3e-78, UAA 6.1e-06, MFS_1 1.2e-32

  T3 AGTTGTTCTTCAG CCTCAAGGAATAGGTTCTCC

- TMC5–CP110, +16:19415986..19451239 (distance: 35252 nt)
  Donor exon (score:0.998387):NM_001105248.exon21/22, NM_024780.exon17/18, NM_001105249.exon20/21
  Acceptor exon (score:0.996094): NM_014711.exon4/15
  Translation: ptc
  5′ gene domains: TMC 1.2e-40
  3′ gene domains:
  TIC domains: TMC 1.1e-40

  N1 GCTTTTTGCATTTGGGGGAACATGATGGCAGTCTTG CTTAACATTGAGAAAAGAAAGGAAATGCAACAAGAAAAG

- GPX7–FAM159A, +1:52845205..52881123 (distance: 35917 nt)
  Donor exon (score:0.998169):NM_015696.exon2/3
  Acceptor exon (score:0.995491): NM_001042693.exon2/3
  Translation: ptc
  5′ gene domains: GSHPx 2.6e-38
  3′ gene domains:
  TIC domains: GSHPx 1e-38

  HBR ATCCTGCCTTCAAGTACCTGGCCC CATTGGCGCTCTCATAGGCCTGTCCG

- C22orf39–HIRA, -22:17814901..17778301 (distance: 36599 nt)
  Donor exon (score:0.998181):NM_173793.exon2/3
  Acceptor exon (score:0.999625): NM_003325.exon2/25
  Translation: ptc
  5′ gene domains:
  3′ gene domains: Hira 6.9e-58, WD40 2.4e-09, HIRA_B 1.9e-08
  TIC domains:
  AceView: HIRA.jApr07
  Supporting ESTs: DC317105 (cerebrum normal), CN264339 (embryonic tissue normal), CD244790 (brain normal), DB475435 (cerebrum uncharacterized histology), DC318988 (brain normal), DC378060 (brain normal), DC309121 (cerebellum normal)

```
HBR  CGCCGGAACGCCGAGGCCCAG  GCAAGCCGATTTTTTCAGTTGATATTCAC
```

- SERF1B—SERF1A–SMN2, +5:69358098..69394998 (distance: 36899 nt)
  Donor exon (score:0.999150):NM_022978.exon2/3, NM_021967.exon2/3, NM_022968.exon2/3
  Acceptor exon (score:0.998017): NM_022876.exon2/8, NM_022877.exon2/7, NM_017411.exon2/9, NM_022875.exon2/8
  Translation: ptc
  5′ gene domains: 4F5 5.2e-16
  3′ gene domains: SMN 1.2e-100
  TIC domains: 4F5 6.4e-11

```
 T2           AGAAAGCAGAG  AGCGATGATTCTGACATTTGGG
HBR  GCCTCTCAGAGAAAGCAGAG  AGCGATGATTCTGACATTTGGGAgGATACA
```

- SMARCC1–CSPG5, -3:47626560..47589368 (distance: 37191 nt)
  Donor exon (score:0.999426):NM_003074.exon26/28
  Acceptor exon (score:0.996585): NM_006574.exon3/5
  Translation: ptc
  5′ gene domains: SWIRM 2.9e-32, Myb_DNA-binding 5.3e-09
  3′ gene domains: Chon_Sulph_att 2.5e-114, Neural_ProG_Cyt 4.9e-24
  TIC domains: SWIRM 2.7e-32, Myb_DNA-binding 4.9e-09

```
HBR  TGCACCACCAGATGCCACCACCTCATCCACCCCAGCCAG  GTGCAACACGC
```

- TSTD1–F11R, -1:159275294..159237767 (distance: 37526 nt)
  Donor exon (score:0.994976):NM_001113206.exon1/3, NM_001113205.exon1/3, NM_001113207.exon1/4
  Acceptor exon (score:0.999899): NM_016946.exon2/10
  Translation: full
  5′ gene domains: Rhodanese 1.6e-16
  3′ gene domains: I-set 3.4e-09, V-set 4.3e-11
  TIC domains: I-set 3e-09, V-set 3.8e-11
  AceView: F11R.pApr07
  Supporting ESTs: DC410017 (thymus uncharacterized histology), DB185348 (liver neoplasia), DC410686 (thymus uncharacterized histology), CN347865 (embryonic tissue normal), DC393602 (colon neoplasia), CX755127 (embryonic tissue normal), CR976629 (uncharacterized tissue normal), DC374044 (brain normal)

```
 T2       TGCTACGCGCACCATGGCTGGAG  GCTCCCTGGC
 N2  CGCGGTTGCTACGCGCACCATGGCTGGAG  GCTCCCTGGCATTGGGCAGTG
HBR                  CAgGGCTGGAG  GCTCCCTGGCATTGGGCAGTGTTACAGTGCACTCTTCTG
```

- SLC45A3–ELK4, -1:203897612..203859642 (distance: 37969 nt)
  Donor exon (score:0.992461):NM_033102.exon4/5
  Acceptor exon (score:0.998226): NM_001973.exon2/5, NM_021795.exon2/3
  Translation: full
  5′ gene domains: MFS_1 4.9e-11
  3′ gene domains: Ets 1e-37
  TIC domains: MFS_1 9.5e-11, Ets 2.9e-37

```
 T3  CCGGGAGAAGCAG  CTCATTGCTATGGACAGTGC
 T3    GGGAGAAGCAG  CTCATTGCTATGGACAGTGCTA
```

- FAM188B–AQP1, +7:30889133..30928206 (distance: 39072 nt)
  Donor exon (score:0.998175):NM_032222.exon17/18
  Acceptor exon (score:0.999822): NM_198098.exon2/4
  Translation: ptc
  5′ gene domains:
  3′ gene domains: MIP 5.1e-84
  TIC domains:

```
 N1                      CTCTGCATCAGAACCAA  CTGGCTGATGGTGTGAACTCGGGCCAGGGCCTGGGCATCGAGATCATCGGGACCCTCC
 N2  CGACCTTGTCCCACCCCTCGAGCTCTGCATCAGAACCAA  CTGGCTGATGG
```

- TPD52L2–DNAJC5, +20:61991035..62030132 (distance: 39096 nt)
  Donor exon (score:0.997806):NM_003288.exon6/7, NM_199361.exon7/8, NM_199359.exon5/6, NM_199363.exon6/7, NM_199362.exon7/8, NM_199360.exon8/9
  Acceptor exon (score:0.999518): NM_025219.exon2/5
  Translation: ptc
  5′ gene domains: TPD52 1.2e-52
  3′ gene domains: DnaJ 1.5e-26
  TIC domains: TPD52 2.8e-49

UHR TTTGAGGACCGAGTTGGGACCATAAAG AATAGCCTAACATGGCAGACCAG

- PDZD4–IDH3G, -X:152748888..152709500 (distance: 39387 nt)
  Donor exon (score:0.998583):NM_032512.exon1/8
  Acceptor exon (score:0.995340): NM_174869.exon2/12, NM_004135.exon2/13
  Translation: full
  5′ gene domains: PDZ 1.8e-14
  3′ gene domains: Iso_dh 2.1e-72
  TIC domains: Iso_dh 2e-72

HBR CAAAGTGATGCTGCAG GTTCTAGGCGCCCACGAGGTCCCCTCGAGGAACA

- TPD52–MRPS28, -8:81117410..81077970 (distance: 39439 nt)
  Donor exon (score:0.904539):NM_005079.exon5/6
  Acceptor exon (score:0.999137): NM_014018.exon2/3
  Translation: full
  5′ gene domains: TPD52 6.4e-74
  3′ gene domains: MRP-S35 9.6e-51
  TIC domains: TPD52 4.4e-63, MRP-S35 2.2e-50

N2 TTGAAGAAAAGGTCGAAAACTTAAAG GGTTCTCCAAAAAATGTGGAATCC

- LIPT1–MRPL30, +2:99137946..99177646 (distance: 39699 nt)
  Donor exon (score:0.997365):NM_145196.exon1/3, NM_145197.exon1/4, NM_015929.exon1/3, NM_145198.exon1/4, NM_145199.exon1/2
  Acceptor exon (score:0.999870): NM_145212.exon4/6
  Translation: tss-tle
  5′ gene domains: BPL_LplA_LipB 9.9e-22
  3′ gene domains: Ribosomal_L30 6.7e-10
  TIC domains:

N3 GGAAGCCGCAG GTGTTTCAGGCCTCACCTGAAG

- FXYD2–DSCAML1, -11:117196591..117156735 (distance: 39855 nt)
  Donor exon (score:0.998490):NM_001680.exon5/6, NM_021603.exon5/6
  Acceptor exon (score:0.999449): NM_020693.exon2/33
  Translation: ptc
  5′ gene domains: ATP1G1_PLM_MAT8 6.2e-27
  3′ gene domains: I-set 5.2e-16, fn3 3.4e-14, ig 1e-08
  TIC domains: ATP1G1_PLM_MAT8 6.2e-27

UHR ATCAATGAAGATGAGCCGTAACAGCAG CCCGCCCTGAAGATGTTGGCACC

- VPS45–PLEKHO1, +1:148349366..148389726 (distance: 40359 nt)
  Donor exon (score:0.996998):NM_007259.exon14/15
  Acceptor exon (score:0.987030): NM_016274.exon2/6
  Translation: ptc
  5′ gene domains: Sec1 3.8e-156
  3′ gene domains: PH 7.9e-11
  TIC domains: Sec1 1e-153

HBR ACAACACGAAAAG GGACCTCAGGATGGAAACCAGCAGCCTGCACCGCCCG

- LYPLA1–TCEA1, -8:55126125..55085605 (distance: 40519 nt)
  Donor exon (score:0.999196):NM_006330.exon8/9
  Acceptor exon (score:0.994448): NM_006756.exon2/10
  Translation: full
  5′ gene domains: Abhydrolase_2 3.8e-83
  3′ gene domains: TFIIS_M 2.1e-43, TFIIS_C 4e-19, Med26 8.5e-19
  TIC domains: Abhydrolase_2 5.6e-77, TFIIS_M 5.6e-43, TFIIS_C 7.9e-19, Med26 1.7e-18

UHR AAGGTATGATGCACAGTTCGTGTCAACAG GCTGGAGCATTGGATTTGCTA

- RPS6KA3–EIF1AX, -X:20105035..20063880 (distance: 41154 nt)
  Donor exon (score:0.984854):NM_004586.exon11/22
  Acceptor exon (score:0.998770): NM_001412.exon3/7
  Translation: full
  5′ gene domains: Pkinase 6e-72, Pkinase_Tyr 5.1e-42, Pkinase_C 1.1e-10
  3′ gene domains: eIF-1a 2.1e-27
  TIC domains: Pkinase 5e-72, Pkinase_Tyr 3.9e-44, eIF-1a 1e-24

```
 N2  GCTTTTCAAGCGAAATCCTGCAAACAGATTAG  AGTATGCTCAGGTAATCA
```

- PEX26–TUBA8, +22:16942780..16984246 (distance: 41465 nt)
  Donor exon (score:0.999237):NM_017929.exon3/6, NM_001127649.exon2/5
  Acceptor exon (score:0.999871): NM_018943.exon2/5
  Translation: ptc
  5′ gene domains: Pex26 5.1e-170
  3′ gene domains: Tubulin 8.3e-71, Tubulin_C 2e-50
  TIC domains: Pex26 7.3e-74
  AceView: PEX26andTUBA8.dApr07
  Supporting ESTs: BX407403 (brain normal), BX461656 (brain normal)

```
UHR  CAAAGTCCTGGAGCTGTG  CGGGAATGCATATCAGTCCACGTGGGCCAAGC
```

- GCSH–C16orf46, -16:79687237..79645200 (distance: 42036 nt)
  Donor exon (score:0.998236):NM_004483.exon1/5
  Acceptor exon (score:0.996115): NM_001100873.exon4/4
  Translation: tle
  5′ gene domains: GCV_H 4.9e-50
  3′ gene domains:
  TIC domains:

```
 T2           CCGCTCTGCTCTCGG  CACAGGAAAAAGAAGATA
HBR                  GCTCTCGG  CACAGGAAAAAGAAGATAAAGTAGGAGAGGGATgAGTTTCAC
UHR  CACTGGACCCGCTCTGCTCTCGG  CACAGGAAAAAGAAGATAAAGTAGGAG
```

- NPTXR–DNAL4, -22:37549034..37506960 (distance: 42073 nt)
  Donor exon (score:0.999539):NM_014293.exon4/5
  Acceptor exon (score:0.995564): NM_005740.exon3/4
  Translation: full
  5′ gene domains: Pentaxin 7.2e-33
  3′ gene domains: Dynein_light 9.8e-29
  TIC domains: Pentaxin 8.2e-13, Dynein_light 2.2e-26

```
UHR  TATCTTGGGCCAGGAGCAG  CACTCGGACATGCCAGAGGAGATGCGCGTGG
```

- IVD–BAHD1, +15:38495853..38537942 (distance: 42088 nt)
  Donor exon (score:0.998387):NM_002225.exon11/12
  Acceptor exon (score:0.999405): NM_014952.exon2/7
  Translation: full
  5′ gene domains: Acyl-CoA_dh_1 1.7e-41, Acyl-CoA_dh_N 1.6e-36, Acyl-CoA_dh_M 1.9e-18, Acyl-CoA_dh_2 4.6e-09
  3′ gene domains: BAH 9.1e-14
  TIC domains: Acyl-CoA_dh_N 7.5e-36, Acyl-CoA_dh_1 9.4e-25, Acyl-CoA_dh_M 7e-18, BAH 1.7e-13

```
 N1  AGCTGAGTGTGCCACACAGGTAGCCCTGGACGGCATTCAGTGTTTTG  GTTGGAAGTACTCCATGACACACACTCG
```

- FBXO46–SNRPD2, -19:50925947..50883664 (distance: 42282 nt)
  Donor exon (score:0.993368):NM_001080469.exon1/2
  Acceptor exon (score:0.998418): NM_177542.exon3/4, NM_004597.exon2/3
  Translation: tss
  5′ gene domains:
  3′ gene domains: LSM 4.1e-18
  TIC domains: LSM 3e-18

```
 T1                                    CGGCGATG  GAGCCTCCTCAACAAGCCCAAGAGT
 T2                                  CGCGGCGATG  GAGCCTCCTCAACAAGCCCAAGA
UHR  GGGAAACCCGCGTAGCCGAGGGAGATGCAGCGCGCGGCGATG  GAGCCTCC
```

- SYS1–DBNDD2, +20:43427740..43470514 (distance: 42773 nt)
  Donor exon (score:0.999088):NM_033542.exon3/4
  Acceptor exon (score:0.999696): NM_001048224.exon2/4, NM_001048221.exon2/4, NM_001048223.exon2/4, NM_001048222.exon2/4
  Translation: ptc
  5′ gene domains: SYS1 6.3e-57
  3′ gene domains: Dysbindin 2.5e-38
  TIC domains: SYS1 1.7e-19
  AceView: DBNDD2.jApr07
  Supporting ESTs: DN994280 (brain normal), BM721587 (eye normal), Z44280 (brain normal), BI758351 (brain normal), BI199175 (brain neoplasia), BE737467 (pancreas neoplasia), AI929037 (cerebrum normal)

```
 N1  CCGGCTCTCaATGATGTCCTTCATCCTCAACGCCCTCACCTG  GAGCTGAC
 N2                              AACGCCCTCACCTG  GAGCTGACATGGACCCAAATCCTCGGGCCGCCCTGG
HBR   CGGCTCTCCATGATGTCCTTCATCCTCAACGCCCTCACCTG  GAGCTGACA
HBR      CTCTCCATGATGTCCTTCATCCTCAACGCCCTCACCTG  GAGCTGACATGG
HBR        CTCCATGATGTCCTTCATCCTCAACGCCCTCACCTG  GAGCTGACATGGAC
HBR              GATGTCCTTCATCCTCAACGCCCTCACCTG  GAGCTGACATGGACCCAAAT
HBR                         TCCTCAACGCCCTCACCTG  GAGCTGACATGGACCCAAATCCTCGGGCCGC
HBR                             CAACGCCCTCACCTG  GAGCTGACATGGACCCAAATCCTCGGGCCGCCCTG
HBR                                    CTCACCTG  GAGCTGACATGGACCCAAATCCTCGGGCCGCCCTGGAGCGCC
UHR  CCGGCTCTCCATGATGTCCTTCATCCTCAACGCCCTCACCTG  GAGCTGAC
```

- DYX1C1–CCPG1, -15:53511987..53468923 (distance: 43063 nt)
  Donor exon (score:0.999316):NM_130810.exon9/10
  Acceptor exon (score:0.995639): NM_004748.exon2/8, NM_020739.exon2/8
  Translation: ptc
  5′ gene domains: CS 9.4e-14
  3′ gene domains:
  TIC domains: CS 8.3e-14
  AceView: DYX1C1andCCPG1.aApr07

```
 N2  CAGCATTCTGTCAACTAGAATTGTATGTAGAAG  ACCTGAAAGATGTCTGA
```

- VDAC1–C5orf15, -5:133368237..133323610 (distance: 44626 nt)
  Donor exon (score:0.998039):NM_003374.exon1/9
  Acceptor exon (score:0.999140): NM_020199.exon2/3
  Translation: tss
  5′ gene domains: Porin_3 2.4e-76
  3′ gene domains:
  TIC domains:

```
UHR  CcCCGAGCCCGCCGCCACATCCTCTGAG  TTGTATCACGGACTGATTCACC
```

- FEN1–FADS2, +11:61317076..61361826 (distance: 44749 nt)
  Donor exon (score:0.998325):NM_004111.exon1/2
  Acceptor exon (score:0.997464): NM_004265.exon2/12
  Translation: tss
  5′ gene domains: XPG_N 6.1e-38, XPG_I 2.4e-29, 5_3_exonuc 2.9e-07
  3′ gene domains: FA_desaturase 1.5e-38, Cyt-b5 3.2e-23
  TIC domains: FA_desaturase 5.4e-39

```
 N2  CGCCGAGGCCGCGTGTCCCAAAGGCCAG  GATGCCTTCCGCGCCTTCCACC
UHR                   CCAAAGGCCAG  GATGCCTTCCGCGCCTTCCACCCTGACCTGGAATTCGTG
```

- MLYCD–OSGIN1, +16:82503473..82548746 (distance: 45272 nt)
  Donor exon (score:0.998942):NM_012213.exon4/5
  Acceptor exon (score:0.999743): NM_182981.exon2/6, NM_013370.exon3/7, NM_182980.exon2/6
  Translation: ptc
  5′ gene domains: MCD 3.8e-92
  3′ gene domains:
  TIC domains: MCD 1.2e-45

```
 N1                          GTCAAGGAGTTGCAG  GcCCGCTGCCAGCCCCAAGCCCCCCACCAGCCATGAGCTCCTCCAGAAAGGACCACCTCG
 N2  GGAACATTCCTCATAAAGCGAGTCGTCAAGGAGTTGCAG  GTCCGCTGCCA
```

- FAM47E–STBD1, +4:77404020..77449321 (distance: 45300 nt)
  Donor exon (score:0.998521):NM_001136570.exon3/8
  Acceptor exon (score:0.998868): NM_003943.exon2/2
  Translation: tle
  5′ gene domains:
  3′ gene domains: CBM_20 4.9e-19
  TIC domains:
  AceView: gergaw.aApr07
  Supporting ESTs: DA281661 (cerebrum normal), BP259782 (heart uncharacterized histology)

```
HBR  CTTTTAAAAAAAACATTCTACCCAAGTCTACCTGGGACC  AGCATCTTCAAG
```

- SRP9–EPHX1, +1:224037693..224083049 (distance: 45355 nt)
  Donor exon (score:0.996827):NM_003133.exon2/3, NM_001130440.exon2/4
  Acceptor exon (score:0.999323): NM_000120.exon2/9, NM_001136018.exon2/9

Translation: ptc
5′ gene domains: SRP9-21 7.2e-17
3′ gene domains: EHN 4.3e-35, Abhydrolase_1 1.1e-14
TIC domains: SRP9-21 2.5e-14
AceView: SRP9andEPHX1.eApr07
Supporting ESTs: DA417873 (brain normal), DA175843 (cerebrum normal)

`N2 ATGATTTAGTT GAGCCATGTGGCTAGAAATCCTCCTCACTTCAGTGCTGG`

- KLK11–KLK7, -19:56218160..56172759 (distance: 45400 nt)
  Donor exon (score:0.997970):NM_001136032.exon5/6, NM_144947.exon5/6, NM_006853.exon5/6
  Acceptor exon (score:0.998896): NM_139277.exon6/6, NM_005046.exon6/6
  Translation: full
  5′ gene domains: Trypsin 3.5e-70
  3′ gene domains: Trypsin 2.9e-68
  TIC domains: Trypsin 6e-69, Trypsin 6e-69

  `T3 GGGGGCAAtGACTCCTGCCAG GGTGACTCAGGG`

- TPD52L2–DNAJC5, +20:61984617..62030132 (distance: 45514 nt)
  Donor exon (score:0.995068):NM_003288.exon5/7, NM_199361.exon4/8, NM_199359.exon4/6, NM_199363.exon4/7, NM_199362.exon5/8, NM_199360.exon5/9
  Acceptor exon (score:0.999518): NM_025219.exon2/5
  Translation: ptc
  5′ gene domains: TPD52 1.2e-52
  3′ gene domains: DnaJ 1.5e-26
  TIC domains: TPD52 1e-40
  AceView: TPD52L2andDNAJC5.vdApr07
  Supporting ESTs: BP313230 (mammary gland neoplasia)

  `N2 CTTGGAGACATGAG AATAGCCTAACATGGCAGACCAGAGACAGCtCTCAC`

- SAR1A–AIFM2, -10:71600175..71553859 (distance: 46315 nt)
  Donor exon (score:0.997355):NM_001142648.exon1/8, NM_020150.exon1/7
  Acceptor exon (score:0.996077): NM_032797.exon2/9
  Translation: tss
  5′ gene domains: Arf 1.3e-63, SRPRB 1.1e-10, Ras 8.6e-08, Miro 5.4e-07
  3′ gene domains: Pyr_redox_2 1.8e-25, Pyr_redox 7.6e-09
  TIC domains: Pyr_redox_2 1.8e-25, Pyr_redox 7.6e-09
  AceView: SAR1AandTYSND1andAIFM2.rApr07
  Supporting ESTs: DA739481 (testis neoplasia), DB266594 (uterus uncharacterized histology)

  `UHR GAGCCCCTCAGGCC TGCCTGATTTGAGATGGGGTCCCAGGTCTCGGTGGA`

- C13orf38–SOHLH2, -13:35720743..35674230 (distance: 46512 nt)
  Donor exon (score:0.999414):NM_001144981.exon7/8, NM_001144982.exon6/7, NM_001144983.exon6/7, NM_001144984.exon5/6, NM_001144985.exon6/7, NM_001144986.exon6/7
  Acceptor exon (score:0.999836): NM_017826.exon2/11
  Translation: ptc
  5′ gene domains:
  3′ gene domains: HLH 1.2e-09
  TIC domains:
  AceView: SOHLH2.oApr07
  Supporting ESTs: DC401054 (testis uncharacterized histology), CB961415 (placenta normal), BI560024 (testis normal), BJ995886 (liver neoplasia), DB460883 (testis uncharacterized histology), BG722947 (testis normal), BI465027 (testis normal), AL707893 (muscle normal), AW961069 (uncharacterized tissue uncharacterized histology), BI561236 (testis normal), AW961078 (uncharacterized tissue uncharacterized histology)

  `UHR GAATCTAGTGAAAAC GCAAAAATAGACATCTTATTAGTTGGAGATGTCAC`

- SDHAF2–C11orf66, +11:60962161..61008736 (distance: 46574 nt)
  Donor exon (score:0.998107):NM_017841.exon3/4
  Acceptor exon (score:0.998877): NM_145017.exon5/14
  Translation: ptc
  5′ gene domains: Sdh5 2.2e-18
  3′ gene domains:
  TIC domains: Sdh5 1.7e-18
  AceView: C11orf79andC11orf66.fApr07
  Supporting ESTs: DA106900 (cerebellum normal), DA154051 (cerebrum normal)

```
HBR  GGGATATTTACTACTGGGCCACAG  GTCCGGAAGGTCCATTTCGACACCCA
HBR        TTTACTACTGGGCCACAG  GTCCGGAAGGTCCATTTCGACACCCAGGAGCA
UHR         TTACTACTGGGCCACAG  GTCCGGAAGGTCCATTTCGACACCCAGGAGCAC
```

- SFRS18–COQ3, -6:99979812..99932082 (distance: 47729 nt)
  Donor exon (score:0.999173):NM_015491.exon1/11, NM_032870.exon1/12
  Acceptor exon (score:0.932494): NM_017421.exon4/7
  Translation: tss
  5′ gene domains:
  3′ gene domains: Methyltransf_11 8.5e-20, Methyltransf_12 3.2e-13, CMAS 4.4e-10, Ubie_methyltran 1.8e-07
  TIC domains: Methyltransf_11 2.9e-20, Methyltransf_12 1.1e-13, CMAS 1.5e-09, Ubie_methyltran 1.2e-06

```
N1  CGACAAGGGTGTTCCCGAACTAGAGTGG  GGACAATCTTCTGAAAACAATT
```

- DPAGT1–HYOU1, -11:118473062..118425125 (distance: 47936 nt)
  Donor exon (score:0.999069):NM_001382.exon8/9
  Acceptor exon (score:0.994882): NM_006389.exon17/26, NM_001130991.exon17/26
  Translation: full
  5′ gene domains: Glycos_transf_4 7e-36
  3′ gene domains: HSP70 3.9e-100
  TIC domains: Glycos_transf_4 2.5e-35, HSP70 0.00039

```
T2  GCTgCTGCTGCTGCTGCAG  GAGGAAGAGGAGAG
```

- AZGP1–GJC3, -7:99407305..99359162 (distance: 48142 nt)
  Donor exon (score:0.996163):NM_001185.exon2/4
  Acceptor exon (score:0.966875): NM_181538.exon2/2
  Translation: full
  5′ gene domains: MHC_I 8.9e-68, C1-set 1.1e-17
  3′ gene domains: Connexin 4.5e-32, Connexin_CCC 2.5e-24
  TIC domains: MHC_I 8.5e-29
  AceView: AZGP1andGJE1.fApr07
  Supporting ESTs: AA449344 (embryonic tissue normal), BE839154 (prostate normal), AI806273 (pooled tissue normal), AI918871 (prostate normal), T64965 (pooled tissue normal), AA449603 (embryonic tissue normal)

```
T1                                       GGAGTATTACAACGACAGTAACG  TTCCAGGAAG
T1                                        GAGTATTACAACGACAGTAACG  TTCCAGGAAGA
T2                                                   CGACAGTAACG  TTCCAGGAAGAAGCTTAGCCCA
T2                                                    GACAGTAACG  TTCCAGGAAGAAGCTTAGCCCAG
T2                                                    GACAGTAACG  TTCCAGGAAtAAGCTTAGCCCAG
T3                                            ATTACAACGACAGTAACG  TTCCAGGAAGAAGCT
T3                                                    GACAGTAACG  TTCCAGGAAGAAGCTTAGCCCAG
N1  GGAGGACATCTTTATGGAGACCCTGAAAGACATCGTGGAGTATTACAACGACAGTAACG  TTCCAGGAcGAAGCTT
N1     GGACATCTTTATGGAGACCCTGAAAGACATCGTGGAGTATTACAACGACAGTAACG  TTCCAGGAAGAAGCTTAGC
N1                          TGAAAGACATCGTGGAGTATTACAACGACAGTAACG  TTCCAGGAAGAAGCTTAGCCCAGGAAAAACAAAGACCAG
N1                                ACATCGTGGAGTATTACAACGACAGTAACG  TTCCAGGAAGAAGCTTAGCCCAGGAAAAACAAAGACCAGTTGGAC
N1                                  ATCGTGGAGTATTACAACGACAGTAACG  TTCCAGGAAGAAGCTTAGCCCA
N1                                  ATCGTGGAGTATTACAACGACAGTAACG  TTCCAGGAAGAAGCTTAGCCCAGGAAAAACAAAGACCAGTTGGACCC
N1                                   TCGTGGAGTATTgCAACGACAGTAACG  TTCCAGGAAGAAGCTTAGCCCAGGAAAAACAAAGACCAGTTGGACCCA
N1                                     GcGGAGTATTACAACGACAGTAACG  TTCCAGGAAGAAGCTTAGCCCAGGA
N1                                     GTGGAGTATTACAACGACAGTAACG  TTCCAGGAAGAAGCTTAGCCCAGGAAAAACAAAGACCAGTTGGACCCAGA
N1                                        GAGTATTACAACGACAGTAACG  TTCnAGGAAGAAGCTTAGCCCAGGAAAA
N1                                        GAGTATTACAACGACAGTAACG  TTCCAGGAAGAAGCTTAGCCCAGGAAAAACAAAGACCAGTTGGACCCAGAGAT
N1                                          GTATTACAACGACAGTAACG  TTCCAGGAAGAAGCTTAGCCCAGGAAAAAC
N1                                             TTACAACGACAGTAACG  TTCCAGGAAGAAGCTTAGCCCAGGAAAAACAAAGACCAGTTGGACCCAGAGAgGCCTG
N1                                                   CGACAGTAACG  TTCCAGGAAGAAGCTTAGCCCAGGAAAAACAAAGACCAGTTGGACCCAGAGATGCCTGA[illegible]
N1                                                    GACAGTAACG  TTCCAGGAAGAAGCTTAGCCCAGGAAAAACAAAGACCAGTTGGACCCAGAGATGCCTGA[illegible]
N2                           GAAAGACATCGTGGAGTATTACAACGACAGTAACG  TTCCAGGAAGAAGCT
N2                             AAGACATCGTGGAGTATTACAACGACAGTAACG  TTCCAGGAAGAAGCTTA
N2                                  ATCGTGGAGTATTACAACGACAGTAACG  TTCCAGGAAGAAGCTTAGCCCA
N2                                     GTGGAGTATTACAACGACAGTAACG  TTCCAGGAAGAAGCTTAGCCCAGGA
N2                                     GTGGAGTATTACAACGACAGTAACG  TTCCAGGAAGAAGCTTAGCCCAGGA
N2                                      TGGAGTATTACAACGACAGTAACG  TTCCAGGAAGAAGCTTAGCCCAGGAA
N2                                       GGAGTATTACAACGACAGTAACG  TTCCAGGAAGAAGCTTAGCCCAGGAAA
N2                                       GGAGTATTACAACGACAGTAACG  TTCCAGGAAGAAGCTTAGCCCAGGAAA
N2                                        GAGTATTACAACGACAGTAACG  TTCCAGGAAGAAGCTTAGCCCAGGAAAA
N2                                        GAGTATTACAACGACAGTAACG  TTCCAGtAAGAAGCTTAGCCCAGGAAAA
```

```
 N2         GTATTACAACGACAGTAACG  TTCCAGGAAGAAGCTTAGCCCAGGAAAAAC
 N2            TTACAACGACAGTAACG  TTCCAGGAAGAAGCTTAGCCCAGGAAAAACAAA
 N2                  CGACAGTAACG  TTCCAGGAAGAAGCTTAGCCCAGGAAAAACAAAGACCAG
 N2                  CGACAGTAACG  TTCCAGGAAGAAGCTTAGCCCAGGAAAAACAAAGACCAG
 N2                     CAGTAACG  TTCCAGGAAGAAGCTTAGCCCAGGAAAAACAAAGACCAGTTG
 N3    GTGGAGTATTACAACGACAGTAACG  TTCCAGGA
 N3              ACAACGACAGTAACG  TTCCAGGAAGAAGCTTAG
 N3                     CAGTAACG  TTCCAGGAAGAAGCTTAGCCCAGGA
HBR ATCGTGGAGTATTACAACGACAGTAACG  TTCCAGGAAGAAGCTTAGCCCg
```

- BIK–TSPO, +22:41836753..41885160 (distance: 48406 nt)
  Donor exon (score:0.999388):NM_001197.exon1/5
  Acceptor exon (score:0.999738): NM_000714.exon2/4
  Translation: tss
  5′ gene domains: bcl-2I13 8.1e-94
  3′ gene domains: TspO_MBR 4.3e-45
  TIC domains: TspO_MBR 4.3e-45

```
 T3  CGCCGCCGCCAG  AGCTCCCCTGAACAGCAGCTG
```

- C11orf73–CCDC81, +11:85726220..85774741 (distance: 48520 nt)
  Donor exon (score:0.998551):NM_016401.exon3/5
  Acceptor exon (score:0.983118): NM_021827.exon2/14
  Translation: ptc
  5′ gene domains: DUF775 2.7e-55
  3′ gene domains:
  TIC domains: DUF775 6e-37

```
UHR  TGTATCCTCAGTTGACTCATTCACTCAG  AAGTCTCTATTATCTGGGGGAA
```

- STAG3L2–GTF2IRD2, -7:73938186..73889441 (distance: 48744 nt)
  Donor exon (score:0.988565):NM_001025202.exon6/8
  Acceptor exon (score:0.996912): NM_173537.exon2/16
  Translation: ptc
  5′ gene domains:
  3′ gene domains: GTF2I 4.4e-31
  TIC domains:

```
 T3  GATTACTGATACTTATCCTTAA  GGATCATGGCC
```

- HDAC8–CITED1, -X:71488308..71439509 (distance: 48798 nt)
  Donor exon (score:0.998710):NM_018486.exon10/11
  Acceptor exon (score:0.999242): NM_004143.exon2/3, NM_001144885.exon2/3, NM_001144886.exon2/3
  Translation: full,ptc
  5′ gene domains: Hist_deacetyl 4.7e-83
  3′ gene domains: CITED 3.6e-61
  TIC domains: Hist_deacetyl 1.7e-82, CITED 8.3e-60

```
 T3         CCTCAACTACATCAAAG  CACAACAGCTCCAGCT
 T3          CTCAACTACATCAAAG  CACAACAGCTCCAGCTG
 N1  AACAAATCCTCAACTACATCAAAG  CACAACAGCTCCAGCTGGCAGCATCACTTCCCGCCAATTTATCCAACTTCT
UHR       ATCCTCAACTACATCAAAG  CACAACAGCTCCAGCTGGCAGCATCACTTCC
```

- TOPORS–DDX58, -9:32540772..32490937 (distance: 49834 nt)
  Donor exon (score:0.999046):NM_005802.exon2/3
  Acceptor exon (score:0.996434): NM_014314.exon2/18
  Translation: ptc
  5′ gene domains: zf-C3HC4 7.2e-07
  3′ gene domains: RIG-I_C-RD 1.8e-38, DEAD 1.2e-16, ResIII 2.7e-13, Helicase_C 4.5e-12
  TIC domains:
  AceView: TOPORSandDDX58.oApr07
  Supporting ESTs: BG180462 (prostate neoplasia), DA483819 (brain normal), DA312581 (cerebrum normal), DB237523 (uncharacterized tissue uncharacterized histology), BP363837 (lung uncharacterized histology), DA227957 (brain normal), DB134474 (thymus uncharacterized histology), DB291052 (uterus uncharacterized histology), DR003655 (cerebrum normal), DA506306 (brain normal), DA394381 (brain normal), DA743129 (testis neoplasia)

```
 N1  CCCAGCCAGGCCTGCGCCGGCATCCTCCGAG  AAGAGGTGCAGTATATTCAGGCTGAGAAAAACAACAAGGGCCCA
HBR               GCGCCGGCATCCTCCGAG  AAGAGGTGCAGTATATTCAGGCTGAGAAAAAC
```

- FKBP1A–SDCBP2, -20:1300720..1249079 (distance: 51640 nt)
  Donor exon (score:0.998158):NM_000801.exon4/5
  Acceptor exon (score:0.996204): NM_080489.exon2/9
  Translation: ptc
  $5'$ gene domains: FKBP_C 1.4e-34
  $3'$ gene domains: PDZ 5.2e-09
  TIC domains: FKBP_C 1.4e-34
  Supporting ESTs: DA979338 (synovium uncharacterized histology)

```
 N1  GAATGACAGGAATGGCCTCCTCCCTTAGCTCCCTGTTCTTGG  GGTGTTCTGAGAATCAGCCATGTCATCCCTGTA
HBR                          CCCTTAGCTCCCTGTTCTTGG  GGTGTTCTGAGAATCAGCCATGTCATCCC
```

- KIAA0753–PITPNM3, -17:6433823..6382126 (distance: 51696 nt)
  Donor exon (score:0.997565):NM_014804.exon18/19
  Acceptor exon (score:0.999238): NM_031220.exon2/20
  Translation: ptc
  $5'$ gene domains:
  $3'$ gene domains: DDHD 3.2e-48, LNS2 6e-39
  TIC domains:

```
UHR  GGCTGTAGGCTCCTTCAACCCGTGGCTGATAGCTGAAAG  GTGGTCCTCCC
```

- PPP2CA–SKP1, -5:133589350..133537612 (distance: 51737 nt)
  Donor exon (score:0.998710):NM_002715.exon1/7
  Acceptor exon (score:0.999354): NM_170679.exon2/6, NM_006930.exon2/5
  Translation: full
  $5'$ gene domains: Metallophos 2.8e-42
  $3'$ gene domains: Skp1 1.6e-35, Skp1_POZ 2.7e-30
  TIC domains: Skp1 2.5e-35, Skp1_POZ 4.2e-30
  AceView: PPP2CAandSKP1A.pApr07
  Supporting ESTs: DA615657 (brain neoplasia)

```
 T3                  AAGAGCCTCTGCGAGAAG  ATGCCTTCAATTAAG
 N1  CCGAGTCCCAGGTCAAGAGCCTCTGCGAGAAG  ATGCCTTCAATTAAGTTG
```

- NCK1–IL20RB, +3:138129759..138181998 (distance: 52238 nt)
  Donor exon (score:0.998235):NM_006153.exon2/4
  Acceptor exon (score:0.981802): NM_144717.exon2/7
  Translation: full
  $5'$ gene domains: SH3_1 2.3e-15, SH3_2 1.3e-11, SH2 2.2e-23
  $3'$ gene domains: Tissue_fac 9.8e-25
  TIC domains: SH3_1 1.1e-13, SH3_2 1e-09, Tissue_fac 4.4e-23

```
HBR  GGAAAGCATCTATTGTGAAAAACCTAAAGGATACCTTAG  ATGAAGTGGCC
```

- ADAM23–LOC200726, +2:207169131..207221630 (distance: 52498 nt)
  Donor exon (score:0.912280):NM_003812.exon24/26
  Acceptor exon (score:0.999760): NM_001102659.exon3/3
  Translation: full
  $5'$ gene domains: Reprolysin 5.2e-58, Pep_M12B_propep 3.2e-39, ADAM_CR 6.9e-30, Disintegrin 1.8e-18
  $3'$ gene domains:
  TIC domains: Reprolysin 5.2e-58, Pep_M12B_propep 3.2e-39, ADAM_CR 6.9e-30, Disintegrin 1.8e-18

```
HBR  GAACCTTCACCCCCCCAAGGATGAAGGACCCAAGG  GTACATATTCTCAGC
```

- RPL11–TCEB3, +1:23894985..23948098 (distance: 53112 nt)
  Donor exon (score:0.998135):NM_000975.exon5/6
  Acceptor exon (score:0.998519): NM_003198.exon2/11
  Translation: full
  $5'$ gene domains: Ribosomal_L5_C 4.7e-23, Ribosomal_L5 2e-19
  $3'$ gene domains: Elongin_A 1e-36, Med26 4.6e-17
  TIC domains: Ribosomal_L5_C 1e-21, Ribosomal_L5 2.4e-18, Elongin_A 1.3e-36, Med26 5.5e-17
  AceView: RPL11andTCEB3.fApr07
  Supporting ESTs: BP236096 (vascular uncharacterized histology), DA660101 (kidney normal)

```
HBR  GCTGGTTCCAGCAGAAG  CTATTGAAATATTTGAAGAAACTCTCCACCCTG
```

- MAP2K5–LBXCOR1, +15:65852144..65905328 (distance: 53183 nt)
  Donor exon (score:0.999516):NM_002757.exon20/21, NM_145160.exon21/22
  Acceptor exon (score:0.998941): NM_001031807.exon2/10

Translation: ptc
5′ gene domains: Pkinase 2.5e-59, Pkinase_Tyr 1.5e-38, PB1 1.8e-11
3′ gene domains: Ski_Sno 1.5e-39, c-SKI_SMAD_bind 1.6e-38
TIC domains: Pkinase 7e-58, Pkinase_Tyr 1.2e-38, PB1 2.1e-11

```
HBR  GAAGAATTGATG  GAGCGGCGGCATGGAGGCTCTCACCACTCAGCTGGGGC
```

- PLEKHO2–ANKDD1A, +15:62940827..62995049 (distance: 54221 nt)
  Donor exon (score:0.995574):NM_025201.exon5/6
  Acceptor exon (score:0.999719): NM_182703.exon2/15
  Translation: ptc
  5′ gene domains: PH 3.5e-10
  3′ gene domains: Death 3.5e-12
  TIC domains: PH 4.5e-11

```
HBR  GCGACGCCGGCCACCAACGAGAGTCCACCTGAAGGAG  TGCTTCCTCTGGA
HBR                          AGTCCACCTGAAGGAG  TGCTTCCTCTGGAGAGGCAGCTCCACGAGGCCGC
```

- RPL11–TCEB3, +1:23893868..23948098 (distance: 54229 nt)
  Donor exon (score:0.974707):NM_000975.exon4/6
  Acceptor exon (score:0.998519): NM_003198.exon2/11
  Translation: full
  5′ gene domains: Ribosomal_L5_C 4.7e-23, Ribosomal_L5 2e-19
  3′ gene domains: Elongin_A 1e-36, Med26 4.6e-17
  TIC domains: Ribosomal_L5_C 5.7e-20, Ribosomal_L5 2.3e-18, Elongin_A 1.2e-36, Med26 5.2e-17
  AceView: RPL11andTCEB3.eApr07
  Supporting ESTs: DA864433 (placenta normal), BP280989 (cerebrum neoplasia)

```
UHR  CTGGACTTCTATGTG  CTATTGAAATATTTGAAGAAACTCTCCACCCTGCC
```

- SDHAF2–C11orf66, +11:60954230..61008736 (distance: 54505 nt)
  Donor exon (score:0.996831):NM_017841.exon1/4
  Acceptor exon (score:0.998877): NM_145017.exon5/14
  Translation: full
  5′ gene domains: Sdh5 2.2e-18
  3′ gene domains:
  TIC domains:

```
 T2            TGTTCTCGACTTCGTCGCTG  GTCCGGAAGGTCC
 N1  TGTCTACAGTGTTCTCGACTTCGTCGCTG  GTCCGGAAGGTCCATTTCGAC
UHR  TGTCTACAGTGTTCTCGACTTCGTCGCTG  GTCCGGAAGGTCCATTTCGAC
```

- POLR2J2–RASA4, -7:102093760..102038652 (distance: 55107 nt)
  Donor exon (score:0.995936):NM_032959.exon4/9
  Acceptor exon (score:0.999467): NM_001079877.exon2/20, NM_006989.exon2/21
  Translation: ptc
  5′ gene domains: RNA_pol_L 1.7e-08
  3′ gene domains: RasGAP 6.4e-46, C2 6e-22, BTK 5.4e-13
  TIC domains: RNA_pol_L 1.7e-08
  Supporting ESTs: DA474156 (skin normal), DC322362 (brain normal)

```
 N1  CTCCCCAGCCACGCTTCCTGTACAGCCTGCAGAACT  CACTGGCAGCAGCGACCCCTACTGCATCGTGAAGGTGGA
UHR                            CTGCAGAACT  CACTGGCAGCAGCGACCCCTACTGCATCGTGAAGGTGGAC
```

- C9orf30–TMEFF1, +9:102244459..102300868 (distance: 56408 nt)
  Donor exon (score:0.996904):NM_080655.exon2/3
  Acceptor exon (score:0.999669): NM_003692.exon2/10
  Translation: full
  5′ gene domains:
  3′ gene domains: Kazal_2 5.3e-11, Kazal_1 9.6e-09
  TIC domains: Kazal_2 6.7e-11, Kazal_1 6.1e-09
  AceView: C9orf30andTMEFF1.aApr07
  Supporting ESTs: DN994340 (brain normal), DA294795 (cerebrum normal), BG168238 (kidney neoplasia), DA293094 (cerebrum normal), DN991602 (brain normal)

```
 T3                        CCTCAGCCCAAG  AATTAAATGTGAGGGAGTCTG
 N2                         CTCAGCCCAAG  AATTAAATGTGAGGGAGTCTGACGTAAGAGTTTGTGATG
 N2                         CTCAGCCCAAG  AATTAAATGTGAGGGAGTCTGACGTAAGAGTTTGTGATG
HBR  GCCCGAATACCACCCCGACGCCTCAGCCCAAG  AATTAAATGTGAGGGAGT
```

```
HBR        AATACCACCCCGACGCCTCAGCCCAAG  AATTAAATGTGAGGGAGTCTGAC
HBR         ATACCcCCCCGACGCCTCAGCCCAAG  AATTAAATGTGAGGGAGTCTGACG
HBR          TACCACCCCGACGCCTCAGCCCAAG  AATTAAATGTGAGGGAGTCTGACGT
UHR    CCGAATACCACCCCGACGCCTCAGCCCAAG  AATTAAATGTGAGGGAGTCT
UHR     CGAATACCACCCCGACGCCTCAGCCCAAG  AATTAAATGTGAGGGAcTCTG
```

- BICD2–IPPK, -9:94517356..94460784 (distance: 56571 nt)
  Donor exon (score:0.999072):NM_015250.exon7/8
  Acceptor exon (score:0.999108): NM_022755.exon2/13
  Translation: full
  5′ gene domains: BicD 0
  3′ gene domains: Ins_P5_2-kin 2.2e-102
  TIC domains: BicD 0, Ins_P5_2-kin 9.3e-95

```
 N2  CGAAGACCAAGCCAGCCACACCGAGC  CGCTGCGTCGTGCTGCGGTTTCTG
```

- POLR2J2–RASA4, -7:102093760..102036964 (distance: 56795 nt)
  Donor exon (score:0.995936):NM_032959.exon4/9
  Acceptor exon (score:0.999087): NM_001079877.exon3/20, NM_006989.exon3/21
  Translation: ptc
  5′ gene domains: RNA_pol_L 1.7e-08
  3′ gene domains: RasGAP 6.4e-46, C2 6e-22, BTK 5.4e-13
  TIC domains: RNA_pol_L 1.7e-08

```
HBR  CTTtCTGTACAGCCTGCAGAACT  GACAGCCACAGTGTGGAAGACCCTGTG
```

- UBE2D2–CXXC5, +5:138983230..139040133 (distance: 56902 nt)
  Donor exon (score:0.998817):NM_003339.exon6/7, NM_181838.exon7/8
  Acceptor exon (score:0.985547): NM_016463.exon2/3
  Translation: ptc
  5′ gene domains: UQ_con 4.9e-48
  3′ gene domains: zf-CXXC 1.4e-07
  TIC domains: UQ_con 3.6e-46

```
 N1  CTGAGATTGCTCGGATCTACAAAACAGATAGAGAAAA  AGTGAAGACATTT
UHR     AGATTGCgCGGATCTACAAAACAGATAGAGAAAA  AGTGAAGACATTTCCA
```

- C1orf151–NBL1, +1:19796190..19854092 (distance: 57901 nt)
  Donor exon (score:0.998429):NM_001032363.exon1/4
  Acceptor exon (score:0.998864): NM_182744.exon2/4, NM_005380.exon2/4
  Translation: ptc
  5′ gene domains: DUF543 3.4e-27
  3′ gene domains: DAN 2.9e-34, Cys_knot 5.2e-07
  TIC domains: DUF543 0.0004
  Supporting ESTs: DA050976 (brain neoplasia), BM474063 (uterus neoplasia)

```
 N1             GTGAAGATAG  GGCTCTGGAGGCCACGGGCATGATGCTTCGGGTCCTGGTG
 N1             GTGAAGATAG  GGCTCTGGAGGCCACGGGCATGATGCTTCGGGTCCTGGTGGGGGCTGTCCTCCCTGCCATGCTAC
HBR  GGCGGATGCGGTCGTGAAGATAG  GGCTCTGGAGGCCACGGGCATGATGCT
UHR           GTCGTGAAGATAG  GGCTCTGGAGGCCACGGGCATGATGCTTCGGGTCCTG
```

- PCBD2–CATSPER3, +5:134274055..134333528 (distance: 59472 nt)
  Donor exon (score:0.997346):NM_032151.exon2/4
  Acceptor exon (score:0.979264): NM_178019.exon2/8
  Translation: ptc
  5′ gene domains: Pterin_4a 2.1e-31
  3′ gene domains: Ion_trans 2.2e-29, PKD_channel 1e-06
  TIC domains: Pterin_4a 0.0066

```
 N1  AATTCTCCTTCCACAATTTTAATCAG  GAGGAACGATGATGAATGTCGGGC
```

- PLEKHO2–ANKDD1A, +15:62940827..63001173 (distance: 60345 nt)
  Donor exon (score:0.995574):NM_025201.exon5/6
  Acceptor exon (score:0.998526): NM_182703.exon4/15
  Translation: full
  5′ gene domains: PH 3.5e-10
  3′ gene domains: Death 3.5e-12
  TIC domains: PH 4.8e-10, Death 4.2e-12
  Supporting ESTs: BE883819 (uterus neoplasia)

```
UHR  ACGCCGGCCACCAACGAGAGTCCACCTGAAGGAG  TTTGGGATGAATGCGC
UHR                CGAGAGTCCACCTGAAGGAG  TTTGGGATGAATGCGCTTCTCCTGTCTGCC
```

- CTBS–GNG5, -1:84801528..84740241 (distance: 61286 nt)
Donor exon (score:0.991768):NM_004388.exon6/7
Acceptor exon (score:0.997538): NM_005274.exon3/4
Translation: full
5′ gene domains: Glyco_hydro_18 1.2e-45
3′ gene domains: G-gamma 3.4e-22
TIC domains: Glyco_hydro_18 2e-34, G-gamma 7.6e-12
AceView: CTBSandGNG5.eApr07
Supporting ESTs: AW009048 (colon neoplasia), AW361087 (colon neoplasia), AI022937 (skin normal), CF126909 (lung neoplasia), CA425643 (bone neoplasia), AW593084 (pooled tissue normal), AI275812 (uterus neoplasia), AW629022 (pooled tissue normal), AA483394 (colon neoplasia), AA180734 (muscle uncharacterized histology), AI358809 (lymph node neoplasia), CA425032 (bone neoplasia), AI753596 (bone marrow normal), AW770628 (lung neoplasia), BE671698 (lung neoplasia)

```
N1  GGGCTCCTTATTATAACTATAAA  GTTTCCCAGGCAGCTGCAGACTTGAAA
```

- LRRC33–PIGX, +3:197865915..197928126 (distance: 62210 nt)
Donor exon (score:0.998387):NM_198565.exon2/3
Acceptor exon (score:0.981205): NM_017861.exon2/6
Translation: ptc
5′ gene domains:
3′ gene domains: PIG-X 4e-48
TIC domains:
Supporting ESTs: BG533610 (liver normal)

```
N2  CTCCCAAGGAGTCTGCAAGTTG  GCATAAGGGCCATGTGTTCTGAAATTAT
```

- ELAC1–SMAD4, +18:46764931..46827288 (distance: 62356 nt)
Donor exon (score:0.999296):NM_018696.exon3/4
Acceptor exon (score:0.999968): NM_005359.exon2/12
Translation: ptc
5′ gene domains: Lactamase_B 2.5e-13
3′ gene domains: MH2 4e-77, MH1 3e-41
TIC domains: Lactamase_B 1.3e-13
AceView: ELAC1andSMAD4.iApr07
Supporting ESTs: DB215017 (uncharacterized tissue uncharacterized histology)

```
N1  TAAAGACCTTG  GTTATCCTGAATACATGTCTAACAATTTTCCTTGCAACG
```

- NTRK1–PEAR1, +1:155078609..155141164 (distance: 62554 nt)
Donor exon (score:0.999076):NM_001007792.exon2/17
Acceptor exon (score:0.999513): NM_001080471.exon3/23
Translation: full
5′ gene domains: Pkinase_Tyr 2.2e-93, Pkinase 2.6e-45
3′ gene domains:
TIC domains:

```
UHR  GTTGGCAGCAAG  CTTCACTACCACCACCAAGGAGTCCCACTCCCGCCCCT
```

- SNRPG–TIA1, -2:70374254..70311490 (distance: 62763 nt)
Donor exon (score:0.998817):NM_003096.exon1/4
Acceptor exon (score:0.999637): NM_022037.exon3/12, NM_022173.exon3/13
Translation: ptc
5′ gene domains: LSM 2.8e-21
3′ gene domains: RRM_1 2.7e-23
TIC domains:

```
UHR  CGAGTTGAAAAA  ACAGCTGGAAATGATCCCTATTGTTTTGTGGAGTTTCA
```

- CYTSA–ADORA2A, +22:23095288..23159099 (distance: 63810 nt)
Donor exon (score:0.992180):NM_015330.exon14/17
Acceptor exon (score:0.999836): NM_000675.exon2/3
Translation: ptc
5′ gene domains: CH 1.8e-22, CAMSAP_CH 2.4e-07
3′ gene domains: 7tm_1 1e-65, 7TM_GPCR_Srsx 3.2e-12
TIC domains:

UHR CAGAAGGCTATCAG GTGCCTCAGGAACCCTGAAGCTGGGCTGAGCCATGA

- ACSS1–C20orf3, -20:24976721..24912655 (distance: 64065 nt)
  Donor exon (score:0.998107):NM_032501.exon2/14
  Acceptor exon (score:0.995457): NM_020531.exon2/9
  Translation: full
  5′ gene domains: AMP-binding 3.9e-102, DUF3448 3.2e-23
  3′ gene domains: Str_synth 3.2e-28, SGL 2.9e-12
  TIC domains: DUF3448 2.2e-23, Str_synth 4.7e-28, SGL 5e-12

HBR CGATGAGCCTGGAACGGAAGTGAGGATCACCTACAG CTCCTTTAGCGGCA

- PLEKHO2–ANKDD1A, +15:62940827..63005318 (distance: 64490 nt)
  Donor exon (score:0.995574):NM_025201.exon5/6
  Acceptor exon (score:0.997673): NM_182703.exon5/15
  Translation: full
  5′ gene domains: PH 3.5e-10
  3′ gene domains: Death 3.5e-12
  TIC domains: PH 4.4e-10, Death 3.9e-12

HBR GTCCACCTGAAGGAG GATGGCCTGACCTTACTGCACTGCGCAGCCCAAAA

- APITD1–PEX14, +1:10413212..10477918 (distance: 64705 nt)
  Donor exon (score:0.997570):NM_198544.exon1/5, NM_199294.exon1/5
  Acceptor exon (score:0.999072): NM_004565.exon2/9
  Translation: full
  5′ gene domains: Somatostatin 1.8e-08
  3′ gene domains: Pex14_N 6.9e-25
  TIC domains: Pex14_N 6.9e-25

T3 GATTCTCTTACCAACAG CCAAGCTCTACTCCAG

- SFT2D2–TBX19, +1:166462004..166527022 (distance: 65017 nt)
  Donor exon (score:0.999046):NM_199344.exon1/8
  Acceptor exon (score:0.999170): NM_005149.exon2/8
  Translation: ptc
  5′ gene domains: Got1 3.7e-32
  3′ gene domains: T-box 7.8e-82
  TIC domains:
  AceView: SFT2D2andTBX19.cApr07
  Supporting ESTs: DA492985 (brain normal)

UHR GGGCAGGACACGGAGGACCGGAGCGGCCTGTCCGAG ACGGATGTTTCCAG

- HOXC10–HOXC4, +12:52666061..52733211 (distance: 67149 nt)
  Donor exon (score:0.999316):NM_017409.exon1/2
  Acceptor exon (score:0.999316): NM_014620.exon2/4
  Translation: ptc
  5′ gene domains: Homeobox 5.4e-21
  3′ gene domains: Homeobox 2e-22
  TIC domains:

UHR AGACACCTCGGATAACGAAGCGAAAG CGGGGGCTCAACCCCCAGACCTCC

- SFRS6–L3MBTL, +20:41522652..41590711 (distance: 68058 nt)
  Donor exon (score:0.991635):NM_006275.exon5/6
  Acceptor exon (score:0.999375): NM_015478.exon5/19, NM_032107.exon5/18
  Translation: ptc
  5′ gene domains: RRM_1 1.6e-16
  3′ gene domains: MBT 1.7e-32, zf-C2HC 4.2e-14
  TIC domains: RRM_1 8.9e-17

HBR TAGATCTCGAAGTATCTCAAAAAGTCGCTCCCG CAACAGGTGAGAAGAAG

- RHBDD2–POR, +7:75351102..75421243 (distance: 70140 nt)
  Donor exon (score:0.998891):NM_001040456.exon3/4, NM_001040457.exon4/5
  Acceptor exon (score:0.999765): NM_000941.exon2/16
  Translation: full
  5′ gene domains: Rhomboid 2e-17
  3′ gene domains: FAD_binding_1 2e-85, Flavodoxin_1 6.3e-38, NAD_binding_1 1.4e-20
  TIC domains: Rhomboid 1.1e-16, FAD_binding_1 2.7e-85, Flavodoxin_1 8e-38, NAD_binding_1 1.7e-20

```
HBR  CTTCAGCCGAGAGGAGGGCAGCCCAGAGCCGGAA  TTTCATGATCAACATG
HBR              GGAGGGCAGCCCAGAGCCGGAA  TTTCATGATCAACATGGGAGACTCCCAC
```

- WRB–SH3BGR, +21:39685632..39756171 (distance: 70538 nt)
  Donor exon (score:0.999462):NM_004627.exon3/5
  Acceptor exon (score:0.983755): NM_001001713.exon2/7, NM_007341.exon2/7
  Translation: full
  5′ gene domains: CHD5 4.8e-43
  3′ gene domains: SH3BGR 1.2e-46
  TIC domains: CHD5 4e-22, SH3BGR 1.8e-39
  AceView: WRBandSH3BGR.bApr07
  Supporting ESTs: CD514470 (brain normal), CN355586 (embryonic tissue normal)

```
UHR  GTGATAAGTGTCGCTTTCTACGTATTGCAG  ATTAGGAAGAAACAGCAAGA
```

- CYTSA–ADORA2A, +22:23095288..23166551 (distance: 71262 nt)
  Donor exon (score:0.992180):NM_015330.exon14/17
  Acceptor exon (score:0.999526): NM_000675.exon3/3
  Translation: tle
  5′ gene domains: CH 1.8e-22, CAMSAP_CH 2.4e-07
  3′ gene domains: 7tm_1 1e-65, 7TM_GPCR_Srsx 3.2e-12
  TIC domains:

```
HBR  CAGAAGGCTATCAG  GTACAATGGCTTGGTGACCGGCACGAGGGCTAAGGG
```

- ELAC1–SMAD4, +18:46754929..46827288 (distance: 72358 nt)
  Donor exon (score:0.996170):NM_018696.exon2/4
  Acceptor exon (score:0.999968): NM_005359.exon2/12
  Translation: ptc
  5′ gene domains: Lactamase_B 2.5e-13
  3′ gene domains: MH2 4e-77, MH1 3e-41
  TIC domains: Lactamase_B 0.0045
  AceView: ELAC1andSMAD4.pApr07
  Supporting ESTs: AL703589 (muscle normal), DA116592 (cerebellum normal), DA116561 (cerebellum normal), DA496537 (brain normal), DB227625 (uncharacterized tissue uncharacterized histology), DA489021 (brain normal), DB201919 (uncharacterized tissue uncharacterized histology), DA724644 (testis neoplasia), DA441477 (uncharacterized tissue neoplasia), DB098459 (testis uncharacterized histology)

```
 N1  gACAGACACAGCTTATGAAAAGCCAACTTAAAGCAG  GTTATCCTGAATACATGTCTAACAATTTTCCTTGCAACG
```

- FKBP1A–SDCBP2, -20:1321478..1249079 (distance: 72398 nt)
  Donor exon (score:0.999476):NM_000801.exon2/5, NM_054014.exon2/4
  Acceptor exon (score:0.996204): NM_080489.exon2/9
  Translation: ptc
  5′ gene domains: FKBP_C 1.4e-34
  3′ gene domains: PDZ 5.2e-09
  TIC domains:
  AceView: FKBP1AandSDCBP2.tApr07
  Supporting ESTs: DB502960 (brain uncharacterized histology), BI549365 (cerebrum normal), DB493332 (brain uncharacterized histology), BG705414 (cerebrum normal), DA095179 (cerebellum normal), DB045614 (testis normal), DB464303 (cerebrum uncharacterized histology), DB475043 (cerebrum uncharacterized histology), DB141161 (thymus uncharacterized histology), DA061374 (cerebellum normal), DA384295 (brain normal), BM925547 (brain normal), DC332371 (cerebrum normal), DB505449 (brain uncharacterized histology)

```
 T3           AGACCTGCGTGGTGCACTACACCG  GGTGTTCTG
 T3                        GCACTACACCG  GGTGTTCTGAGAATCAGCCATG
 N2                      GTGCACTACACCG  GGTGTTCTGAGAATCAGCCATGTCATCCCTGTACCCc
HBR  AGCGCGGCCAGACCTGCGTGGTGCACTACACCG  GGTGTTCTGAGAATCAG
HBR   GCGCGGCCAGACCTGCGTGGTGCACTACACCG  GGTGTTCTGAGAATCAGC
HBR   GCGgGGCCAGACCTGCGTGGTGCACTACACCG  GGTGTTCTGAGAATCAGC
HBR           AGACCTGCGTGGTGCACTACACCG  GGTGTTCTGAGAATCAGCCATGTCAT
UHR  AGCGCGGCCAGACCTGCGgGGTGCACTACACCG  GGTGTTCTGAGAATCAG
UHR    CGCGGCCAGACCTGCGTGGTGCACTACACCG  GGTGTTCTGAGAATCAGCC
UHR              CCTGCGTGGTGCACTACACCG  GGTGTTCTGAGAATCAGCCATGTCATCCC
UHR                          ACTACACCG  GGTGTTCTGAGAATCAGCCATGTCATCCCTGTACCCATCTC
UHR                          ACTACACCG  GGTGTTCTGAGAATCAGCCATGTCATCCCTGTACCCATCTC
```

- HACL1–COLQ, -3:15579869..15506148 (distance: 73720 nt)
  Donor exon (score:0.997705):NM_012260.exon16/17
  Acceptor exon (score:0.981758): NM_005677.exon2/17, NM_080539.exon2/16, NM_080538.exon2/17
  Translation: ptc
  5′ gene domains: TPP_enzyme_N 2.7e-45, TPP_enzyme_M 1.5e-32, TPP_enzyme_C 1.9e-24
  3′ gene domains: Collagen 9.6e-10
  TIC domains: TPP_enzyme_N 2.8e-45, TPP_enzyme_M 1.5e-32, TPP_enzyme_C 2e-24

```
 N3                            GGAAGGCCCAG  CCCTTCCCAGCCTGGATCAGAA
HBR  CATGATTGAGCCACAAGCCACACGGAAGGCCCAG  CCCTTCCCAGCCTGGA
HBR                        CACGGAAGGCCCAG  CCCTTCCCAGCCTGGATCAGAAGAAGCGTGGTGGCC
UHR       TTGAGCCACAAGCCACACGGAAGGCCCAG  CCCTTCCCAGCCTGGATCAGA
```

- EIF3K–ACTN4, +19:43808582..43883080 (distance: 74497 nt)
  Donor exon (score:0.952995):NM_013234.exon4/8
  Acceptor exon (score:0.998971): NM_004924.exon2/21
  Translation: tle
  5′ gene domains: PCI_Csn8 6.4e-38, SAC3_GANP 7.1e-28
  3′ gene domains:
  TIC domains: SAC3_GANP 1.7e-16, PCI_Csn8 2.2e-13

```
UHR  CAGGCCTTCTGG  ACCTTCACGGCATGGTGCAACTCCCACCTGCGGAAGGC
```

- RAD51L3–RFFL, -17:30452333..30377693 (distance: 74639 nt)
  Donor exon (score:0.998715):NM_001142571.exon9/10, NM_002878.exon9/10, NM_133629.exon6/7
  Acceptor exon (score:0.995918): NM_001017368.exon2/7, NM_057178.exon2/7
  Translation: ptc
  5′ gene domains: Rad51 9.2e-20, KaiC 9.7e-11, DnaB_C 3.6e-07
  3′ gene domains:
  TIC domains: Rad51 5.4e-06, KaiC 1.5e-10, DnaB_C 0.0038
  Supporting ESTs: BI823883 (pooled tissue normal)

```
 N3  GCCAAATCTTCCCGACAG  ATTTTATCATGTGGG
```

- NDUFA9–GALNT8, +12:4664752..4740385 (distance: 75632 nt)
  Donor exon (score:0.998948):NM_005002.exon10/11
  Acceptor exon (score:0.999775): NM_017417.exon7/11
  Translation: full
  5′ gene domains: 3Beta_HSD 9.7e-08, Epimerase 1.1e-08, NmrA 2.7e-09
  3′ gene domains: Glycos_transf_2 5e-22, Ricin_B_lectin 3.4e-12
  TIC domains: 3Beta_HSD 2e-07, Epimerase 2.7e-08, NmrA 6.5e-09, Ricin_B_lectin 2.8e-12

```
UHR  TAACAAGGGATAAAGTGGAGCGG  GTGTGGCAGTGTGGAGGGAAGGTCGAG
```

- PLEKHO2–ANKDD1A, +15:62934289..63010073 (distance: 75783 nt)
  Donor exon (score:0.997691):NM_025201.exon3/6
  Acceptor exon (score:0.996738): NM_182703.exon7/15
  Translation: full
  5′ gene domains: PH 3.5e-10
  3′ gene domains: Death 3.5e-12
  TIC domains: PH 9.5e-05, Death 2.5e-12

```
HBR  TGCTGCGATCCCCAGGGAACAAG  GAGGGGAACACTGCCCTTCATCTGGCT
```

- NEUROD1–CERKL, -2:182253402..182177051 (distance: 76350 nt)
  Donor exon (score:0.997806):NM_002500.exon1/2
  Acceptor exon (score:0.998724): NM_201548.exon2/13, NM_001030312.exon2/10, NM_001030311.exon2/14, NM_001030313.exon2/11
  Translation: tss
  5′ gene domains: Neuro_bHLH 4.1e-51, HLH 1.4e-15
  3′ gene domains: DAGK_cat 2.9e-17
  TIC domains: DAGK_cat 1.6e-13
  AceView: NEUROD1andCERKL.fApr07

```
HBR  CACTGCTCAGGACCTACTAACAACAAAG  GTGATTCTAAGTATGACTTGCT
```

- RPS10–NUDT3, -6:34494124..34417727 (distance: 76396 nt)
  Donor exon (score:0.983597):NM_001014.exon5/6
  Acceptor exon (score:0.997246): NM_006703.exon2/5
  Translation: full
  5′ gene domains: S10_plectin 9.8e-46

3′ gene domains: NUDIX 6.5e-19
TIC domains: S10_plectin 3.9e-45, NUDIX 8.7e-16
AceView: RPS10andNUDT3.fApr07
Supporting ESTs: BU521288 (uterus neoplasia), BE796938 (lung neoplasia), CN332176 (embryonic tissue normal)

```
 N2                   CAGCAACCGAATTCCAGTTT  GTGCTACTCGTGAGCAGTAGTCGCCATCCA
HBR      GGCTGGGGCTGGGTCAGCAACCGAATTCCAGTTT  GTGCTACTCGTGAGCA
HBR             GCTGGGTCAGCAACCGAATTCCAGTTg  GTGCTACTCGTGAGCAGTAGTCG
HBR             GCTGGGTCAGCAACCGAATTCCAGTTT  GTGCTACTCGTGAGCAGTAGTCG
HBR                TGGGTCAGCAACCGAATTCCAGTTT  GTGCTACTCGTGAGCAGTAGTCGCC
HBR               gGGGTCAGCAACCGAATTCCAGTTT  GTGCTACTCGTGAGCAGTAGTCGCC
HBR                   CAGCAACCGAATTCCAGTTT  GTGCTACTCGTGAGCAGTAGTCGCCATCCA
HBR                         CCGAATTCCAGTTT  GTGCTACTCGTGAGCAGTAGTCGCCATCCAGACAGA
HBR                          CGAATTCCAGTTT  GTGCTACTCGTGAGCAGTAGTCGCCATCCAGACAGAT
UHR  CCGAGGCTGGGGCTGGGTCAGCAACCGAATTCCAGTTT  GTGCTACTCGTG
UHR                 GTCAGCAACCGAATTCCAGTTT  GTGCTACTCGTGAGCAGTAGTCGCCATC
UHR                   CAGCAACCGAATTCCAGTTT  GTGCTACTCGTGAGCAGTAGTCGCCATCCA
UHR                          CGAATTCCAGTTT  GTGCTACTCGTGAGCAGTAGTCGCCATCCAGACAGAT
```

- SGK269–TSPAN3, -15:75212402..75135652 (distance: 76749 nt)
  Donor exon (score:0.999341):NM_024776.exon5/6
  Acceptor exon (score:0.998979): NM_005724.exon2/7, NM_198902.exon2/6
  Translation: full
  5′ gene domains: Pkinase 2.6e-13
  3′ gene domains: Tetraspannin 2.3e-24
  TIC domains: Tetraspannin 7e-23

```
HBR  GATCCACTTAATAACTATGCAGTCAAG  GGGGCAGCTGGCATTTgATGCTA
```

- CHURC1–FNTB, +14:64462551..64540720 (distance: 78168 nt)
  Donor exon (score:0.997705):NM_145165.exon3/4
  Acceptor exon (score:0.995401): NM_002028.exon2/12
  Translation: full
  5′ gene domains: Churchill 7.8e-60
  3′ gene domains: Prenyltrans 1e-12
  TIC domains: Churchill 7.9e-40, Prenyltrans 1.1e-12
  AceView: CHURC1andFNTB.iApr07
  Supporting ESTs: DA225696 (brain normal), DA179557 (cerebrum normal), DA305636 (cerebrum normal), DA515843 (brain normal), DA448500 (uncharacterized tissue neoplasia), DA266761 (brain normal)

```
 N1  TGTCATCATGTAATAGCCAGACATGAGTATACATTCAGTATCATGGATGAATTTCAG  GCAAAAGTAGAAGAAAAG
 N1          TGTAATAGCCAGACATGAGTATACATTCAGTATCATGGATGAATTTCAG  GCAAAAGTAGAAGAAAAGATCCAAGA
UHR                                                  AATTTCAG  GCAAAAGTAGAAGAAAAGATCCAAGAGGTCTTCAGTTCTTAC
```

- MRAS–ESYT3, +3:139574608..139653580 (distance: 78971 nt)
  Donor exon (score:0.997965):NM_001085049.exon2/6, NM_012219.exon2/6
  Acceptor exon (score:0.995590): NM_031913.exon2/23
  Translation: ptc
  5′ gene domains: Ras 2.7e-53, Miro 2.6e-21, GTP_EFTU 6.8e-08
  3′ gene domains: C2 2e-19
  TIC domains: Ras 4.2e-12, Miro 0.001

```
HBR  TGACAATCAATGGGCCATCTTGGACG  ATCCACTTCCCGGACGTGGAGCGG
```

- CHURC1–FNTB, +14:64460597..64540720 (distance: 80122 nt)
  Donor exon (score:0.999281):NM_145165.exon2/4
  Acceptor exon (score:0.995401): NM_002028.exon2/12
  Translation: ptc
  5′ gene domains: Churchill 7.8e-60
  3′ gene domains: Prenyltrans 1e-12
  TIC domains: Churchill 1.7e-27
  AceView: CHURC1andFNTB.oApr07
  Supporting ESTs: DB180004 (kidney neoplasia), DA437669 (uncharacterized tissue neoplasia)

```
 N2  GGAGAAGAAATAGTTACCTATGATC  GCAAAAGTAGAAGAAAAGATCCAAG
 N2  GGAGAAGAAATAGTTACCTATGATC  GCAAAAGTAGAAGAAAAGATCCAAG
 N2  GGAGAAGAAAgAGTTACCTATGATC  GCAAAAGTAGAAGAAAAGATCCAAG
```

- RNASET2–RPS6KA2, -6:167272373..167191737 (distance: 80635 nt)
  Donor exon (score:0.997365):NM_003730.exon6/9
  Acceptor exon (score:0.996085): NM_001006932.exon2/22
  Translation: ptc
  5′ gene domains: Ribonuclease_T2 9.3e-44
  3′ gene domains: Pkinase 5.3e-72, Pkinase_Tyr 4.7e-41, Pkinase_C 3.2e-11
  TIC domains: Ribonuclease_T2 2e-31

```
 N1                 AGGGAGCTGGACCTCAACAG  ACCACAGAGGAGGATCTCAACCTGGATGTG
UHR  GCCcGGAACTCTACAGGGAGCTGGACCTCAACAG  ACCACAGAGGAGGATC
```

- BACE2–FAM3B, +21:41551123..41632175 (distance: 81051 nt)
  Donor exon (score:0.998715):NM_138991.exon7/8, NM_012105.exon8/9
  Acceptor exon (score:0.994805): NM_058186.exon3/8, NM_206964.exon2/7
  Translation: full
  5′ gene domains: Asp 1.1e-45
  3′ gene domains:
  TIC domains: Asp 1.8e-45

```
UHR  GTGGGCTTCGCAGCGAGCCCCTGTGCAG  CTCCAGTCCCCAAAAGGCAAAA
```

- P2RY6–ARHGEF17, +11:72653342..72735576 (distance: 82233 nt)
  Donor exon (score:0.999539):NM_176796.exon1/4, NM_176797.exon1/3
  Acceptor exon (score:0.999860): NM_014786.exon2/21
  Translation: tss
  5′ gene domains: 7tm_1 3.1e-50
  3′ gene domains: RhoGEF 1.4e-42
  TIC domains: RhoGEF 4.9e-43

```
HBR  GCCGGACCAGCTGGTGCTGCGCGGCGCAG  GACATGCGGAAGCACGTGGCC
HBR                        CGGCcCAG  GACATGCGGAAGCACGTGGCCATGACCCTGCTGGACACAGAG
```

- NSL1–BATF3, -1:211022337..210937030 (distance: 85306 nt)
  Donor exon (score:0.999388):NM_001042549.exon4/7, NM_015471.exon4/6
  Acceptor exon (score:0.996019): NM_018664.exon2/3
  Translation: ptc
  5′ gene domains: Mis14 2.1e-32
  3′ gene domains: bZIP_1 1.4e-13, bZIP_2 3.3e-10
  TIC domains: Mis14 1.8e-23

```
UHR  ATGACCCTGATCCAG  AGCCCTGAGGATGATGACAGGAAGGTCCGAAGGAG
```

- PCBD2–CATSPER3, +5:134274055..134359862 (distance: 85806 nt)
  Donor exon (score:0.997346):NM_032151.exon2/4
  Acceptor exon (score:0.980767): NM_178019.exon3/8
  Translation: full
  5′ gene domains: Pterin_4a 2.1e-31
  3′ gene domains: Ion_trans 2.2e-29, PKD_channel 1e-06
  TIC domains: Ion_trans 2e-29, PKD_channel 8.9e-07

```
 N1  AAAGAATTCTCCTTCCACAATTTTAATCAG  TTCTCGGAGATCTTCTTTGT
```

- TTLL5–C14orf179, +14:75438320..75524981 (distance: 86660 nt)
  Donor exon (score:0.996170):NM_015072.exon31/32
  Acceptor exon (score:0.999728): NM_052873.exon2/8, NM_001102564.exon2/9
  Translation: ptc
  5′ gene domains: TTL 9.5e-91
  3′ gene domains:
  TIC domains: TTL 9.8e-91
  Supporting ESTs: AW505498 (lymph node normal)

```
 N2                          CAGCTCTACAG  AGGGCCAAGATGGGTCGCCGAGCTCAACAGGAGTCAGCG
UHR  CCTGCAGCCTTTGTGCCCATCACCAGCTCTACAG  AGGGCCAAGATGGGTC
```

- HDAC11–FBLN2, +3:13500064..13586815 (distance: 86750 nt)
  Donor exon (score:0.996222):NM_001136041.exon3/10, NM_024827.exon3/10
  Acceptor exon (score:0.998904): NM_001998.exon2/17, NM_001004019.exon2/18
  Translation: ptc
  5′ gene domains: Hist_deacetyl 1.6e-61
  3′ gene domains: EGF_CA 4e-12, ANATO 3.8e-07
  TIC domains: Hist_deacetyl 7.5e-06

```
HBR  CTGGTGGTGCACACGAGGCGCTATCTTAATGAGCTCAAG  GGTCTTACAGG
```

- WDR92–C1D, -2:68215338..68127955 (distance: 87382 nt)
  Donor exon (score:0.996127):NM_138458.exon7/8
  Acceptor exon (score:0.958075): NM_006333.exon2/5, NM_173177.exon2/5
  Translation: ptc
  5′ gene domains:
  3′ gene domains: Sas10_Utp3 3e-19
  TIC domains:

```
 N2  CACCTCTGGAAGTA  TCAGCCATAATGGCAGGTGAAGAAATTAATGAAGAC
```

- HDAC11–FBLN2, +3:13497894..13586815 (distance: 88920 nt)
  Donor exon (score:0.998372):NM_001136041.exon2/10, NM_024827.exon2/10
  Acceptor exon (score:0.998904): NM_001998.exon2/17, NM_001004019.exon2/18
  Translation: full
  5′ gene domains: Hist_deacetyl 1.6e-61
  3′ gene domains: EGF_CA 4e-12, ANATO 3.8e-07
  TIC domains: EGF_CA 4.4e-12, ANATO 4.1e-07

```
HBR  AAAATGGGGCAAAGTGATCAATTTCCTAAAAG  GGTCTTACAGGAGAGGGG
```

- RAD51L3–RFFL, -17:30470243..30377693 (distance: 92549 nt)
  Donor exon (score:0.998710):NM_001142571.exon2/10, NM_002878.exon2/10, NM_133629.exon2/7
  Acceptor exon (score:0.995918): NM_001017368.exon2/7, NM_057178.exon2/7
  Translation: ptc
  5′ gene domains: Rad51 9.2e-20, KaiC 9.7e-11, DnaB_C 3.6e-07
  3′ gene domains:
  TIC domains:

```
HBR  GGAAGAGGTAGCTCAGAAATGTGGCTTGTCTTACAAG  ATTTTATCATGTG
```

- GPR75–ASB3, -2:53940469..53846226 (distance: 94242 nt)
  Donor exon (score:0.998325):NM_006794.exon1/2
  Acceptor exon (score:0.998200): NM_016115.exon2/10
  Translation: tss
  5′ gene domains: 7tm_1 3.3e-20
  3′ gene domains: Ank 2.7e-09, SOCS_box 1.5e-14
  TIC domains: Ank 2.9e-09, SOCS_box 1.6e-14
  AceView: GPR75andASB3.bApr07
  Supporting ESTs: DA349354 (brain normal), BM926369 (brain normal), DC314536 (cerebrum normal), DB168761 (kidney neoplasia), DA201990 (brain normal), DA080065 (cerebellum normal), BG702192 (cerebrum normal), BP350378 (brain uncharacterized histology)

```
 T3      CGGCGCTGCGGCCACCCGGCAG  ACTGGTCAAAC
HBR  GGGGCGGCGCTGCGGCCACCCGGCAG  ACTGGTCAAACAAATGGATTTTAC
HBR              GCGGCCACCCGGCAG  ACTGGTCAAACAAATGGATTTTACAGAGGCTTACG
HBR              GCGGCCACCCGGCAG  ACTGGTCAAACAAATGGATTTTtCAGAGGCTTACG
HBR                   CACCCGGCAG  ACTGGTCAAACAAATGGATTTTACAGAGGCTTACGCGGAC
```

- CDKL3–PPP2CA, -5:133666180..133569721 (distance: 96458 nt)
  Donor exon (score:0.997718):NM_001113575.exon12/13
  Acceptor exon (score:0.999534): NM_002715.exon2/7
  Translation: full
  5′ gene domains: Pkinase 1.3e-73, Pkinase_Tyr 1.1e-39
  3′ gene domains: Metallophos 2.8e-42
  TIC domains: Pkinase 2.9e-73, Pkinase_Tyr 2.4e-39, Metallophos 2.6e-41

```
HBR  AAAAACAAGAG  GCTAAAGAAATCCTGACAAAAGAATCCAACGTGCAAGAG
```

- FBXL18–TNRC18, -7:5497388..5400752 (distance: 96635 nt)
  Donor exon (score:0.999388):NM_024963.exon4/5
  Acceptor exon (score:0.999431): NM_001080495.exon3/30
  Translation: ptc
  5′ gene domains:
  3′ gene domains: BAH 3.3e-09
  TIC domains:

```
UHR  CTGCAGCAGTCGCTTCTCCGCAG  GCGAGGCCTTCTTGGGCAGCTTTGTGG
```

- CTNNBIP1–CLSTN1, -1:9853832..9756039 (distance: 97792 nt)
  Donor exon (score:0.998363):NM_020248.exon5/6, NM_001012329.exon4/5
  Acceptor exon (score:0.963892): NM_014944.exon2/18, NM_001009566.exon2/19
  Translation: full
  5′ gene domains: ICAT 3.7e-33
  3′ gene domains: Cadherin 1.1e-08
  TIC domains: ICAT 4.9e-25, Cadherin 1.2e-08

```
 N1                        CCGCACTCCATCGACCAGG  TTAACAAGCACAAGCCCTGGCTGGAGCCCACCTACCACGGCATAGTCACAGAGAAC
HBR  CAGCCAGCTCAGCCAGCTGCCTCCGCACTCCATCGACCgGG  TTAACAAGC
HBR    GCCAGCTCAGCCAGCTGCCTCCGCACTCCATCGACCAGG  TTAACAAGCAC
HBR        GCTCAGCCAGCTGCCTCCGCACTCCATCGACCAGG  TTAACAAGCACAAGC
HBR         CTCAGCCAGCTGCCTCCGCACTCCATCGACCAGG  TTAACAAGCACAAGCC
HBR         CTCAGCCAGCTGCCTCCGCACTCCATCGACCAGG  TTAACAAGCACAAGCC
HBR           CAGCCAGCTGCCTCCGCACTCCATCGACCAGG  TTAACAAGCACAAGCCCT
HBR              CCAGCTGCCTCCGCACTCCATCGACCAGG  TTAACAAGCACAAGCCCTGGC
HBR                        CCGCACTCCATCGACCAGG  TTAACAAGCACAAGCCCTGGCTGGAGCCCAC
HBR                                  TtGACCAGG  TTAACAAGCACAAGCCCTGGCTGGAGCCCACCTACCACGGC
```

- CNRIP1–PPP3R1, -2:68397793..68297767 (distance: 100025 nt)
  Donor exon (score:0.999147):NM_015463.exon2/3, NM_001111101.exon2/3
  Acceptor exon (score:0.999905): NM_000945.exon2/6
  Translation: full
  5′ gene domains:
  3′ gene domains: efhand 9.1e-07
  TIC domains: efhand 1.9e-06

```
HBR  GAACGGCAACCCATCCAGATCACCATGCCG  GGAAATGAGGCAAGTTATCC
HBR              ATCCAGATCcCCATGCCG  GGAAATGAGGCAAGTTATCCTTTGGAAATGTG
HBR                   GATCACCATGCCG  GGAAATGAGGCAAGTTATCCTTTGGAAATGTGCTCAC
```

- VWA5B2–EIF4G1, +3:185434215..185535209 (distance: 100993 nt)
  Donor exon (score:0.997365):NM_138345.exon4/19
  Acceptor exon (score:0.998589): NM_198241.exon33/33, NM_198244.exon30/30, NM_004953.exon26/26, NM_198242.exon29/29, NM_182917.exon32/32
  Translation: full
  5′ gene domains:
  3′ gene domains: MIF4G 2.5e-68, MA3 2e-28, W2 2.6e-16
  TIC domains: W2 7.2e-15

```
HBR  cGCCTGCTTGCAG  ACCTGCTGCGGATGTTCTTTGACGCACTGTATGACGA
```

- RPSA–MOBP, +3:39428272..39529878 (distance: 101605 nt)
  Donor exon (score:0.998914):NM_001012321.exon4/6, NM_002295.exon5/7
  Acceptor exon (score:0.991227): NM_182935.exon4/4
  Translation: tle
  5′ gene domains: Ribosomal_S2 1.4e-38
  3′ gene domains:
  TIC domains: Ribosomal_S2 8.1e-39

```
 T3  CTTCTAtAGAGATCCTGAAGAG  ATTGAAAAGGA
```

- FBXL18–TNRC18, -7:5497388..5395493 (distance: 101894 nt)
  Donor exon (score:0.999388):NM_024963.exon4/5
  Acceptor exon (score:0.999722): NM_001080495.exon5/30
  Translation: ptc
  5′ gene domains:
  3′ gene domains: BAH 3.3e-09
  TIC domains:

```
 T2  TCcCTTCTCCGCAG  ACGGTTTCTACCTGCCCAC
```

- C3orf31–VGLL4, -3:11825991..11719521 (distance: 106469 nt)
  Donor exon (score:0.996545):NM_138807.exon6/7
  Acceptor exon (score:0.999384): NM_014667.exon2/6
  Translation: ptc
  5′ gene domains: Mmp37 2.5e-110
  3′ gene domains:
  TIC domains: Mmp37 5e-108

```
HBR  TGTGGTGCGACTAG  GCTCCAACCAGGAATGGAGACGCCATTGGATGTTTT
```

- FAM18B2–CDRT4, -17:15389824..15282242 (distance: 107581 nt)
  Donor exon (score:0.999088):NM_145301.exon5/6, NM_001135036.exon5/6
  Acceptor exon (score:0.988523): NM_173622.exon4/4
  Translation: tle
  5′ gene domains: DUF846 3.1e-53
  3′ gene domains:
  TIC domains: DUF846 1.7e-45

```
UHR  TTCTCCTTCACAGTAAAGTGGCTG  GACTCACAGAAAACACTGGACTTCCC
```

- NUDCD3–CAMK2B, -7:44398421..44290349 (distance: 108071 nt)
  Donor exon (score:0.995661):NM_015332.exon5/6
  Acceptor exon (score:0.999616): NM_172082.exon2/20, NM_172083.exon2/18, NM_172084.exon2/16, NM_172078.exon2/21, NM_172081.exon2/19, NM_172079.exon2/20, NM_172080.exon2/20, NM_001220.exon2/24
  Translation: ptc
  5′ gene domains: CS 9.5e-17
  3′ gene domains: Pkinase 3.5e-73, CaMKII_AD 4.2e-60, Pkinase_Tyr 4.1e-32
  TIC domains: CS 8.6e-17

```
HBR  CTGCAGGGCAAGCCACAGAGCCATGAGCTG  GGGGGCTTTCTCTGTGGTCC
```

- FCF1–YLPM1, +14:74252555..74365669 (distance: 113113 nt)
  Donor exon (score:0.992015):NM_015962.exon4/8
  Acceptor exon (score:0.998546): NM_019589.exon19/21
  Translation: full
  5′ gene domains: Fcf1 1.7e-41
  3′ gene domains:
  TIC domains:

```
HBR  TCAATGATGGACTGTCTGTATGCCAAGT  ACAAGTTGGATGGCTTGAGGAC
```

- RAMP1–UBE2F, +2:238433109..238546473 (distance: 113363 nt)
  Donor exon (score:0.997652):NM_005855.exon1/3
  Acceptor exon (score:0.999719): NM_080678.exon2/10
  Translation: ptc
  5′ gene domains: RAMP 5.6e-49
  3′ gene domains: UQ_con 1.9e-30
  TIC domains:

```
HBR  CTCCCGCGGCGCGGCCTCTGGCTGCTCCTGG  GGTAAAGGCAGCAGTAATG
```

- POC1B–DUSP6, -12:88384678..88268933 (distance: 115744 nt)
  Donor exon (score:0.996033):NM_172240.exon9/12
  Acceptor exon (score:0.999715): NM_001946.exon2/3
  Translation: ptc
  5′ gene domains: WD40 6.1e-14
  3′ gene domains: DSPc 5.8e-43, Rhodanese 5.8e-14
  TIC domains: WD40 4.2e-14

```
UHR  ATCCCCATGAGGAAAAAGTTGAGACTGTAGAA  GTGGCTTCAGTAAGTTCC
```

- TMEM111–CIDEC, -3:10003191..9886753 (distance: 116437 nt)
  Donor exon (score:0.993068):NM_018447.exon1/8
  Acceptor exon (score:0.997162): NM_022094.exon5/6
  Translation: ptc
  5′ gene domains: DUF106 5.3e-52
  3′ gene domains: CIDE-N 7.6e-31
  TIC domains: DUF106 5.3e-10

```
 N1  CGCCACTACGTGTCCATCCTGCTGgAGAGCGACAAGAAGCTCACCCAGGAACAAGTATCTGACAG  GGGACAAGGC
 N1    CCACTACGTGTCCATCCTGCTGgAGAGCGACAAGAAGCTCACCCAGGAACAAGTATCTGACAG  GGGACAAGGCAC
 N1                                       CTCACCCAGGAACAAGTATCTGACAG  GGGACAAGGCACCCACaGTCCCTC
 N1                                             CAGGAACAAGTATCTGACAG  GGGACAAGGCACCCACaGTCCCTCTCCCATAAGCCTGCCAAGAAGATTGATGT[illegible]
 N1                                               GGAACAAGTATCTGACAG  GGGACAAGGCACCCACaGTCCCTCTCCCATAAGCCTGCCAAGAAGATTGATGT[illegible]
 N1                                                GAACAAGTATCTGACAG  GGGACAAGGCACCCACaGTCCCTCTCCCATAAG
 N2                            AGCGACAAGAAGCTCACCCAGGAACAAGTATCTGACAG  GGGACgAGGCAC
HBR                          AGAGCGACAAGAAGCTCACCCAGGAACAAGTATCTGACAG  GGGACgAGGC
HBR                          AGAGCGACAAGAAGCTCACCCAGGAACAAGTATCTGACAG  GGGACgAGGC
```

```
HBR                                   CGACAAGAAGCTCACCCAGGAACAAGTATCTGACAG  GGGACgAGGCACCC
HBR                                           AAGCTCACCCAGGAACAAGTATCTGACAG  GGGACAAGGCACCCACaGTCC
HBR                                                            ACAAGTATCTGACAG  GGGACAAGGCACCCACaGTCCCTCTCCCATAAGCC
```

- C15orf40–HOMER2, -15:81469993..81352597 (distance: 117395 nt)
Donor exon (score:0.994425):NM_144597.exon1/3
Acceptor exon (score:0.998735): NM_199332.exon2/9, NM_004839.exon2/9, NM_199331.exon2/9, NM_199330.exon2/9
Translation: ptc
5′ gene domains: DUF167 1.1e-24
3′ gene domains: WH1 1.3e-35
TIC domains:

```
 N2  GCTCCAAACAAAATGCTGTAACAG  AGAACAGCCCATCTTCACCACCCGAG
```

- PPFIBP1–MRPS35, +12:27679315..27799381 (distance: 120065 nt)
Donor exon (score:0.983255):NM_177444.exon4/5, NM_003622.exon4/30
Acceptor exon (score:0.999631): NM_021821.exon8/8
Translation: full
5′ gene domains: SAM_1 5.5e-17, SAM_2 3.7e-14
3′ gene domains: MRP-S28 5e-36
TIC domains:

```
HBR  CGCTTGTTGAATGGCTTCAGAGTCAAATG  AATACTGAAGAATGGGAAAAA
```

- RAD18–OXTR, -3:8907073..8785277 (distance: 121795 nt)
Donor exon (score:0.988565):NM_020165.exon12/13
Acceptor exon (score:0.999142): NM_000916.exon2/4
Translation: ptc
5′ gene domains: SAP 8.3e-12
3′ gene domains: 7tm_1 7.5e-51, 7TM_GPCR_Srv 7.4e-07, 7TM_GPCR_Srsx 4.1e-07
TIC domains: SAP 7.7e-12

```
UHR  AGAGGAAGCCTGGGAAGCATCACATAA  TGGAAGCCGCTGAACATCCCGAG
UHR                 AAGCATCACATAA  TGGAAGCCGCTGAACATCCCGAGGAACTGGCACGCTG
```

- TIMM23–AGAP7, -10:51276994..51153248 (distance: 123745 nt)
Donor exon (score:0.999388):NM_006327.exon6/7
Acceptor exon (score:0.980247): NM_001077685.exon2/7
Translation: full
5′ gene domains: Tim17 7.9e-23
3′ gene domains: ArfGap 3.3e-38, PH 7.4e-15, Ank 1.4e-07
TIC domains: Tim17 1.7e-16, ArfGap 4e-38, PH 9.4e-15, Ank 1.6e-07

```
HBR  CTGGAACCATGACAGGCATGTTGTATAAATGTACAG  CTTTGGAGTTTAAC
```

- IDH3A–DNAJA4, +15:76228827..76359442 (distance: 130614 nt)
Donor exon (score:0.998710):NM_005530.exon1/11
Acceptor exon (score:0.999251): NM_001130183.exon6/7, NM_001130182.exon6/7, NM_018602.exon7/8
Translation: ptc
5′ gene domains: Iso_dh 1.9e-110
3′ gene domains: DnaJ_C 1.6e-24, DnaJ_CXXCXGXG 1.7e-17, DnaJ 8.3e-29
TIC domains:

```
 T3  GGATCTCTAAG  GTGAGGTGATAAAGCACGGGGg
```

- RNF115–PDZK1, +1:144322698..144458399 (distance: 135700 nt)
Donor exon (score:0.999088):NM_014455.exon1/9
Acceptor exon (score:0.994615): NM_002614.exon3/10
Translation: ptc
5′ gene domains:
3′ gene domains: PDZ 7.3e-14
TIC domains:

```
HBR  GCCACTTTTGCAAGGGCGAGGTCAGCCCCAAACTACCG  AAATGACCTCCA
```

- ITGAL–PRR14, +16:30437541..30574173 (distance: 136631 nt)
Donor exon (score:0.993825):NM_002209.exon29/31, NM_001114380.exon27/29
Acceptor exon (score:0.991079): NM_024031.exon9/12
Translation: ptc
5′ gene domains: Integrin_alpha2 7.3e-78, VWA 5.1e-19, FG-GAP 3.3e-07
3′ gene domains:
TIC domains: Integrin_alpha2 6.2e-78, VWA 4.6e-19, FG-GAP 3e-07

```
T3  CCCTGGCCCAG  GTTGGGTTCAACCAAAGGGAAG
```

- RAD18–OXTR, -3:8907073..8769910 (distance: 137162 nt)
  Donor exon (score:0.988565):NM_020165.exon12/13
  Acceptor exon (score:0.978795): NM_000916.exon4/4
  Translation: tle
  5′ gene domains: SAP 8.3e-12
  3′ gene domains: 7tm_1 7.5e-51, 7TM_GPCR_Srv 7.4e-07, 7TM_GPCR_Srsx 4.1e-07
  TIC domains: SAP 9.7e-12

```
UHR  GCCTGGGAAGCATCACATAA  CCTCGGCCTTCATCATCGTCATGCTCCTGG
```

- TAF13–CLCC1, -1:109419132..109281563 (distance: 137568 nt)
  Donor exon (score:0.997470):NM_005645.exon2/4
  Acceptor exon (score:0.999189): NM_001048210.exon10/12, NM_015127.exon10/12
  Translation: ptc
  5′ gene domains: TFIID-18kDa 1.1e-37
  3′ gene domains: MCLC 1.6e-246
  TIC domains:

```
N1  AAGAAATTGGAGGAGGTGCAGAAGGTGGACAGGGTAAAAGAAAGAGACTTTTTTCTAAAGAAT  AGTTTCTGCTAT
```

- PRKAG2–RHEB, -7:150960088..150819033 (distance: 141054 nt)
  Donor exon (score:0.999297):NM_024429.exon1/12, NM_001040633.exon5/16, NM_016203.exon5/16
  Acceptor exon (score:0.999394): NM_005614.exon2/8
  Translation: full
  5′ gene domains: CBS 6.1e-09
  3′ gene domains: Ras 8.6e-47, Miro 4.9e-16, Arf 6.1e-08
  TIC domains: Ras 7.4e-42, Miro 5.9e-12, Arf 0.00043

```
HBR  CGAGGACGAAG  GGAAATCCTCATTGACGATTCAATTTGTTGAAGGCaAAT
```

- BPTF–KPNA2, +17:63320743..63463688 (distance: 142944 nt)
  Donor exon (score:0.997705):NM_004459.exon9/30, NM_182641.exon7/28
  Acceptor exon (score:0.999364): NM_002266.exon2/11
  Translation: ptc
  5′ gene domains: Bromodomain 2e-25, DDT 4e-21, PHD 8e-10
  3′ gene domains: Arm 7.6e-12, IBB 2.1e-26
  TIC domains: DDT 9.6e-22, PHD 1.1e-08

```
 T1                                                        ACATACCAG  CTTTCTCCCTTTGTCTCATAACCA
 T3                                      GGCGAGAATCTTTAGGACATACCAG  CTTTCTCC
 T3                                                       GACATACCAG  CTTTCTCCCTTTGTCTCATAACC
 N1  GAGTGTGCAGTTAAACCAGTTGTGATGCTACCAATATGGCGAGAATCTTTAGGACATACCAG  CTTTCTCCCTTTG
 N1             TAAACCAGTTGTGATGCTACCAATATGGCGAGAATCTTTAGGACATACCAG  CTTTCTCCCTTTGTCTCATAACCA
 N1                                       GCGAGAATCTTTAGGACATACCAG  CTTTCTCCCTTTGTCTCATAACCATGTCCACCAAtGAGAATGCTAATACAC
 N1                                           GAATCTTTAGGACATACCAG  CTTTCTCCCTTTGTCTCATAACCATGTCCA
 N2                                CTACCAATATGGCGAGAATCTTTAGGACATACCAG  CTTTCTCCCTTTGTC
 N2                                        CGAGAATCTTTAGGACATACCAG  CTTTCTCCCTTTGTCTCATAACCATGT
 N2                                           GAATCTTTAGGACATACCAG  CTTTCTCCCTTTGTCTCATAACCATGTCCA
 N2                                            AATCTTTAGGACATACCAG  CTTTCTCCCTTTGTCTCATAACCATGTCCAC
 N2                                                TTTAGGACATACCAG  CTTTCTCCCTTTGTCTCATAACCATGTCCACCAAt
HBR                           TGTGATGCTgCCAATATGGCGAGAATCTTTAGGACATACCAG  CTTTCTCC
HBR                           TGTGATGCTACCAATATGGCGAGAATCTTTAGGACATACCAG  CTTTCTaC
HBR                                              TCTTTAGGACATACCAG  CTTTCTCCCTTTGTCTCATAACCATGTCCACCA
HBR                                                    GGACATACCAG  CTTTCTCCCTTTGTCTCATAACCATGTCCACCAAtGAGA
UHR                           TGTGATGCTACCAATATGGCGAGAATCTTTAGGACATACCAG  CTTTCTCC
UHR                                  CCAATATGGCGAGAATCTTTAGGACATACCAG  CTTTCTCCCTTTGTCTCA
UHR                                       GCGAGAATCTTTAGGACATACCAG  CTTTCTCCCTTTGTCTCATAACCATG
UHR                                         AGAATCTTTAGGACATACCAG  CTTTCTCCCTTTGTCTCATAACCATGTCC
UHR                                               CTTTAGGACATACCAG  CTTTCTCCCTTTGTCTCATAACCATGTCCACCAA
UHR                                                    GGACATACCAG  CTTTCTCCCTTTGTCTCATAACCATGTCCACCAAtGAGA
UHR                                                     GACATACCAG  CTTTCTCCCTTTGTCTCATAACCATGTCCACCAAtGAGAA
```

- TULP3–TEAD4, +12:2870415..3017395 (distance: 146979 nt)
  Donor exon (score:0.994137):NM_003324.exon1/12
  Acceptor exon (score:0.999821): NM_201443.exon9/11, NM_003213.exon11/13, NM_201441.exon10/12
  Translation: ptc

5′ gene domains: Tub 5.5e-114
3′ gene domains: TEA 2e-105
TIC domains:

```
N3  GCTCAGTCCCAGCGGCGACAG  GCAGACCTCAAC
```

- FERMT3–RPS6KA4, +11:63743406..63892180 (distance: 148773 nt)
  Donor exon (score:0.995770):NM_178443.exon7/15, NM_031471.exon7/15
  Acceptor exon (score:0.995764): NM_001006944.exon10/17, NM_003942.exon10/17
  Translation: full
  5′ gene domains: FERM_M 2.2e-40, PH 2.6e-08
  3′ gene domains: Pkinase 1.6e-65, Pkinase_Tyr 3.4e-30
  TIC domains: FERM_M 2.3e-15, Pkinase 1.6e-64, Pkinase_Tyr 2.9e-30

```
N2  CCGCCCTGCAG  GGATACTCCTTTGTGGCACCCTCCATTCTCTTTGACCAC
```

- CTSC–RAB38, -11:87673346..87522771 (distance: 150574 nt)
  Donor exon (score:0.996998):NM_001814.exon5/7
  Acceptor exon (score:0.996953): NM_022337.exon2/3
  Translation: full
  5′ gene domains: Peptidase_C1 7.4e-63, CathepsinC_exc 3.4e-57
  3′ gene domains: Ras 3.5e-43, Miro 7.7e-18, Arf 1.1e-10, Gtr1_RagA 1e-06
  TIC domains: CathepsinC_exc 2.6e-57, Ras 1.1e-20, Miro 0.00045

```
UHR  TTCGAAACCAAG  GTCAAGAAAGATTTGGAAACATGACGAGGGTCTATTAC
```

- PGAP2–STIM1, +11:3786457..3945358 (distance: 158900 nt)
  Donor exon (score:0.997652):NM_014489.exon1/7
  Acceptor exon (score:0.997212): NM_003156.exon2/12
  Translation: tss
  5′ gene domains: Frag1 1.6e-43
  3′ gene domains: SAM_2 4.9e-07
  TIC domains: SAM_2 4.2e-07

```
HBR  CGCCACCACCGCGTGG  AGTTTTGCCGAATTGACAAGCCCCTGTGTCACAG
```

- FAM122B–PLAC1, -X:133743517..133563818 (distance: 179698 nt)
  Donor exon (score:0.965184):NM_145284.exon8/9
  Acceptor exon (score:0.999574): NM_021796.exon2/3
  Translation: ptc
  5′ gene domains:
  3′ gene domains:
  TIC domains:

```
UHR  GCCGCGCAACTGTCTGATCTCAGTTCATG  CTGCCTTCACCAGTGAGCACA
```

- NOXA1–ARRDC1, +9:139447347..139627888 (distance: 180540 nt)
  Donor exon (score:0.999014):NM_006647.exon9/14
  Acceptor exon (score:0.997839): NM_152285.exon4/8
  Translation: full
  5′ gene domains: PB1 2.2e-08
  3′ gene domains: Arrestin_N 7e-27, Arrestin_C 1.2e-16
  TIC domains: Arrestin_C 2.2e-16, Arrestin_N 1.6e-06

```
HBR  CTgCTCTCTCCCCAG  CCACTGCACCCACGTCCTTTGAGGGTCCTTTCGGG
HBR         CTCCCCAG  CCACTGCACCCACGTCCTTTGAGGGTCCTTTCGGGAAGATCG
```

- DEGS1–CNIH4, +1:222437743..222620204 (distance: 182460 nt)
  Donor exon (score:0.990760):NM_003676.exon1/3
  Acceptor exon (score:0.999208): NM_014184.exon3/5
  Translation: ptc
  5′ gene domains: FA_desaturase 1.1e-24, Lipid_DES 7.4e-22
  3′ gene domains: Cornichon 3.3e-47
  TIC domains: Lipid_DES 2e-11

```
UHR  CGCCGGGAGATCCTGG  TGGGTAATTCCAGAATTGATTGGCCATACCATTG
```

- ELL–KIAA1683, -19:18423362..18239336 (distance: 184025 nt)
  Donor exon (score:0.999462):NM_006532.exon7/12
  Acceptor exon (score:0.998958): NM_025249.exon3/4

Translation: ptc
5′ gene domains: ELL 5.9e-108, Occludin_ELL 1.9e-36
3′ gene domains:
TIC domains: ELL 9.8e-109

```
T3   GCCCCCACAG  GCAGAGCTGACCTGTCCGGTAAT
N2  CGCCCCCACAG  GCAGAGCTGACCTGTCCGGTAATCAAGGCAATGCAGCCG
N2     CCCCACAG  GCAGAGCTGACCTGTCCGGTAATCAAGGCAATGCAGCCGGCC
```

- PPP1CB–WDR43, +2:28828546..29017831 (distance: 189284 nt)
  Donor exon (score:0.998363):NM_002709.exon1/8, NM_206876.exon2/9
  Acceptor exon (score:0.997219): NM_015131.exon15/18
  Translation: ptc
  5′ gene domains: Metallophos 7.5e-46
  3′ gene domains: WD40 4.3e-07, Utp12 2.8e-21
  TIC domains:

```
N2  CCCGGCTGCTGGAGG  TTGCCTGACCTGGTACCCCcGCTGGGGACACTCTA
```

- C6orf153–CUL9, +6:43100848..43298032 (distance: 197183 nt)
  Donor exon (score:0.998942):NM_033112.exon2/7
  Acceptor exon (score:0.994527): NM_015089.exon36/41
  Translation: ptc
  5′ gene domains: DUF947 1.4e-47
  3′ gene domains: Cullin 8e-84, Cul7 1.7e-31, IBR 1.4e-10, APC10 2.2e-08
  TIC domains:

```
N2  CCACCTATCCAAAATGCATGTGTTGCAGATAAGCACAG  GTAAGCAAGGCt
```

- ZNF596–FBXO25, +8:172415..371341 (distance: 198925 nt)
  Donor exon (score:0.998948):NM_173539.exon1/6
  Acceptor exon (score:0.986345): NM_183421.exon3/11, NM_183420.exon3/10, NM_012173.exon3/9
  Translation: tss
  5′ gene domains: KRAB 1.3e-20
  3′ gene domains:
  TIC domains:

```
 T1               CCGGAGCCaAGGTCCGCTCGG  TATCTTAAATAG
 T3                        aAGGTCCGCTCGG  TATCTTAAATAGTGAAGATG
 T3                            GTCCGCTCGG  TATCTTAAATAGTGAAGATGGAG
 N3               CCGGAGCCaAGGTCCGCTCGG  TATCTTAAATAG
HBR  CGGAAACGGATTCTCCGGAGCCaAGGTCCGCTCGG  TATCTTAAATAGTGA
```
