# Supplementary material for: Deep RNA sequencing analysis of readthrough gene fusions in human prostate adenocarcinoma and reference samples
Source: BMC Med Genomics. 2011 Jan 24;4:11. doi: 10.1186/1755-8794-4-11 (PMC3041646; doi:10.1186/1755-8794-4-11)
Supplement: Additional file 3 — TICs with intervening exons. [file 1755-8794-4-11-S3.PDF]

Additional file 3 — TICs with intervening exons

	Upstream-to-intervening				IE	Intervening-to-downstream				
Type	ESTs	Gene	Exon	Distance	Length	Distance	Gene	Exon	ESTs	CDS
3	0	ACAD10 (NM_025247)	20/21	19860	116	6198	ALDH2 (NM_000690)	2/13	1	F,p
3	0	ADCK2 (NM_052853)	7/8	6543	36	5475	NDUFB2 (NM_004546)	2/4	4	p,p
I	8	ARMCX5 (NM_022838)	1/3	5635	172	108129	GPRASP2 (NM_138437)	2/4	4	t,t
I	9	ARMCX5 (NM_022838)	2/3	3973	172	108129	GPRASP2 (NM_138437)	2/4	4	t,t
I	2	BCL2L2 (NM_004050)	3/4	11536	96	2351	PABPN1 (NM_004643)	2/7	1	F,F
I	0	C12orf52 (NM_032848)	3/4	20798	187	18696	TPCN1 (NM_017901)	2/28	0	p,p
3	0	CNPY2 (NM_014255)	5/6	10738	295	13281	CS (NM_004077)	2/11	5	p,p
I	0	CXXC5 (NM_016463)	1/3	73058	65	87424	PSD2 (NM_032289)	2/15	0	t,t
I	0	DET1 (NM_017996)	5/6	5196	155	45659	MRPL46 (NM_022163)	2/4	1	p,p
O	1	EIF3K (NM_013234)	4/8	2401	263	71835	ACTN4 (NM_004924)	2/21	0	F,p
5	0	ESRRA (NM_004451)	6/7	353	293	4950	PRDX5 (NM_012094)	4/6	0	p,p
3	0	FAM116B (NM_001001794)	18/19	18010	53	4129	PLXNB2 (NM_012401)	2/36	4	p,p
3	0	LDLRAD3 (NM_174902)	4/6	295385	166	6986	PRR5L (NM_024841)	3/10	38	p,p
3	0	MBLAC1 (NM_203397)	1/2	21785	205	446	C7orf59 (NM_001008395)	2/4	17	tp,t
5	5	NDUFB8 (NM_005004)	4/5	2260	163	6980	SEC31B (NM_015490)	2/26	0	p,p
3	0	NIT1 (NM_005600)	6/7	33694	145	2880	UFC1 (NM_016406)	2/6	3	F,p
3	0	NIT1 (NM_005600)	6/7	33694	21	3004	UFC1 (NM_016406)	2/6	0	F,p
5	3	NME4 (NM_005009)	4/5	370	186	4662	DECR2 (NM_020664)	2/9	1	F,p
5	0	SF3A2 (NM_007165)	8/9	117	91	2468	AMH (NM_000479)	2/5	0	p,F
I	0	SLC25A27 (NM_004277)	6/9	12734	113	14182	TDRD6 (NM_001010870)	2/4	1	p,p
I	5	SSTR2 (NM_001050)	1/2	22551	189	8480	COG1 (NM_018714)	2/14	5	t,t
I	1	STX16 (NM_003763)	7/8	18013	57	1958	NPEPL1 (NM_024663)	2/12	2	F,F
I	1	STX16 (NM_003763)	7/8	18013	107	1908	NPEPL1 (NM_024663)	2/12	5	F,p
I	2	STXBP1 (NM_003165)	18/20	12434	238	14191	C9orf117 (NM_001012502)	2/9	0	p,p
I	0	TNFAIP8L2 (NM_024575)	1/2	7209	106	2435	SCNM1 (NM_024041)	2/7	0	t,t
I	2	TUT1 (NM_022830)	8/9	1749	232	1935	EEF1G (NM_001404)	2/10	9	p,p
5	1	VAMP8 (NM_003761)	2/3	2644	66	9849	VAMP5 (NM_006634)	2/3	1	F,F
5	0	VAMP8 (NM_003761)	2/3	2587	123	9849	VAMP5 (NM_006634)	2/3	1	F,F
5	0	ZFP41 (NM_173832)	2/3	3654	129	19582	GLI4 (NM_138465)	3/4	0	p,p
I	0	ZNF673 (NM_017776)	5/6	99966	93	34409	CHST7 (NM_019886)	2/2	2	l,l

"Type" indicates location of intervening region. I = Intergenic region, 5 = Last exon of upstream gene, 3 = First exon of downstream gene, O = Other (For *EIF3K–ACTN4*, between exons 4 and 5 of upstream gene). "Distance" indicates length of splice. "ESTs" indicate number of supporting ESTs. IE: Intervening exon. "CDS" shows the coding potential of the TICIE without the intervening exon, followed by the coding potential with the intervening exon: F = Full CDS, t = new TSS preserving $3'$ stop codon, l = $3'$ frameshift extending to last exon, p = $3'$ frameshift with premature termination codon (PTC), tl = new TSS and frameshift extending to last exon; tp = new TSS and frameshift with PTC.
