# Supplementary material for: Deep RNA sequencing analysis of readthrough gene fusions in human prostate adenocarcinoma and reference samples
Source: BMC Med Genomics. 2011 Jan 24;4:11. doi: 10.1186/1755-8794-4-11 (PMC3041646; doi:10.1186/1755-8794-4-11)
Supplement: Additional file 4 — Distant fusions found by spliced alignment approach. [file 1755-8794-4-11-S4.PDF]

Additional file 4 — Distant fusions found by spliced alignment approach

		Donor				Acceptor		
Type	Samples	Gene	Exon	Chr	Chr	Gene	Exon	Distance
L	UHR(1)	C10orf76 (NM_024541)	2/26	−10	−10	KCNIP2 (NM_014591)	4/10	210813
L	HBR(1)	RUNDC2A (NM_032167)	4/8	+16	+16	SNX29 (NM_001080530)	8/14	250488
L	UHR(1)	JAKMIP3 (NM_001105521)	18/24	+10	+10	PWWP2B (NM_138499)	3/3	263070
L	HBR(1)	C10orf26 (NM_001083913)	1/4	+10	+10	CNNM2 (NM_017649)	2/8	305563
L	UHR(1)	TADA2B (NM_152293)	1/2	+4	+4	SORCS2 (NM_020777)	2/27	352438
L	HBR(1)	FAM155B (NM_015686)	1/3	+X	+X	EDA (NM_001399)	2/8	450890
L	N2(1)	NOC4L (NM_024078)	9/15	+12	+12	FBRSL1 (NM_001142641)	6/17	454746
L	HBR(2)	IQCJ (NM_001042705)	4/5	+3	+3	SCHIP1 (NM_014575)	2/8	501759
L	N1(1)	COBL (NM_015198)	1/13	−7	−7	GRB10 (NM_005311)	15/16	721062
L	N1(1)	IER3IP1 (NM_016097)	2/3	−18	−18	ATP5A1 (NM_004046)	3/12	1011989
L	N2(1)	CUGBP2 (NM_006561)	2/14	+10	+10	CDC123 (NM_006023)	7/13	1065301
L	T1(1)	C16orf58 (NM_022744)	12/13	−16	−16	NUPR1 (NM_012385)	2/3	2953853
L	N3(1),T2(5),T3(15)	TMPRSS2 (NM_005656)	1/14	−21	−21	ERG (NM_004449)	4/11	3062463
L	N1(1)	DNAJC2 (NM_014377)	4/17	−7	−7	CYP3A5 (NM_000777)	10/13	3659120
L	UHR(1)	RTTN (NM_173630)	28/49	−18	−18	KDSR (NM_002035)	2/10	6746632
L	HBR(1)	ELAVL3 (NM_001420)	1/7	−19	−19	ZBTB7A (NM_015898)	2/3	7446169
L	HBR(1)	YWHAZ (NM_003406)	2/6	−8	−8	C8orf83 (NM_001171797)	3/5	8031585
L	HBR(1)	IFLTD1 (NM_001145727)	1/8	−12	−12	LMO3 (NM_018640)	3/4	9087955
L	UHR(1)	ELK3 (NM_005230)	4/5	+12	+12	POLR3B (NM_018082)	12/28	10150971
L	UHR(1)	CCDC88A (NM_018084)	2/32	−2	−2	SOS1 (NM_005633)	2/23	16351057
L	N2(1)	SRFBP1 (NM_152546)	1/8	+5	+5	C5orf32 (NM_032412)	2/3	18228552
L	N1(1)	TCF4 (NM_003199)	3/20	−18	−18	C18orf34 (NM_198995)	21/22	22594865
L	UHR(1)	MSL1 (NM_001012241)	3/10	+17	+17	FN3K (NM_022158)	6/6	42765396
L	HBR(1)	NFIX (NM_002501)	2/10	+19	+19	LENG8 (NM_052925)	3/16	46657715
L	HBR(1)	CYB5D2 (NM_144611)	3/4	+17	+17	MRPS7 (NM_015971)	2/5	66765269
L	N1(1)	SMAP1 (NM_021940)	5/10	+6	+6	VTA1 (NM_016485)	2/8	70963896
L	N1(1)	WDR70 (NM_018034)	10/18	+5	+5	COMMD10 (NM_016144)	5/7	77856566
L	HBR(1)	FAM155A (NM_001080396)	1/3	−13	−13	EFHA1 (NM_152726)	2/12	86276947
L	N1(1)	RAI14 (NM_015577)	2/18	+5	+5	C5orf32 (NM_032412)	2/3	104831397
L	N2(1)	LMCD1 (NM_014583)	1/6	+3	+3	MBNL1 (NM_021038)	5/10	145127094
S	UHR(1)	B3GAT3 (NM_012200)	2/5	−11	−11	GANAB (NM_198334)	18/24	6857
S	HBR(1)	RPL18 (NM_000979)	1/7	−19	−19	DBP (NM_001352)	3/4	14515
S	UHR(1)	BRWD1 (NM_018963)	5/42	−21	−21	HMGN1 (NM_004965)	5/6	46843
S	UHR(1)	PAPOLA (NM_032632)	1/22	+14	+14	AK7 (NM_152327)	6/18	64766
S	UHR(1)	RPS6KB1 (NM_003161)	2/15	+17	+17	TMEM49 (NM_030938)	11/12	72317
S	UHR(2)	RPS6KB1 (NM_003161)	4/15	+17	+17	TMEM49 (NM_030938)	12/12	74936
S	UHR(1)	WHSC1L1 (NM_017778)	1/10	−8	−8	FGFR1 (NM_015850)	2/18	75736
S	UHR(1)	SMARCA4 (NM_003072)	4/35	+19	+19	CARM1 (NM_199141)	2/16	81643
S	UHR(1)	DNAH10 (NM_207437)	13/78	+12	+12	TCTN2 (NM_024809)	15/18	94840
S	UHR(1)	USP42 (NM_032172)	3/18	+7	+7	AIMP2 (NM_006303)	2/4	100378
S	UHR(1)	HOMEZ (NM_020834)	1/2	−14	−14	MYH6 (NM_002471)	33/39	100711
S	UHR(1)	ZC3H11A (NM_014827)	7/20	+1	+1	ATP2B4 (NM_001684)	16/21	107874
S	UHR(1)	BCAS3 (NM_017679)	6/24	+17	+17	PPM1D (NM_003620)	2/6	123759
S	UHR(2)	GCN1L1 (NM_006836)	2/58	−12	−12	MSI1 (NM_002442)	12/15	157217
S	N2(1)	GOLGA4 (NM_002078)	1/24	+3	+3	MLH1 (NM_000249)	12/19	217986
S	HBR(1)	DCK (NM_000788)	2/7	+4	+4	RUFY3 (NM_014961)	2/13	235664
S	HBR(1)	C10orf68 (NM_024688)	7/23	+10	+10	CCDC7 (NM_145023)	8/18	239250
S	N2(1)	KIAA1267 (NM_015443)	2/14	−17	−17	ARL17B (NM_001103154)	3/4	258310
S	N3(1)	MECOM (NM_004991)	1/17	−3	−3	PHC3 (NM_024947)	5/15	485909
S	HBR(1)	DNER (NM_139072)	12/13	−2	−2	TRIP12 (NM_004238)	2/41	513256
S	UHR(1)	SAFB (NM_002967)	1/21	+19	+19	KDM4B (NM_015015)	6/23	575919
S	UHR(1)	CHEK2 (NM_007194)	2/15	−22	−22	AP1B1 (NM_001127)	16/23	604732
S	UHR(1)	SAPS3 (NM_018312)	1/23	+11	+11	DPP3 (NM_005700)	17/18	1956213
S	UHR(1)	TANC2 (NM_025185)	2/25	+17	+17	CA4 (NM_000717)	3/8	2916405
S	UHR(1)	TANC2 (NM_025185)	2/25	+17	+17	CA4 (NM_000717)	2/8	2917651
S	HBR(1)	RAB1A (NM_004161)	3/6	−2	−2	PPP3R1 (NM_000945)	2/6	3119159
S	UHR(1)	HMGN1 (NM_004965)	4/6	−21	−21	U2AF1 (NM_006758)	6/8	3745880
S	UHR(1)	KCTD9 (NM_017634)	4/12	−8	−8	STAR (NM_000349)	7/7	12767052
S	N2(1)	CD46 (NM_002389)	1/14	+1	+1	C1orf21 (NM_030806)	3/6	23248933
S	HBR(1)	CNTN1 (NM_001843)	1/24	+12	+12	GAPDH (NM_002046)	7/9	32855926
S	HBR(1)	EXOC2 (NM_018303)	1/28	−6	−6	PLA2G7 (NM_005084)	3/12	46154774
S	N2(1)	PCDHA3 (NM_018906)	1/4	+5	+5	GPR98 (NM_032119)	84/90	49866373
S	HBR(1)	DKK3 (NM_013253)	6/8	−11	−11	PPP1R14B (NM_138689)	4/4	51823794

		Donor				Acceptor		
Type	Samples	Gene	Exon	Chr	Chr	Gene	Exon	Distance
S	UHR(1)	KNTC1 (NM_014708)	42/64	+12	+12	CNOT2 (NM_014515)	4/16	52642609
S	UHR(1)	TANC1 (NM_033394)	2/27	+2	+2	LCLAT1 (NM_182551)	3/7	128963991
S	N2(1)	BTBD9 (NM_052893)	7/12	−6	−6	WDR27 (NM_182552)	24/25	131071602
I	UHR(1)	PLOD1 (NM_000302)	1/19	+1	−1	KIAA2013 (NM_138346)	2/3	11366
I	N1(1)	MRPL47 (NM_020409)	1/7	−3	+3	NDUFB5 (NM_002492)	6/6	19393
I	UHR(1)	HS2ST1 (NM_012262)	1/7	+1	−1	SEP15 (NM_004261)	3/5	34435
I	UHR(1)	USP42 (NM_032172)	1/18	+7	−7	EIF2AK1 (NM_014413)	3/15	54987
I	N2(1)	FAM126B (NM_173822)	1/12	−2	+2	CFLAR (NM_003879)	2/10	58119
I	UHR(1)	ACOT9 (NM_001033583)	1/15	−X	+X	PRDX4 (NM_006406)	4/7	63960
I	UHR(1)	PFDN5 (NM_002624)	2/6	+12	−12	ITGB7 (NM_000889)	6/16	99121
I	UHR(1)	EIF4EBP2 (NM_004096)	1/3	+10	−10	LRRC20 (NM_018205)	4/4	103038
I	N3(1)	C10orf18 (NM_017782)	1/21	+10	−10	GDI2 (NM_001494)	2/11	115530
I	UHR(1)	SLC16A3 (NM_004207)	4/5	+17	−17	CCDC57 (NM_198082)	17/17	136027
I	UHR(1)	VPS53 (NM_018289)	14/18	−17	+17	C17orf97 (NM_001013672)	2/2	147161
I	UHR(1)	SAPS2 (NM_014678)	2/23	+22	−22	SCO2 (NM_005138)	2/2	152274
I	UHR(7)	GAS6 (NM_000820)	12/15	+13	−13	RASA3 (NM_007368)	23/24	185397
I	N1(1)	PSMA5 (NM_002790)	2/9	−1	+1	SARS (NM_006513)	3/11	193508
I	HBR(1)	GALNT9 (NM_001122636)	1/11	−12	+12	NOC4L (NM_024078)	10/15	214146
I	UHR(1)	FAM120B (NM_032448)	1/11	+6	−6	PSMB1 (NM_002793)	2/6	242271
I	T2(1)	BCAP29 (NM_018844)	5/8	+7	−7	COG5 (NM_006348)	12/22	297656
I	UHR(2)	TGOLN2 (NM_006464)	3/4	−2	+2	USP39 (NM_006590)	11/13	320033
I	UHR(1)	MTX2 (NM_006554)	3/10	+2	−2	KIAA1715 (NM_030650)	7/13	333306
I	UHR(1)	DYNC1H1 (NM_001376)	16/78	+14	−14	CINP (NM_032630)	4/5	352774
I	UHR(1)	CTNNB1 (NM_001904)	1/15	+3	−3	ULK4 (NM_017886)	31/37	416047
I	UHR(1)	IL17RA (NM_014339)	1/13	+22	−22	ATP6V1E1 (NM_001696)	5/9	517828
I	UHR(1)	ZKSCAN1 (NM_003439)	1/6	+7	−7	ATP5J2 (NM_004889)	2/4	555533
I	UHR(1)	ARNT (NM_001668)	1/22	−1	+1	MRPS21 (NM_018997)	2/2	568537
I	UHR(1)	RPRD1A (NM_018170)	6/7	−18	+18	FHOD3 (NM_025135)	8/25	575774
I	HBR(1)	PPP1R12A (NM_002480)	1/25	−12	+12	SYT1 (NM_005639)	9/11	581193
I	N1(1)	SPTBN2 (NM_006946)	25/37	−11	+11	SF3B2 (NM_006842)	15/22	630850
I	N1(1)	DARS (NM_001349)	5/16	−2	+2	RAB3GAP1 (NM_012233)	4/24	852380
I	UHR(4)	ARFGEF2 (NM_006420)	1/39	+20	−20	SULF2 (NM_018837)	3/21	1172861
I	HBR(1)	RALGDS (NM_006266)	1/18	−9	+9	BAT2L1 (NM_013318)	31/31	1625232
I	N1(1)	SUMO1 (NM_003352)	1/5	−2	+2	SPATS2L (NM_015535)	4/13	1826129
I	UHR(1)	NACA (NM_005594)	6/8	−12	+12	HNRNPA1 (NM_002136)	6/10	2430489
I	UHR(1)	RASGRF2 (NM_006909)	1/27	+5	−5	AP3B1 (NM_003664)	23/27	2921747
I	N3(1)	FCHO2 (NM_138782)	1/26	+5	−5	AP3B1 (NM_003664)	21/27	5144893
I	HBR(1)	PPM1B (NM_002706)	1/6	+2	−2	HNRPLL (NM_138394)	8/13	5595844
I	UHR(1)	AFTPH (NM_017657)	1/9	+2	−2	RTN4 (NM_007008)	2/7	9536912
I	HBR(1)	TPM4 (NM_003290)	2/8	+19	−19	TUBB4 (NM_006087)	4/4	9606624
I	UHR(1)	KIAA1370 (NM_019600)	2/13	−15	+15	GANC (NM_198141)	5/24	10385270
I	HBR(1)	WIZ (NM_021241)	3/8	−19	+19	KDM4B (NM_015015)	6/23	10410130
I	UHR(2)	LITAF (NM_001136472)	1/4	−16	+16	DECR2 (NM_020664)	2/9	11193286
I	UHR(1)	SYNJ2BP (NM_018373)	1/4	−14	+14	KTN1 (NM_004986)	2/42	14804880
I	HBR(1)	ZBED3 (NM_032367)	1/3	−5	+5	RAB3C (NM_138453)	3/5	18361106
I	HBR(1)	LIMCH1 (NM_014988)	1/27	+4	−4	KCNIP4 (NM_025221)	3/9	20596322
I	N2(1)	PLEKHM3 (NM_001080475)	7/8	−2	+2	NUP35 (NM_138285)	6/9	24703657
I	N2(1)	HK2 (NM_000189)	1/18	+2	−2	RNF149 (NM_173647)	2/7	26362797
I	N1(1)	WIPF1 (NM_001077269)	1/8	−2	+2	ACVR2A (NM_001616)	5/11	26866518
I	N1(1)	ACTR3B (NM_020445)	1/12	+7	−7	POT1 (NM_015450)	3/19	27745008
I	T3(1)	MYH7 (NM_000257)	29/40	−14	+14	KTN1 (NM_004986)	35/42	32249811
I	T3(1)	NBR1 (NM_005899)	15/21	+17	−17	ACTG1 (NM_001614)	4/6	38389113
I	UHR(1)	SERBP1 (NM_015640)	2/8	−1	+1	SPEN (NM_015001)	11/15	51537233
I	UHR(1)	TBC1D15 (NM_022771)	1/18	+12	−12	UBC (NM_021009)	2/2	53444415
I	N1(1)	MTAP (NM_002451)	7/8	+9	−9	RASEF (NM_152573)	2/17	62981232
I	N1(1)	SPATS2 (NM_023071)	2/14	+12	−12	RILPL1 (NM_178314)	5/7	74484965
I	UHR(1)	CDON (NM_016952)	13/20	−11	+11	DGKZ (NM_003646)	24/31	79015326
I	N1(2)	REV1 (NM_016316)	3/23	−2	+2	CPSF3 (NM_016207)	10/18	89944295
I	N1(1)	ACACB (NM_001093)	38/52	+12	−12	CHD4 (NM_001273)	38/40	101615940
I	UHR(1)	C9orf86 (NM_017995)	7/10	+9	−9	VCP (NM_007126)	17/17	103799424
I	HBR(1)	UBR4 (NM_020765)	17/106	−1	+1	CACNA1E (NM_000721)	31/47	160610814
T	HBR(1)	ABCA2 (NM_001606)	1/49	−9	−7	FASTK (NM_006712)	3/10	
T	UHR(1)	ABCC1 (NM_004996)	20/31	+16	−11	RPS25 (NM_001028)	4/5	
T	N1(1)	ABCD3 (NM_002858)	1/23	+1	+6	CDC5L (NM_001253)	15/16	
T	UHR(1)	ABI2 (NM_005759)	1/10	+2	−1	POGZ (NM_015100)	8/19	
T	N2(1)	ABR (NM_001092)	15/22	−17	+2	EIF5B (NM_015904)	2/24	

		Donor			Acceptor			
Type	Samples	Gene	Exon	Chr	Chr	Gene	Exon	Distance
T	UHR(1)	ACP1 (NM_004300)	1/6	+2	−7	NDUFA5 (NM_005000)	3/5	
T	N1(1)	ACTR6 (NM_022496)	4/11	+12	−7	BAIAP2L1 (NM_018842)	4/14	
T	UHR(1)	ADD2 (NM_001617)	14/16	−2	−9	RPS6 (NM_001010)	4/6	
T	UHR(1)	AGRN (NM_198576)	24/36	+1	+19	RPS9 (NM_001013)	5/5	
T	HBR(1)	AKT1 (NM_005163)	4/14	−14	−19	SAFB2 (NM_014649)	21/21	
T	UHR(1)	AMD1 (NM_001634)	1/9	+6	+16	SRCAP (NM_006662)	31/34	
T	UHR(1)	AMMECR1L (NM_031445)	2/8	−2	−11	TRPT1 (NM_001033678)	2/8	
T	HBR(1)	ANK2 (NM_001148)	45/46	+4	+X	PDHA1 (NM_000284)	6/11	
T	UHR(1)	ANKHD1 (NM_017747)	11/34	+5	+1	SCNN1D (NM_002978)	14/14	
T	N1(1)	ANXA4 (NM_001153)	2/13	+2	+7	CALD1 (NM_004342)	13/14	
T	UHR(1)	APEX1 (NM_001641)	2/5	+14	+11	RPL27A (NM_000990)	3/5	
T	UHR(1)	ARPC5L (NM_030978)	2/4	+9	−7	HNRNPA2B1 (NM_002137)	10/11	
T	UHR(1)	ASPH (NM_004318)	3/25	−8	−1	ENO1 (NM_001428)	8/12	
T	T3(1)	ATP5I (NM_007100)	2/4	−4	−17	MTMR4 (NM_004687)	16/19	
T	UHR(1)	B2M (NM_004048)	3/4	+15	+19	ILF3 (NM_004516)	15/18	
T	N1(1)	B3GALNT2 (NM_152490)	2/12	−1	+15	TCF12 (NM_003205)	6/20	
T	N1(1)	B3GALNT2 (NM_152490)	2/12	−1	+X	WDR44 (NM_019045)	13/20	
T	UHR(1)	B3GNTL1 (NM_001009905)	11/13	−17	+20	SLC9A8 (NM_015266)	12/16	
T	N2(1)	BAZ2B (NM_013450)	18/37	−2	−1	IVNS1ABP (NM_006469)	8/15	
T	UHR(1)	BCAP29 (NM_018844)	7/8	+7	+20	DLGAP4 (NM_014902)	7/12	
T	UHR(1)	BCAS3 (NM_017679)	5/24	+17	−1	IVNS1ABP (NM_006469)	8/15	
T	UHR(4)	BCAS4 (NM_017843)	1/6	+20	+17	BCAS3 (NM_017679)	23/24	
T	N2(1)	BCL7A (NM_020993)	1/6	+12	−7	MKRN1 (NM_013446)	3/8	
T	UHR(3)	BCR (NM_004327)	14/23	+22	+9	ABL1 (NM_005157)	2/11	
T	UHR(1)	BRCA1 (NM_007300)	13/24	−17	−5	SPARC (NM_003118)	9/10	
T	HBR(1)	BRWD1 (NM_018963)	4/42	−21	+10	ZMIZ1 (NM_020338)	24/25	
T	T2(1)	C10orf116 (NM_006829)	2/3	+10	−6	C (NM_002117)	8/8	
T	T2(1)	C13orf15 (NM_014059)	1/5	+13	−6	BAT3 (NM_004639)	12/25	
T	HBR(1)	C4orf14 (NM_032313)	6/7	−4	−1	CRTC2 (NM_181715)	12/14	
T	UHR(1)	C9orf16 (NM_024112)	1/2	+9	−5	CCNH (NM_001239)	9/9	
T	N2(1)	C9orf3 (NM_032823)	14/15	+9	−8	SHARPIN (NM_030974)	2/9	
T	UHR(1)	CABIN1 (NM_012295)	29/37	+22	−14	ACIN1 (NM_014977)	9/19	
T	HBR(1)	CACNA1B (NM_000718)	3/47	+9	−17	CYTH1 (NM_004762)	2/14	
T	HBR(1)	CADPS (NM_003716)	28/30	−3	−1	STX6 (NM_005819)	4/8	
T	HBR(1)	CAMKK2 (NM_006549)	16/17	−12	−19	CCDC123 (NM_032816)	19/19	
T	N1(2)	CAMTA1 (NM_015215)	3/23	+1	−12	SPPL3 (NM_139015)	3/11	
T	UHR(1)	CCBL2, RBMXL1 (NM_019610)	1/2	−1	−19	CHERP (NM_006387)	12/17	
T	HBR(1)	CCDC82 (NM_024725)	1/10	−11	+6	NCOA7 (NM_181782)	11/16	
T	T3(1)	CD163 (NM_004244)	13/17	−12	+19	HNRNPUL1 (NM_007040)	14/15	
T	N1(1)	CD46 (NM_002389)	1/14	+1	+20	GNAS (NM_000516)	2/13	
T	HBR(1)	CHD2 (NM_001271)	2/39	+15	+12	FGFR1OP2 (NM_015633)	6/7	
T	T2(1)	CHD2 (NM_001271)	2/39	+15	+1	SFRS11 (NM_004768)	2/13	
T	HBR(1)	CIRH1A (NM_032830)	8/17	+16	−12	FAIM2 (NM_012306)	3/12	
T	UHR(1)	CLDN12 (NM_012129)	1/3	+7	−8	PRKDC (NM_006904)	47/86	
T	HBR(1)	CLK3 (NM_003992)	3/13	+15	−2	BIN1 (NM_004305)	2/15	
T	HBR(1)	CLU (NM_001831)	8/9	−8	−9	PTGES2 (NM_025072)	5/7	
T	UHR(1)	CMPK1 (NM_016308)	5/6	+1	+12	STRAP (NM_007178)	10/10	
T	N2(1)	CNOT1 (NM_016284)	29/49	−16	−10	C10orf4 (NM_145246)	13/14	
T	HBR(1)	CNTN1 (NM_001843)	15/24	+12	+16	FUS (NM_004960)	8/15	
T	HBR(1)	COG4 (NM_015386)	8/19	−16	−12	EEA1 (NM_003566)	2/29	
T	N1(1)	CPSF6 (NM_007007)	9/10	+12	+9	KDM4C (NM_015061)	15/22	
T	HBR(1)	CRY1 (NM_004075)	12/13	−12	−19	SAFB2 (NM_014649)	16/21	
T	N2(1)	CTDSPL (NM_005808)	1/7	+3	+14	YLPM1 (NM_019589)	19/21	
T	N3(1)	CUX1 (NM_001913)	1/23	+7	−6	C6orf106 (NM_022758)	2/4	
T	UHR(1)	DDX5 (NM_004396)	1/13	−17	−16	GALNS (NM_000512)	10/14	
T	UHR(1)	DFFA (NM_004401)	2/6	−1	−20	SULF2 (NM_198596)	19/21	
T	N1(1)	DGKD (NM_152879)	1/30	+2	−18	CYB5A (NM_001914)	5/6	
T	HBR(1)	DNM1 (NM_004408)	9/22	+9	−16	MTSS1L (NM_138383)	13/15	
T	UHR(2)	DYNC1H1 (NM_001376)	24/78	+14	+12	EIF4B (NM_001417)	8/15	
T	UHR(1)	EGFL7 (NM_016215)	2/10	+9	−19	TCF3 (NM_003200)	2/19	
T	N1(1)	ELOVL5 (NM_021814)	3/8	−6	−8	FAM82B (NM_016033)	9/10	
T	HBR(1)	EPB41 (NM_004437)	15/19	+1	+15	USP3 (NM_006537)	2/15	
T	N1(1)	FGFR4 (NM_002011)	2/18	+5	−12	LIMA1 (NM_016357)	7/11	
T	UHR(1)	FNDC3A (NM_001079673)	2/26	+13	+10	VTI1A (NM_145206)	4/8	
T	UHR(1)	FOSL1 (NM_005438)	1/4	−11	+12	PTMS (NM_002824)	2/5	
T	UHR(1)	FTH1 (NM_002032)	1/4	−11	+19	SIN3B (NM_015260)	15/20	

		Donor			Acceptor			
Type	Samples	Gene	Exon	Chr	Chr	Gene	Exon	Distance
T	HBR(1)	GABBR2 (NM_005458)	7/19	−9	−2	CHN1 (NM_001822)	8/13	
T	HBR(1)	GALNTL1 (NM_020692)	14/16	+14	−19	SGTA (NM_003021)	9/12	
T	HBR(1)	GLYCTK (NM_145262)	1/5	+3	−9	EXD3 (NM_017820)	20/22	
T	UHR(1)	GNA12 (NM_007353)	2/4	−7	+1	RNPC3 (NM_017619)	3/3	
T	UHR(1)	GOLGA8B (NM_001023567)	2/16	−15	−12	NAP1L1 (NM_004537)	12/15	
T	HBR(1)	HN1L (NM_144570)	1/5	+16	+22	RBX1 (NM_014248)	3/5	
T	UHR(1)	HNRNPM (NM_031203)	6/17	+19	+14	PABPN1 (NM_004643)	6/7	
T	N1(1)	HOMER2 (NM_004839)	4/9	−15	−2	EXOC6B (NM_015189)	12/22	
T	UHR(1)	HS6ST2 (NM_147175)	3/4	−X	+11	YAP1 (NM_006106)	3/7	
T	HBR(1)	HSP90AA1 (NM_005348)	8/11	−14	−2	DCTN1 (NM_004082)	15/32	
T	HBR(1)	ING3 (NM_019071)	4/12	+7	−16	CHMP1A (NM_002768)	5/7	
T	UHR(1)	INTS1 (NM_001080453)	22/48	−7	+11	RPS6KB2 (NM_003952)	5/15	
T	T1(1)	INTS3 (NM_023015)	15/30	+1	−3	XPC (NM_004628)	9/16	
T	UHR(1)	KDM6A (NM_021140)	2/29	+X	−18	OSBPL1A (NM_018030)	2/14	
T	HBR(1)	KDM6B (NM_001080424)	1/22	+17	+1	SKI (NM_003036)	2/7	
T	T2(1)	KDR (NM_002253)	9/30	−4	−3	RPL29 (NM_000992)	2/4	
T	HBR(1)	KHDRBS3 (NM_006558)	1/9	+8	+12	CABP1 (NM_004276)	2/6	
T	HBR(1)	KIAA1370 (NM_019600)	10/13	−15	+20	PHF20 (NM_016436)	18/18	
T	UHR(1)	KIAA1429 (NM_015496)	7/24	−8	−17	CCDC47 (NM_020198)	5/13	
T	UHR(1)	KRI1 (NM_023008)	12/19	−19	−17	UTP6 (NM_018428)	14/19	
T	N2(1)	LASS4 (NM_024552)	2/12	+19	+2	UBXN4 (NM_014607)	7/13	
T	UHR(1)	TPM4 (NM_003290)	2/8	+19	+X	IGBP1 (NM_001551)	7/7	
T	N1(1)	LOC375190 (NM_001145710)	3/11	+2	−12	MPHOSPH9 (NM_022782)	20/20	
T	N1(1)	LPCAT1 (NM_024830)	7/14	−5	−2	EIF2B4 (NM_015636)	13/13	
T	HBR(1)	LRCH1 (NM_015116)	1/19	+13	−5	FAM13B (NM_016603)	2/23	
T	UHR(1)	LRRC41 (NM_006369)	1/10	−1	+19	DAZAP1 (NM_018959)	2/12	
T	HBR(1)	MAN2A2 (NM_006122)	9/22	+15	−2	ATP5G3 (NM_001689)	2/5	
T	UHR(1)	MAPK14 (NM_001315)	3/12	+6	+16	LCMT1 (NM_016309)	7/11	
T	HBR(1)	MAX (NM_002382)	3/5	−14	+4	ENOPH1 (NM_021204)	2/6	
T	T3(2)	MBTPS1 (NM_003791)	22/23	−16	+15	SERF2 (NM_001018108)	3/3	
T	HBR(1)	ME3 (NM_006680)	8/15	−11	+12	CUX2 (NM_015267)	17/22	
T	HBR(1)	MED10 (NM_032286)	3/4	−5	−2	KIDINS220 (NM_020738)	7/30	
T	N2(1)	MED12L (NM_053002)	14/43	+3	−19	AES (NM_001130)	5/7	
T	UHR(1)	MED1 (NM_004774)	1/17	−17	−12	FMNL3 (NM_175736)	6/26	
T	N2(1)	MOBKL1B (NM_018221)	1/6	−2	−5	PPAP2A (NM_003711)	4/6	
T	HBR(1)	MOG (NM_002433)	2/8	+6	−21	S100B (NM_006272)	3/3	
T	UHR(1)	MYBBP1A (NM_014520)	17/26	−17	−22	RPL3 (NM_000967)	8/10	
T	HBR(1)	MYCBP2 (NM_015057)	81/84	−13	−5	ZFR (NM_016107)	13/20	
T	UHR(1)	MYH9 (NM_002473)	28/41	−22	−9	SDCCAG3 (NM_006643)	7/9	
T	N2(1)	MYO7A (NM_000260)	37/49	+11	−22	DDX17 (NM_006386)	13/13	
T	N1(1)	NAPA (NM_003827)	1/11	−19	+1	RAG1AP1 (NM_018845)	2/6	
T	UHR(1)	NCOR2 (NM_006312)	17/48	−12	+9	C9orf3 (NM_032823)	15/15	
T	N2(1)	NDUFA6 (NM_002490)	1/3	−22	+12	SPATS2 (NM_023071)	10/14	
T	N2(1)	NFRKB (NM_001143835)	2/27	−11	−19	DMKN (NM_033317)	16/16	
T	UHR(1)	NISCH (NM_007184)	3/21	+3	+10	ABCC2 (NM_000392)	20/32	
T	N1(1)	NSUN5 (NM_018044)	4/9	−7	−1	DENND4B (NM_014856)	25/28	
T	UHR(1)	NUP210 (NM_024923)	20/40	−3	+12	SLC25A3 (NM_002635)	4/8	
T	UHR(1)	NUP214 (NM_005085)	29/36	+9	−22	XKR3 (NM_175878)	2/4	
T	HBR(1)	OCIAD1 (NM_017830)	1/9	+4	+16	A2BP1 (NM_018723)	7/18	
T	N3(1)	OGDHL (NM_018245)	12/23	−10	−11	PC (NM_000920)	21/22	
T	N2(2)	OGT (NM_181672)	6/22	+X	−5	RBM22 (NM_018047)	4/11	
T	N1(1)	ORMDL1 (NM_016467)	1/5	−2	+21	COL18A1 (NM_030582)	2/41	
T	UHR(1)	PACRGL (NM_145048)	4/8	+4	+1	UBAP2L (NM_014847)	21/27	
T	N1(1)	PAIP1 (NM_006451)	4/11	−5	+13	MPHOSPH8 (NM_017520)	13/14	
T	HBR(1)	PAK2 (NM_002577)	2/15	+3	−16	FBXO31 (NM_024735)	2/9	
T	UHR(1)	PAPOLA (NM_032632)	4/22	+14	+2	BRE (NM_004899)	9/13	
T	N1(1)	PARD3 (NM_019619)	2/25	−10	+4	ARHGAP10 (NM_024605)	5/23	
T	UHR(1)	PBX3 (NM_006195)	2/9	+9	+16	CHTF18 (NM_022092)	14/22	
T	UHR(1)	PCDHGC3 (NM_032403)	1/4	+5	+7	PCOLCE (NM_002593)	4/9	
T	UHR(1)	PDCD11 (NM_014976)	5/36	+10	+19	SMARCA4 (NM_003072)	30/35	
T	HBR(1)	PDGFRB (NM_002609)	1/23	−5	−7	SCRN1 (NM_014766)	3/8	
T	HBR(1)	PI4KA (NM_058004)	8/55	−22	+2	GCC2 (NM_181453)	10/23	
T	N2(1)	PPP2R1A (NM_014225)	1/15	+19	−12	HDAC7 (NM_015401)	2/26	
T	HBR(1)	PROSC (NM_007198)	4/8	+8	+12	ADIPOR2 (NM_024551)	7/8	
T	HBR(1)	PSMC3 (NM_002804)	2/12	−11	−5	CDH18 (NM_004934)	2/13	
T	UHR(1)	PTDSS2 (NM_030783)	4/12	+11	−16	KIFC3 (NM_005550)	2/19	

		Donor			Acceptor			
Type	Samples	Gene	Exon	Chr	Chr	Gene	Exon	Distance
T	HBR(1)	PTPN2 (NM_002828)	1/9	−18	+10	ZMYND11 (NM_006624)	2/15	
T	UHR(1)	PTPRK (NM_002844)	1/30	−6	+7	FSCN1 (NM_003088)	2/5	
T	UHR(1)	PTRH2 (NM_016077)	1/2	−17	+19	U2AF2 (NM_007279)	8/12	
T	HBR(1)	RABGAP1L (NM_014857)	1/21	+1	−4	PPP3CA (NM_000944)	2/14	
T	UHR(1)	RCN2 (NM_002902)	3/7	+15	+12	ACSS3 (NM_024560)	6/16	
T	HBR(1)	RFC5 (NM_007370)	1/11	+12	+19	HNRNPM (NM_005968)	9/16	
T	HBR(1)	RNF220 (NM_018150)	5/15	+1	+2	PQLC3 (NM_152391)	6/7	
T	N1(2)	ROR2 (NM_004560)	1/9	−9	−17	USP36 (NM_025090)	2/20	
T	N1(1)	RPA1 (NM_002945)	5/17	+17	−1	GNB1 (NM_002074)	2/12	
T	UHR(1)	RPL13A (NM_012423)	1/8	+19	−22	DGCR14 (NM_022719)	9/10	
T	UHR(1)	RPL18A (NM_000980)	2/5	+19	−6	BAT1 (NM_004640)	4/11	
T	UHR(1)	RPL5 (NM_000969)	7/8	+1	+2	QPCT (NM_012413)	7/7	
T	UHR(1)	RPL7A (NM_000972)	2/8	+9	−5	CSNK1A1 (NM_001892)	10/10	
T	T3(1)	RPLP2 (NM_001004)	2/5	+11	+X	PGK1 (NM_000291)	5/11	
T	N1(1)	RPS10 (NM_001014)	4/6	−6	−4	NR3C2 (NM_000901)	9/9	
T	UHR(1)	RPS18 (NM_022551)	3/6	+6	+1	RPL5 (NM_000969)	8/8	
T	UHR(1)	RPS4X (NM_001007)	6/7	−X	+3	RPL15 (NM_002948)	3/4	
T	T3(1)	RPS6KA4 (NM_003942)	4/17	+11	−Y	MAFIP (NM_001033515)	4/4	
T	UHR(1)	RPS6KC1 (NM_012424)	2/15	+1	−4	ANKRD50 (NM_020337)	3/5	
T	N1(1)	SAT1 (NM_002970)	3/6	+X	+1	RNPC3 (NM_017619)	3/3	
T	T3(2)	SEC31A (NM_014933)	1/27	−4	−6	C6orf62 (NM_030939)	2/5	
T	HBR(1)	SEMA4D (NM_006378)	15/18	−9	−6	CCND3 (NM_001760)	3/5	
T	N1(1)	SENP2 (NM_021627)	16/17	+3	+22	RBX1 (NM_014248)	3/5	
T	UHR(1)	SF1 (NM_004630)	2/13	−11	+2	ZNF638 (NM_014497)	11/28	
T	HBR(1)	SHPRH (NM_173082)	25/30	−6	−11	FBXO3 (NM_012175)	11/11	
T	UHR(1)	SKIV2L2 (NM_015360)	1/27	+5	+14	ACTR10 (NM_018477)	11/13	
T	HBR(1)	SLC14A2 (NM_007163)	5/20	+18	+8	RALYL (NM_173848)	2/9	
T	UHR(1)	SLC39A8 (NM_022154)	5/8	−4	+12	SOCS2 (NM_003877)	3/3	
T	UHR(1)	SMARCC2 (NM_003075)	11/28	−12	−1	TOMM20 (NM_014765)	2/5	
T	HBR(1)	SMARCD3 (NM_003078)	2/14	−7	−16	RNPS1 (NM_006711)	4/8	
T	UHR(1)	SNHG3 (NM_001048199)	1/12	+1	+12	DDX11 (NM_004399)	18/26	
T	N1(1)	SNHG3 (NM_001048199)	2/12	+1	−9	AUH (NM_001698)	7/10	
T	UHR(1)	SNHG3 (NM_001048199)	2/12	+1	−8	EEF1D (NM_001960)	2/8	
T	UHR(1)	SNHG4, MATR3 (NM_199189)	3/18	+5	+12	PCBP2 (NM_005016)	7/15	
T	T3(1)	SNRNP25 (NM_024571)	1/5	+16	−1	CAPZB (NM_004930)	9/9	
T	HBR(1)	SNX29 (NM_001080530)	7/14	+16	+13	UFM1 (NM_016617)	3/6	
T	N1(1)	SNX5 (NM_014426)	11/13	−20	+17	DPH1 (NM_001383)	7/13	
T	UHR(1)	SOLH (NM_005632)	2/14	+16	+12	NOC4L (NM_024078)	10/15	
T	HBR(1)	SPOCK3 (NM_016950)	5/12	−4	−13	SMAD9 (NM_005905)	2/6	
T	N2(1)	SSU72 (NM_014188)	1/5	−1	+17	BCAS3 (NM_017679)	3/24	
T	N1(1)	ST6GALNAC1 (NM_018414)	1/9	−17	+20	WFDC2 (NM_006103)	3/4	
T	UHR(1)	ST8SIA4 (NM_005668)	3/5	−5	+17	UBB (NM_018955)	2/2	
T	HBR(1)	STAG3L4 (NM_022906)	4/5	+7	+20	TOX2 (NM_032883)	3/10	
T	UHR(1)	STON2 (NM_033104)	3/5	−14	+9	MURC (NM_001018116)	2/2	
T	N2(1)	TBC1D5 (NM_001134380)	1/23	−3	−6	ECHDC1 (NM_018479)	2/5	
T	T3(1)	TDP1 (NM_018319)	1/17	+14	+11	SAPS3 (NM_018312)	15/23	
T	UHR(1)	TECR (NM_138501)	1/13	+19	+20	GNAS (NM_000516)	2/13	
T	UHR(1)	TIA1 (NM_022037)	4/12	−2	+8	VDAC3 (NM_005662)	10/10	
T	UHR(1)	TIMELESS (NM_003920)	13/29	−12	+19	RPL13A (NM_012423)	2/8	
T	UHR(2)	TIMM9 (NM_012460)	3/6	−14	−8	PRKDC (NM_006904)	26/86	
T	UHR(1)	TMEM223 (NM_001080501)	1/2	−11	+1	PLA2G4A (NM_024420)	14/18	
T	UHR(1)	TMEM49 (NM_030938)	7/12	+17	−1	UBR4 (NM_020765)	95/106	
T	N3(1)	TMPRSS2 (NM_005656)	1/14	−21	−4	CTBP1 (NM_001012614)	2/10	
T	N2(1)	TMPRSS2 (NM_005656)	4/14	−21	−11	C11orf60 (NM_020153)	3/13	
T	HBR(1)	TPCN2 (NM_139075)	7/25	+11	−17	MED24 (NM_014815)	11/26	
T	N2(1)	TRA2A (NM_013293)	2/8	−7	+11	BANF1 (NM_003860)	2/3	
T	UHR(1)	TRIP12 (NM_004238)	1/41	−2	−3	CCDC52 (NM_144718)	4/18	
T	N1(1)	TTBK2 (NM_173500)	3/15	−15	−12	RSRC2 (NM_023012)	7/10	
T	UHR(1)	TTYH1 (NM_020659)	4/14	+19	+1	XKR8 (NM_018053)	2/3	
T	UHR(1)	TULP4 (NM_020245)	6/14	+6	−8	PDE7A (NM_002603)	2/13	
T	N1(1)	TXN (NM_003329)	4/5	−9	−1	TPM3 (NM_152263)	5/10	
T	HBR(1)	UBA1 (NM_003334)	1/26	+X	−15	GATM (NM_001482)	3/9	
T	UHR(1)	UBE2V1 (NM_001032288)	1/4	−20	+17	TBX2 (NM_005994)	2/7	
T	UHR(1)	UCHL3 (NM_006002)	8/9	+13	−20	SYCP2 (NM_014258)	36/44	
T	N2(1)	UGDH (NM_003359)	2/12	−4	−22	PATZ1 (NM_014323)	2/5	
T	HBR(1)	UNC13A (NM_001080421)	16/44	−19	+16	PDIA2 (NM_006849)	2/11	

Type	Samples	Donor Gene	Donor Exon	Donor Chr	Acceptor Chr	Acceptor Gene	Acceptor Exon	Distance
T	UHR(1)	URB1 (NM_014825)	23/39	−21	+6	RPS18 (NM_022551)	4/6	
T	N2(1)	USP11 (NM_004651)	1/21	+X	+7	DNAJB6 (NM_058246)	9/10	
T	UHR(1)	USP18 (NM_017414)	1/11	+22	+2	ZNF638 (NM_014497)	11/28	
T	HBR(1)	USP22 (NM_015276)	5/13	−17	+3	ATP1B3 (NM_001679)	7/7	
T	HBR(1)	VOPP1 (NM_030796)	1/5	−7	−11	PICALM (NM_007166)	2/20	
T	N2(1)	VPS36 (NM_016075)	11/14	−13	−1	RERE (NM_012102)	11/24	
T	N1(1)	WASL (NM_003941)	1/11	−7	−1	EPS15 (NM_001981)	14/25	
T	UHR(1)	WDR27 (NM_182552)	3/25	−6	+5	MAML1 (NM_014757)	2/5	
T	HBR(1)	WDR47 (NM_014969)	13/15	−1	+5	TXNDC15 (NM_024715)	2/5	
T	HBR(1)	XPO7 (NM_015024)	1/28	+8	+17	RAB37 (NM_175738)	2/9	
T	N1(2)	ZDHHC8 (NM_013373)	4/11	+22	+19	UBL5 (NM_024292)	3/5	
T	UHR(1)	ZFYVE21 (NM_024071)	1/7	+14	+13	TFDP1 (NM_007111)	5/12	
T	N1(1)	ZHX2 (NM_014943)	1/4	+8	−7	COG5 (NM_006348)	4/22	
T	UHR(1)	ZNF26 (NM_019591)	2/4	+12	+6	DDX43 (NM_018665)	7/17	
T	HBR(1)	ZNF280D (NM_017661)	16/22	−15	+22	ZNF74 (NM_003426)	5/5	
T	HBR(1)	ZNF382 (NM_032825)	2/5	+19	+9	RABGAP1 (NM_012197)	14/26	
T	N1(1)	ZNF461 (NM_153257)	4/6	−19	−3	RHOA (NM_001664)	4/5	
T	N2(1)	ZNF7 (NM_003416)	4/5	+8	+5	CKMT2 (NM_001825)	8/11	
T	N2(1)	ZNF7 (NM_003416)	4/5	+8	+12	NOC4L (NM_024078)	10/15	

[0] "Type" indicates arrangement of exons. L = long-distance intrachromosomal splicing > 200,000 bp, I = inversion, S = scrambled genes, T = translocation.
