# Supplementary material for: Deep RNA sequencing analysis of readthrough gene fusions in human prostate adenocarcinoma and reference samples
Source: BMC Med Genomics. 2011 Jan 24;4:11. doi: 10.1186/1755-8794-4-11 (PMC3041646; doi:10.1186/1755-8794-4-11)
Supplement: Additional file 5 — Supporting reads for distant fusions. [file 1755-8794-4-11-S5.PDF]

Additional file 5 — Supporting reads for distant fusions

- C10orf76–KCNIP2, -10:103789760..103578946 (distance: 210813 nt)
  Donor exon: NM_024541.exon2/26
  Acceptor exon: NM_173191.exon4/10, NM_014591.exon4/10, NM_173193.exon2/8, NM_173192.exon3/9, NM_173195.exon2/8, NM_173194.exon2/8, NM_173197.exon2/6

```
UHR  TGATGAGATCTTCATG  ACAGCGTGGACGATGAATTTGAATTGTCCACCGT
```

- RUNDC2A–SNX29, +16:12028802..12279291 (distance: 250488 nt)
  Donor exon: NM_032167.exon4/8
  Acceptor exon: NM_001080530.exon8/14

```
HBR  GGGCTTTGCCAGCAAAACCGAAACAG  TGCCAAATCTTTGGAGTGTTGATG
```

- JAKMIP3–PWWP2B, +10:133817484..134080555 (distance: 263070 nt)
  Donor exon: NM_001105521.exon18/24
  Acceptor exon: NM_138499.exon3/3, NM_001098637.exon3/3

```
UHR  CCGGAAACAGGCCTTGGACCAGGCCAACAAG  GTCACCGACGATGAGGGCG
```

- C10orf26–CNNM2, +10:104493890..104799454 (distance: 305563 nt)
  Donor exon: NM_001083913.exon1/4
  Acceptor exon: NM_017649.exon2/8, NM_199076.exon2/7

```
HBR  TCAGCCAGGGCTGAACCCCCGCAG  GTAAATCTCACCTGGCTATCGTGCAG
```

- TADA2B–SORCS2, +4:7096477..7448916 (distance: 352438 nt)
  Donor exon: NM_152293.exon1/2
  Acceptor exon: NM_020777.exon2/27

```
UHR  GCCATCGAGCAGTTCGGCTTCGGAAACTGG  GTGATCTTGATCCTGACGAA
```

- FAM155B–EDA, +X:68642711..69093602 (distance: 450890 nt)
  Donor exon: NM_015686.exon1/3
  Acceptor exon: NM_001399.exon2/8, NM_001005609.exon2/8, NM_001005610.exon2/2, NM_001005612.exon3/3

```
HBR  TCAATCCGGTCCTGCACGAAAGGCTGTAAG  ATGGCCCTATTGAATTTCTT
```

- NOC4L–FBRSL1, +12:131199393..131654140 (distance: 454746 nt)
  Donor exon: NM_024078.exon9/15
  Acceptor exon: NM_001142641.exon6/17

```
 N2  CTCACCCGCGCCTGCGACCTCG  GCATCCGTGGGCTCTGAGAAGCTCTTTG
```

- IQCJ–SCHIP1, +3:160463166..160964926 (distance: 501759 nt)
  Donor exon: NM_001042705.exon4/5
  Acceptor exon: NM_014575.exon2/8

```
HBR  CAGCATGAACACCTTCTCCGACAGCAGCACACCC  GATTACCGAGAGGATG
HBR              CTTCTCCGACAGCAGCACACCC  GATTACCGAGAGGATGGGATGGATCTAG
```

- COBL–GRB10, -7:51351784..50630721 (distance: 721062 nt)
  Donor exon: NM_015198.exon1/13
  Acceptor exon: NM_001001549.exon14/15, NM_001001550.exon15/16, NM_001001555.exon16/17, NM_005311.exon15/16

```
 N1  GCCTCGGCGGCCAAGCCCCCGACCGG  GCTTTTTCTCCTCCGTGACAGCCA
```

- IER3IP1–ATP5A1, -18:42937805..41925815 (distance: 1011989 nt)
  Donor exon: NM_016097.exon2/3
  Acceptor exon: NM_004046.exon3/12, NM_001001937.exon4/13

```
 N1  TCGATCTGTAAGAACCGTGATGAGAG  GGACTGCTGAGATGTCCTCTATTCTTGAAGAGCGTATTCTTGGAGCTGA
```

- CUGBP2–CDC123, +10:11247651..12312953 (distance: 1065301 nt)
  Donor exon: NM_001025077.exon2/13, NM_006561.exon2/14, NM_001025076.exon2/14, NM_001083591.exon2/14
  Acceptor exon: NM_006023.exon7/13

```
 N2  GAACCCTCCGCAGAGTAAAG  GTTTATTCATTGTACTGATGATTCTCCAGA
```

- C16orf58–NUPR1, -16:31410831..28456977 (distance: 2953853 nt)
  Donor exon: NM_022744.exon12/13
  Acceptor exon: NM_012385.exon2/3

```
T1  CCAAAGTTCTTGAAAG  GAGGTGGAGGCCGGAAA
```

- TMPRSS2–ERG, -21:41801878..38739414 (distance: 3062463 nt)
  Donor exon: NM_005656.exon1/14
  Acceptor exon: NM_001136154.exon4/12, NM_182918.exon2/10, NM_004449.exon4/11

```
T2  CGCCTGGAGCGCGGCAG  GAAGCCTTATCAGTTG
T2  CGCCTGGAGCGCGGCAG  GAAGCCTTATCAGTTG
T2  CGCCTGGAGCGCGGCAG  GAAGCCTTATCAGTTG
T2  CGCCTGGAGCGCGGCAG  GAAGCCTTATCAGTTG
T2   GCCTGGAGCGCGGCAG  GAAGCCTTATCAGTTGT
T3  CGCCTGGAGCGCGGCAG  GAAGCCTTATCAGTTG
T3  CGCCTGGAGCGCGGCAG  GAAGCCTTATCAGTTG
T3  CGCCTGGAGCGCGGCAG  GAAGCCTTATCAGTTG
T3  CGCCTGGAGCGCGGCAG  GAAGCCTTATCAGTTG
T3  CGCCTGGAGCGCGGCAG  GAAGCCTTATCAGTTG
T3  CGCCTGGAGCGCGGCAG  GAAGCCTTATCAGTTG
T3  CGCCTGGAGCGCGGCAG  GAAGCCTTATCAGTTG
T3  CGCCTGGAGCGCGGCAG  GAAGCCTTATCAGTTG
T3   GCCTGGAGCGCGGCAG  GAAGCCTTATCAGTTGT
T3   GCCTGGAGCGCGGCAG  GAAGCCTTATCAGTTGT
T3   GCCTGGAGCGCGGCAG  GAAGCCTTATCAGTTGT
T3   GCCTGGAGCGCGGCAG  GAAGCCTTATCAGTTGT
T3   GCCTGGAGCGCGGCAG  GAAGCCTTATCAGTTGT
T3   GCCTGGAGCGCGGCAG  GAAGCCTTATCAGTTGT
T3   GCCTGGAGCGCGGCAG  GAAGCCTTATCAGTTGT
N3   GCCTGGAGCGCGGCAG  GAAGCCTTATCAGTTGT
```

- DNAJC2–CYP3A5, -7:102755339..99096218 (distance: 3659120 nt)
  Donor exon: NM_014377.exon4/17, NM_001129887.exon4/15
  Acceptor exon: NM_000777.exon10/13

```
N1  GAGATAATGACTACTTCACTTGCATAACTAAAG  CTCTGTCTGATCTGGAGCTCGCAGCCCAGTCAATAATCTTCA
```

- RTTN–KDSR, -18:65927714..59181081 (distance: 6746632 nt)
  Donor exon: NM_173630.exon28/49
  Acceptor exon: NM_002035.exon2/10

```
UHR  GTACTTGTCAGGTCTCCTTGAG  GTTACAGGAGGTTCCAGTGGCAgCGGGA
```

- ELAVL3–ZBTB7A, -19:11452415..4006245 (distance: 7446169 nt)
  Donor exon: NM_001420.exon1/7, NM_032281.exon1/7
  Acceptor exon: NM_015898.exon2/3

```
HBR  CCGCGCCCGCCGGGCACACAAGAATGGTCACT  GTCTCGGCGCGGAAGATG
```

- YWHAZ–C8orf83, -8:102030000..93998414 (distance: 8031585 nt)
  Donor exon: NM_003406.exon2/6, NM_145690.exon2/6, NM_001135699.exon2/6, NM_001135700.exon2/6, NM_001135701.exon2/6, NM_001135702.exon2/6
  Acceptor exon: NM_001171799.exon2/4, NM_001171797.exon3/5

```
HBR  CTGCAATGATGTACTG  CAGGCATTTTGGAGATCAAAGATGGGTAGAAAAG
```

- IFLTD1–LMO3, -12:25692695..16604739 (distance: 9087955 nt)
  Donor exon: NM_001145727.exon1/8
  Acceptor exon: NM_001001395.exon3/4, NM_018640.exon3/4

```
HBR  TCGGTGTTTCCAGAGTCCAGACCCACCACA  GCTCTTTGGTGTAACGGGAA
```

- ELK3–POLR3B, +12:95177762..105328734 (distance: 10150971 nt)
  Donor exon: NM_005230.exon4/5
  Acceptor exon: NM_001160708.exon12/28, NM_018082.exon12/28

```
UHR  CAGGCTGCAAGGGCCAAGCACGCTGTTCCAG  GTTAAGGAATTCAATTTCC
```

- CCDC88A–SOS1, -2:55499456..39148398 (distance: 16351057 nt)
  Donor exon: NM_018084.exon2/32, NM_001135597.exon2/33
  Acceptor exon: NM_005633.exon2/23

```
UHR  AACCAGGTCATGCTCCAAAT  GTCCAGGGGCAAGTTCATCCTACTCTCGAG
```

- SRFBP1–C5orf32, +5:121325662..139554215 (distance: 18228552 nt)
Donor exon: NM_152546.exon1/8
Acceptor exon: NM_032412.exon2/3

`N2  CTGAACCTCAATAACGAG  GTGCACTTTACAGGTCCCCGATGAACCAAGAG`

- TCF4–C18orf34, -18:51403509..28808643 (distance: 22594865 nt)
Donor exon: NM_001083962.exon3/20, NM_003199.exon3/20
Acceptor exon: NM_198995.exon21/22, NM_001105528.exon21/22

`N1  GAAAAATGGACCAACTTCTTTGGCAAGTGGACATTTTACTGGCTCAA  CTCTGTCAGCTGCAGAGAAGGATGCACA`

- MSL1–FN3K, +17:35536185..78301582 (distance: 42765396 nt)
Donor exon: NM_001012241.exon3/10
Acceptor exon: NM_022158.exon6/6

`UHR  GGAAAGGGACATAAAAG  GTGAAGATCCCGGATCTGTTTTGTGGCCTAGAG`

- NFIX–LENG8, +19:12997366..59655082 (distance: 46657715 nt)
Donor exon: NM_002501.exon2/10
Acceptor exon: NM_052925.exon3/16

`HBR  ATCTTTATCTGGCTTACTTTGTCCACACTCCGG  GTCTTCTCAGTACAGCA`

- CYB5D2–MRPS7, +17:4004903..70770173 (distance: 66765269 nt)
Donor exon: NM_144611.exon3/4, NR_023346.exon3/4, NR_023347.exon3/4
Acceptor exon: NM_015971.exon2/5

`HBR  GGCAGCCGGCTCTGGTGCTCCCAGAAGAG  GCTAACTCAGGTGAGATGGAG`

- SMAP1–VTA1, +6:71565161..142529058 (distance: 70963896 nt)
Donor exon: NM_021940.exon5/10, NM_001044305.exon6/11
Acceptor exon: NM_016485.exon2/8

`N1  GGCAAAACCACTTACAGCTGAAAAG  GTCGTTTATACGCAATGCAGACTGGAATGAAGATCGATAGTAAAACTCCT`

- WDR70–COMMD10, +5:37641097..115497664 (distance: 77856566 nt)
Donor exon: NM_018034.exon10/18
Acceptor exon: NM_016144.exon5/7

`N1  TGGGACCGAAATTTGACT  CTAGAGACtGTTGGATGGCAGCTTAACCTTCA`

- FAM155A–EFHA1, -13:107316031..21039083 (distance: 86276947 nt)
Donor exon: NM_001080396.exon1/3
Acceptor exon: NM_152726.exon2/12

`HBR  ACTCGGTGAAATCCTGTCCTGAAGACTGTAAG  AAAAATGTTGAACATGGA`

- RAI14–C5orf32, +5:34722817..139554215 (distance: 104831397 nt)
Donor exon: NM_015577.exon2/18, NM_001145520.exon2/18, NM_001145521.exon3/19, NM_001145522.exon2/17, NM_001145525.exo[illegible]
Acceptor exon: NM_032412.exon2/3

`N1  GAAGAGCTTGAAAGCGAAGTTCAGGAAGAGTGAC  GTGCACTTTACAGGTC`

- LMCD1–MBNL1, +3:8518666..153645761 (distance: 145127094 nt)
Donor exon: NM_014583.exon1/6
Acceptor exon: NM_021038.exon5/10, NM_207292.exon5/9, NM_207293.exon4/9, NM_207294.exon3/7, NM_207295.exon3/8, NM_207296.exon4/8, NM_207297.exon4/8

`N2  ACCCAGGAGTTAAAAAG  GTATGTCGAGAGTACCAACGTGGCAATTGCAAC`

- B3GAT3–GANAB, -11:62144545..62151401 (distance: 6857 nt)
Donor exon: NM_012200.exon2/5
Acceptor exon: NM_198334.exon18/24, NM_198335.exon19/25

`UHR  GTTACCCCCACCTATGCCAG  GCCCCTGTGGGTGCAGTACCCTCAGGATGT`

- RPL18–DBP, -19:53814210..53828724 (distance: 14515 nt)
Donor exon: NM_000979.exon1/7
Acceptor exon: NM_001352.exon3/4

`HBR  CAGGAGGCGCCATCATG  GCCTGACCTCTCGGGACACACCCAGCCCTGTGG`

- BRWD1–HMGN1, -21:39592228..39639070 (distance: 46843 nt)
  Donor exon: NM_018963.exon5/42, NM_033656.exon5/41
  Acceptor exon: NM_004965.exon5/6

```
UHR  GGCAGTCTTTGCTACGTACAGCAAAAG  GATAAATCTTCAGACAAAAAAGT
```

- PAPOLA–AK7, +14:96038690..95973925 (distance: 64766 nt)
  Donor exon: NM_032632.exon1/22
  Acceptor exon: NM_152327.exon6/18

```
UHR  GGCGGTGCCGGCGCCGGAGACGATGCCGTT  GCCAGAAAATTTGCAGCATA
```

- RPS6KB1–TMEM49, +17:55342754..55270438 (distance: 72317 nt)
  Donor exon: NM_003161.exon2/15
  Acceptor exon: NM_030938.exon11/12

```
UHR  GGGGAGTTGGACCATATGAACT  TGCTGTCCCCGGCATAGGTCCATCTCTG
```

- RPS6KB1–TMEM49, +17:55346846..55271911 (distance: 74936 nt)
  Donor exon: NM_003161.exon4/15
  Acceptor exon: NM_030938.exon12/12

```
UHR  GCCATGAAGGTGCTTAAAAAG  GGAGAAAACTGGTTGTCCTGGATGTTTGA
UHR     ATGAAGGTGCTTAAAAAG  GGAGAAAACTGGTTGTCCTGGATGTTTGAAAA
```

- WHSC1L1–FGFR1, -8:38358474..38434209 (distance: 75736 nt)
  Donor exon: NM_017778.exon1/10, NM_023034.exon1/24
  Acceptor exon: NM_023110.exon2/18, NM_015850.exon2/18, NM_023105.exon2/17, NM_023106.exon2/17, NM_023107.exon2/6, NM_023108.exon2/6, NM_023111.exon2/18

```
UHR  CACGGCCGGGGGGTTAGAATGGAACAAACTGAAG  GGTCAGTTTGAAAAGG
```

- SMARCA4–CARM1, +19:10958269..10876627 (distance: 81643 nt)
  Donor exon: NM_003072.exon4/35, NM_001128844.exon5/36, NM_001128845.exon3/33, NM_001128846.exon3/33, NM_001128847.exo[illegible]
  NM_001128848.exon3/33, NM_001128849.exon4/36
  Acceptor exon: NM_199141.exon2/16

```
UHR  CCAAATTACAGCAGGCCTCATG  ATGAAGATGTGTGTGTCTTTAAGTGCTC
```

- DNAH10–TCTN2, +12:122849871..122755032 (distance: 94840 nt)
  Donor exon: NM_207437.exon13/78
  Acceptor exon: NM_024809.exon15/18, NM_001143850.exon15/18

```
UHR  CCAGCTCTCATGAAGAAGAGCCTTTTGACCAAG  GTGTAGATGCCCCTGAT
```

- USP42–AIMP2, +7:6121680..6021303 (distance: 100378 nt)
  Donor exon: NM_032172.exon3/18
  Acceptor exon: NM_006303.exon2/4

```
UHR  ATCACATGAACACTCCAAAACAT  GAAGAGTCTAACCTGTCTCTGCAAGCT
```

- HOMEZ–MYH6, -14:22824962..22925672 (distance: 100711 nt)
  Donor exon: NM_020834.exon1/2
  Acceptor exon: NM_002471.exon33/39

```
UHR  CGCCGCCCGGGCTGGACTGCG  GCCTCCCTGGAGCACGAGGAGGGCAAGAT
```

- ZC3H11A–ATP2B4, +1:202064173..201956300 (distance: 107874 nt)
  Donor exon: NM_014827.exon7/20
  Acceptor exon: NM_001001396.exon16/22, NM_001684.exon16/21

```
UHR  TGGCCTTTTCCTACCTCCGAGCAAAA  GGCATCGCAGGCACAGATGTAGCA
```

- BCAS3–PPM1D, +17:56179422..56055664 (distance: 123759 nt)
  Donor exon: NM_017679.exon6/24, NM_001099432.exon6/25
  Acceptor exon: NM_003620.exon2/6

```
UHR  CTTGCCTGCTCCACAGTTTG  CGGAATGGCCAAAGACTATGACGGGTCTTC
```

- GCN1L1–MSI1, -12:119112484..119269700 (distance: 157217 nt)
  Donor exon: NM_006836.exon2/58
  Acceptor exon: NM_002442.exon12/15

```
UHR  GTGAACTTGGGAAGTGTGTTGCTGGAAAAG  CCATTCCTCTCACTGCCTAC
UHR       ACTTGGGAAGTGTGTTGCTGGAAAAG  CCATTCCTCTCACTGCCTACGGAC
```

- GOLGA4–MLH1, +3:37260117..37042132 (distance: 217986 nt)
  Donor exon: NM_002078.exon1/24
  Acceptor exon: NM_000249.exon12/19, NM_001167617.exon12/19, NM_001167618.exon12/19, NM_001167619.exon11/18

```
 N2  CGCTGGCTCCTGCTCAG  ACTTTGCTACCAGGACTTGCTGGCCCCTCTGGG
```

- DCK–RUFY3, +4:72082763..71847100 (distance: 235664 nt)
  Donor exon: NM_000788.exon2/7
  Acceptor exon: NM_001037442.exon2/18, NM_014961.exon2/13, NM_001130709.exon2/12

```
HBR  GTTCAAAGTACTCAAGATGAATTTGAG  ATCCTAATTATCTCATGGCTAAT
```

- C10orf68–CCDC7, +10:33040664..32801415 (distance: 239250 nt)
  Donor exon: NM_024688.exon7/23
  Acceptor exon: NM_145023.exon8/18, NM_001026383.exon8/18

```
HBR  CTGAAGGAGAAGGACGTAGCA  GGATAAAGAAAATCGACCAGAAGCAGTGA
```

- KIAA1267–LOC100294341—ARL17B, -17:41527743..41786052 (distance: 258310 nt)
  Donor exon: NM_015443.exon2/14
  Acceptor exon: XM_002344068.exon3/4, NM_001103154.exon3/4

```
 N2  ACAGATACGTGCTAATAAG  GTTTCTGTGTGGAGACAGTAGAATATAAAAA
```

- MECOM–PHC3, -3:170863818..171349726 (distance: 485909 nt)
  Donor exon: NM_004991.exon1/17
  Acceptor exon: NM_024947.exon5/15

```
 N3  AAGGAAACTGGCCACAA  ATCAACCTCTCCACTT
```

- DNER–TRIP12, -2:229939833..230453088 (distance: 513256 nt)
  Donor exon: NM_139072.exon12/13
  Acceptor exon: NM_004238.exon2/41

```
HBR  CAGCAATGCCATTGCATCCATCCGGCATGCCAG  TGAATGGTAGTGTCTAG
```

- SAFB–KDM4B, +19:5574405..4998487 (distance: 575919 nt)
  Donor exon: NM_002967.exon1/21
  Acceptor exon: NM_015015.exon6/23

```
UHR  AGCGTTTTGATGGAGCGGCTGAAGAAG  GACGTGGCCCAGTGGAACATCGG
```

- CHEK2–AP1B1, -22:27460391..28065122 (distance: 604732 nt)
  Donor exon: NM_007194.exon2/15, NM_145862.exon2/14, NM_001005735.exon2/16
  Acceptor exon: NM_145730.exon15/21, NM_001127.exon16/23, NM_001166019.exon15/21

```
UHR  ATGGATTTGCCAATCTTG  ATTGGGGGCACCAACTTCGTGGCACCTCCAAC
```

- SAPS3–DPP3, +11:67984871..66028659 (distance: 1956213 nt)
  Donor exon: NM_018312.exon1/23, NM_001164160.exon1/25, NM_001164161.exon1/24, NM_001164162.exon1/24, NM_001164163.exo
  NM_001164164.exon1/23
  Acceptor exon: NM_005700.exon17/18, NM_130443.exon17/18

```
UHR  GCGGCGGGTCGTGGGCACCTCCAG  GTGCTGAAGTCCACAGGGGATGTGGC
```

- TANC2–CA4, +17:58505107..55588703 (distance: 2916405 nt)
  Donor exon: NM_025185.exon2/25
  Acceptor exon: NM_000717.exon3/8

```
UHR  CGCCAAAGCCGCTCTGGGCAAG  TGCCAGTCAAGTGGGGTGGAAACTGCCA
```

- TANC2–CA4, +17:58505107..55587457 (distance: 2917651 nt)
  Donor exon: NM_025185.exon2/25
  Acceptor exon: NM_000717.exon2/8

```
UHR  CAAAGCCGCTCTGGGCAAG  AGTCACACTGGTGCTACGAGGTTCAAGCCGA
```

- RAB1A–PPP3R1, -2:65178609..68297767 (distance: 3119159 nt)
  Donor exon: NM_015543.exon3/4, NM_004161.exon3/6
  Acceptor exon: NM_000945.exon2/6

```
HBR  AAACAATCAAGCTTCAAATA  GGAAATGAGGCAAGTTATCCTTTGGAAATG
```

- HMGN1–U2AF1, -21:39642088..43387967 (distance: 3745880 nt)
  Donor exon: NM_004965.exon4/6
  Acceptor exon: NM_006758.exon6/8, NM_001025203.exon6/8, NM_001025204.exon7/9

  `UHR  AGCGAAGCCGAAAAAGGCAGCAGCGAAG  TTTCGCCGTGAGGAAGATGCGG`

- KCTD9–STAR, -8:25354010..38121061 (distance: 12767052 nt)
  Donor exon: NM_017634.exon4/12
  Acceptor exon: NM_000349.exon7/7, NM_001007243.exon8/8

  `UHR  GAGGGCGGTACTTTACAACTACACG  GGGTGGCTGCCCAAGAGCATCATCA`

- CD46–C1orf21, +1:205992277..182743345 (distance: 23248933 nt)
  Donor exon: NM_172359.exon1/13, NM_172360.exon1/12, NM_172361.exon1/10, NM_002389.exon1/14, NM_172350.exon1/10, NM_172351.exon1/13, NM_172352.exon1/12, NM_172353.exon1/11, NM_172354.exon1/13, NM_172355.exon1/12, NM_172356.exon1/[illegible] NM_172357.exon1/11, NM_172358.exon1/12, NM_153826.exon1/12
  Acceptor exon: NM_030806.exon3/6

  `N2  TGCTGCTGTACTCCTTCTCCG  ATGAGTATAGGATCAAACCAGTGGAAGAG`

- CNTN1–GAPDH, +12:39372661..6516736 (distance: 32855926 nt)
  Donor exon: NM_175038.exon1/23, NM_001843.exon1/24
  Acceptor exon: NM_002046.exon7/9

  `HBR  GTGCCGCACCGAGGCGAGCAGGAGCAGGGAACAG  CAATGCCTCCTGCACC`

- EXOC2–PLA2G7, -6:638019..46792792 (distance: 46154774 nt)
  Donor exon: NM_018303.exon1/28
  Acceptor exon: NM_005084.exon3/12, NM_001168357.exon3/12

  `HBR  GGCCGTGAGCTGCGGCAGGACGGCTGGAG  CATGGGTCAACAAAATACAAG`

- PCDHA3–GPR98, +5:140163360..90296988 (distance: 49866373 nt)
  Donor exon: NM_018906.exon1/4
  Acceptor exon: NM_032119.exon84/90, NR_003149.exon84/90

  `N2  TTGATCTCTCAGCCAAA  ATTCTGTTTCTGGCGTCTGCATACGCAAGTCCC`

- DKK3–LOC100293737—PPP1R14B, -11:11945077..63768870 (distance: 51823794 nt)
  Donor exon: NM_015881.exon6/8, NM_013253.exon6/8, NM_001018057.exon5/7
  Acceptor exon: XM_002344574.exon4/4, NM_138689.exon4/4

  `HBR  GCCGGGGCTGTGCTGTGCCTTCCAGAGAG  GCCTTCATTTCTGGCCTGCTG`

- KNTC1–CNOT2, +12:121641953..68999345 (distance: 52642609 nt)
  Donor exon: NM_014708.exon42/64
  Acceptor exon: NM_014515.exon4/16

  `UHR  CCCAGTGGGGCATTCGTCTTGGTAAACTTGGT  ATGCTGGCATCACCATCT`

- TANC1–LCLAT1, +2:159565947..30601957 (distance: 128963991 nt)
  Donor exon: NM_033394.exon2/27, NM_001145909.exon2/27
  Acceptor exon: NM_001002257.exon2/6, NM_182551.exon3/7

  `UHR  AAGTTATCAAAAACAG  AATCATGGTGTCATGGAAAGGGATTTACTTTATA`

- BTBD9–WDR27, -6:38653354..169724955 (distance: 131071602 nt)
  Donor exon: NM_052893.exon7/12, NM_001099272.exon6/11, NM_152733.exon5/10
  Acceptor exon: NM_182552.exon24/25

  `N2  ATATTTTCCAGCCCGTGTCTGCAG  GCTTACGTGTATGAAATGGGCTCAAG`

- PLOD1–KIAA2013, +1:11917499..-1:11906133 (distance: 11366 nt)
  Donor exon: NM_000302.exon1/19
  Acceptor exon: NM_138346.exon2/3

  `UHR  AGCGAAGGGCGACGCCAAGCCGGAGG  GAGTGGAAATGAAGAAGATCACTG`

- MRPL47–NDUFB5, -3:180805009..+3:180824402 (distance: 19393 nt)
  Donor exon: NM_177988.exon1/6, NM_020409.exon1/7
  Acceptor exon: NM_002492.exon6/6

  `N1  CTGAAATCTTCCCGATCGTTAATAACTCCTCAGGTCCCTGCCTGCACAGG  GGTAAAGGAGCTGGAAGTGCGAAAA`

- HS2ST1–SEP15, +1:87153431..-1:87118996 (distance: 34435 nt)
  Donor exon: NM_012262.exon1/7, NM_001134492.exon1/5
  Acceptor exon: NM_004261.exon3/5, NM_203341.exon3/4

```
UHR  AGGAGTCCCGCTCGAAGCTAG  CTGTATGCAGGAGCTATTCTTGAAGTTTG
```

- USP42–EIF2AK1, +7:6111189..-7:6056202 (distance: 54987 nt)
  Donor exon: NM_032172.exon1/18
  Acceptor exon: NM_014413.exon3/15, NM_001134335.exon3/15

```
UHR  CGAGGGGGATGGAGCGAGCGCCGAGCCGGGTCAG  TACTTTGCCAGACGTT
```

- FAM126B–CFLAR, -2:201644578..+2:201702697 (distance: 58119 nt)
  Donor exon: NM_173822.exon1/12
  Acceptor exon: NM_003879.exon2/10, NM_001127183.exon2/10, NM_001127184.exon2/6

```
 N2  GCCGGAGACAGCAGGGTGACAG  AACTCCCCCACTGGAAAGGATTCTGAAA
```

- ACOT9–PRDX4, -X:23671163..+X:23607203 (distance: 63960 nt)
  Donor exon: NM_001033583.exon1/15, NM_001037171.exon1/16
  Acceptor exon: NM_006406.exon4/7

```
UHR  TGAGGCGGGCAGCACTGCG  GATTAATACCCCTCGAAGACAAGGAGGACTT
```

- PFDN5–ITGB7, +12:51975992..-12:51876871 (distance: 99121 nt)
  Donor exon: NM_002624.exon2/6
  Acceptor exon: NM_000889.exon6/16

```
UHR  GTCTGAACGTGCTGAACAAGAGCAACGAGG  GTTTTGGTTCCTTTGTGGAC
```

- EIF4EBP2–LRRC20, +10:71834308..-10:71731270 (distance: 103038 nt)
  Donor exon: NM_004096.exon1/3
  Acceptor exon: NM_018205.exon4/4, NM_018239.exon4/4, NM_207119.exon5/5

```
UHR  CTCCACCACACCGGGAG  ATGTGCCCGTGGAGAAGCTGGCCGCCATGCCAG
```

- C10orf18–GDI2, +10:5767144..-10:5882674 (distance: 115530 nt)
  Donor exon: NM_017782.exon1/21
  Acceptor exon: NM_001494.exon2/11, NM_001115156.exon2/10

```
 N3  GCCACGCCAAGGACGG  GAATGTATCCTGTCAGG
```

- SLC16A3–CCDC57, +17:77789058..-17:77653031 (distance: 136027 nt)
  Donor exon: NM_004207.exon4/5, NM_001042422.exon4/5, NM_001042423.exon4/5
  Acceptor exon: NM_198082.exon17/17

```
UHR  CGGTGGCCGTGCTCGTCGGGCCCCCTTCGGGAG  AGCTCCCAGCTCCTCCA
```

- VPS53–C17orf97, -17:410416..+17:263255 (distance: 147161 nt)
  Donor exon: NM_018289.exon14/18, NM_001128159.exon15/22
  Acceptor exon: NM_001013672.exon2/2

```
UHR  TACTGTCTGGCCACCACCCAGCAG  ATAAACATCAGAGTCAGAGCCTGAAG
```

- SAPS2–SCO2, +22:49157445..-22:49309719 (distance: 152274 nt)
  Donor exon: NM_014678.exon2/23
  Acceptor exon: NM_001169109.exon2/2, NM_001169110.exon2/2, NM_001169111.exon2/2, NM_005138.exon2/2

```
UHR  CAAGAGATTTCCACAGAAG  GAGCATCAGATCCATGCTGCTGCTGACTCGG
```

- GAS6–RASA3, +13:113583974..-13:113769371 (distance: 185397 nt)
  Donor exon: NM_000820.exon12/15, NM_001143945.exon6/9, NM_001143946.exon4/7
  Acceptor exon: NM_007368.exon23/24

```
UHR  GAGCGGCTTCGCCTTCTACAGCCTGGACTACA  AGGCCTGTGGGAGCAAAT
UHR    GCGGCTTCGCCTTCTACAGCCTGGACTACA  AGGCCTGTGGGAGCAAATCT
UHR    GCGGCTTCGCCTTCTACAGCCTGGACTACA  AGGCCTGTGGGAGCAAATCT
UHR       GCTTCGCCTTCTACAGCCTGGACTACA  AGGCCTGTGGGAGCAAATCTGTG
UHR            GCCTTCTACAGCCTGGACTACA  AGGCCTGTGGGAGCAAATCTGTGTATGA
UHR            GCCTTCTACAGCCTGGACTACA  AGGCCTGTGGGAGCAAATCTGTGTATGA
UHR                 CTACAGCCTGGACTACA  AGGCCTGTGGGAGCAAATCTGTGTATGACGGCC
```

- PSMA5–SARS, -1:109766005..+1:109572497 (distance: 193508 nt)
  Donor exon: NM_002790.exon2/9
  Acceptor exon: NM_006513.exon3/11

N1 TTTCAAGTGGAATATGCCATTGAGGCTATCAAG AAAAAAGAGCCAGTGGGAGATGATGAGTCTGTCCCAGAGAAT

- GALNT9–NOC4L, -12:131415625..+12:131201479 (distance: 214146 nt)
  Donor exon: NM_001122636.exon1/11
  Acceptor exon: NM_024078.exon10/15

HBR CTACAACCAGCTCAACG GGGGGGCCCTCAGCCTCTTGGCCTTGAACGGGC

- FAM120B–PSMB1, +6:170457855..-6:170700126 (distance: 242271 nt)
  Donor exon: NM_032448.exon1/11
  Acceptor exon: NM_002793.exon2/6

UHR CGCGTCTGCGGCTGGTGTTGGCGCATCTCTAG TACTATACTGGCAATTGC

- BCAP29–COG5, +7:107023683..-7:106726027 (distance: 297656 nt)
  Donor exon: NM_018844.exon5/8, NM_001008405.exon4/8, NR_027830.exon4/7
  Acceptor exon: NM_006348.exon12/22, NM_181733.exon12/21, NM_001161520.exon12/21

T2 CGAAAAACTAAAAAGG CTTCGATGTTTTTGAAG

- TGOLN2–USP39, -2:85405549..+2:85725582 (distance: 320033 nt)
  Donor exon: NM_006464.exon3/4
  Acceptor exon: NM_006590.exon11/13

UHR CAACGTTTGGACCAGAAG AAATGTGGATCTGAGAGAATACTTGTCTGAAG
UHR CAACGTTTGGACCAGAAG AAATGTGGATCTGAGAGAATACTTGTCTGAAG

- MTX2–KIAA1715, +2:176870869..-2:176537563 (distance: 333306 nt)
  Donor exon: NM_006554.exon3/10, NR_027850.exon4/11
  Acceptor exon: NM_030650.exon7/13

UHR CAGCTTCTCTTGCAGTGCAG CTTGAAGAAGTCATGGAAAAAGAAACTTAC

- DYNC1H1–CINP, +14:101533364..-14:101886138 (distance: 352774 nt)
  Donor exon: NM_001376.exon16/78
  Acceptor exon: NM_032630.exon4/5

UHR GACCAAGCCTGTCACG ACCAAAATACAGGTGAAAATGGAAAAGCTGTCTT

- CTNNB1–ULK4, +3:41216165..-3:41632212 (distance: 416047 nt)
  Donor exon: NM_001904.exon1/15, NM_001098209.exon1/16, NM_001098210.exon1/16
  Acceptor exon: NM_017886.exon31/37

UHR CCCGCGGCGGGAGGAGCCTGTTCCCCTGAG ACTTGTGGAAGAAAGCAAAC

- IL17RA–ATP6V1E1, +22:15946119..-22:16463947 (distance: 517828 nt)
  Donor exon: NM_014339.exon1/13
  Acceptor exon: NM_001696.exon5/9, NM_001039366.exon4/8

UHR CGCTGGTCTGCTCCCAGCCG GACCTACTAAATGAAGCAAAACAGAGACTC

- ZKSCAN1–ATP5J2, +7:99451285..-7:98895752 (distance: 555533 nt)
  Donor exon: NM_003439.exon1/6
  Acceptor exon: NM_004889.exon2/4, NM_001003713.exon2/4, NM_001003714.exon2/3, NM_001039178.exon2/3

UHR CCAGGCCGCTCCCGGGGCTCACG TACCAGTGAAGGACAAGAAACTTCTGG

- ARNT–MRPS21, -1:149115643..+1:148547106 (distance: 568537 nt)
  Donor exon: NM_001668.exon1/22, NM_178426.exon1/13, NM_178427.exon1/21
  Acceptor exon: NM_018997.exon2/2, NM_031901.exon3/3

UHR GCGGCGACTACTGCCAACCCCG AATCCTCACTATGGATGGGCTCATTGAG

- RPRD1A–FHOD3, -18:31860861..+18:32436635 (distance: 575774 nt)
  Donor exon: NM_018170.exon6/7
  Acceptor exon: NM_025135.exon8/25

UHR AAAGAGCATAAATTGGAA GGGTCAAACCTTGGTCAAATATCATGGAAATC

- PPP1R12A–SYT1, -12:78852606..+12:78271413 (distance: 581193 nt)
  Donor exon: NM_002480.exon1/25, NM_001143885.exon2/26
  Acceptor exon: NM_001135805.exon10/12, NM_005639.exon9/11, NM_001135806.exon8/10

HBR CGCCAATGTGGACGGACTCACTGCCCTGCACCAG CAAGAGAAATTGGGTG

- SPTBN2–SF3B2, -11:66216583..+11:65585733 (distance: 630850 nt)
  Donor exon: NM_006946.exon25/37
  Acceptor exon: NM_006842.exon15/22

```
 N1  GTGGgGGCGGCCTCCCACGAGCTGGGCCAGGACTACGAGCATGTGACT  GGGAAGGAGTTCGAGACACGACTGAAG
```

- DARS–RAB3GAP1, -2:136417418..+2:135565038 (distance: 852380 nt)
  Donor exon: NM_001349.exon5/16
  Acceptor exon: NM_012233.exon4/24

```
 N1  GAAAATTGGAAGCTGTACACAGCAAGACGTTGAGTTACATGTTCAGAAG  GGTATATTTACTTCTGGCACATGGGA
```

- ARFGEF2–SULF2, +20:46971954..-20:45799093 (distance: 1172861 nt)
  Donor exon: NM_006420.exon1/39
  Acceptor exon: NM_018837.exon3/21, NM_001161841.exon3/21, NM_198596.exon3/21

```
UHR  TGCGCAGGGCCTGCCAGGTGGCGCTCG  GTTCCATGCAGGTGATGAACAAG
UHR        GGGCCTGCCAGGTGGCGCTCG  GTTCCATGCAGGTGATGAACAAGACCCGG
UHR         GGCCTGCCAGGTGGCGCTCG  GTTCCATGCAGGTGATGAACAAGACCCGGC
UHR         GGCCTGCCAGGTGGCGCTCG  GTTCCATGCAGGTGATGAACAAGACCCGGC
```

- RALGDS–BAT2L1, -9:134986180..+9:133360948 (distance: 1625232 nt)
  Donor exon: NM_006266.exon1/18
  Acceptor exon: NM_013318.exon31/31

```
HBR  CCTGCCGCGCCCGGAG  GCAAAACAACGAGTGGATGAGAAACCCAGCCTGG
```

- SUMO1–SPATS2L, -2:202811408..+2:200985279 (distance: 1826129 nt)
  Donor exon: NM_003352.exon1/5, NM_001005781.exon1/6, NM_001005782.exon1/4
  Acceptor exon: NM_015535.exon4/13, NM_001100422.exon4/13, NM_001100423.exon4/13, NM_001100424.exon4/12

```
 N1  GCCACCGTCATCATGTCTGACCAG  ATCTATGCAGTTAGATCAGTTGTTCCCAACAAAAGCAATAATGAAATAGTC
```

- NACA–HNRNPA1, -12:55393113..+12:52962624 (distance: 2430489 nt)
  Donor exon: NM_005594.exon6/8, NM_001113201.exon6/8, NM_001113202.exon6/8, NM_001113203.exon7/9
  Acceptor exon: NM_002136.exon6/10, NM_031157.exon6/11

```
UHR  AGAGTGAAGAGGAAGAG  GTCGAAGTGGTTCTGGAAACTTTGGTGGTGGTC
```

- RASGRF2–AP3B1, +5:80292601..-5:77370854 (distance: 2921747 nt)
  Donor exon: NM_006909.exon1/27
  Acceptor exon: NM_003664.exon23/27

```
UHR  CGAGACGCGCTGGACAAGCAG  GTCAGTACTCCTGCATTTGTACCAACGAA
```

- FCHO2–AP3B1, +5:72287712..-5:77432605 (distance: 5144893 nt)
  Donor exon: NM_001146032.exon1/25, NM_138782.exon1/26
  Acceptor exon: NM_003664.exon21/27

```
 N3  CGTCGAGAATTTTTGG  GAGAAAGAAAAGAAAAC
```

- PPM1B–HNRPLL, +2:44249917..-2:38654073 (distance: 5595844 nt)
  Donor exon: NM_002706.exon1/6, NM_177969.exon1/5, NM_001033557.exon1/6, NM_001033556.exon1/3, NM_177968.exon1/6
  Acceptor exon: NM_138394.exon8/13, NM_001142650.exon9/14

```
HBR  GCGCCCTAGACATCTTCTCCCTCCCTTGCCTCAG  GATCCCATGGTCCATT
```

- AFTPH–RTN4, +2:64605250..-2:55068338 (distance: 9536912 nt)
  Donor exon: NM_017657.exon1/9, NM_001002243.exon1/9, NM_203437.exon1/10
  Acceptor exon: NM_020532.exon4/9, NM_207520.exon3/8, NM_153828.exon2/7, NM_007008.exon2/7, NM_207521.exon4/9

```
UHR  GCAGCCGCCCCGAAGGAGCCAG  TTGTTGACCTCCTGTACTGGAGAGACAT
```

- LOC100129096—TPM4–TUBB4, +19:16053856..-19:6447232 (distance: 9606624 nt)
  Donor exon: XR_079235.exon2/8, NM_003290.exon2/8, NM_001145160.exon3/9
  Acceptor exon: NM_006087.exon4/4

```
HBR  AAAAGCTGCAGATGAGAGTGAGAG  GCCAATCCGGAGCCGGCAACAACTGG
```

- KIAA1370–GANC, -15:50757495..+15:40372225 (distance: 10385270 nt)
  Donor exon: NM_019600.exon2/13
  Acceptor exon: NM_198141.exon5/24

```
UHR  CGAAAATTGGATGTGAAAATGAAGCCAGACCGAG  GCTGATTTCATGCTCT
```

- WIZ–KDM4B, -19:15408617..+19:4998487 (distance: 10410130 nt)
  Donor exon: NM_021241.exon3/8
  Acceptor exon: NM_015015.exon6/23

```
HBR  GGCCAGCCTATGGAGATG  GACGTGGCCCAGTGGAACATCGGGAGCCTCCG
```

- LITAF–DECR2, -16:11588243..+16:394957 (distance: 11193286 nt)
  Donor exon: NM_001136472.exon1/4, NM_001136473.exon1/5
  Acceptor exon: NM_020664.exon2/9

```
UHR  CCCGGCTCGACAGGCGGCGCGGGCGGCG  GGACAAAGTGGCCTTCATCACA
UHR              GGCGGCGCGGGCGGCG  GGACAAAGTGGCCTTCATCACAGGAGGCGGCTCT
```

- SYNJ2BP–KTN1, -14:69953370..+14:55148490 (distance: 14804880 nt)
  Donor exon: NM_018373.exon1/4
  Acceptor exon: NM_182926.exon3/46, NM_004986.exon2/42, NM_001079521.exon2/44, NM_001079522.exon3/43

```
UHR  TCTTACCAGAGGGCCCTCAG  GTgTTATAGGATCACATTGACAAAAGTACC
```

- ZBED3–RAB3C, -5:76418692..+5:58057586 (distance: 18361106 nt)
  Donor exon: NM_032367.exon1/3
  Acceptor exon: NM_138453.exon3/5

```
HBR  TCCGGCCGCGTTTCCCGGGTAAAGGG  GACACAGCAGGCCAGGAAAGATAC
```

- LIMCH1–KCNIP4, +4:41057710..-4:20461388 (distance: 20596322 nt)
  Donor exon: NM_014988.exon1/27, NM_001112717.exon1/26, NM_001112718.exon1/26
  Acceptor exon: NM_025221.exon3/9

```
HBR  CTTCTCCGAGGCGCAGAAGTGGATTGAG  ACAGCGTGGAAGATGAACTGGA
```

- PLEKHM3–NUP35, -2:208434074..+2:183730417 (distance: 24703657 nt)
  Donor exon: NM_001080475.exon7/8
  Acceptor exon: NM_138285.exon6/9

```
 N2  GGATATTTCAACAAGCAG  GTTTCCTCAAGCATCTGCTTCCTACATATTAC
```

- HK2–RNF149, +2:74915278..-2:101278075 (distance: 26362797 nt)
  Donor exon: NM_000189.exon1/18
  Acceptor exon: NM_173647.exon2/7

```
 N2  CACGGAGCTCAACCATGACCAAGTGCAGAAG  GAACAGGAAATATAGTGGT
```

- WIPF1–ACVR2A, -2:175255748..+2:148389230 (distance: 26866518 nt)
  Donor exon: NM_001077269.exon1/8
  Acceptor exon: NM_001616.exon5/11

```
 N1  CGGAGTCGTCCGGAGCCGCTGCGCG  GACCCAGGACCACCCCCACCTTCTC
```

- ACTR3B–POT1, +7:152087944..-7:124342936 (distance: 27745008 nt)
  Donor exon: NM_020445.exon1/12, NM_001040135.exon1/10
  Acceptor exon: NR_003102.exon3/20, NR_003103.exon3/18, NM_001042594.exon3/18, NR_003104.exon3/20, NM_015450.exon3/19

```
 N1  GTGGACTGTGGCACCGG  GGAAAACTTTCACTTATCTCTTCTTTAACTTATCGTGGAAATAAAACAGCTGTTTTGC
```

- MYH7–KTN1, -14:22958226..+14:55208037 (distance: 32249811 nt)
  Donor exon: NM_000257.exon29/40
  Acceptor exon: NM_182926.exon37/46, NM_004986.exon35/42, NM_001079521.exon36/44, NM_001079522.exon36/43

```
 T3  GGAGGAGGAGGTTAAG  GTTCTAGAGCACAAGTT
```

- NBR1–ACTG1, +17:38704134..-17:77093247 (distance: 38389113 nt)
  Donor exon: NM_031862.exon15/21, NM_005899.exon15/21, NM_031858.exon15/21
  Acceptor exon: NM_001614.exon4/6

```
 T3  ATTTAAAGCACTTCCTG  ATTATGTTTGAGACCT
```

- SERBP1–SPEN, -1:67664406..+1:16127173 (distance: 51537233 nt)
  Donor exon: NM_001018067.exon2/8, NM_001018068.exon2/8, NM_001018069.exon2/8, NM_015640.exon2/8
  Acceptor exon: NM_015001.exon11/15

```
UHR  GTGAAGGAGGCGAATTTTCAGTTGATAG  AGAGGAACGAAGGGCATCCTAC
```

- TBC1D15–UBC, +12:70519858..-12:123964273 (distance: 53444415 nt)
  Donor exon: NM_022771.exon1/18, NM_001146213.exon1/17, NM_001146214.exon1/18, NR_027449.exon1/16
  Acceptor exon: NM_021009.exon2/2

  UHR GGCGGCGGCGGGTGTTGTGAGCGGGAAG ACAATGCAGATCTTCGTGAAGA

- MTAP–RASEF, +9:21849424..-9:84830656 (distance: 62981232 nt)
  Donor exon: NM_002451.exon7/8
  Acceptor exon: NM_152573.exon2/17

  N1 TGGTCAGAAACCCTCCATAACCTGAAG AGAAGAGCAAGTTAGTACCTTGTACCAAAACATCAACCTTGTGGAGCC

- SPATS2–RILPL1, +12:48051340..-12:122536305 (distance: 74484965 nt)
  Donor exon: NM_023071.exon2/14
  Acceptor exon: NM_178314.exon5/7

  N1 AATGTGACTACCCTGGTTACCTCATAT ACGGAGCCGGTGGGAGAGGAGAG

- CDON–DGKZ, -11:125369976..+11:46354650 (distance: 79015326 nt)
  Donor exon: NM_016952.exon13/20
  Acceptor exon: NM_201533.exon24/31, NM_003646.exon24/31, NM_201532.exon24/31, NM_001105540.exon25/32

  UHR GCGATACTCAGATCATGCTAAAGTGGACG CCACCACTGCCAGCCGCTTCT

- REV1–CPSF3, -2:99445390..+2:9501095 (distance: 89944295 nt)
  Donor exon: NM_016316.exon3/23, NM_001037872.exon3/23
  Acceptor exon: NM_016207.exon10/18

  N1 GGAGTTGCCATCTATGTTAATGGATACACAG CACATCATGTCTGAACCTG
  N1 GGAGTTGCCATCTATGTTAATGGATACACAG CACATCATGTCTGAACCTG

- ACACB–CHD4, +12:108168636..-12:6552696 (distance: 101615940 nt)
  Donor exon: NM_001093.exon38/52
  Acceptor exon: NM_001273.exon38/40

  N1 CCCAGAAGACCCCCACAAA CTCTTAGAACAAGCTCTGGTGATTGAGGAACAGCTGCGCCGGGCTGCTTACTTGAA

- C9orf86–VCP, +9:138846643..-9:35047219 (distance: 103799424 nt)
  Donor exon: NM_024718.exon7/15, NM_017995.exon7/10
  Acceptor exon: NM_007126.exon17/17

  UHR GTTCTTCAATATCCCATTTTTGCAGCTTCAG ATTCCCTTCAGGGAACCAG

- UBR4–CACNA1E, -1:19382892..+1:179993706 (distance: 160610814 nt)
  Donor exon: NM_020765.exon17/106
  Acceptor exon: NM_000721.exon31/47

  HBR CTGACCTTCAGTCACCTAACCTGCAG AGGGCGTGCATCGACTTCGCCATC

- ABCA2–FASTK, -9:139042348..-7:150407041 (distance: 0 nt)
  Donor exon: NM_001606.exon1/49
  Acceptor exon: NM_033015.exon2/9, NM_006712.exon3/10

  HBR AGAACGTGACGCTCAAACGCCGGAGCCCG GCTTTCCATCTGATGGTCCCC

- ABCC1–RPS25, +16:16104075..-11:118391859 (distance: 0 nt)
  Donor exon: NM_004996.exon20/31, NM_019900.exon20/30, NM_019862.exon19/30, NM_019899.exon18/29, NM_019898.exon19/30
  Acceptor exon: NM_001028.exon4/5

  UHR TGACGGACAGTGCAGGGAAGCAACTGCAGAG GACTTATCAAACTGGTTTC

- ABCD3–CDC5L, +1:94656732..+6:44521370 (distance: 0 nt)
  Donor exon: NM_002858.exon1/23, NM_001122674.exon1/9
  Acceptor exon: NM_001253.exon15/16

  N1 CGTTCCTGCTGCTCTGCCTGCTCCACAAGCGGCGCCGCGCCCTCGGCCTGCACGG ATAAACAGGGGTCACATGAC

- ABI2–POGZ, +2:203901599..-1:149664161 (distance: 0 nt)
  Donor exon: NM_005759.exon1/10
  Acceptor exon: NM_207171.exon7/18, NM_015100.exon8/19

  UHR GCGAGAACAACTACATACAG TGACCTCTTCCATCCCAGTATTTGACCTCC

- ABR–EIF5B, -17:900040..+2:99343131 (distance: 0 nt)
  Donor exon: NM_001092.exon15/22, NM_021962.exon16/23, NM_001159746.exon16/23
  Acceptor exon: NM_015904.exon2/24

  N2 GCAAAGGACAGATCCAG CACCAAGGATGACATTGATCTTGATGCCTTGGC

- ACP1–NDUFA5, +2:255007..-7:122977876 (distance: 0 nt)
  Donor exon: NM_001040649.exon1/3, NM_004300.exon1/6, NM_007099.exon1/6, NR_024080.exon1/7
  Acceptor exon: NM_005000.exon3/5

  UHR GTGCTGTTTGTGTGTCTGG AGGCTAAGAATATTGTACACAAAGATTCTTG

- ACTR6–BAIAP2L1, +12:99125695..-7:97787546 (distance: 0 nt)
  Donor exon: NM_022496.exon4/11
  Acceptor exon: NM_018842.exon4/14

  N1 AAGAATACCAGTTTCAAGCAGTATTAAGAGTAAATG GACATGTCCTCATAGAGATTTCAAGTACCCACAAGAAAC

- ADD2–RPS6, -2:70755318..-9:19368512 (distance: 0 nt)
  Donor exon: NM_001617.exon14/16, NM_017483.exon10/12, NM_017488.exon14/17
  Acceptor exon: NM_001010.exon4/6

  UHR AGAGGTGGAGAGGAAGAAACTAGAACTTGATG GAGAGAAGGATATTCCTG

- AGRN–RPS9, +1:974302..+19:59403078 (distance: 0 nt)
  Donor exon: NM_198576.exon24/36
  Acceptor exon: NM_001013.exon5/5

  UHR CGCTGCTAGATGGCCGCGTGCAGCTCAG GGTCCGCAAGCAGGTGGTGAAC

- AKT1–SAFB2, -14:104314041..-19:5538410 (distance: 0 nt)
  Donor exon: NM_005163.exon4/14, NM_001014431.exon4/14, NM_001014432.exon5/15
  Acceptor exon: NM_014649.exon21/21

  HBR TGGAGACTCCTGAGGAGCG GCGAGGCGGCTTTGCACAAGGTGGACATTCC

- AMD1–SRCAP, +6:111303111..+16:30653318 (distance: 0 nt)
  Donor exon: NM_001634.exon1/9, NM_001033059.exon1/6
  Acceptor exon: NM_006662.exon31/34

  UHR GATCTGGGGATCTTCGCACTATCCCAAG CAGACCATCCGAGAGCTGTTTG

- AMMECR1L–TRPT1, -2:128358247..-11:63749917 (distance: 0 nt)
  Donor exon: NM_031445.exon2/8
  Acceptor exon: NM_001160389.exon2/8, NM_001033678.exon2/8, NM_001160390.exon2/8, NM_001160392.exon2/7

  UHR CTGTTCCTCTGGCAATACAAACTTTCTGCTTTCA GTCTTAACCATGAACT

- ANK2–PDHA1, +4:114514054..+X:19282530 (distance: 0 nt)
  Donor exon: NM_001148.exon45/46, NM_020977.exon44/45, NM_001127493.exon46/47
  Acceptor exon: NM_000284.exon6/11

  HBR GTGACACCGAGCAGTCAGAG GTGCCCCTGGGCGCTGGGATTGCTCTAGCC

- ANKHD1—ANKHD1–EIF4EBP3-SCNN1D, +5:139842436..+1:1216497 (distance: 0 nt)
  Donor exon: NM_017747.exon11/34, NM_020690.exon11/36
  Acceptor exon: NM_001130413.exon15/15, NM_002978.exon14/14

  UHR GCACTGTGCAGTTTCTTATTAGCAAAG GTGCCGCAGCTGCTCTCGGCCAT

- ANXA4–CALD1, +2:69862210..+7:134300597 (distance: 0 nt)
  Donor exon: NM_001153.exon2/13
  Acceptor exon: NM_004342.exon13/14, NM_033138.exon14/15, NM_033139.exon12/13, NM_033140.exon11/12, NM_033157.exon14/1

  N1 CTGATCTTGACCTAGAGTCATGGCCATG GACTTGAGACCAGGAGACGTAT

- APEX1–RPL27A, +14:19993702..+11:8662129 (distance: 0 nt)
  Donor exon: NM_001641.exon2/5, NM_080648.exon2/5, NM_080649.exon2/5
  Acceptor exon: NM_000990.exon3/5

  UHR GCGGAAGACGGGGATGAGCTCAGGACAG GCAAGCACCGGAAGCACCCCGG

- ARPC5L–HNRNPA2B1, +9:126675864..-7:26198722 (distance: 0 nt)
  Donor exon: NM_030978.exon2/4
  Acceptor exon: NM_002137.exon10/11, NM_031243.exon11/12

```
UHR  CAACACCAAGAATCAAGCTGTGAAG  GAAACTATGGTCCAGGAGGCAGTGG
```

- ASPH–ENO1, -8:62756081..-1:8848128 (distance: 0 nt)
  Donor exon: NM_032466.exon3/14, NM_032468.exon4/15, NM_020164.exon4/6, NM_001164750.exon3/25, NM_001164751.exon4/15, NM_001164752.exon4/14, NM_032467.exon3/5, NM_001164753.exon4/14, NM_001164754.exon3/13, NM_001164755.exon3/13, NM_001164756.exon4/5, NM_004318.exon3/25
  Acceptor exon: NM_001428.exon8/12

```
UHR  TGATGCCAAAGTTTTATTAG  GCCTGGAGCTGCTGAAGACTGCTATTGGGA
```

- ATP5I–MTMR4, -4:657701..-17:53928690 (distance: 0 nt)
  Donor exon: NM_007100.exon2/4
  Acceptor exon: NM_004687.exon16/19

```
 T3  CGGAGCCACGCGCTACA  ATTACCTAAAACCAGA
```

- B2M–ILF3, +15:42795846..+19:10655042 (distance: 0 nt)
  Donor exon: NM_004048.exon3/4
  Acceptor exon: NM_012218.exon15/20, NM_004516.exon15/18, NM_153464.exon15/18, NM_017620.exon15/20, NM_001137673.exon1

```
UHR  CGAGACATGTAAGCAGCATCATGGAG  GTCGAAGTGGATGGACAGAAGTTC
```

- B3GALNT2–TCF12, -1:233724614..+15:55245892 (distance: 0 nt)
  Donor exon: NM_152490.exon2/12
  Acceptor exon: NM_003205.exon6/20, NM_207036.exon6/21, NM_207037.exon6/21, NM_207038.exon6/20

```
 N1  GAAGCACCTGGATGAGACATTTGCTACAGCATCCCACATTAAGTCAACG  GAAAAACATCAGAGAGAGGCTCATTT
```

- B3GALNT2–WDR44, -1:233724614..+X:117450772 (distance: 0 nt)
  Donor exon: NM_152490.exon2/12
  Acceptor exon: NM_019045.exon13/20

```
 N1  TTTGCTACAGCATCCCACATTAAGTCAACG  GTATGCAGTGGAACTGATGAAGACCCTGATGATAAAAACGCACCC
```

- B3GNTL1–SLC9A8, -17:78507860..+20:47927925 (distance: 0 nt)
  Donor exon: NM_001009905.exon11/13
  Acceptor exon: NM_015266.exon12/16

```
UHR  CGGCCACCCTTCGTCATCTGCGTGAAGCTG  AAACATGTGTGTTTGCATTT
```

- BAZ2B–IVNS1ABP, -2:159963586..-1:183541398 (distance: 0 nt)
  Donor exon: NM_013450.exon18/37
  Acceptor exon: NM_006469.exon8/15

```
 N2  GCCAAATTATTGGAGGCCGAGAAACGAATAAAG  GTTCAAACCTTGTACTA
```

- BCAP29–DLGAP4, +7:107041113..+20:34558522 (distance: 0 nt)
  Donor exon: NM_018844.exon7/8, NM_001008405.exon6/8, NR_027830.exon6/7
  Acceptor exon: NM_183006.exon2/7, NM_014902.exon7/12, NM_001042486.exon2/7

```
UHR  AAAGAACACTCTGAACTTCAG  GTTCATCATGCCTAGTGGCGTATAAGAAG
```

- BCAS3–IVNS1ABP, +17:56141468..-1:183541398 (distance: 0 nt)
  Donor exon: NM_017679.exon5/24, NM_001099432.exon5/25
  Acceptor exon: NM_006469.exon8/15

```
UHR  GGAATGCAGGTCTGGAGCATCCCT  GTTCAAACCTTGTACTACTCAGCTGA
```

- BCAS4–BCAS3, +20:48845117..+17:56800470 (distance: 0 nt)
  Donor exon: NM_017843.exon1/6, NM_198799.exon1/5, NM_001010974.exon1/4
  Acceptor exon: NM_017679.exon23/24, NM_001099432.exon24/25

```
UHR  CCTGACCCCCGAtCCTGGGGCCGAG  GTACCTTTGACAGGAGCGTGACCCT
UHR    TGACCCCCGAtCCTGGGGCCGAG  GTACCTTTGACAGGAGCGTGACCCTGC
UHR         CCCGAtCCTGGGGCCGAG  GTACCTTTGACAGGAGCGTGACCCTGCTGGAG
UHR          CCGAtCCTGGGGCCGAG  GTACCTTTGACAGGAGCGTGACCCTGCTGGAGG
```

- BCL7A–MKRN1, +12:120944472..-7:139806205 (distance: 0 nt)
  Donor exon: NM_020993.exon1/6, NM_001024808.exon1/6
  Acceptor exon: NM_013446.exon3/8, NM_001145125.exon3/5

```
 N2  CGATCGAGAAAGTGCGCAAATG  ATATGAACATAGCAAACCATTGAAACAG
```

- BCR–ABL1, +22:21962600..+9:132719272 (distance: 0 nt)
Donor exon: NM_004327.exon14/23, NM_021574.exon14/22
Acceptor exon: NM_007313.exon2/11, NM_005157.exon2/11

```
UHR  GTCCACTCAGCCACTGGATTTAAGCAGAGTTCAA  AAGCCCTTCAGCGGCC
UHR                   ATTTAAGCAGAGTTCAA  AAGCCCTTCAGCGGCCAGTAGCATCTGACTTTG
UHR                    TTTAAGCAGAGTTCAA  AAGCCCTTCAGCGGCCAGTAGCATCTGACTTTGA
```

- BRCA1–SPARC, -17:38484877..-5:151023989 (distance: 0 nt)
Donor exon: NM_007300.exon13/24
Acceptor exon: NM_003118.exon9/10

```
UHR  TAAAAGGCCTAGAGAACATATATCgG  GTACCTCTCCCACACCGAGCTGGC
```

- BRWD1–ZMIZ1, -21:39606637..+10:80740687 (distance: 0 nt)
Donor exon: NM_001007246.exon4/5, NM_018963.exon4/42, NM_033656.exon4/41
Acceptor exon: NM_020338.exon24/25

```
HBR  ACAACAGGAGCTACGAGGAGTTG  ATGCCACACGCTGGCAGCTCTGACCAG
```

- C10orf116–HLA-C, +10:88719999..-6:31344929 (distance: 0 nt)
Donor exon: NM_006829.exon2/3
Acceptor exon: NM_002117.exon8/8

```
 T2  GAGGCGGGGCAGAAAG  CCTGAGACAGCTGCCTG
```

- C13orf15–BAT3, +13:40929892..-6:31719950 (distance: 0 nt)
Donor exon: NM_014059.exon1/5
Acceptor exon: NM_004639.exon12/25, NM_080702.exon12/25, NM_080703.exon12/25, NM_001098534.exon12/25

```
 T2  GCCGCCGCGGCCGCAG  GACAGCAGGTGCCAGGC
```

- C4orf14–CRTC2, -4:57525329..-1:152188484 (distance: 0 nt)
Donor exon: NM_032313.exon6/7
Acceptor exon: NM_181715.exon12/14

```
HBR  CCGACATCAAGTTTTCCTCTGCAG  GGCGTCCCCCTGGATACCAGTAAACT
```

- C9orf16–CCNH, +9:129962586..-5:86726057 (distance: 0 nt)
Donor exon: NM_024112.exon1/2
Acceptor exon: NM_001239.exon9/9

```
UHR  CGAGGAGGACGGCTTCGGGGAAGCAG  GAAGAATGGACTGATGACGACCTG
```

- C9orf3–SHARPIN, +9:96884822..-8:145230116 (distance: 0 nt)
Donor exon: NM_032823.exon14/15
Acceptor exon: NM_030974.exon2/9

```
 N2  CCGAAATGTTATTTTAACGAG  GTTAATTTGGAGTGGCCCCTGGAGTCAGT
```

- CABIN1–ACIN1, +22:22860382..-14:22608666 (distance: 0 nt)
Donor exon: NM_012295.exon29/37
Acceptor exon: NM_001164814.exon9/19, NM_001164815.exon8/18, NM_001164816.exon2/12, NM_014977.exon9/19, NM_001164817.exon3/13

```
UHR  GAGGAACAAGACCAATTTCTTCAAC  TGAGGAGAAGGAGGAAGTGACCATG
```

- CACNA1B—LOC100294433–CYTH1, +9:139897156..-17:74217409 (distance: 0 nt)
Donor exon: NM_000718.exon3/47, XR_079263.exon3/47
Acceptor exon: NM_017456.exon2/13, NM_004762.exon2/14

```
HBR  TTCGTGGTCGTCCTCACAGG  TTCCCAGTGACCTGACAGCAGAGGAGCGTC
```

- CADPS–STX6, -3:62398819..-1:179229184 (distance: 0 nt)
Donor exon: NM_183393.exon25/27, NM_183394.exon26/28, NM_003716.exon28/30
Acceptor exon: NM_005819.exon4/8

```
HBR  TAGAAAGGTTATTTGAT  GACATGAAAGATCAGATGTCAACTTCATCTGTG
```

- CAMKK2–CCDC123, -12:120166759..-19:38062124 (distance: 0 nt)
Donor exon: NM_172216.exon15/16, NM_006549.exon16/17, NM_172214.exon16/17, NM_172215.exon15/16
Acceptor exon: NM_032816.exon19/19

```
HBR  AGTCCCTGTCTGAGCTCAAG  AGAAATGGAAGGTGAACTTGAAGTTATTTG
```

- CAMTA1–SPPL3, +1:6807857..-12:119713743 (distance: 0 nt)
  Donor exon: NM_015215.exon3/23
  Acceptor exon: NM_139015.exon3/11

```
N1  AAATGTTCAAGTTTACCAAAAGAGAGGCACCGCTGGAACACTAATGAG  GTCCCTTAATATGGACTTTGAAAATCA
N1                            GGCACCGCTGGAACACTAATGAG  GTCCCTTAATATGGACTTTGAAAATCA
```

- CCBL2—RBMXL1–CHERP, -1:89230856..-19:16493465 (distance: 0 nt)
  Donor exon: NM_001008662.exon1/13, NM_001008661.exon1/14, NM_019610.exon1/2, NM_001162536.exon1/3
  Acceptor exon: NM_006387.exon12/17

```
UHR  GCCTAGGGAGGCGAGGTTCCCGCACCGGATAG  CTGGAAGATCACGAGTAC
```

- CCDC82–NCOA7, -11:95762656..+6:126278172 (distance: 0 nt)
  Donor exon: NM_024725.exon1/10
  Acceptor exon: NM_001122842.exon11/16, NM_181782.exon11/16

```
HBR  GCAGAGGTGGAGGCGCTTTGAAAG  GGTGGATCATTTGTACACATTCTTTG
```

- CD163–HNRNPUL1, -12:7526829..+19:46503421 (distance: 0 nt)
  Donor exon: NM_004244.exon13/17, NM_203416.exon13/17
  Acceptor exon: NM_007040.exon14/15, NM_144732.exon14/15

```
 T3  ACAAAAAGCCACAACAG  CCGAGTTACAGCCAGC
```

- CD46–GNAS, +1:205992277..+20:56904062 (distance: 0 nt)
  Donor exon: NM_172359.exon1/13, NM_172360.exon1/12, NM_172361.exon1/10, NM_002389.exon1/14, NM_172350.exon1/10, NM_172351.exon1/13, NM_172352.exon1/12, NM_172353.exon1/11, NM_172354.exon1/13, NM_172355.exon1/12, NM_172356.exon1/ NM_172357.exon1/11, NM_172358.exon1/12, NM_153826.exon1/12
  Acceptor exon: NM_000516.exon2/13, NM_080426.exon2/13, NM_001077488.exon2/14, NM_080425.exon2/13, NM_001077489.exon2/ NM_016592.exon2/14, NM_001077490.exon2/13, NR_003259.exon2/13

```
 N1  TTCTGGCGGCCATGGTGTTGCTGCTGTACTCCTTCTCCG  GTGCTGGAGAATCTGGTAAAAGCACCATTGTGAAGC
```

- CHD2–FGFR1OP2, +15:91245533..+12:27007542 (distance: 0 nt)
  Donor exon: NM_001042572.exon2/13, NM_001271.exon2/39
  Acceptor exon: NM_015633.exon6/7

```
HBR  GGACAGTTCGCTACACAGCAATGCATCGAG  GAACTGCAAGCACATGTTGA
```

- CHD2–SFRS11, +15:91245533..+1:70459883 (distance: 0 nt)
  Donor exon: NM_001042572.exon2/13, NM_001271.exon2/39
  Acceptor exon: NM_004768.exon2/13

```
 T2  ACAGCAATGCATCGAG  CAGGAGCGAGAACCCGA
```

- CIRH1A–FAIM2, +16:67742308..-12:48578140 (distance: 0 nt)
  Donor exon: NM_032830.exon8/17
  Acceptor exon: NM_012306.exon3/12

```
HBR  ATGCCGCTCTCCGAAAAATCACCTTTCCCCAC  GCAGCAGCTCCAGCTATG
```

- CLDN12–LOC731751—PRKDC, +7:89870799..-8:48934100 (distance: 0 nt)
  Donor exon: NM_012129.exon1/3
  Acceptor exon: XM_001129414.exon47/85, NM_001081640.exon47/85, NM_006904.exon47/86

```
UHR  CCCTGCGTGTGAGAAGCAG  GAGCAGCGGGACCCCACGGTGCATGATGATG
```

- CLK3–BIN1, +15:72699619..-2:127550752 (distance: 0 nt)
  Donor exon: NM_003992.exon3/13, NM_001130028.exon3/13
  Acceptor exon: NM_004305.exon2/15, NM_139343.exon2/19, NM_139344.exon2/18, NM_139345.exon2/16, NM_139346.exon2/16, NM_139347.exon2/16, NM_139348.exon2/15, NM_139349.exon2/15, NM_139350.exon2/14, NM_139351.exon2/13

```
HBR  CTTGTAGCAGCGCCTCCTCG  GTTCTCCAGAAGCTGGGGAAGGCAGATGAG
```

- CLU–PTGES2, -8:27511894..-9:129925234 (distance: 0 nt)
  Donor exon: NM_001171138.exon8/9, NM_001831.exon8/9, NM_203339.exon8/9
  Acceptor exon: NM_025072.exon5/7, NR_027811.exon6/8, NR_027812.exon5/7

```
HBR  AATACCGCAAAAAGCACCG  GGAGGAGATGAAGTGGCGGCAGTGGGCGGAC
```

- CMPK1–STRAP, +1:47613552..+12:15947118 (distance: 0 nt)
  Donor exon: NM_016308.exon5/6, NM_001136140.exon4/5
  Acceptor exon: NM_007178.exon10/10

UHR CTAAATCTGTTGATGAA AAGAAATTGCTTCAGAGAATTCAGATTGCATCT

- CNOT1–C10orf4, -16:57137726..-10:95420595 (distance: 0 nt)
  Donor exon: NM_016284.exon29/49, NM_206999.exon29/31
  Acceptor exon: NM_145246.exon13/14

  N2 GAAGAACTCCCTCCCATCACAACCACAA GAAACTCTGATGAGGAAGAAAG

- CNTN1–FUS, +12:39639303..+16:31107147 (distance: 0 nt)
  Donor exon: NM_175038.exon14/23, NM_001843.exon15/24
  Acceptor exon: NM_004960.exon8/15, NR_028388.exon7/14, NM_001170937.exon8/15, NM_001170634.exon8/15

  HBR AATTCTTCAGCTTCAGCTGACCTTGTAGTGAGAG GCCCTCGGGACCAAGG

- COG4–EEA1, -16:69099810..-12:91809628 (distance: 0 nt)
  Donor exon: NM_015386.exon8/19
  Acceptor exon: NM_003566.exon2/29

  HBR CAGAAAAAAATCGAACCAAG ACTCCTGGGAGAGTTGGCTCTCAAGGTTCTG

- CPSF6–KDM4C, +12:67942609..+9:7005853 (distance: 0 nt)
  Donor exon: NM_007007.exon9/10
  Acceptor exon: NM_015061.exon15/22, NM_001146694.exon15/21, NM_001146695.exon15/18, NM_001146696.exon15/18

  N1 CGAATATCGTCATCGTTAGAAG GTTGTTATGGTATTCCTTCTCATGAGATCTGTGATGGATGGCTGTGTGCCCGG

- CRY1–SAFB2, -12:105910695..-19:5543898 (distance: 0 nt)
  Donor exon: NM_004075.exon12/13
  Acceptor exon: NM_014649.exon16/21

  HBR CTAAAGTCCAGAGACAGAGCACTAATTAG ACGAGATGATGCCTATTGGCC

- CTDSPL–YLPM1, +3:37878773..+14:74365669 (distance: 0 nt)
  Donor exon: NM_005808.exon1/7, NM_001008392.exon1/8
  Acceptor exon: NM_019589.exon19/21

  N2 GCCGGGCGCGGGCGAGAAAG ACAAGTTGGATGGCTTGAGGACTGGTACTA

- CUX1–C6orf106, +7:101246093..-6:34730534 (distance: 0 nt)
  Donor exon: NM_001913.exon1/23, NM_181500.exon1/23
  Acceptor exon: NM_024294.exon2/5, NM_022758.exon2/4

  N3 ATTTACAGCAGCTGCAG GAACCTACAAGCAGCA

- DDX5–GALNS, -17:59932656..-16:87420747 (distance: 0 nt)
  Donor exon: NM_004396.exon1/13
  Acceptor exon: NM_000512.exon10/14

  UHR CCGAGACCGCGGCCGGGACCGAGG GTGAGCCACCAGCTGGGCAGCATCAT

- DFFA–SULF2, -1:10451821..-20:45721872 (distance: 0 nt)
  Donor exon: NM_004401.exon2/6, NM_213566.exon2/5
  Acceptor exon: NM_198596.exon19/21

  UHR AGTAATGAGAAATGGGCATACAACAATTCAG ATGGAGGAAGCTATGAGCA

- DGKD–CYB5A, +2:233928059..-18:70073990 (distance: 0 nt)
  Donor exon: NM_152879.exon1/30
  Acceptor exon: NM_148923.exon4/5, NM_001914.exon5/6

  N1 GTCAGATCCGACAGAAG GAAACTCTTATCACTACTATTGATTCTAGTTCC

- DNM1–MTSS1L, +9:130024960..-16:69256500 (distance: 0 nt)
  Donor exon: NM_004408.exon9/22, NM_001005336.exon9/23
  Acceptor exon: NM_138383.exon13/15

  HBR AGAATATCCATGGCATTAG GACTGGTCCAAGGTCGGCTCCCATGAGCAGC

- DYNC1H1–EIF4B, +14:101540773..+12:51708066 (distance: 0 nt)
  Donor exon: NM_001376.exon24/78
  Acceptor exon: NM_001417.exon8/15

  UHR GGGTATTTCATCTCGGGAAGGAGAGGAG GCTATGATTCCCGGATAGGCAG
  UHR GGGTATTTCATCTCGGGAAGGAGAGGAG GCTATGATTCCCGGATAGGCAG

- EGFL7–TCF3, +9:138679058..-19:1601286 (distance: 0 nt)
  Donor exon: NM_016215.exon2/10, NM_201446.exon2/10
  Acceptor exon: NM_003200.exon2/19

  `UHR  CTGATTCTCCTCCGCCAG  GGTTTCCAGGCCTGAGGTGCCCGCCCTGGCCC`

- ELOVL5–FAM82B, -6:53264533..-8:87556298 (distance: 0 nt)
  Donor exon: NM_021814.exon3/8
  Acceptor exon: NM_016033.exon9/10

  `N1  GGATTTTAGTGGTGTATAACCTTGGACTCACACTGCTGTCTCTGTATATGTTCTGTGAG  TGGATCCAAACTTCTA`

- EPB41–USP3, +1:29264257..+15:61611899 (distance: 0 nt)
  Donor exon: NM_001166005.exon16/21, NM_203343.exon12/17, NM_203342.exon16/21, NM_001166007.exon13/18, NM_004437.exon15/19
  Acceptor exon: NM_006537.exon2/15

  `HBR  GGGCAAATCCCCACAGGAGAAGGA  TGTGCCGGTCCAACAAAAGCCCTTGG`

- FGFR4–LIMA1, +5:176449300..-12:48880934 (distance: 0 nt)
  Donor exon: NM_002011.exon2/18, NM_213647.exon2/18, NM_022963.exon1/16
  Acceptor exon: NM_016357.exon7/11, NM_001113547.exon4/8, NM_001113546.exon7/11

  `N1  CCCTGGAGGCCTCTGAGGAAGTGGAGCTTG  AATGAGCTGAAAGCCAGTGG`

- FNDC3A–VTI1A, +13:48478426..+10:114276836 (distance: 0 nt)
  Donor exon: NM_001079673.exon2/26
  Acceptor exon: NM_145206.exon4/8

  `UHR  TGCAGATGGAACACAACAG  AAAAGGTCACGGATCGCCTACAGTGACGAAG`

- FOSL1–PTMS, -11:65424288..+12:6749030 (distance: 0 nt)
  Donor exon: NM_005438.exon1/4
  Acceptor exon: NM_002824.exon2/5

  `UHR  CGCAGCGCAGGCAGCCCAGCAG  GACCTGAAGGAGAAGAAGGAGAAGGTGG`

- FTH1–SIN3B, -11:61491360..+19:16843040 (distance: 0 nt)
  Donor exon: NM_002032.exon1/4
  Acceptor exon: NM_015260.exon15/20

  `UHR  CTCCTACGTTTACCTGTCCATG  GTGAAGTGGAGCTGGAGGAGTACTACCC`

- GABBR2–CHN1, -9:100256084..-2:175397492 (distance: 0 nt)
  Donor exon: NM_005458.exon7/19
  Acceptor exon: NM_001025201.exon8/13, NM_001822.exon8/13

  `HBR  ACGAGACCAACTTCTTCGGGGTCACG  GTGCATACATTCAGAGGGCCACAC`

- GALNTL1–SGTA, +14:68884472..-19:2710292 (distance: 0 nt)
  Donor exon: NM_020692.exon14/16, NM_001168368.exon14/15
  Acceptor exon: NM_003021.exon9/12

  `HBR  TAGAGAAGGCAAGCAG  GCTTCGAACCTAATGAACAATCCCCAGATTCAGC`

- GLYCTK–EXD3, +3:52296932..-9:139323973 (distance: 0 nt)
  Donor exon: NM_145262.exon1/5, NR_026700.exon1/6, NR_026701.exon1/6, NR_026702.exon1/5, NM_001144951.exon1/4, NR_026699.exon1/5
  Acceptor exon: NM_017820.exon20/22

  `HBR  CGCGGCCGGAGCTGTGGGCTG  GCCTGTAACTGTGACCAGTACCTAAAGGT`

- GNA12–RNPC3, -7:2801088..+1:103899179 (distance: 0 nt)
  Donor exon: NM_007353.exon2/4
  Acceptor exon: NM_017619.exon3/3

  `UHR  AGGCTTTCAGCCGGAGAAGCGAGTTTCAGCTG  GTTTTTTTGAATCCCGTG`

- GOLGA8B–NAP1L1, -15:32613547..-12:74730700 (distance: 0 nt)
  Donor exon: NM_001023567.exon2/16
  Acceptor exon: NM_004537.exon12/15, NM_139207.exon12/16

  `UHR  GTTGCCACTCATCTGAaGCT  GATGATGATGCTGAAGCTATCCTTGCTGCA`

- HN1L–RBX1, +16:1668358..+22:39689997 (distance: 0 nt)
  Donor exon: NM_144570.exon1/5
  Acceptor exon: NM_014248.exon3/5

  `HBR GCGAGGGCGGCCGCGCCGGCTCCAG GCATAGAATGTCAAGCTAACCAGGC`

- HNRNPM–PABPN1, +19:8436246..+14:22863187 (distance: 0 nt)
  Donor exon: NM_031203.exon6/17
  Acceptor exon: NM_004643.exon6/7

  `UHR TGCCAGGAGAGCAATGCAAAAG GTGATCCCAAAACGAACCAACAGACCAG`

- HOMER2–EXOC6B, -15:81329973..-2:72580609 (distance: 0 nt)
  Donor exon: NM_199331.exon4/9, NM_199332.exon4/9, NM_004839.exon4/9, NM_199330.exon4/9
  Acceptor exon: NM_015189.exon12/22

  `N1 AGACCTCAAGTAATCATTCCCAA TCTTACTGTTCTGATCCAAACCTTGTGTTAGATTTGAAGAACCTCATTGTGC`

- HS6ST2–YAP1, -X:131918518..+11:101538397 (distance: 0 nt)
  Donor exon: NM_147175.exon3/4
  Acceptor exon: NM_001130145.exon3/9, NM_006106.exon3/7

  `UHR ACGCCAGGCTGAGACCGTCCAG TCACATCGATCAGACAACAACATGGCAG`

- HSP90AA1–DCTN1, -14:101619636..-2:74449849 (distance: 0 nt)
  Donor exon: NM_001017963.exon9/12, NM_005348.exon8/11
  Acceptor exon: NM_004082.exon15/32, NM_023019.exon10/27, NM_001135040.exon12/28, NM_001135041.exon10/26

  `HBR GGAGAACCAGAAACATATCTATTATATCACAG GATGTGAATCGGGAACTG`

- ING3–CHMP1A, +7:120382914..-16:88241240 (distance: 0 nt)
  Donor exon: NM_019071.exon4/12, NM_198267.exon4/5
  Acceptor exon: NM_002768.exon5/7, NM_001083314.exon4/6

  `HBR AACCAGATATATGACTTG GTGACCAAGAATATGGCCCAGGTGACCAAAGC`

- INTS1–RPS6KB2, -7:1492787..+11:66955415 (distance: 0 nt)
  Donor exon: NM_001080453.exon22/48
  Acceptor exon: NM_003952.exon5/15

  `UHR CGCGTGCTGGCCATGAAG GCCAAAATTGTGCGCAATGCCAAGGACACAGC`

- INTS3–XPC, +1:152001947..-3:14175396 (distance: 0 nt)
  Donor exon: NM_023015.exon15/30
  Acceptor exon: NM_004628.exon9/16, NM_001145769.exon9/16, NR_027299.exon8/15

  `T1 GATCTCAACAGCAAAG GGAAAGAAACCTTCCAA`

- KDM6A–OSBPL1A, +X:44618177..-18:20073344 (distance: 0 nt)
  Donor exon: NM_021140.exon2/29
  Acceptor exon: NM_080597.exon16/28, NM_018030.exon2/14

  `UHR GGCGCCAGGACGAAGGCCCTACTGGGCAAG GTGAGGAATTTTAAATTGGA`

- KDM6B–SKI, +17:7684067..+1:2224277 (distance: 0 nt)
  Donor exon: NM_001080424.exon1/22
  Acceptor exon: NM_003036.exon2/7

  `HBR CAATGAGACAGGGCACACCAAACTCCATCTG GTCTCCTCTGAGCCTCCGG`

- KDR–RPL29, -4:55671327..-3:52004525 (distance: 0 nt)
  Donor exon: NM_002253.exon9/30
  Acceptor exon: NM_000992.exon2/4

  `T2 TCTCTGGTTGTGTATG GTGCAGACATGGCCAAG`

- KHDRBS3–CABP1, +8:136539379..+12:119582064 (distance: 0 nt)
  Donor exon: NM_006558.exon1/9
  Acceptor exon: NM_031205.exon3/7, NM_004276.exon2/6, NM_001033677.exon2/6

  `HBR TCCTTCACGCACGCCCTGCGCCTGGTGAACCAAG GATAGATCACTGCGAC`

- KIAA1370–PHF20, -15:50673067..+20:33998821 (distance: 0 nt)
  Donor exon: NM_019600.exon10/13
  Acceptor exon: NM_016436.exon18/18

HBR AGATGACAATGCTCCCTCTCCTTATATG TGTTGGAGAGCTGGCTGGACTA

- KIAA1429–CCDC47, -8:95610474..-17:59192443 (distance: 0 nt)
  Donor exon: NM_015496.exon7/24, NM_183009.exon7/13
  Acceptor exon: NM_020198.exon5/13

UHR AGAAGGTGAAGAGGATGAAGAAGGcGAAG GGGATGATGGAACTAACAAAG

- KRI1–UTP6, -19:10531035..-17:27226545 (distance: 0 nt)
  Donor exon: NM_023008.exon12/19
  Acceptor exon: NM_018428.exon14/19

UHR CCCAGCACGACCAGCTCATGCAG AGTGTTTCGTTGCTGTGTTATAACTTC

- LASS4–UBXN4, +19:8181746..+2:136243809 (distance: 0 nt)
  Donor exon: NM_024552.exon2/12
  Acceptor exon: NM_014607.exon7/13

N2 GTTTTTCCAGCTCACCCACTGCCAGCAGAG ACTAACAAAAAAACTTGAAG

- LOC100129096—TPM4–IGBP1, +19:16053856..+X:69302408 (distance: 0 nt)
  Donor exon: XR_079235.exon2/8, NM_003290.exon2/8, NM_001145160.exon3/9
  Acceptor exon: NM_001551.exon7/7

UHR AGGAGGCAGAAAAAGCTGCAGATGAGAGTGAGAG AGGAATTCAGAAAAGC

- LOC375190–MPHOSPH9, +2:24211561..-12:122207408 (distance: 0 nt)
  Donor exon: NM_001145710.exon3/11
  Acceptor exon: NM_022782.exon20/20

N1 AAAGAAAATTCTGTAATTAAG GAAGCCTTGGAAGATCGTTTGGAAAGGATTAATCGAGAACTGGGTTCAGTTCGC

- LPCAT1–EIF2B4, -5:1534057..-2:27440970 (distance: 0 nt)
  Donor exon: NM_024830.exon7/14
  Acceptor exon: NM_172195.exon12/12, NM_015636.exon13/13, NM_001034116.exon13/13

N1 ACGTGGCAAGGACCTGGAGC ATGACCCTGATGATCTGCAATGTAAGCGGGGAGAACATGTTGCGCTGGCTAACTG

- LRCH1–FAM13B, +13:46025839..-5:137384785 (distance: 0 nt)
  Donor exon: NM_015116.exon1/19, NM_001164211.exon1/20, NM_001164213.exon1/19
  Acceptor exon: NM_016603.exon2/23, NM_001101800.exon2/22, NM_001101801.exon2/22

HBR CGGACACGGTGCAGGCAG ATTAACTGCTGAAGTACTGATCGAGTTCTGCA

- LRRC41–DAZAP1, -1:46541383..+19:1368499 (distance: 0 nt)
  Donor exon: NM_006369.exon1/10
  Acceptor exon: NM_018959.exon2/12, NM_170711.exon2/13

UHR CCATATGGGGGTTCTGGAGAGCGGGGTGTGGG GAAGCTCTTCGTGGGCGG

- MAN2A2–ATP5G3, +15:89254526..-2:175754440 (distance: 0 nt)
  Donor exon: NM_006122.exon9/22
  Acceptor exon: NM_001689.exon2/5

HBR CTTAGACCGAGTCCTGGAAGCCCACCTGCG AGAGGAAGCGGGAGAGGAGC

- MAPK14–LCMT1, +6:36135102..+16:25083420 (distance: 0 nt)
  Donor exon: NM_139012.exon3/12, NM_139014.exon3/11, NM_001315.exon3/12, NM_139013.exon3/10
  Acceptor exon: NM_001032391.exon5/9, NM_016309.exon7/11

UHR TGGAGGAATTCAATGATGT ATTGCCAACACTCCTGATAGCTGAATGTGTG

- MAX–ENOPH1, -14:64630179..+4:83588097 (distance: 0 nt)
  Donor exon: NM_197957.exon3/4, NM_145112.exon2/4, NM_145113.exon3/6, NM_145116.exon3/4, NM_002382.exon3/5, NM_145114.exon3/4
  Acceptor exon: NM_021204.exon2/6

HBR CAGTCCCATCACTCCAAGGAGAGAAG GACATTTTATTTCCTTACATCGAA

- MBTPS1–SERF2, -16:82647111..+15:41873200 (distance: 0 nt)
  Donor exon: NM_003791.exon22/23
  Acceptor exon: NM_001018108.exon3/3

T3 CTGGGACATTCCTGGAG GGACTCGGAGATCATG
T3 CTGGGACATTCCTGGAG GGACTCGGAGATCATG

- ME3–CUX2, -11:85853781..+12:110242199 (distance: 0 nt)
  Donor exon: NM_006680.exon8/15, NM_001014811.exon7/14, NM_001161586.exon8/15
  Acceptor exon: NM_015267.exon17/22

  HBR CAAGTACTGCATGTTCAATGATGACATCCAAG GCGAGCCCAAGACCTCGG

- MED10–KIDINS220, -5:6427437..-2:8863950 (distance: 0 nt)
  Donor exon: NM_032286.exon3/4
  Acceptor exon: NM_020738.exon7/30

  HBR GCAAGATCGACACCATGAAG TATGGAACCACCCCTTTAGTTTGGGCTGCA

- MED12L–AES, +3:152394143..-19:3006724 (distance: 0 nt)
  Donor exon: NM_053002.exon14/43
  Acceptor exon: NM_001130.exon5/7, NM_198969.exon5/7, NM_198970.exon5/7

  N2 CACATTTTCCTATACCTCTG GCTGAGATCGTCAAAAGGCTGAACGGGATT

- MED1–FMNL3, -17:34860817..-12:48338644 (distance: 0 nt)
  Donor exon: NM_004774.exon1/17
  Acceptor exon: NM_175736.exon6/26

  UHR GAAAGCTCAGGGGGAAACCGAGG GTTTGACTTTGAGGGTCTGGAAAGTGG

- MOBKL1B–PPAP2A, -2:74259296..-5:54773493 (distance: 0 nt)
  Donor exon: NM_018221.exon1/6
  Acceptor exon: NM_003711.exon4/6, NM_176895.exon4/6

  N2 CCTCTGAGGACCGAAGATGAGCTTCCTCTT GTTGTCCTTCTATTCAGGCC

- MOG–S100B, +6:29735422..-21:46843844 (distance: 0 nt)
  Donor exon: NM_002433.exon2/8, NM_001008229.exon2/6, NM_206811.exon2/8, NM_206813.exon2/9, NM_206810.exon2/7, NM_206809.exon2/8, NM_206812.exon2/7, NM_001008228.exon2/8, NM_206814.exon2/9
  Acceptor exon: NM_006272.exon3/3

  HBR GGAGGCAGCAATGGAATTGAAAGTAGAAG GAAATCAAAGAGCAGGAGGTT

- MYBBP1A–RPL3, -17:4395070..-22:38039680 (distance: 0 nt)
  Donor exon: NM_014520.exon17/26, NM_001105538.exon17/27
  Acceptor exon: NM_000967.exon8/10, NM_001033853.exon8/10

  UHR TGCAGGCTGGGAAGGCGCTG GGTGGCTTTGTCCACTATGGTGAAGTGACC

- MYCBP2–ZFR, -13:76527694..-5:32424537 (distance: 0 nt)
  Donor exon: NM_015057.exon81/84
  Acceptor exon: NM_016107.exon13/20

  HBR TGCATATTATGTGTGCTACAAATGCAGAAAG CCCTTACGTCGTCCTGACT

- MYH9–SDCCAG3, -22:35020084..-9:138418966 (distance: 0 nt)
  Donor exon: NM_002473.exon28/41
  Acceptor exon: NM_006643.exon7/9, NM_001039707.exon8/10, NM_001039708.exon6/8

  UHR AGAGCTGGCCGACAAGGTCACCAAGCTGCAG AAACGGGCTGTAAAGGCAG

- MYO7A–DDX17, +11:76591117..-22:37212391 (distance: 0 nt)
  Donor exon: NM_000260.exon37/49, NM_001127180.exon37/49
  Acceptor exon: NM_006386.exon13/13, NM_030881.exon2/2, NM_001098504.exon13/13, NM_001098505.exon2/2

  N2 GGAGTTTTCCTATGACTACTTCAG GTGGTCGTTCTCGTTACCGGACCACT

- NAPA–RAG1AP1, -19:52709912..+1:153375399 (distance: 0 nt)
  Donor exon: NM_003827.exon1/11
  Acceptor exon: NM_018845.exon2/6, NM_001122837.exon2/5, NM_001122839.exon2/5

  N1 GGCGGAGCGCAAAGTGAAGAACTCGCAGTCCTTCTTCTCTGGCCTCTTTGG CTCGGACCTCAGGCACATGCGAAT

- NCOR2–C9orf3, -12:123448618..+9:96888785 (distance: 0 nt)
  Donor exon: NM_006312.exon17/48, NM_001077261.exon17/47
  Acceptor exon: NM_032823.exon15/15

  UHR AAGAAATGGAAACAGCCAAGAAAG GAAAGACCACAGCAAGATTCTTTCAT

- NDUFA6–SPATS2, -22:40816556..+12:48194605 (distance: 0 nt)
  Donor exon: NM_002490.exon1/3
  Acceptor exon: NM_023071.exon10/14

```
 N2  GCCTGGTATCGGGAGGTGCCGAACACTG  TTTAATGGATCGAGAAGTGGCG
```

- NFRKB–DMKN, -11:129269464..-19:40680280 (distance: 0 nt)
  Donor exon: NM_001143835.exon2/27
  Acceptor exon: NM_033317.exon16/16, NM_001035516.exon5/5, NM_001126059.exon11/11, NM_001126061.exon13/13

```
 N2  AGGAAATTGACACCTGGCGTG  GCAATTTCTTGCAACCACCACCGAGGCCC
```

- NISCH–ABCC2, +3:52467900..+10:101580054 (distance: 0 nt)
  Donor exon: NM_007184.exon3/21
  Acceptor exon: NM_000392.exon20/32

```
UHR  TGGCCCACTTCTTGCATTTTCACTTCTAT  TCCATGATGGCAGTGAAGAAG
```

- NSUN5–DENND4B, -7:72359327..-1:152170164 (distance: 0 nt)
  Donor exon: NM_018044.exon4/9, NM_148956.exon4/10, NM_001168347.exon4/10, NM_001168348.exon4/9
  Acceptor exon: NM_014856.exon25/28

```
 N1  GTTTCTCCTATCAGGGTCGGGCTTCCAG  GCCCCATCTCCTTGGCTAACCC
```

- NUP210–SLC25A3, -3:13368379..+12:97515765 (distance: 0 nt)
  Donor exon: NM_024923.exon20/40
  Acceptor exon: NM_213611.exon3/7, NM_002635.exon4/8, NM_005888.exon4/8

```
UHR  GGCCAGGGGTGTCGCCATG  GTGGACCCCCAAAAGTACAAGGGCATATTTA
```

- NUP214–XKR3, +9:133064223..-22:15668973 (distance: 0 nt)
  Donor exon: NM_005085.exon29/36
  Acceptor exon: NM_175878.exon2/4

```
UHR  GCAACCTCTGGGTTCAGCTTTTGCCAAGCTTCAG  CACCCTGAGAATGGAG
```

- OCIAD1–A2BP1, +4:48528023..+16:7508150 (distance: 0 nt)
  Donor exon: NM_001079841.exon1/8, NM_017830.exon1/9, NM_001079840.exon1/8
  Acceptor exon: NM_018723.exon7/18, NM_145891.exon2/13, NM_145892.exon2/13, NM_145893.exon2/14, NM_001142333.exon7/17, NM_001142334.exon3/14

```
HBR  CTTGCAGTCGCCTGCTGCTGTCGTCGGGAG  GGTAATCAGGAAGCAGCCGC
```

- OGDHL–PC, -10:50623396..-11:66373417 (distance: 0 nt)
  Donor exon: NM_018245.exon12/23, NM_001143996.exon11/22, NM_001143997.exon10/21
  Acceptor exon: NM_022172.exon20/21, NM_000920.exon21/22, NM_001040716.exon22/23

```
 N3  CCTGCAGGAGTTTGAG  GTGGAGCTGGAGCGGGG
```

- OGT–RBM22, +X:70691167..-5:150058386 (distance: 0 nt)
  Donor exon: NM_181672.exon6/22, NM_181673.exon6/22
  Acceptor exon: NM_018047.exon4/11

```
 N2  CACGCATTTTTGACAG  ATCTGTGCCAGGCCATTCACAGTGTTTCGCTGGT
 N2  CACGCATTTTTGACAG  ATCTGTGCCAGGCCATTCACAGTGTTTCGCTGGT
```

- ORMDL1–COL18A1, -2:190357240..+21:45712584 (distance: 0 nt)
  Donor exon: NM_016467.exon1/5, NM_001128150.exon1/4
  Acceptor exon: NM_030582.exon2/41, NM_130445.exon3/42, NM_130444.exon2/41

```
 N1  GCGGCCCGGAGTGGGGCGGCGG  AGCGCATCAGCGAGGAGGTGGGGCTGCT
```

- PACRGL–UBAP2L, +4:20318591..+1:152498077 (distance: 0 nt)
  Donor exon: NM_145048.exon4/8
  Acceptor exon: NM_001127320.exon21/25, NM_014847.exon21/27

```
UHR  CTAAAGGAGGTATTCCTTGCAG  CCACAAGTATATGGTTATGATGACTTGC
```

- PAIP1–MPHOSPH8, -5:43578863..+13:19143346 (distance: 0 nt)
  Donor exon: NM_006451.exon4/11, NM_183323.exon4/11, NM_182789.exon4/11
  Acceptor exon: NM_017520.exon13/14

```
 N1  TTCCGCCAATTGCTACTTCAAAG  GACAGTCATTTTGTTTACTCATTCAGC
```

- PAK2–FBXO31, +3:197994101..-16:85951473 (distance: 0 nt)
  Donor exon: NM_002577.exon2/15
  Acceptor exon: NM_024735.exon2/9, NR_024568.exon3/10

  `HBR  CTCCATATTCTCAGGCACAGAGAAAG  AGTATGGTGTTTGCGAAAACTTGC`

- PAPOLA–BRE, +14:96061481..+2:28317694 (distance: 0 nt)
  Donor exon: NM_032632.exon4/22
  Acceptor exon: NM_004899.exon9/13, NM_199191.exon9/12, NM_199192.exon9/13, NM_199193.exon10/14, NM_199194.exon10/13

  `UHR  GGATCTTACAGATTAGGAGTGCATACAAAAG  GTGCAGTACGTGATTCAAG`

- PARD3–ARHGAP10, -10:35025252..+4:148998154 (distance: 0 nt)
  Donor exon: NM_019619.exon2/25
  Acceptor exon: NM_024605.exon5/23

  `N1  ATGACATTCTTTGTGATGTAGCAGACGATAAAGACAGA  GAAGAAAAAAAGAAGTTTGACAAAGAGACAGAAAAGA`

- PBX3–CHTF18, +9:127550723..+16:783145 (distance: 0 nt)
  Donor exon: NM_006195.exon2/9, NM_001134778.exon2/9, NR_024122.exon2/8, NR_024123.exon2/7
  Acceptor exon: NM_022092.exon14/22

  `UHR  CTGTGTGAGATCAAAGAGAAAACAG  TTCCTGTACAGCCGGGGCCAGCGGG`

- PCDHGC3–PCOLCE, +5:140835897..+7:100040650 (distance: 0 nt)
  Donor exon: NM_032403.exon1/4
  Acceptor exon: NM_002593.exon4/9

  `UHR  GGCCTGGAGGAGCGGACTG  AGCACCAATTTTGCGGGGGGCGGCTGGAGAA`

- PDCD11–SMARCA4, +10:105154930..+19:11012983 (distance: 0 nt)
  Donor exon: NM_014976.exon5/36
  Acceptor exon: NM_003072.exon30/35, NM_001128844.exon31/36, NM_001128847.exon28/33, NM_001128848.exon28/33, NM_001128849.exon31/36

  `UHR  CTGAGGCCCTGAAGCCTGGCATG  GCCATCGAGGAGGGCACGCTGGAGGAG`

- PDGFRB–SCRN1, -5:149515153..-7:29961501 (distance: 0 nt)
  Donor exon: NM_002609.exon1/23
  Acceptor exon: NM_014766.exon3/8, NM_001145515.exon2/7, NM_001145513.exon3/8, NM_001145514.exon3/8

  `HBR  CCACACCAGAAGCCATCAGCAGCAAG  TGCACTTACATTTCAATCGACCAA`

- PI4KA–GCC2, -22:19497646..+2:108464585 (distance: 0 nt)
  Donor exon: NM_058004.exon8/55
  Acceptor exon: NM_181453.exon10/23, NR_028063.exon9/22

  `HBR  TGGAAATGCTTCGGGAACTCTTAAACCTG  AATCTTTTATTAGAATATGAA`

- PPP2R1A–HDAC7, +19:57385239..-12:46482324 (distance: 0 nt)
  Donor exon: NM_014225.exon1/15
  Acceptor exon: NM_015401.exon2/26, NM_001098416.exon2/25

  `N2  GACGAACTCCGCAATGAGGACGTTCAG  ATGGGACCCAGGTGAGCCCGGGT`

- PROSC–ADIPOR2, +8:37743031..+12:1763307 (distance: 0 nt)
  Donor exon: NM_007198.exon4/8
  Acceptor exon: NM_024551.exon7/8

  `HBR  CAGAAACAAAATGTCAACAAATTGATGG  GAGTGTTTTTGGGCCTAGGCCT`

- PSMC3–CDH18, -11:47403994..-5:20017044 (distance: 0 nt)
  Donor exon: NM_002804.exon2/12
  Acceptor exon: NM_004934.exon2/13, NM_001167667.exon2/13

  `HBR  CACACGGCTGCTGGACAGTGAGATCAAG  ATAGACTGAAGAGGAGCAAGAA`

- PTDSS2–KIFC3, +11:469152..-16:56389695 (distance: 0 nt)
  Donor exon: NM_030783.exon4/12
  Acceptor exon: NM_005550.exon2/19, NM_001130100.exon2/20

  `UHR  TCATCTTTATACTCTTCCAG  GCTGCCTGGTGCCCCGAGGAGGCTGCTGAG`

- PTPN2–ZMYND11, -18:12874072..+10:215934 (distance: 0 nt)
  Donor exon: NM_080422.exon1/10, NM_080423.exon1/9, NM_002828.exon1/9
  Acceptor exon: NM_006624.exon2/15, NM_212479.exon2/14, NM_001161482.exon2/15

  ```
  HBR  CAGCGTCGCTGGCAGCCGCTGTACTTG  CTAAAGAAGTAAACAGGTCATGG
  ```

- PTPRK–FSCN1, -6:128883097..+7:5609414 (distance: 0 nt)
  Donor exon: NM_002844.exon1/30, NM_001135648.exon1/31
  Acceptor exon: NM_003088.exon2/5

  ```
  UHR  CAAGGCCAGTTCTCCGCAG  GTATGGACCTGTCTGCCAATCAGGACGAGGA
  ```

- PTRH2–U2AF2, -17:55139514..+19:60871685 (distance: 0 nt)
  Donor exon: NM_016077.exon1/2
  Acceptor exon: NM_007279.exon8/12, NM_001012478.exon8/12

  ```
  UHR  GCGCGAGTGAGGAAAGGAGGTACTGTAG  GGGTTGTGTCCACTGTGGTCCC
  ```

- RABGAP1L–PPP3CA, +1:172395418..-4:102336296 (distance: 0 nt)
  Donor exon: NM_014857.exon1/21
  Acceptor exon: NM_000944.exon2/14, NM_001130691.exon2/13, NM_001130692.exon2/12

  ```
  HBR  AGCGCCCGCGGAGACGTGAAGAG  CTGTTCCATTTCCTCCAAGTCACCGGC
  ```

- RCN2–ACSS3, +15:75015118..+12:80069754 (distance: 0 nt)
  Donor exon: NM_002902.exon3/7
  Acceptor exon: NM_024560.exon6/16

  ```
  UHR  GGATGATGCAGAAGAGGAGTCCTTTAGGAAG  GGTGTGATTAGGCCCACTG
  ```

- RFC5–HNRNPM, +12:116939080..+19:8439658 (distance: 0 nt)
  Donor exon: NM_007370.exon1/11, NM_181578.exon1/12, NM_001130112.exon1/12, NM_001130113.exon1/12
  Acceptor exon: NM_031203.exon10/17, NM_005968.exon9/16

  ```
  HBR  CGGCGACCAAGATCAGGAACCTGCCCTG  GATGAGAGGGCCTTACCAAAAG
  ```

- RNF220–PQLC3, +1:44864657..+2:11232545 (distance: 0 nt)
  Donor exon: NM_018150.exon5/15
  Acceptor exon: NM_152391.exon6/7

  ```
  HBR  GATGACCTCCACCATTCAGACAGATACCAG  CAAGAATAATCACAACCTTA
  ```

- ROR2–USP36, -9:93751970..-17:74346427 (distance: 0 nt)
  Donor exon: NM_004560.exon1/9
  Acceptor exon: NM_025090.exon2/20

  ```
   N1  AGTGTCCCGGACTTCAG  GCTCTGACGCCCGCTCTGCGGCTTCGGTGTTTG
   N1  AGTGTCCCGGACTTCAG  GCTCTGACGCCCGCTCTGCGGCTTCGGTGTTTG
  ```

- RPA1–GNB1, +17:1703233..-1:1760537 (distance: 0 nt)
  Donor exon: NM_002945.exon5/17
  Acceptor exon: NM_002074.exon2/12

  ```
   N1  TGGCAATCCAGTGCCCTATAATGAAG  ACAAATTTACATGTATTGGAGACCAGACCAGAAGCCCTTCTGAATTAAG
  ```

- RPL13A–DGCR14, +19:54682713..-22:17502688 (distance: 0 nt)
  Donor exon: NM_012423.exon1/8
  Acceptor exon: NM_022719.exon9/10

  ```
  UHR  GCCGAAGATGGCGGAGGTGCAG  ATCCTGGAGCCAGGCCGCAGGGAGCGGC
  ```

- RPL18A–BAT1, +19:17833281..-6:31614611 (distance: 0 nt)
  Donor exon: NM_000980.exon2/5
  Acceptor exon: NM_080598.exon4/11, NM_004640.exon4/11

  ```
  UHR  GGGGAGATTGTCTACTGTGGGCAG  GTGTCTGTACTGGTGATGTGTCACAC
  ```

- RPL5–QPCT, +1:93078784..+2:37453329 (distance: 0 nt)
  Donor exon: NM_000969.exon7/8
  Acceptor exon: NM_012413.exon7/7

  ```
  UHR  CAAGAAAGAAGTTAAAAAGAAGAG  GTGTTCCAGTTCTGCATCTGATACCG
  ```

- RPL7A–CSNK1A1, +9:135205718..-5:148856616 (distance: 0 nt)
  Donor exon: NM_000972.exon2/8
  Acceptor exon: NM_001892.exon10/10, NM_001025105.exon11/11

```
UHR  CCTAAGAATTTTGGCATTG  GTTTCTAAGCATGAATTGAGGAACAGAAGAA
```

- RPLP2–PGK1, +11:800357..+X:77259465 (distance: 0 nt)
  Donor exon: NM_001004.exon2/5
  Acceptor exon: NM_000291.exon5/11

```
 T3  ACGACCGGCTCAACAAG  GTTAAAGCCGAGCCAG
```

- RPS10–NR3C2, -6:34497485..-4:149222100 (distance: 0 nt)
  Donor exon: NM_001014.exon4/6
  Acceptor exon: NM_000901.exon9/9, NM_001166104.exon8/8

```
 N1  GACAGAGATACCTACAGACGGAGTGCTGTGCCAC  CTGGTGAGCGACCTGC
```

- RPS18–RPL5, +6:33351638..+1:93079911 (distance: 0 nt)
  Donor exon: NM_022551.exon3/6
  Acceptor exon: NM_000969.exon8/8

```
UHR  AGAGGGCGGGAGAACTCACTGAGGATGAG  GTGGAACCGTCCCAAAATGTC
```

- RPS4X–RPL15, -X:71409807..+3:23934935 (distance: 0 nt)
  Donor exon: NM_001007.exon6/7
  Acceptor exon: NM_002948.exon3/4

```
UHR  CAACATTTTTGTTATTGGCAAG  GTTACGTTATATATAGGATTCGTGTTCG
```

- RPS6KA4–LOC727768—MAFIP—LOC100132288, +11:63884640..-Y:11770716 (distance: 0 nt)
  Donor exon: NM_003942.exon4/17, NM_001006944.exon4/17
  Acceptor exon: XM_002344445.exon7/9, XM_002344449.exon7/8, NM_001033515.exon4/4

```
 T3  GGAACACCTGCACAAG  ATGCTGCGGGAAATCAC
```

- RPS6KC1–ANKRD50, +1:211311006..-4:125819510 (distance: 0 nt)
  Donor exon: NM_012424.exon2/15
  Acceptor exon: NM_020337.exon3/5, NM_001167882.exon2/4

```
UHR  CAGAGGATGTCCAGGAG  GTGTGTTCTACTCCCTCTTCTGGGAATGAAGCC
```

- SAT1–RNPC3, +X:23711921..+1:103899179 (distance: 0 nt)
  Donor exon: NM_002970.exon3/6, NR_027783.exon3/7
  Acceptor exon: NM_017619.exon3/3

```
 N1  GAGCACTGGACTCCGGAAG  GTTTTTTTGAATCCCGTGTCCTTCAAGTGtTTTCTAACTTTGAGAGGAAGAAATTG
```

- SEC31A–C6orf62, -4:84031265..-6:24824531 (distance: 0 nt)
  Donor exon: NM_014933.exon1/27, NM_016211.exon1/25, NM_001077207.exon1/27, NM_001077208.exon1/27, NM_001077206.exon1[illegible]
  Acceptor exon: NM_030939.exon2/5

```
 T3  CGAAGGTCCTCGCCAG  AAGAAAAAAGTCAGCACT
 T3  CGAAGGTCCTCGCCAG  AAGAAAAAAGTCAGCACT
```

- SEMA4D–CCND3, -9:91191102..-6:42013110 (distance: 0 nt)
  Donor exon: NM_006378.exon15/18, NM_001142287.exon15/21
  Acceptor exon: NM_001760.exon3/5, NM_001136125.exon2/4, NM_001136017.exon3/5, NM_001136126.exon2/4

```
HBR  CTGCTGCTGTCTTCAAAGAAG  GACTGGGAGGTGCTGGTCCTAGGGAAGCT
```

- SENP2–RBX1, +3:186826875..+22:39689997 (distance: 0 nt)
  Donor exon: NM_021627.exon16/17
  Acceptor exon: NM_014248.exon3/5

```
 N1  GACAAACCTATCACATTTACTCAG  GCATAGAATGTCAAGCTAACCAGGCG
```

- SF1–ZNF638, -11:64300546..+2:71476779 (distance: 0 nt)
  Donor exon: NM_004630.exon2/13, NM_201995.exon2/14, NM_201998.exon2/13
  Acceptor exon: NM_014497.exon11/28, NM_001014972.exon11/28

```
UHR  CAAGAAAGAGCTTATATAG  CCAAAACTGGACAAGCCAAGGCATCTGTAGC
```

- SHPRH–FBXO3, -6:146273277..-11:33720206 (distance: 0 nt)
  Donor exon: NM_001042683.exon25/30, NM_173082.exon25/30
  Acceptor exon: NM_012175.exon11/11

  `HBR  AAGCAAACCAGGAGGAGGACATCCCTGTGAAG  GAAATGGGTCCTGATGAA`

- SKIV2L2–ACTR10, +5:54639732..+14:57766870 (distance: 0 nt)
  Donor exon: NM_015360.exon1/27
  Acceptor exon: NM_018477.exon11/13

  `UHR  GGGGCCTCCAGGGTCTGCAGACAAGGCAGG  AGATTCAGTTGTGGAAATTC`

- SLC14A2–RALYL, +18:41466441..+8:85604089 (distance: 0 nt)
  Donor exon: NM_007163.exon5/20
  Acceptor exon: NM_173848.exon2/9, NM_001100391.exon2/9, NM_001100392.exon3/10, NM_001100393.exon2/9

  `HBR  TTCACAGCCATGTCCTG  GATTAAAGCAAGGAGAGCCAATCATGACTGGCA`

- SLC39A8–SOCS2, -4:103444497..+12:92492629 (distance: 0 nt)
  Donor exon: NM_001135146.exon6/9, NM_001135147.exon6/11, NM_001135148.exon5/8, NM_022154.exon5/8
  Acceptor exon: NM_003877.exon3/3

  `UHR  TCAGTGTGGTATCTCTACAG  GATGGTACTGGGGAAGTATGACTGTTAATG`

- SMARCC2–TOMM20, -12:54861028..-1:233352310 (distance: 0 nt)
  Donor exon: NM_001130420.exon11/30, NM_003075.exon11/28, NM_139067.exon11/29
  Acceptor exon: NM_014765.exon2/5

  `UHR  AGAAGAGGTGACACTTCCCAAAACAG  GAAGAAAGAAACAGAAGCTTGCCA`

- SMARCD3–RNPS1, -7:150603133..-16:2253289 (distance: 0 nt)
  Donor exon: NM_001003802.exon2/14, NM_003078.exon2/14
  Acceptor exon: NM_006711.exon4/8, NM_080594.exon4/8

  `HBR  CACCCACCCACCGTGGTACAG  GTCTCGGTCCAGCTCGACTTCCAGCTCAG`

- SNHG3—SNHG3–RCC1-DDX11, +1:28705183..+12:31142086 (distance: 0 nt)
  Donor exon: NR_002909.exon1/3, NM_001048199.exon1/12, NR_030725.exon1/13, NR_030726.exon1/13
  Acceptor exon: NM_004399.exon18/26, NM_152438.exon18/27, NM_030653.exon18/27

  `UHR  TTCGCAGTGGTCGCTTCTTCTCCTTG  GCAGCCTCAGTCAGAGCACCCTGA`

- SNHG3—SNHG3–RCC1-AUH, +1:28707259..-9:93023095 (distance: 0 nt)
  Donor exon: NR_002909.exon2/3, NM_001048199.exon2/12, NR_030725.exon2/13, NR_030726.exon2/13
  Acceptor exon: NM_001698.exon7/10

  `N1  GATTTGTTAAGGATTCCAAGTAACTCTTATTTG  GGGGGACACAGCGATTGCCACGCGCCATTGGAATGTCCCTGG`

- SNHG3—SNHG3–RCC1-EEF1D, +1:28707259..-8:144740165 (distance: 0 nt)
  Donor exon: NR_002909.exon2/3, NM_001048199.exon2/12, NR_030725.exon2/13, NR_030726.exon2/13
  Acceptor exon: NM_032378.exon4/10, NM_001130053.exon4/10, NM_001130056.exon2/7, NM_001130057.exon2/8, NM_001960.exon2/8

  `UHR  GATTTGTTAAGGATTCCAAGTAACTCTTATTTG  CAGAAAAAATGGCTACAA`

- SNHG4—MATR3–PCBP2, +5:138641996..+12:52141066 (distance: 0 nt)
  Donor exon: NR_003141.exon3/4, NM_199189.exon3/18
  Acceptor exon: NM_001098620.exon7/14, NM_005016.exon7/15, NM_031989.exon7/15, NM_001128911.exon7/15, NM_001128912.exo
  NM_001128913.exon7/14, NM_001128914.exon7/13

  `UHR  TGACAGTCTGCATGTGCAGTTTTCAG  AGTACAGGGGCTCAGGTCCAGGTG`

- SNRNP25–CAPZB, +16:44058..-1:19538698 (distance: 0 nt)
  Donor exon: NM_024571.exon1/5
  Acceptor exon: NM_004930.exon9/9

  `T3  CGATCTGCCGATCCAG  GTCTGTGCAGACTTTTG`

- SNX29–UFM1, +16:12201041..+13:37826376 (distance: 0 nt)
  Donor exon: NM_001080530.exon7/14
  Acceptor exon: NM_016617.exon3/6

  `HBR  AGAGAAGGTCAAACAGCTGAAG  ACTCAGTGTTCCTGAAAGTACACCTTTC`

- SNX5–DPH1, -20:17876130..+17:1889799 (distance: 0 nt)
  Donor exon: NM_014426.exon11/13, NM_152227.exon12/14
  Acceptor exon: NM_001383.exon7/13

  ```
   N1  TCCGAATCTGCAAAAGAAG  GTATCTTGGAGATGGCCGCTTCCATCTGGAG
  ```

- SOLH–NOC4L, +16:523999..+12:131201479 (distance: 0 nt)
  Donor exon: NM_005632.exon2/14
  Acceptor exon: NM_024078.exon10/15

  ```
  UHR  GCTTGCTGCAGCTTCAG  GGGGGGCCCTCAGCCTCTTGGCCTTGAACGGGC
  ```

- SPOCK3–SMAD9, -4:168158075..-13:36352012 (distance: 0 nt)
  Donor exon: NM_016950.exon5/12, NM_001040159.exon4/11
  Acceptor exon: NM_001127217.exon2/7, NM_005905.exon2/6

  ```
  HBR  GTCTGCATTAGTCACCGGAGGCTTACACACAG  AGTTGGCCACCCTGTTCA
  ```

- SSU72–BCAS3, -1:1499721..+17:56116125 (distance: 0 nt)
  Donor exon: NM_014188.exon1/5
  Acceptor exon: NM_017679.exon3/24, NM_001099432.exon3/25

  ```
   N2  TGGAGGCGCACAACATCCTCAG  AGAGCAGTCCTACATGGAAAGTGTTGTG
  ```

- ST6GALNAC1–WFDC2, -17:72151185..+20:43541996 (distance: 0 nt)
  Donor exon: NM_018414.exon1/9
  Acceptor exon: NM_006103.exon3/4

  ```
   N1  TCTTTTATTAAGGAGCCTCAAACAAAGCCTTCCAG  ATAAGGAGGGTTCCTGCCCCCAGGTGAACATTAACTTTCC
  ```

- ST8SIA4–UBB, -5:100249946..+17:16225941 (distance: 0 nt)
  Donor exon: NM_005668.exon3/5
  Acceptor exon: NM_018955.exon2/2

  ```
  UHR  TGACAGTCACAATTTTGTAATAAG  GTCAAAATGCAGATCTTCGTGAAcAC
  ```

- STAG3L4–TOX2, +7:66412137..+20:42035421 (distance: 0 nt)
  Donor exon: NM_022906.exon4/5
  Acceptor exon: NM_032883.exon3/10, NM_001098796.exon2/9, NM_001098798.exon2/8, NM_001098797.exon2/9

  ```
  HBR  AGAGCCTGTTGGAGAAACACAAAGAG  TTTGATGGTGACAGTGCCTACGTG
  ```

- STON2–MURC, -14:80907085..+9:102387868 (distance: 0 nt)
  Donor exon: NM_033104.exon3/5
  Acceptor exon: NM_001018116.exon2/2

  ```
  UHR  GAGTGGCCAGGCTTCTGGGGCTGACTCAACTG  GAGAAGTTTCGGTGTCCG
  ```

- TBC1D5–ECHDC1, -3:17758977..-6:127693868 (distance: 0 nt)
  Donor exon: NM_001134380.exon1/23
  Acceptor exon: NM_001139510.exon2/6, NM_001002030.exon2/6, NM_018479.exon2/5

  ```
   N2  GGGAGCCCTTCTGCGCCACAG  AAATGGCGAAAAGTCTTTTGAAGACAGCC
  ```

- TDP1–SAPS3, +14:89492067..+11:68111971 (distance: 0 nt)
  Donor exon: NM_018319.exon1/17, NM_001008744.exon1/16
  Acceptor exon: NM_018312.exon15/23, NM_001164160.exon16/25, NM_001164161.exon16/24, NM_001164163.exon15/23, NM_001164164.exon15/23

  ```
   T3  GTTCTGTGCGCCTCAG  GCCTTTTCTGATTATCA
  ```

- TECR–GNAS, +19:14501523..+20:56904062 (distance: 0 nt)
  Donor exon: NM_138501.exon1/13
  Acceptor exon: NM_000516.exon2/13, NM_080426.exon2/13, NM_001077488.exon2/14, NM_080425.exon2/13, NM_001077489.exon2/
  NM_016592.exon2/14, NM_001077490.exon2/13, NR_003259.exon2/13

  ```
  UHR  GCCATGAAGCATTACGAG  GTGCTGGAGAATCTGGTAAAAGCACCATTGaG
  ```

- TIA1–VDAC3, -2:70309900..+8:42382046 (distance: 0 nt)
  Donor exon: NM_022173.exon4/13, NM_022037.exon4/12
  Acceptor exon: NM_005662.exon10/10, NM_001135694.exon11/11

  ```
  UHR  GCAGTCAAAAGAAAGATACAAGCA  GAGTCAAATTGACTTTATCAGCTTTA
  ```

- TIMELESS–RPL13A, -12:55108287..+19:54684919 (distance: 0 nt)
  Donor exon: NM_003920.exon13/29
  Acceptor exon: NM_012423.exon2/8

  `UHR  GCCGTGGGAACCTGGTGGTGCAG  GTCCTGGTGCTTGATGGTCGAGGCCAT`

- TIMM9–LOC731751—PRKDC, -14:57960479..-8:48980089 (distance: 0 nt)
  Donor exon: NM_012460.exon3/6
  Acceptor exon: XM_001129414.exon26/85, NM_001081640.exon26/85, NM_006904.exon26/86

  `UHR  GGAAACTCATGCATCAGAAAAG  GTGACAAGGCAACTGTATGAGCCACTAG`
  `UHR  GGAAACTCATGCATCAGAAAAG  GTGACAAGGCAACTGTATGAGCCACTAG`

- TMEM223–PLA2G4A, -11:62315727..+1:185191857 (distance: 0 nt)
  Donor exon: NM_001080501.exon1/2
  Acceptor exon: NM_024420.exon14/18

  `UHR  GGTCTGGCCGTCGGCTGCGGCGCCATCG  GCACTGAAAATGAAGATGCTGG`

- TMEM49–UBR4, +17:55206028..-1:19293220 (distance: 0 nt)
  Donor exon: NM_030938.exon7/12
  Acceptor exon: NM_020765.exon95/106

  `UHR  GCTGGAACATGCAGAGTCTGCACAA  GGCAACCTCCTCCTGACAGGTGACA`

- TMPRSS2–CTBP1, -21:41801878..-4:1225307 (distance: 0 nt)
  Donor exon: NM_005656.exon1/14
  Acceptor exon: NM_001012614.exon2/10

  `N3  CGCCTGGAGCGCGGCAG  TAAGACTTTGACCCCG`

- TMPRSS2–C11orf60, -21:41783304..-11:117935789 (distance: 0 nt)
  Donor exon: NM_005656.exon4/14, NM_001135099.exon4/14
  Acceptor exon: NM_020153.exon3/13, NM_001168618.exon3/12

  `N2  GGCCGCTGGCCTACTCTGGAAGTTCA  GACTTCCTCGTTCTGCCCATCTTC`

- TPCN2–MED24, +11:68588011..-17:35441399 (distance: 0 nt)
  Donor exon: NM_139075.exon7/25
  Acceptor exon: NM_014815.exon11/26, NM_001079518.exon10/25

  `HBR  CGGAATGCTGCTGTTCGCTGGTGGGAAG  GACTTCACTGAGGATGTCAACT`

- TRA2A–BANF1, -7:23527851..+11:65527282 (distance: 0 nt)
  Donor exon: NM_013293.exon2/8
  Acceptor exon: NM_003860.exon2/3, NM_001143985.exon2/3

  `N2  CAAGATCAAAATCCAG  ATTAAGCCTGATCAAGATGACAACCTCCCAAAAG`

- TRIP12–CCDC52, -2:230494840..-3:114701119 (distance: 0 nt)
  Donor exon: NM_004238.exon1/41
  Acceptor exon: NM_144718.exon4/18

  `UHR  CCGGACCGGAGACTTTGGGGCCTAACTAG  GTACGCCGTCATGAAATACAC`

- TTBK2–RSRC2, -15:40952101..-12:121561688 (distance: 0 nt)
  Donor exon: NM_173500.exon3/15
  Acceptor exon: NM_198262.exon8/11, NM_198261.exon8/11, NM_023012.exon7/10

  `N1  AAGTTGCTGTTTTGAAAAAGCTGCAAG  GTTGGAAAGGGCAAAGAAATTAC`

- TTYH1–XKR8, +19:59625396..+1:28162595 (distance: 0 nt)
  Donor exon: NM_020659.exon4/14, NM_001005367.exon4/13
  Acceptor exon: NM_018053.exon2/3

  `UHR  AAATGTGTCCTTTGTGGAGGAGTACAG  GTGCGTGCAGGAGCTGCGGCAGG`

- TULP4–PDE7A, +6:158802749..-8:66857632 (distance: 0 nt)
  Donor exon: NM_020245.exon6/14, NM_001007466.exon6/13
  Acceptor exon: NM_002603.exon2/13, NM_002604.exon2/12

  `UHR  CTGGACACTCTCGTGCAG  AGGCGTGGAGCTATTTCCTATGACAGTTCTGA`

- TXN–TPM3, -9:112046879..-1:152412078 (distance: 0 nt)
  Donor exon: NM_003329.exon4/5
  Acceptor exon: NM_153649.exon4/8, NM_152263.exon5/10, NM_001043351.exon4/8, NM_001043352.exon4/8, NM_001043353.exon4/

  `N1 AGTGTGAAGTCAAATGCATGCCAACATTCCAGTTTTTTAAGAAGGGACAAAAG GTGGCTCGTAAGTTGGTGATCA`

- UBA1–GATM, +X:46938367..-15:43449011 (distance: 0 nt)
  Donor exon: NM_003334.exon1/26
  Acceptor exon: NM_001482.exon3/9

  `HBR CGGGGAACCGGCATTG GCCAACACATATGAAAAGTACTGGCCATTTTACC`

- UBE2V1–TBX2, -20:48163051..+17:56833827 (distance: 0 nt)
  Donor exon: NM_001032288.exon1/4
  Acceptor exon: NM_005994.exon2/7

  `UHR GGCAGCCACCACGGGCTCGG GCGGATGTTCCCCCCCTTCAAGGTGCGAGT`

- UCHL3–SYCP2, +13:75076964..-20:57877375 (distance: 0 nt)
  Donor exon: NM_006002.exon8/9
  Acceptor exon: NM_014258.exon36/44

  `UHR CTAGTGATGAAACTTTATTAGAG GAAAGTCATTTAGCATCTTCATTATCC`

- UGDH–PATZ1, -4:39199366..-22:30068946 (distance: 0 nt)
  Donor exon: NM_003359.exon2/12
  Acceptor exon: NM_014323.exon2/5, NM_032051.exon2/3, NM_032052.exon2/5, NM_032050.exon2/4

  `N2 CCTACACTTCCTATTTATGAG GCCTGATCACTTGAACGGACATATCAAGC`

- UNC13A–PDIA2, -19:17620240..+16:274388 (distance: 0 nt)
  Donor exon: NM_001080421.exon16/44
  Acceptor exon: NM_006849.exon2/11

  `HBR GGACCTGCTCAACGCCGACTGCCTGCAGC ATGCCCCGTGGTGTGGGCACT`

- URB1–RPS18, -21:32639554..+6:33351720 (distance: 0 nt)
  Donor exon: NM_014825.exon23/39
  Acceptor exon: NM_022551.exon4/6

  `UHR TCACACCCCAAGCAGTCACAAGAG GTGGAACGTGTGATCACCATTATGCA`

- USP11–DNAJB6, +X:46977562..+7:156895250 (distance: 0 nt)
  Donor exon: NM_004651.exon1/21
  Acceptor exon: NM_058246.exon9/10

  `N2 GCGAGAACGTCCACTGCGcGCCGGCGAAAGCTG GTGTGGCCGACGACGAT`

- USP18–ZNF638, +22:17012989..+2:71476779 (distance: 0 nt)
  Donor exon: NM_017414.exon1/11
  Acceptor exon: NM_014497.exon11/28, NM_001014972.exon11/28

  `UHR GCAGCGGAGGCTGGACGCTTGCATGGCGCTTGAG CCAAAACTGGACAAGC`

- USP22–ATP1B3, -17:20861847..+3:143127063 (distance: 0 nt)
  Donor exon: NM_015276.exon5/13
  Acceptor exon: NM_001679.exon7/7

  `HBR GTGAGATGTCCTCACTGTTTCAGGAG GTTGGGTATCTACAGCCATTGGTT`

- VOPP1–PICALM, -7:55607458..-11:85420301 (distance: 0 nt)
  Donor exon: NM_030796.exon1/5
  Acceptor exon: NM_007166.exon2/20, NM_001008660.exon2/20

  `HBR CGGCGCTGCTGCTCGGGCTGCTCTTGGAG ACTTAATTCAGTGCACAAATG`

- VPS36–RERE, -13:51890128..-1:8477809 (distance: 0 nt)
  Donor exon: NM_016075.exon11/14
  Acceptor exon: NM_012102.exon11/24, NM_001042681.exon10/23

  `N2 TGCTGGAAGCACTGAAATTACCTCTCAG GAGCATGGCGGCATTTGCAGGA`

- WASL–EPS15, -7:123175908..-1:51647956 (distance: 0 nt)
  Donor exon: NM_003941.exon1/11
  Acceptor exon: NM_001159969.exon2/13, NM_001981.exon14/25

N1 CTTTCCTCGGCAAGAAATGTGTG GATCTTCAAGATGAAGTTCAAAGGGAG

- WDR27–MAML1, -6:169814276..+5:179124933 (distance: 0 nt)
  Donor exon: NM_182552.exon3/25
  Acceptor exon: NM_014757.exon2/5

UHR AGAGAGAAAGTACTTCAAG CATCTTCATGATACAGTTAAGAGGAATCTTG

- WDR47–TXNDC15, -1:109325878..+5:134251284 (distance: 0 nt)
  Donor exon: NM_014969.exon13/15, NM_001142550.exon13/15, NM_001142551.exon13/15
  Acceptor exon: NM_024715.exon2/5

HBR GTTGTTGGCACAACATTTCATGGAACTG TTGCAGAGGAAAGTGGTCGCTT

- XPO7–RAB37, +8:21833245..+17:70236990 (distance: 0 nt)
  Donor exon: NM_001100161.exon1/28, NM_015024.exon1/28
  Acceptor exon: NM_175738.exon2/9

HBR GGAGCAAAATGGCGGATCATGTGCAG ACCATCCTGGTGGGTGACAGTGGT

- ZDHHC8–UBL5, +22:18507415..+19:9800268 (distance: 0 nt)
  Donor exon: NM_013373.exon4/11
  Acceptor exon: NM_024292.exon3/5, NM_001048241.exon3/5

N1 AGGGGCTGGGAGCCGCGCACACCACCATCAC CACGGATGATACCATCGGG
N1 AGGGGCTGGGAGCCGCGCACACCACCATCAC CACGGATGATACCATCGGG

- ZFYVE21–TFDP1, +14:103252069..+13:113333939 (distance: 0 nt)
  Donor exon: NM_024071.exon1/7
  Acceptor exon: NM_007111.exon5/12

UHR AGTGGGTCCCGGACAAGGAG GTAATTGGTACGCCTCAGAGACCGGCAGCG

- ZHX2–COG5, +8:123863366..-7:106976013 (distance: 0 nt)
  Donor exon: NM_014943.exon1/4
  Acceptor exon: NM_006348.exon4/22, NM_181733.exon4/21, NM_001161520.exon4/21

N1 CGGGTCCCCGCGTTCACAGCCCCAGCGCAG GTGTTCTTCAGATGATGCAG

- ZNF26—LOC100287515–DDX43, +12:132093827..+6:74172808 (distance: 0 nt)
  Donor exon: NM_019591.exon2/4, XM_002343887.exon2/5
  Acceptor exon: NM_018665.exon7/17

UHR CATAACCTGATATCAGTGG TCACAGGCATGGCCCATTGTGTTGCAAGGAA

- ZNF280D–ZNF74, -15:54745885..+22:19089667 (distance: 0 nt)
  Donor exon: NM_017661.exon16/22, NM_001002843.exon15/21
  Acceptor exon: NM_003426.exon5/5, NR_003253.exon4/4

HBR CTGTAGCAAAGCCTATGTAAATCATATGATGAG AATGGGAGCTGAAGGCG

- ZNF382–RABGAP1, +19:41790364..+9:124867448 (distance: 0 nt)
  Donor exon: NM_032825.exon2/5
  Acceptor exon: NM_012197.exon14/26

HBR TTCATCTAGAAATCTCAAAGCCATGTCTCAG GAGTCTCCCCAGGACAGTG

- ZNF461–RHOA, -19:41839190..-3:49375063 (distance: 0 nt)
  Donor exon: NM_153257.exon4/6
  Acceptor exon: NM_001664.exon4/5

N1 GTGAGGGAAGAGACAGGAAGATGGTGCCCAG AAAACATCCCAGAAAAGTGGACCCCAGAAGTCAAGCATTTCTGT

- ZNF7–CKMT2, +8:146033696..+5:80589308 (distance: 0 nt)
  Donor exon: NM_003416.exon4/5
  Acceptor exon: NM_001825.exon8/11, NM_001099735.exon7/10, NM_001099736.exon7/10

N2 CAGAGGCACCAAGGACCTCCAAGACAG GCATAATTATGATAAGACATTTC

- ZNF7–NOC4L, +8:146033696..+12:131201479 (distance: 0 nt)
  Donor exon: NM_003416.exon4/5
  Acceptor exon: NM_024078.exon10/15

N2 CAAGGACCTCCAAGACAG GGGGGGCCCTCAGCCTCTTGGCCTTGAACGGG
