# Supplementary material for: Deep RNA sequencing analysis of readthrough gene fusions in human prostate adenocarcinoma and reference samples
Source: BMC Med Genomics. 2011 Jan 24;4:11. doi: 10.1186/1755-8794-4-11 (PMC3041646; doi:10.1186/1755-8794-4-11)
Supplement: Additional file 6 — Sample information and pathology evaluation. [file 1755-8794-4-11-S6.PDF]

Additional file 6 — Sample information and pathology evaluation

Sample ID	Sample Name	Diagnosis	Matching tumor ID	Vendor	Tumor content	RNA-Seq ID	SLC45A3–ELK4	IRS2–NUFIP1	SEC31A–C6orf62
111995	HF-23751-(1)	Prostate adenocarcinoma	-	UroPath Pty LLC, Australia	95	T1	Y	Y	Y
112038	HF-23261-(1)	Prostate adenocarcinoma	-	SeraCare LifeSciences, MA	70	T2	Y	Y	Y
113061	HF-24126-(1)	Prostate adenocarcinoma	-	Indivumed, Germany	80	T3	Y	Y	Y
111976	HF-23732	Matched normal sample	111995	UroPath Pty LLC, Australia	-	N1	Y	Y	Y
112035	HF-23260	Matched normal sample	112038	SeraCare LifeSciences, MA	-	N2	Y	Y	Y
113063	HF-24127-(1)	Matched normal sample	113061	Indivumed, Germany	-	N3	Y	Y	Y
112861	HF-24061-(1)	Matched normal sample	112857	Indivumed, Germany	-	-	Y	-	-
112885	HF-24069-(1)	Matched normal sample	112881	Indivumed, Germany	-	-	Y	-	-
112897	HF-24073-(1)	Matched normal sample	112893	Indivumed, Germany	-	-	Y	-	-
112903	HF-24075-(1)	Matched normal sample	112899	Indivumed, Germany	-	-	Y	-	-
112921	HF-24081-(1)	Matched normal sample	112917	Indivumed, Germany	-	-	Y	-	-
112991	HF-24131-(1)	Matched normal sample	112989	Indivumed, Germany	-	-	Y	-	-
113039	HF-24115-(1)	Matched normal sample	113037	Indivumed, Germany	-	-	Y	-	-
113051	HF-24121-(1)	Matched normal sample	113049	Indivumed, Germany	-	-	Y	-	-
113059	HF-24125-(1)	Matched normal sample	113057	Indivumed, Germany	-	-	Y	-	-
113083	HF-24097-(1)	Matched normal sample	113081	Indivumed, Germany	-	-	Y	-	-
113091	HF-24101-(1)	Matched normal sample	113089	Indivumed, Germany	-	-	Y	-	-
113099	HF-24105-(1)	Matched normal sample	113097	Indivumed, Germany	-	-	Y	-	-
113103	HF-24107-(1)	Matched normal sample	113101	Indivumed, Germany	-	-	Y	-	-
112789	HF-24037-(1)	Matched normal sample	112785	Indivumed, Germany	-	-	Y	-	-
112042	HF-23263-(1)	Matched normal sample	112040	SeraCare LifeSciences, MA	-	-	Y	-	-
112034	HF-22800-(1)	Matched normal sample	112032	Asterand, MI	-	-	Y	-	-
111870	HF-23730-(1)	Matched normal sample	111880	UroPath Pty LLC, Australia	-	-	Y	-	-
111866	HF-23728-(1)	Matched normal sample	111878	UroPath Pty LLC, Australia	-	-	Y	-	-
110348	HF-22403-(1)	Matched normal sample	110350	Asterand, MI	-	-	Y	-	-
111801	HF-23247-(1)	Matched normal sample	111797	SeraCare LifeSciences, MA	-	-	Y	-	-
113097	HF-24104-(1)	Prostate adenocarcinoma	-	Indivumed, Germany	70	-	Y	Y	Y
112929	HF-24084-(1)	Prostate adenocarcinoma	-	Indivumed, Germany	80	-	-	Y	Y
112839	HF-24054-(1)	Prostate adenocarcinoma	-	Indivumed, Germany	80	-	-	Y	Y
113089	HF-24100-(1)	Prostate adenocarcinoma	-	Indivumed, Germany	80	-	Y	Y	Y
113101	HF-24106-(1)	Prostate adenocarcinoma	-	Indivumed, Germany	70	-	Y	Y	Y
113105	HF-24108-(1)	Prostate adenocarcinoma	-	Indivumed, Germany	60	-	-	Y	Y
111880	HF-23749-(1)	Prostate adenocarcinoma	-	UroPath Pty LLC, Australia	60	-	Y	Y	Y
113001	HF-24136-(1)	Prostate adenocarcinoma	-	Indivumed, Germany	80	-	-	Y	Y
113009	HF-24140-(1)	Prostate adenocarcinoma	-	Indivumed, Germany	80	-	-	Y	Y
113065	HF-24128-(1)	Prostate adenocarcinoma	-	Indivumed, Germany	60	-	-	Y	Y
110350	HF-22404-(1)	Prostate adenocarcinoma	-	Asterand, MI	90	-	Y	Y	Y
113021	HF-24146-(1)	Prostate adenocarcinoma	-	Indivumed, Germany	80	-	-	Y	Y
113013	HF-24142-(1)	Prostate adenocarcinoma	-	Indivumed, Germany	80	-	-	Y	Y
112887	HF-24070-(1)	Prostate adenocarcinoma	-	Indivumed, Germany	70	-	-	Y	Y
113025	HF-24148-(1)	Prostate adenocarcinoma	-	Indivumed, Germany	75	-	-	Y	Y
112785	HF-24036-(1)	Prostate adenocarcinoma	-	Indivumed, Germany	50	-	Y	Y	Y
112791	HF-24038-(1)	Prostate adenocarcinoma	-	Indivumed, Germany	80	-	-	Y	Y
113045	HF-24118-(1)	Prostate adenocarcinoma	-	Indivumed, Germany	70	-	-	Y	Y
112941	HF-24088-(1)	Prostate adenocarcinoma	-	Indivumed, Germany	70	-	-	Y	Y

Sample ID	Sample Name	Diagnosis	Matching tumor ID	Vendor	Tumor content	RNA-Seq ID	SLC45A3–ELK4	IRS2–NUFIP1	SEC31A–C6orf62
113017	HF-24144-(1)	Prostate adenocarcinoma	-	Indivumed, Germany	90	-	-	Y	Y
112989	HF-24130-(1)	Prostate adenocarcinoma	-	Indivumed, Germany	80	-	Y	Y	Y
113057	HF-24124-(1)	Prostate adenocarcinoma	-	Indivumed, Germany	80	-	Y	Y	Y
113049	HF-24120-(1)	Prostate adenocarcinoma	-	Indivumed, Germany	70	-	Y	Y	Y
113093	HF-24102-(1)	Prostate adenocarcinoma	-	Indivumed, Germany	85	-	-	Y	Y
112917	HF-24080-(1)	Prostate adenocarcinoma	-	Indivumed, Germany	85	-	Y	Y	Y
112827	HF-24050-(1)	Prostate adenocarcinoma	-	Indivumed, Germany	70	-	-	Y	Y
111878	HF-23747-(1)	Prostate adenocarcinoma	-	UroPath Pty LLC, Australia	95	-	Y	Y	Y
111873	HF-23742-(1)	Prostate adenocarcinoma	-	UroPath Pty LLC, Australia	60	-	-	Y	Y
111991	HF-23739-(1)	Prostate adenocarcinoma	-	UroPath Pty LLC, Australia	70	-	-	Y	Y
112040	HF-23262-(1)	Prostate adenocarcinoma	-	SeraCare LifeSciences, MA	80	-	Y	Y	Y
113716	HF-24331-(1)	Prostate adenocarcinoma	-	Cytomyx, LLC, MA	80	-	-	Y	Y
112905	HF-24076-(1)	Prostate adenocarcinoma	-	Indivumed, Germany	70	-	-	Y	Y
112923	HF-24082-(1)	Prostate adenocarcinoma	-	Indivumed, Germany	75	-	-	Y	Y
112032	HF-22799-(1)	Prostate adenocarcinoma	-	Asterand, MI	75	-	Y	Y	Y
112997	HF-24134-(1)	Prostate adenocarcinoma	-	Indivumed, Germany	80	-	-	Y	Y
113081	HF-24096-(1)	Prostate adenocarcinoma	-	Indivumed, Germany	80	-	Y	Y	Y
113077	HF-24094-(1)	Prostate adenocarcinoma	-	Indivumed, Germany	80	-	-	Y	Y
112881	HF-24068-(1)	Prostate adenocarcinoma	-	Indivumed, Germany	80	-	Y	Y	Y
113069	HF-24090-(1)	Prostate adenocarcinoma	-	Indivumed, Germany	70	-	-	Y	Y
111797	HF-23246-(1)	Prostate adenocarcinoma	-	SeraCare LifeSciences, MA	50	-	Y	Y	Y
112851	HF-24058-(1)	Prostate adenocarcinoma	-	Indivumed, Germany	80	-	-	Y	Y
112779	HF-24034-(1)	Prostate adenocarcinoma	-	Indivumed, Germany	80	-	-	Y	Y
112899	HF-24074-(1)	Prostate adenocarcinoma	-	Indivumed, Germany	80	-	Y	Y	Y
112893	HF-24072-(1)	Prostate adenocarcinoma	-	Indivumed, Germany	75	-	Y	Y	Y
112857	HF-24060-(1)	Prostate adenocarcinoma	-	Indivumed, Germany	90	-	Y	Y	Y
113037	HF-24114-(1)	Prostate adenocarcinoma	-	Indivumed, Germany	70	-	Y	Y	Y
113085	HF-24098-(1)	Prostate adenocarcinoma	-	Indivumed, Germany	70	-	-	Y	Y
112935	HF-24086-(1)	Prostate adenocarcinoma	-	Indivumed, Germany	80	-	-	Y	Y
111827	HF-23256-(1)	Prostate adenocarcinoma	-	SeraCare LifeSciences, MA	50	-	-	Y	Y
112869	HF-24064-(1)	Prostate adenocarcinoma	-	Indivumed, Germany	50	-	-	Y	Y
113005	HF-24138-(1)	Prostate adenocarcinoma	-	Indivumed, Germany	80	-	-	Y	Y
CRL-5800	NCI-H23	NSCLC Adenocarcinoma		ATCC	-	-	Y	-	-
CRL-5810	NCI-H522	NSCLC Adenocarcinoma		ATCC	-	-	Y	-	-
CRL-5889	NCI-H1703	NSCLC Adenocarcinoma, squamous cell		ATCC	-	-	Y	-	-
HTB-182	NCI-H520	Carcinoma, squamous cell		ATCC	-	-	Y	-	-
CRL-2049	DMS 79	SCLC Carcinoma		ATCC	-	-	Y	-	-
CRL-5899	NCI-H1838	NSCLC Adenocarcinoma		ATCC	-	-	Y	-	-
CRL-5875	NCI-H1563	NSCLC Adenocarcinoma		ATCC	-	-	Y	-	-
CRL-5891	NCI-H1734	NSCLCAdenocarcinoma		ATCC	-	-	Y	-	-
CCL-257	NCI-H1688	SCLC Carcinoma		ATCC	-	-	Y	-	-

Y = tested by RT-PCR
