# Supplementary material for: Deep RNA sequencing analysis of readthrough gene fusions in human prostate adenocarcinoma and reference samples
Source: BMC Med Genomics. 2011 Jan 24;4:11. doi: 10.1186/1755-8794-4-11 (PMC3041646; doi:10.1186/1755-8794-4-11)
Supplement: Additional file 7 — Primers and probes used for qRT-PCR measurements. [file 1755-8794-4-11-S7.PDF]

Additional file 7 — Primers and probes used for qRT-PCR measurements

For events that were confirmed in a pilot study, the successful set of primers and probes is shown. For events that were not confirmed, both sets are shown.

TIC or fusion	Probe/Forward primer/Reverse primer	Probe/Forward primer/Reverse primer
ADCK4–NUMBL	AGGCTTTGAAACCAAGGGCGGA<br>CAGAAGTCCAGGGACCTCAA<br>CTCCGTCCTGCAGGTTTCT	
AZGP1–GJC3	AGCTTCTTCCTGGAACGTTACTGTCGTTG<br>AGCCAACTTCAGAAGGCCA<br>ATCTCTGGGTCCAACTGGTCT	
C16orf58–NUPR1	AAAGGAGGTGGAGGCCGGAA<br>GAAGTGTTGGACATGCTGTTCC<br>GGTCACCAGTTTCCTCTCGTG	AAAGGAGGTGGAGGCCGGAA<br>GGACATGCTGTTCCCAAAGTT<br>GTTGGTGTTGGCAGCAGCT
DUS4L–BCAP29	CGGATTACTGGGACAGATGGTGTGAAGA<br>CATCAGAAGCTTAAAGGAAGCAGA<br>CAGCCCATTGGAGTGTCATT	
HDAC8–CITED1	CATCAAAGCACAACAGCTCCAGCTG<br>CGAATCCAACAAATCCTCAACT<br>TGACATCAAGTGCAGGCCTC	
IRS2–NUFIP1	AGGCCACCATCGTGAAAGAGTCTGATAAG<br>GACTTCTTGTCCCACCACTTGA<br>GCCATAGCTACTCATTAGTGAGCATA	
KRT24–NCOR1	CAATTTGCTGGTTTCTGAGATCTTCAATTATTG<br>GAGGCTAACACTGATCTGGAGAAC<br>TTCTTCTTGCGAGGGTCG	CAATTTGCTGGTTTCTGAGATCTTCAATTATTG<br>GGTCTGGAGACGGTGGATC<br>CTTCTTGCGAGGGTCGAG
LIN37–GPSN2	TCCAGAATCTCCACCTCCTCATCCTC<br>AGCGTGAATGCTCTCCCAG<br>CTCCACCTTGTCCAAGAAACAC	TCCAGAATCTCCACCTCCTCATCCTC<br>TGAATGCTCTCCCAGCTCAC<br>CCTTGTCCAAGAAACACAGCTT
MSMB–NCOA4 e2–e2	CATTCTCCTCACTGCTCTCCTGGTTGA<br>GACTTTATGCAATGCATCATGCT<br>TGCCACTCTGGTCTTGGAAG	
SEC31A–C6orf62	CCGAAGGTCCTCGCCAGAAGAA<br>CGCTGCACTAACGCAGGAT<br>GTCATGACTGGTATAACCTCAGACA	
SLC45A3–ELK4	CACTGTCCATAGCAATGAGCTGCTTCTC<br>CAGATCCTGCCCTACACACTG<br>CTGAAGAAGGAACTGCCACAG	
TMPRSS2–ERG e1–e4	CTCACTCACAACTGATAAGGCTTCCTGCC<br>TAGGCGCGAGCTAAGCAG<br>GTAGGCACACTCAAACAACGAC	
TMPRSS2–ERG e1–e5	TGCATTCATCAGGAGAGTTCCTGCC<br>TAGGCGCGAGCTAAGCAG<br>GGGCTGCCCACCATCTT	
